# Spectroscopic and Structural Study of a New Conducting Pyrazolium Salt

**DOI:** 10.3390/molecules26154657

**Published:** 2021-07-31

**Authors:** Sylwia Zięba, Agata Piotrowska, Adam Mizera, Paweł Ławniczak, Karolina H. Markiewicz, Andrzej Gzella, Alina T. Dubis, Andrzej Łapiński

**Affiliations:** 1 Institute of Molecular Physics Polish Academy of Sciences, Smoluchowskiego 17, 60-179 Poznań, Poland; zieba@ifmpan.poznan.pl (S.Z.); mizera@ifmpan.poznan.pl (A.M.); lawniczak@ifmpan.poznan.pl (P.Ł.); 2 Faculty of Materials Engineering and Technical Physics, Poznań University of Technology, Piotrowo 3, 60-965 Poznań, Poland; agata.m.piotrowska@student.put.poznan.pl; 3 Faculty of Chemistry, University of Bialystok, Ciołkowskiego 1K, 15-245 Białystok, Poland; k.markiewicz@uwb.edu.pl (K.H.M.); alina@uwb.edu.pl (A.T.D.); 4 Departament of Organic Chemistry, Poznan University of Medical Sciences, Grunwaldzka 6, 60-780 Poznań, Poland; akgzella@ump.edu.pl

**Keywords:** proton conductors, X-ray crystallography, IR and Raman spectroscopy, DSC/TGA analysis, impedance spectroscopy, hydrogen bond network, Hirshfeld surface analysis, QTAiM analysis

## Abstract

The increase in conductivity with temperature in 1*H*-pyrazol-2-ium 2,6-dicarboxybenzoate monohydrate was analyzed, and the influence of the mobility of the water was discussed in this study. The electric properties of the salt were studied using the impedance spectroscopy method. WB97XD/6-311++G(d,p) calculations were performed, and the quantum theory of atoms in molecules (QTAiM) approach and the Hirshfeld surface method were applied to analyze the hydrogen bond interaction. It was found that temperature influences the spectroscopic properties of pyrazolium salt, particularly the carbonyl and hydroxyl frequencies. The influence of water molecules, connected by three-center hydrogen bonds with co-planar tetrameters, on the formation of structural defects is also discussed in this report.

## 1. Introduction

Solid proton conductors are attractive materials due to the possibility of their application as an electrolyte in fuel cells for portable electronics and transportation [1]. These solid materials with good proton conductivity and negligible electronic conductivity are often referred to as solid electrolytes. They allow the passage of electrical current through the material by the movement of protons. According to present knowledge, the conductivity of solids by polyatomic protonic species, such as H_3_O^+^ and NH_4_^+^, is relatively low. Therefore, proton conduction based on the movement of H^+^ attracts researchers’ attention [2]. There are many advantages of facilities using solid electrolytes as compared to liquid electrolytes [3,4]; for example, the only by-products of the reaction occurring in fuel cells based on solid electrolytes are water and heat. This advantage allows the production of pollution-free electrochemical devices, such as the compact power batteries used in mobile and laptops.

Recent studies reveal the numerous requirements for a proton conductor to be used as an electrolyte in a fuel cell [5,6]. For instance, the conduction should be selective to protons, and the electrolyte must be an electrical insulator to prevent the internal short circuit of the cell. Additionally, proton conductors must be thermally stable, which prevents decomposition on heating with the loss of its conductivity. Proton conductors with thermal stability allow the construction of fuel cells with minor corrosion problems.

Some inorganic salts, such as hydrogen sulfates (CsHSO_4_), reveal high proton conductivity close to 10^−1^ S·cm^−1^ at high temperatures [7,8]. Organic polymers such as Nafion are also good proton-conducting materials, with conductivity close to ∼ 10^−1^ S·cm^−1^ [9], and are currently used in proton exchange membrane fuel cells (PEMFCs) in the USA space program and for transport applications [10,11]. Recently, heteroaromatic triphosphate, 2,4,6-triphosphono-1,3,5-triazine (TPT), was used in the synthesis of novel proton conductors (1-methyl-4-phenyl-1,2,3,6-tetrahydropyridine (MTPT), M = Ce, Zr, Fe) via polymerization with metal ions. Besides TPT, a new class of imidazole-based organic proton conductors has been developed lately [12,13]. Many proton-conducting salts are based on heterocyclic bases and aromatic acids, such as imidazolium salts of benzoic, salicylic, orthophthalic, and terephthalic acids [14,15]. Nevertheless, the hydrogen bonds formed in such solids were weak and medium strength, and their mechanism of conductivity is based on the Grotthuss mechanism. On the other hand, the number and location of the carboxylic group influence the thermal stability and electrical conductivity of the proton conductors [15]. For example, imidazolium orthophthalate and terephthalate revealed high thermal stability with values of 464 and 422 K, respectively, and imidazolium orthophthalate also presented good electrical conductivity (10^−1^ S⸱m^−1^) [14]. There are many proton-conducting salts with high proton conductivity. They fulfill only a few requirements for application as an electrolyte in a fuel cell. For example, several materials exhibit good conductivities but contain crystal water or hydrated protons. They will lose water and conductivity on heating such as hydrated acid or ion-exchanged ceramics [16,17]. It was shown that the oscillation of hydrogen atoms in hydrogen bonds corresponds to a net transport of charge. The cooperative proton transfer phenomenon, followed by reorientation of donating and accepting groups, is a Grotthuss-type conductivity [18,19]. Thus, the transfer phenomenon is similar to an internal acid–base reaction. Proton transfer needs activation energy. If the proton donating and accepting groups are chemically identical, X—H^…^X, the proton transfer is energetically cost-free. In the case where the proton donating and accepting groups are chemically different X—H^…^Y, the proton-transfer needs extra activation energy, as in the case of transfer of a proton from a NH^+^ group to carboxylic group COO^−^. Based on our previous studies on conducting materials, we consider a new conducting pyrazolium salt of hydrated 1,2,3-benzene tricarboxylic acid. It was shown previously that due to the presence of water molecules in the crystal lattice of imidazolium selenate dihydrate, the three-dimensional hydrogen bond exists [20]. There are also some inorganic acid hydrates with high proton conductivity stable up to 350 °C. These salts include, for instance, H_2_Ti_4_O_9_×1.2H_2_O or zirconium sulfate phosphonates [21]. The conductivity of such salts is related to the water content.

The main goal of this study is to extend the knowledge of a conductivity phenomenon within a new hydrated pyrazolium salt. In the first part of this study, we carried out thermal, optical, and structural analyses. In the second part, the geometrical and topological parameters of the salt under investigation were analyzed in terms of the quantum theory of atoms in molecules (QTAiM) and density functional theory (DFT) methods. In proton-conducting systems, intermolecular interactions play a crucial role. Therefore, a study of their role in the real system and theoretical models has outstanding significance. To the best of our knowledge, the QTAiM theoretical calculations and Hirshfeld Surface approach have not been applied so far to analyze the HBs’ interactions in pyrazolium hemimellitate hydrate.

## 2. Results and Discussions

### 2.1. Crystallographic Investigations

**Figure 1:**
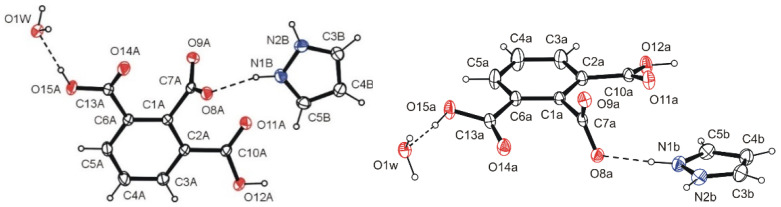
**Two** views of the molecule of PyrHem×H_2_O, showing the atomic labeling scheme. Non-H atoms are drawn as 30% probability displacement ellipsoids, and H atoms are drawn as spheres of arbitrary size.

X-ray studies confirmed that the 1*H*-pyrazol-2-ium 2,6-dicarboxybenzoate monohydrate (1:1:1) (PyrHem×H_2_O) has the character of salt, and its formula moiety is C_9_H_5_O_6_^–^, C_3_H_5_N_2_^+^, H_2_O. It was found that the carboxylate group in the anion is in the C-1 position (Figure 1). This is indicated by the found comparable bond lengths C7a−O8a (1.2534(18) Å) and C7a−O9a (1.2522(18) Å), which are intermediate between the length of the single bond (1.305(2) Å) and double C*sp*^2^–O) (1.221(1) Å) in the carboxyl group. In the 2,6-dicarboxybenzoate anion, the carboxyl groups deviate only slightly from the ring plane C1a―C6a (r.m.s.d.: 0.0022 Å). The dihedral angles (C2a-)COOH/C1a—C6a and (C6a-)COOH/C1a—C6a are 18.87(5)° and 18.14(5)°, respectively. The situation is different for the C-1 carboxylate group, which forms a dihedral angle of 79.57(6)° with the plane of the C1a—C6a ring. The position of the latter is stabilized by the hydrogen bonds N1b—H1b···O8a and N2b—H2b···O9a^i^, linking the molecules of the 2,6-dicarboxybenzoate anion and the pyrazolinium cation in centrosymmetric tetrameters (Figure 1).

**Figure 2:**
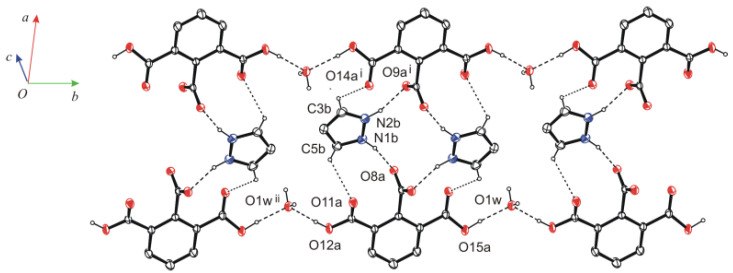
Hydrogen bonds linking molecules into tapes growing along the *b*-axis.

**Figure 3:**
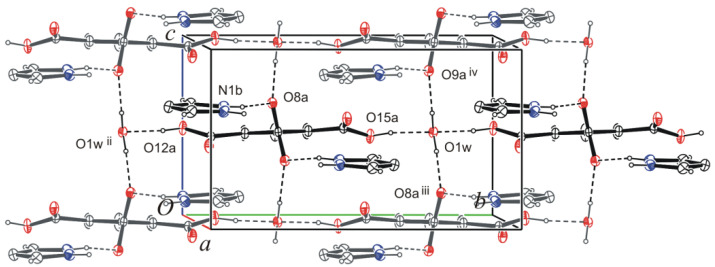
Hydrogen bonds O1w—H1wA∙∙∙O9a^iv^ and O1w—H1wB ∙∙∙O8a^iii^ linking types into layers parallel to the *bc* plane. The symmetry codes are explained in Table 1.

Co-planar tetrameters are further connected by water molecules via three-center hydrogen bonds O12a—H12a···O1w^ii^ and O15a—H15a···O1W (or O15a—H15a···O1W···H12a^ix^—C12a^ix^; (ix) *x*, -1+*y*, *z*) into tapes growing along the *b*-axis (Figure 2). In the latter bifurcated hydrogen bonds, a water oxygen atom acts twice as a proton acceptor. The tapes of molecules arranged antiparallelly one above the other are connected via water molecules by hydrogen bonds O1w—H1wB···O8a^iii^ and O1W—H1WA∙∙∙O9a^iv^ into layers parallel to the *bc* plane (Figure 3 and Figure S2, Table 1). In these hydrogen bonds, as can be seen, the oxygen atom of water molecules acts as a proton donor twice in hydrogen bonds. The layered structure (Figure S1) is additionally stabilized by non-classical three-center hydrogen bonds C3b—H3b∙∙∙O14a^i^, C3b—H3b∙∙∙O14a^v^ and C5b—H5b⋅∙∙O11a, C5b—H5b⋅∙∙O11a^vi^, as well as interactions of π(*Pyr*^viii^)···π(*Pyr*)···π(*Pyr*^vii^) stacking (*Pyr* = pyrazolinium cation) type (see Figure S3).

### 2.2. Thermal Properties Analysis

**Figure 4:**
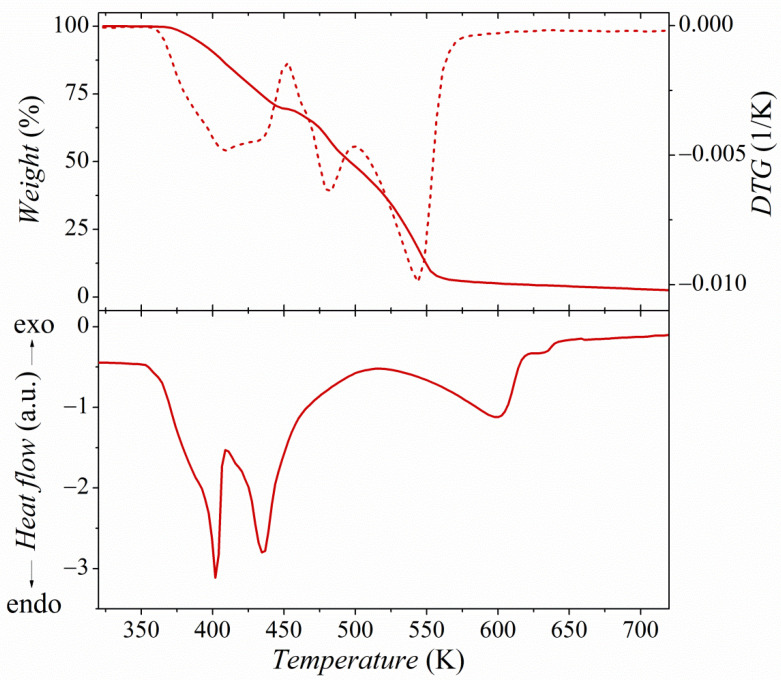
TGA/DTG curves (top panel) and DSC curves (bottom panel) of PyrHem×H_2_O.

Differential scanning calorimetry (DSC) and thermogravimetric analysis (TGA) were used to study the thermal properties of the substrates and the product. The pure hemimellitic acid (Hem×H_2_O) decomposes in a broad temperature range from 400 to 650 K (Figure S4). The endothermic peaks observed in the DSC thermogram with the minima at 440, 470, and 600 K represent dehydration, melting, and acid decomposition [22]. Pyrazole (Pyr) decomposes in one stage below 450 K (Figure S5). The DSC thermogram of pyrazole shows two sharp endothermic peaks at 340 and 470 K, attributed to its melting and decomposition. Several overlapping endothermic peaks with the minima at 400, 435, and 600 K can be observed in the DSC thermogram of the PyrHem×H_2_O (Figure 4). They are related to the dehydration, melting, and decomposition of the compound. The melting range of PyrHem×H_2_O is between 393 and 443 K. The formation of gas bubbles was noted when the sample was heated above 420 K. It seems most likely that decarboxylation of anion moiety proceeds [23]. The salt has a different and more complex decomposition profile compared to pure acid (Hem×H_2_O) and pyrazole (Pyr). It decomposes in a few consecutive overlapping stages between 350 and 650 K. These steps may be related to the dehydration, decarboxylation, and decomposition of pyrazole and acid, or its anhydride. However, it is not possible to define these stages precisely, as they overlap.

### 2.3. Intermolecular Interactions Analysis

The analysis of intermolecular interactions in the studied hydrated pyrazolium hemimellitate crystal was carried out using the Hirshfeld surface, fingerprint plots, and the quantum theory of atoms in molecules (QTAiM). The Hirshfeld surface of a molecule in a crystal is constructed by partitioning the crystal space into regions in which the electron distribution of the sum of spherical atoms of the molecule dominates the corresponding sum over the crystal. It can be described using the molecular weight-function *w*(*r*):

(1) $$w\left(r\right)=\frac{\sum_{A\in molecule}\rho_{A}\left(r\right)}{\sum_{A\in crystal}\rho_{A}\left(r\right)}=\frac{\rho_{promolecule}\left(r\right)}{\rho_{procrystal}\left(r\right)}\geq 0.5$$

where *ρ_A_*(*r*) is a spherically averaged electron density of various atoms centered on the nucleus *A*. The Hirshfeld surface is defined as the region around a molecule where the weight function ≥0.5. The contribution to electron density from the promolecule to the procrystal exceeds that of all other molecules in this region in the crystal.

**Figure 5:**
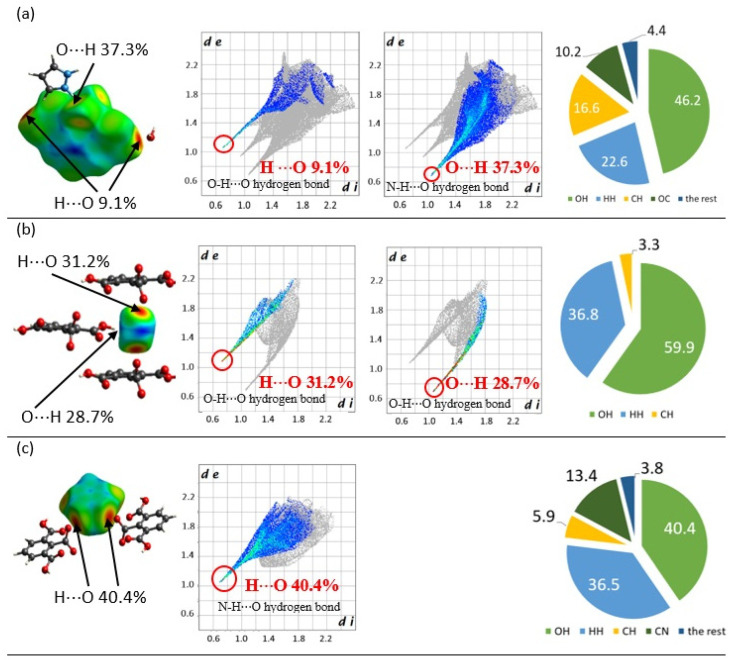
On the left, the Hirshfeld surfaces for the hemimellitate ion (**a**), the water molecule (**b**), and the pyrazolium ion (**c**) mapped with *d*_i_; mapping range: red (short distance) through green to blue (long-distance). In the middle, fingerprint plots (*d*_e_ versus *d*_i_) for these features involving specific pairs of atoms; on the right, histograms showing the percentage of different types of interactions taking place in the studied salt.

The Hirshfeld surfaces and fingerprint plots for the hemimellitic ion, the water molecule, and the pyrazolium ion are shown in Figure 5. The Hirshfeld surfaces are defined by points where the contribution in the electron density of the target molecule equals the contribution of all other molecules. The dominant interaction (H⸱⸱⸱O and O⸱⸱⸱H) corresponds to the area presented at Hirshfeld surface: red for the highest contribution through green to blue for points with smaller contribution. Based on the analysis of the Hirshfeld surface and fingerprint plots, the hydrogen bonding patterns can be identified. On the fingerprint plot, each point corresponds to a unique (*d*_i_, *d*_e_) pair. The parameters *d*_i_ and *d*_e_ present the distance from the Hirshfeld surface to the nearest nucleus outside and inside the surface, respectively [24,25]. All (*d*_i_, *d*_e_) pairs are presented in colors, which correspond to areas presented at the Hirshfeld surface: red for the highest contribution through green to blue for points pointed out the smallest contribution.

The intermolecular H∙∙∙O and O∙∙∙H contacts in N—H∙∙∙O, C–O^−…^H, and O—H∙∙∙O can be analyzed. For these bonds, the fingerprint plot shows a pair of spikes at the bottom left of the plot, the upper one (*d*_i_ < *d*_e_) associated with the donor atom, the lower one (*d*_i_ > *d*_e_) with the acceptor. The carboxylate anion is a proton-accepting center, whereas carboxyl groups are proton-accepting and proton-donating centers. The contribution of N—H⸱⸱⸱O interaction is 37.3%, whereas O—H ^…^O amounts to 9.1%. The total contribution of O⸱⸱⸱H interaction is 46.2% of all interactions between the anion and the closest environment. The water molecule plays a role as a proton donator and an acceptor; the total O⸱⸱⸱H interaction is 59.9%, and this contribution is related to the O—H⸱⸱⸱O interactions. The pyrazolium ion is connected by N—H⸱⸱⸱O with two hemimellitate ions, and the O···H interaction is responsible for over 40.4% of all interactions between the pyrazolium ion and the closest environment. The structure is stabilized by N—H⸱⸱⸱O (36.8%) and C⸱⸱⸱H (3.3%) interactions. Pyrazolium ions are also stabilized by the C—H^…^O (5.9%) interaction.

To analyze the hydrogen bond interactions, various descriptors originating from Bader’s quantum theory of “Atoms in Molecules” (AIM) are used **[26,27]**. Koch and Popelier [28] proposed criteria for the description of the D—H⋅⋅⋅A hydrogen bond (where A is a proton-acceptor center). The electron density at the bond critical point BCP (*ρ*_H__⋅⋅⋅__A_) should be within a range of 0.002–0.040 a.u. The corresponding Laplacian of electron density at the BCPs (∇^2^*ρ_BCP_*) should be within a range of 0.024–0.139 a.u. Additionally, there are energetic descriptors of BCPs such as electron energy density at BCP (*H*_C_) and its components, potential electron energy density (*V*_C_), and kinetic electron energy density (*G*_C_). The equation gives relationships between topological parameters at the critical point:

(2) $$0.25\;\nabla_{\rho_{BCP}}^{2}=2G_{C}+V_{C\;};\;H_{C}=G_{C}+V_{C}$$

The Rozas group [29] proposed a classification of hydrogen bonds using the electron energy density and Laplacian of electron density at the BCPs. Weak and moderate H-bonds are characterized by ∇^2^*ρ_BCP_* > 0 and *H*_C_ > 0, which indicates them as closed-shell interactions. Suppose *H*_C_ is negative, corresponding to the interaction between the proton and proton acceptor within the hydrogen bridge. In that case, the interaction may be treated as strong hydrogen bonds. The Laplacian of the electron density for interacting pairs of atoms is negative as well. The negative Laplacian for H-bonds indicates their covalent character. If Laplacian is positive and *H*_C_ is negative, strong hydrogen bonding is partially covalent [30]. For a very strong hydrogen bond interaction, ∇*^2^ρ*_BCP_ and *H*_C_ values are both negative. It has been shown that *ρ*_BCP_ and ∇^2^*ρ*_BCP_ correlate with HBs’ energy [31,32]. The increase in hydrogen bond strength is related to the growth of electron density at the bond critical points.

**Figure 6:**
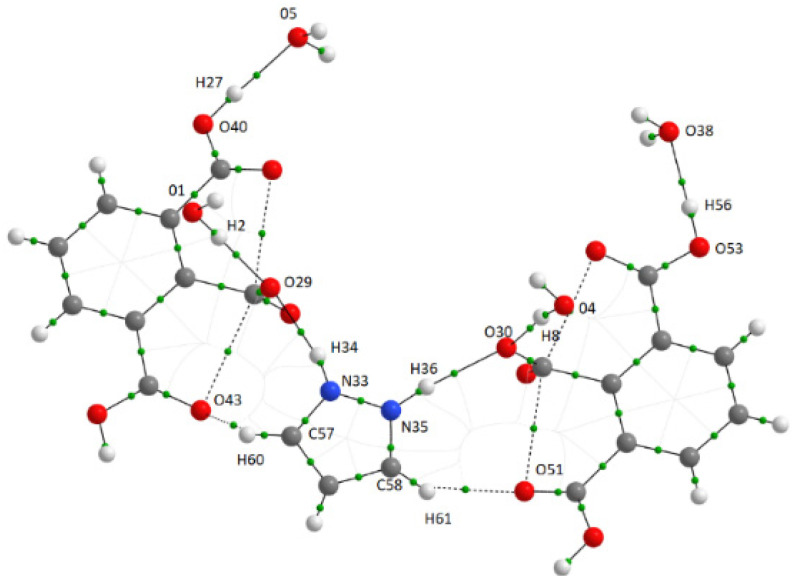
Molecular graphs (representation of bonding interactions according to QTAiM results) of the system analyzed in this study. Green circles correspond to the bond critical point.

For the 1*H*-pyrazol-2-ium 2,6-dicarboxybenzoate monohydrate, geometrical and topological parameters of intermolecular contacts, i.e., O—H^…^O, N—H^…^O, and C—H^…^O (Figure 6) are presented in Table 2. The QTAiM analysis of electron densities at the H^…^O bond critical points showed that *ρ*_BCP_ is in a range of 0.0296 to 0.0340 a.u., and Laplacian varies 0.1087 to 0.1268 a.u. A small *ρ*_BCP_ value indicates the depletion of the electronic charge in the internuclear region. The water molecule plays the role of proton acceptor center (oxygen atom) and proton donator. The most significant electron density at the proton-acceptor O—H^…^O bond critical point is observed for interaction between the hydroxyl proton of the carboxyl group and water oxygen (–COOH^…^OH_2_). It means that HBs’ interaction is more substantial for -COOH^…^water interaction than for –COO^−…^water interaction. The shortest H^…^O distances are observed for –COOH^…^water interaction. There are also –COO^−…^H—N and N-H⋅⋅⋅OH_2_ interactions. The most significant electron density at the bond critical point and the shortest bond length were observed for –COO^−…^H—N interaction. The shortest contact exists connecting the oxygen atom of the water molecule and the hydrogen atom of the carboxylic group. The kinetic and potential electron energy densities at BCP (*G*_C_, *V*_C_) are close to each other for –COO^−…^H—N interaction. The analysis of *V*_C_ and *G*_C_ shows that *G*_C_ is always greater than the modulus of *V*_C_. It means that *H*_C_, the total electron energy density at BCP, is greater than zero. Thus, one can expect that intermolecular interactions are of medium strength.

### 2.4. Electrical Properties Analysis

**Figure 7:**
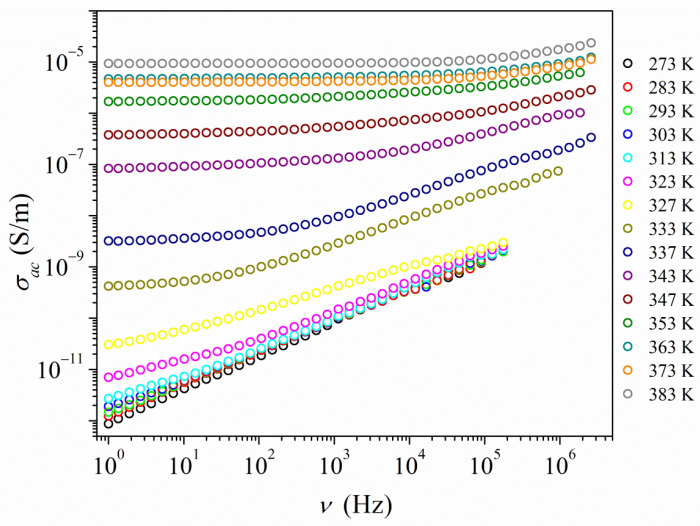
Dependence of the real part of electric conductivity as a function of frequency and temperature obtained for PyrHem×H_2_O.

For PyrHem×H_2_O, the temperature dependence (273–393 K) of the electrical conductivity has been investigated from 1 Hz to 10 MHz. The sample was a cylindrical pellet made of powdered crystallites. The frequency dependence of the real part of complex conductivity is presented in Figure 7.

In the temperature range from 273 to 313 K, the conductivity increases almost linearly with frequency. Measured ac conductivity values are very low and hardly change with temperature changes. Moreover, in a given range of frequency measurements, no plateau was observed to provide information about the value of the dc conductivity. As the temperature rises above 313 K, the frequency relationship of ac conductivity changes, which is typical of the ion conductor response. A plateau appears at the lowest frequencies, corresponding to the dc conductivity.

**Figure 8:**
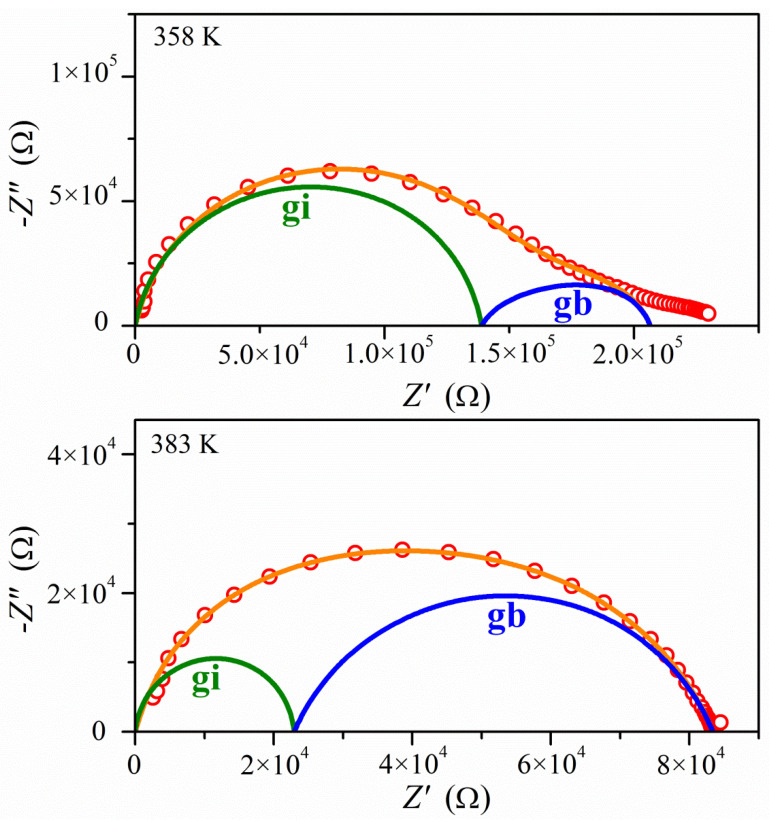
Complex impedance plots Z”(Z’) of the impedance for PyrHem×H_2_O for two temperatures 358 and 383 K. Red points are the experimental data, while the lines are obtained from fitting to Equation (3): olive lines denote the contributions from the interior of the crystallites (gi), blue lines are due to the crystallite boundaries (gb), and orange lines represent fitting for all samples.

Figure 8 shows the impedance dependence in the form of Nyquist plots (imaginary part Z” of total impedance Z* on the real part Z’) collected at temperatures *T* = 358 K and *T* = 383 K for PyrHem×H_2_O. The entire response contains a reply from the two parts of the sample: grain interior (crystallite) and grain boundaries. They can be easily distinguished from the total measured impedance using the proper fitting procedure. For fitting the experimental data, the Cole–Cole formula for double RC parallel equivalent circuits connected in series was used:

(3) $$Z^{*}\left(\omega \right)=\frac{R_{1}}{1+{\left(i\omega R_{1}C_{1}\right)}^{1-\alpha_{1}}}+\frac{\left(R_{2}-R_{1}\right)}{1+\left(i\omega (R_{2}-R_{1}\right)C_{2}{)}^{1-\alpha_{2}}}$$

where *R*_1_ denotes the resistance of the first contribution, *R*_2_ is the resistance of the sum of two contributions (the crystalline and grain boundaries), *C*_1_ and *C*_2_ mean electrical capacities of circuits 1 and 2, *α*_1_ and *α*_2_ are Cole–Cole parameters, and *ω =* 2π*ν* is the angular frequency of the measuring field. Such an approach is commonly used to analyze complex impedance responses in polycrystalline samples and ceramics [33,34]. The two contributions from bulk material (crystallite) and grain boundaries are visible at lower temperatures. As the temperature rises, the shape of the measured response gradually becomes more homogeneous (Figure S6).

**Figure 9:**
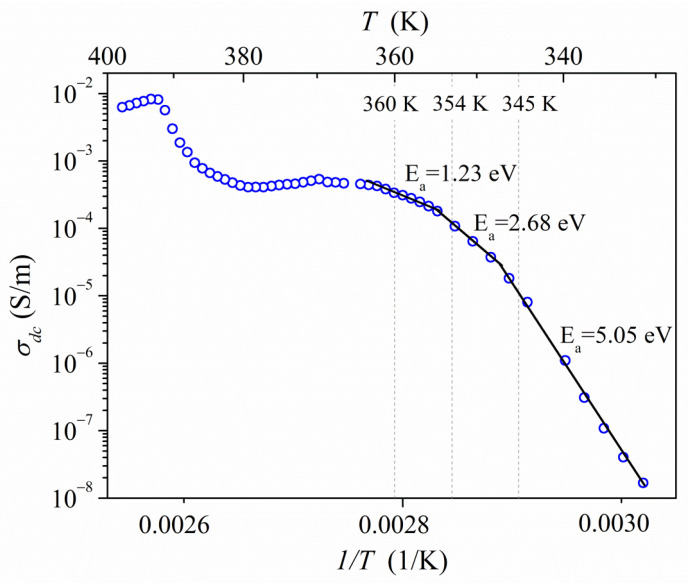
Arrhenius plot of dc conductivity of PyrHem×H_2_O.

The fitting procedure values of total impedance were used to calculate the dc conductivity of PyrHem×H_2_O. The dc conductivity versus the inverse temperature, in the form of the Arrhenius plot, is presented in Figure 9**.** The Arrhenius law has described it:

(4) $$\sigma_{dc}=\sigma_{0}e^{-\frac{E_{a}}{kT}}$$

where *σ_0_* means the pre-exponential factor, *E_a_* is the activation energy, and *k* is the Boltzmann constant. In the temperature range from 323 to 347 K, the conductivity is characterized by high activation energy *E*_σ1_ = 5.05 eV. Next, at the temperature range 347 K ≤ *T* ≤ 354 K and 354 K ≤ *T* ≤ 360 K, the activation energy decreases to the value of *E*_σ2_ = 2.68 and 1.23 eV, respectively. Above 360 K, dc conductivity is almost temperature-independent. The step decrease of conductivity above 388 K can be related to the degradation of the sample due to melting and the degradation of PyrHem×H_2_O. The maximum conductivity amounts to 8.2 × 10^−3^ S⋅m^−1^ at 388 K for the investigated proton-conducting material. It can be seen that the temperature at which the change in the slope of Arrhenius law is observed is in the temperature region where the dehydration process takes place. TGA measurements (Figure 4) show that the weight loss in the temperature range from 318 to 388 K is 5%. It is in good agreement with the endset temperature (354 K) of the endothermic peak with a minimum of 400 K. Water molecules that have emerged from the crystal lattice cause structural defects. An increase in proton conductivity is then possible.

### 2.5. Vibrational Analysis

**Figure 10:**
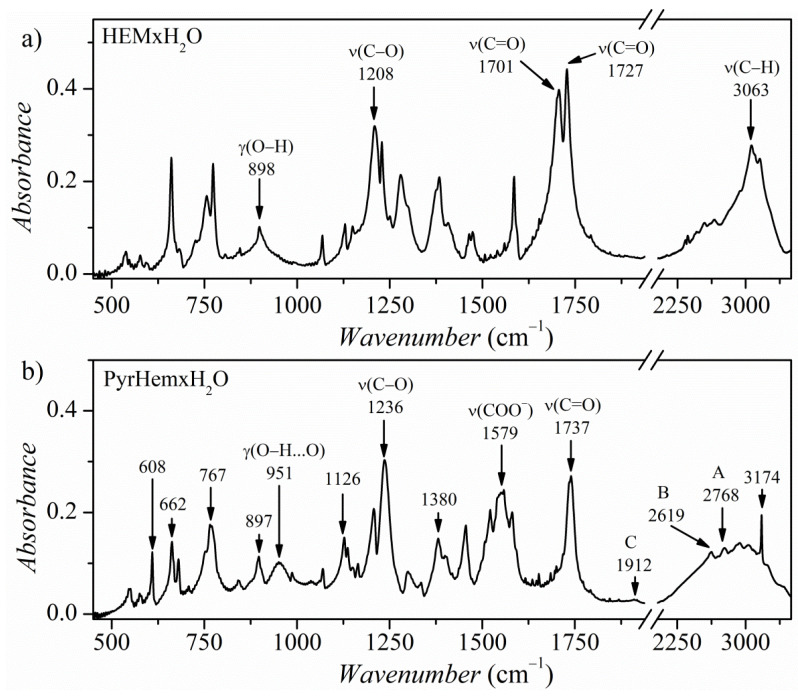
FT-IR absorption spectra of the HemxH_2_O (**a**) and PyrHemxH_2_O (**b**) recorded at 293 K.

Figure 10 shows the infrared spectra of hemimellitic acid hydrate Hem×H_2_O (a) and PyrHem×H_2_O (b) recorded at room temperature. The most prominent experimental vibrational bands with theoretically calculated frequencies are collected in Table S1. In the spectrum of Hem×H_2_O, there is a broad absorption in the range 3200–2200 cm^−1^ due to the stretching vibration of the hydrogen-bonded O-H group. Another band characteristic of the acid species is O-H out of plane vibration, which appears as a broad, medium intensity band with a maximum at 898 cm^−1^. The calculated γ_O-H_ frequency is 889 cm^−1^. It is worth mentioning that H-bond formation affects the stretching and deformational vibrational modes of the proton donating group as N-H and O-H bond and the modes of the proton accepting center, i.e., the C=O group and O-H of a water molecule. The carbonyl stretching band appears at 1727 and 1701 cm^−1^. According to theoretically calculated frequencies of vibrational modes, the lower frequency band (1701 cm^−1^) is due to the C=O stretching vibration of the C=O group, which interacts with a water molecule.

For PyrHem×H_2_O, in the range 3200–2800 cm^−1^, there are bands related to the N-H stretching modes of charged amine derivatives (ν_C=NH+_). In the range of 2800–1900 cm^−1^, multiple combinational bands are present. The bands denoted as A (2768 cm^−1^), B (2619 cm^−1^), and C (1912 cm^−1^) are associated with ν_OH_ stretching vibrations of a hydroxyl group bonded by hydrogen bonds [20,35,36,37]. The bands A, B, and C are due to Fermi resonance between the ν_OH_ stretching vibration, the overtone of the out-of-plane bending vibrations 2γ_OH_, and the combination of the out-of-plane γ_OH_ and in-plane δ_OH_ bending modes [38]. The formation of the amine salt results from the transfer phenomenon of a proton from the Lewis acid unit to the Lewis base. Bands at 1579 and 1380 cm^−1^ are due to the out-of-phase and in-phase COO^−^ stretching vibrations of the carboxylate COO^−^ group, respectively [14,15].

**Figure 11:**
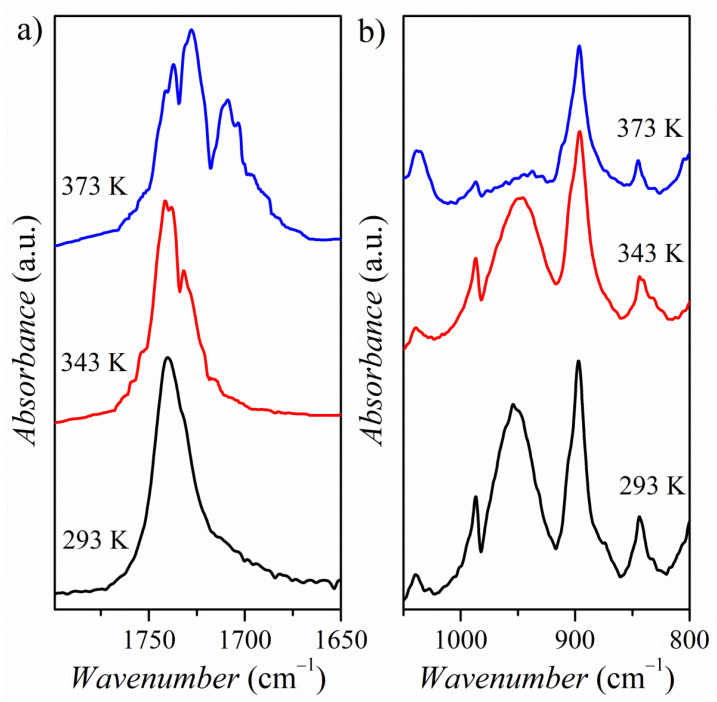
FT-IR absorption spectra of the PyrHem×H_2_O at 293, 343, and 373 K in spectral ranges: 1800–1650 cm^−1^ (**a**) and 1100–800 cm^−1^ (**b**).

The crystal structure of PyrHem×H_2_O revealed that both O—H^…^OH_2_ and C-O^−…+^H-N interactions exist (Figure 1). In the spectral range 500–900 cm^−1^, the HB interaction leads to the formation of bands related to out-of-plane mode γ_O‒H⸱⸱⸱O_ [39]. For PyrHem×H_2_O, the hydroxyl O—H moiety of -COOH carboxylic groups interact with water molecules, forming O—H^…^O hydrogen bonds (Figure 2). The band at 897 cm^−1^ was ascribed to out-of-plane O—H bending mode based on normal modes’ theoretical calculations (see Table S1). A moderately intense, broad IR band at 951 cm^−1^ is due to the O—H bending of the hydrogen-bonded hydroxyl group of the carboxylic moiety. Decreasing intensity of this γ_O‒H…O_ band with temperature in IR spectra of PyrHem×H_2_O was observed (Figure 11). At 303 K intensity ratio, I_770_/I_951_ is 4.03, at 343K it is 5.18, and at 363K it is 15.87, while at 373 K, the band at 951 cm^−1^ is almost invisible. It means that the hydrogen bond between the hydroxyl group of the carboxylic moiety and water molecule is broken with increasing temperature.

**Figure 12:**
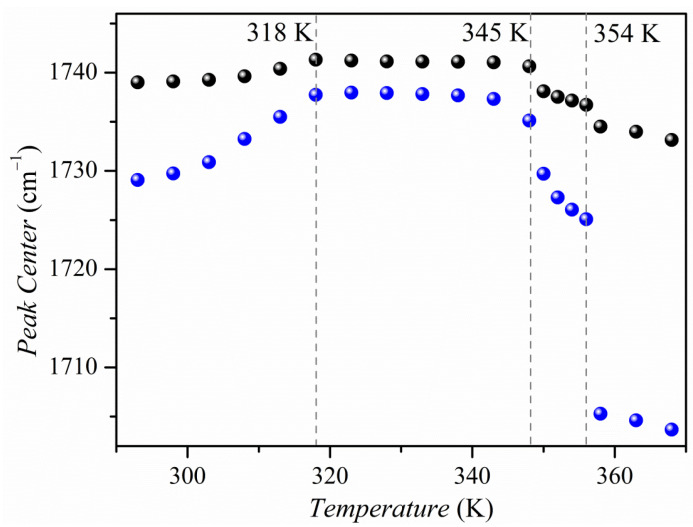
Temperature evolution of the position of the carbonyl bands 1737 (black spheres) and 1728 cm^−1^ (blue spheres).

Figure 11 shows the temperature evolution of the carbonyl band. The intense absorption band of the C=O group is present at 1737 cm^−1^ at 293 K. Under the influence of temperature, the bandwidth broadening and a fine structure of the carbonyl band are revealed. There are two doublets: at 1741, 1737 cm^−1^ and 1732, 1728 cm^−1^. The temperature dependence of the positions of the bands 1737 and 1728 cm^−1^ is presented in Figure 12. This variation in band position correlates very well with changes in activation energy in electrical conductivity measurements (Figure 9).

Moreover, the position of the band 1702 cm^−1^ at high temperatures may indicate that carbonyl groups interact with water molecules. The theoretical calculations for Hem×H_2_O showed that the band associated with the C=O stretching vibration of the C=O group, which interacts with a water molecule, should be observed around 1701 cm^−1^. The observed splits of the carbonyl band in the IR spectrum of PyrHem×H_2_O can be related to the phenomenon of involvement of the mobile water molecules in the electrical conductivity. Their diffusion process occurs within the dynamic network of hydrogen bonds, which may cause a non-equivalence in the carbonyl groups in the crystal lattice. 

**Figure 13:**
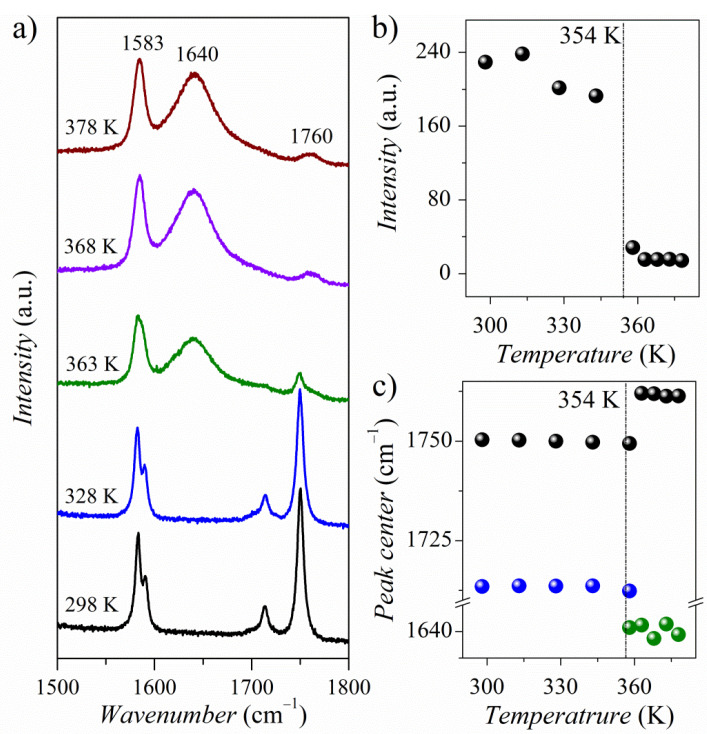
Temperature variation of Raman spectra of the PyrHem×H_2_O (**a**), the temperature dependence of the intensity of the 1760 cm^−1^ band (**b**), and the temperature dependence of the positions of the Raman bands (**c**).

Figure 13a presents temperature-dependent Raman spectra of PyrHem×H_2_O in a spectral range of 1800–1500 cm^−1^. The room temperature spectrum consist of a prominent band at 1760 cm^−1^ (ν_C=O_) and 1583 cm^−1^ (ν_C=C_). Above the 354 K, a new band at 1640 cm^−1^ arises (Figure 13a). According to theoretical calculations, it is due to the deformational vibration of the water molecule δ_O-H_. It may indicate that dynamic water molecules appear in the crystal lattice. This phenomenon influences the band’s position and intensity associated with C = O vibrations (Figure 13b,c).

## 3. Materials and Methods

### 3.1. Experimental Section

#### 3.1.1. Synthesis

Pyrazole (Pyr) (Sigma-Aldrich, purity 98%) and 1,2,3-benzenetricarboxylic acid hydrate (Hem×H_2_O) (Sigma-Aldrich, 98%) are dissolved separately in ethyl acetate (Merck KGaA, 99.8%). The mole ratio of pyrazole to acid amounts 1:1. The solutions were combined and mixed. The precipitate was isolated by filtration, washed out with cold ethyl acetate, and allowed to dry. Crystallization of the salt was carried out in ethyl acetate. The crystals 1*H*-pyrazol-2-ium 2,6-dicarboxybenzoate monohydrate (1:1:1) (PyrHem×H_2_O) formed were transparent plate, 5 mm in length and about 4 mm in thickness.

#### 3.1.2. Crystal Structure Determination

*Crystal data*. C_12_H_12_N_2_O_7_, Mr = 296.24, monoclinic, space group *C*2/*c*, *a* = 31.2067(7), *b* = 11.7802(3), *c* = 6.8118(2) Å, *β* = 91.381(2)°, *V* = 2503.43(11) Å^3^, *Z* = 8, *D*_calc_ = 1.572 g/cm^3^, *μ* = 1.138 mm^−1^, *T* = 293(2) K.

*Data collection*. A colorless pillar crystal (AcOEt) of 0.20×0.16×0.06 mm was used to record 13,154 (Cu *Ka*-radiation, *θ*_max_ = 76.68°) intensities on a Rigaku SuperNova Dual Atlas diffractometer [40] using mirror-monochromatized Cu *Kα* radiation from a high-flux microfocus source (λ = 1.54184 Å). Accurate unit cell parameters were determined by least-squares techniques from the *θ* values of 5946 reflections, *θ* range 2.77–76.06°. The data were corrected for Lorentz polarization and for absorption effects [40]. The 2613 total unique reflections (*R*_int_ = 0.033) were used for structure determination.

*Structure solution and refinement.* The structure was solved by direct methods (SHELXS-97) [41] and refined against *F*^2^ for all data (SHELXL) [42]. The positions of the H atoms bonded to N and O atoms were obtained from the difference Fourier maps and were refined freely. The remaining H atoms were placed geometrically in calculated positions. They were refined with a riding model, with C–H = 0.93 Å (C*_ar_*H) and *U*_iso_(H) =1.2*U*_eq_(C). Final refinement converged with *R* = 0.0530 (for 2392 data with *F*^2^ > 4*σ*(*F*^2^)), wR = 0.1569 (on *F*^2^ for all data), and *S* = 1.037 (on *F*^2^ for all data). The largest difference peak and hole was 0.463 and −0.314 eÅ^3^. The molecular illustrations were drawn using ORTEP-3 for Windows [43]. The software used to prepare material for publication was WINGX [41], OLEX [44], and PLATON [45]. The supplementary crystallographic data are deposited at the Cambridge Crystallographic Data Centre (CCDC), 12 Union ROAD, Cambridge CB2 1EZ (UK) (phone, (+44) 1223/336-408; fax, (+44) 1223/336-033; e-mail, deposit@ccdc.cam.ac.uk; World Wide Web, http://www.ccdc.cam.ac.uk, accessed on 18 April 2021 (deposition no. CCDC 2091027)).

#### 3.1.3. DSC/TGA Analysis

Thermogravimetric analyses (TGA) were performed on a Mettler Toledo Star TGA/DSC unit (Greifensee, Switzerland). Samples weighing 2–3 mg were placed in aluminum oxide crucibles and heated from 50 °C (323 K) to 900 °C (1173 K) at a 10 K min^–1^ under an argon flow rate of 40 mL·min^–1^. Differential scanning calorimetry (DSC) measurements were performed on a Mettler Toledo Star DSC system (Greifensee, Switzerland). A sample (2–3 mg) was placed in an aluminum crucible, sealed, and then heated from 25 °C (298 K) to 480 °C (753 K) at a heating rate of 10 K min^–1^ under an argon flow rate of 40 mL·min^–1^.

#### 3.1.4. FT-IR and Raman Spectroscopy

Bruker Equinox 55 spectrometer connected with FT-IR Hyperion 2000 microscope was used to obtain FT-IR spectra in the spectral range from 450 to 4000 cm^−1^. The spectral resolution equals 2 cm^−1^. The spectra were measured using the KBr pellets technique. The Raman spectrum was recorded on a Jobin-Yvon HORIBA LabRAM HR 800 spectrometer equipped with a CCD detector. Raman spectra were recorded from 50 to 3700 cm^−1^ with the excitation line λ_ext_ = 633 nm and the spectral resolution better than 2 cm^−1^. The laser power was kept below 1 mW to avoid thermal and photochemical degradation of the sample. A cryostat made by Linkam Corp. was used to investigate the vibrational spectra versus temperature from 278 to 378 K.

#### 3.1.5. Impedance Spectroscopy

The electric properties of the investigated materials were studied using the impedance spectroscopy method. Salt was powdered in the agate mortar. The received powder was pressed at room temperature under 30 MPa to form cylindrical pellets (~0.4 mm thick and ~5 mm in diameter). Next, the electrodes were placed at the proper surface using Hans Wolbring GmbH silver paste. The real and imaginary parts of the electric impedance of the prepared samples were measured in the frequency range from 1 Hz to 10 MHz using the computer-controlled Alpha A High-Frequency Analyzer (Novocontrol GmbH). The temperature of the sample was stabilized by the Quatro Cryosystem with an accuracy of 0.1 K.

### 3.2. Computational Methods

The intermolecular interactions were analyzed using the Hirshfeld surface and fingerprint plots analysis. The calculations were carried out using the CrystalExplorer 3.0 program [46]. Normal mode calculations were performed with the Gaussian09 sets of codes [47]. The initial geometry of the pyrazolium hemimellitate was taken from X-ray data, and it was further applied in the geometry optimization. DFT methods with functional WB97XD [48] combined with the 6-311++G(d,p) standard basis set were used. Thus, the calculations were performed at WB97XD/6-311++G(d,p) level of approximation, including the vibrational frequencies. The results of optimizations correspond to energy minima since no imaginary frequencies were found. The method, as mentioned above, overestimates the calculated harmonic frequencies. For this reason, the scaling factors have been proposed in the literature to correct for anharmonicity. The predicted vibrational wavenumbers were scaled down by a single factor of 0.957. Gaussian output wfn files were used as inputs for the QTAiM program to calculate the topological properties of the salt. The bond critical points were located (BCPs and RCPs), and their properties, such as electron densities at critical points (*ρ*_BCP_ and *ρ*_RCP_) and their Laplacians (∇^2^*ρ*_BCP_ and ∇^2^*ρ*_RCP_), were calculated. The additional characteristics of BCPs were analyzed, such as total electron energy density at BCP (*H*_C_) and its components, potential electron energy density (*V*_C_), and kinetic electron energy density (*G*_C_) [49].

## 4. Conclusions

In this paper, we examined the conductive, thermal, and spectroscopic properties of the new proton conductor, 1*H*-pyrazol-2-ium 2,6-dicarboxybenzoate monohydrate with a maximum conductivity of 8.2 × 10^−3^ Sm^−1^ at 388 K. The intermolecular interactions analysis shows that the dominant interactions in the investigated salts are hydrogen bonding. The hydrogen bond between the hydroxyl group of the carboxylic moiety and water molecule is broken with increasing temperature. Free water molecules appear in the crystal lattice. The process of proton diffusion takes place in a dynamic network of hydrogen bonds with the participation of water molecules.

## Figures and Tables

**Table 1:** Hydrogen-bond geometry (Å, °) for PyrHem×H_2_O.

D—H∙∙∙A	D—H	H∙∙∙A	D∙∙∙A	D—H∙∙∙A
N1b—H1b∙∙∙O8a	0.89(2)	1.85(2)	2.7137(17)	165(2)
N2b—H2b∙∙∙O9a ^i^	0.91(2)	1.87(2)	2.7581(17)	165(2)
O12a—H12a∙∙∙O1w ^ii^	0.90(2)	1.82(2)	2.7217(15)	175(3)
O15a—H15a∙∙∙O1w	0.90(3)	1.83(2)	2.7164(15)	172(3)
O1w—H1wA∙∙∙O9a ^iv^	0.92(3)	1.86(3)	2.7695(16)	171(3)
O1w—H1wB∙∙∙O8a ^iii^	0.92(3)	1.86(3)	2.7695(16)	171(3)
C3b—H3b∙∙∙O14a ^i^	0.93	2.46	3.057(2)	122
C3b—H3b∙∙∙O14a ^v^	0.93	2.55	3.072(2)	116
C5b—H5b∙∙⋅O11a	0.93	2.56	3.032(2)	112
C5b—H5b∙∙O11a ^vi^	0.93	2.35	3.071(2)	134

Symmetry codes: (i) 3/2−*x*, 1/2−*y*, 1−*z*; (ii) *x*, −1+*y*, *z*; (iii) *x*, 1−*y*, −1/2+*z*; (iv) *x*, 1−*y*, 1/2+*z*; (v) 3/2−*x*, −1/2+*y*, 3/2−*z*; (vi) *x*, −*y*, 1/2+*z.*

**Table 2:** Geometrical parameters (d_H_⸱⸱⸱o in Ångstrom and < D‒H⸱⸱⸱A in degrees) corresponding to the D‒H⸱⸱⸱A contacts obtained at the B3LYP/6-311++G(d,p); QTAiM parameters (in atomic unit) corresponding to the H⸱⸱⸱O bond critical point (BCPs), the electron density at BCP *ρ*_BCP_; Laplacian of electron density at BCP, ∇^2^*ρ*_BCP_; total electron energy density at BCP, *H*_C_ and the components of the *H*_C_: kinetic electron energy density, *G*_C_; potential electron energy density, *V*_C_. The atomic labeling is presented in Figure 6.

D‒H^…^A	$d_{H\cdots A}$	D-H^…^A	$d_{D-H}$	$\rho_{BCP}$	$\nabla^{2}\rho_{BCP}$	$G_{C}$	$V_{C}$	$H_{C}$
N35—H⸱⸱⸱O30	1.862	164.8	0.918	0.0305	0.1106	0.0262	−0.0250	0.0014
N33—H⸱⸱⸱O29	1.851	164.1	0.886	0.0315	0.1202	0.0283	−0.0266	0.0017
O1—H⸱⸱⸱O29	1.875	175.8	0.900	0.0296	0.1087	0.0258	−0.0243	0.0014
O4—H⸱⸱⸱O30	1.857	171.1	0.919	0.0307	0.1106	0.0265	−0.0253	0.0012
O40—H⸱⸱⸱O5	1.804	172.6	0.918	0.0340	0.1262	0.0309	−0.0302	0.0007
O53—H⸱⸱⸱O38	1.815	174.5	0.911	0.0333	0.1241	0.0302	−0.0293	0.0009
C58—H⸱⸱⸱O51	2.457	122.3	0.931	0.0099	0.0375	0.0077	−0.0061	0.0016
C57—H⸱⸱⸱O57	2.556	112.2	0.929	0.0089	0.0346	0.0071	−0.0056	0.0015

## Data Availability

The data presented in this study are available on request from the corresponding author.

## Supplementary Materials

The following are available online. Figure S1: The molecular packing in the crystal PyrHemxH_2_O, showing sheets (layers) parallel to the *bc* plane. The H atoms not involved in hydrogen bonds have been omitted for clarity, Figure S2: Non-classic C-H⋅⋅⋅O hydrogen bonds linking types into layers parallel to the *bc* plane. The symmetry codes are explained in Table 1, Figure S3: π ··· π interactions between pyrazolium rings. The molecules are shown in two orthogonal projections. Hydrogen atoms have been omitted for clarity, Figure S4: TGA/DTG curves (top panel) and DSC curves (bottom panel) of Hem×H_2_O, Figure S5: TGA/DTG curves (top panel) and DSC curves (bottom panel) of Pyr, Figure S6: Scaled complex impedance plots –Z”(Z’) plot for the measured pyrazolium hemimellitate, Table S1: Experimental and calculated (WB97XD/6-311++G(d,p)) IR stretching and bending frequencies of the O-H, N-H, and C=O groups.

## Sample Availability

Samples of the compound 1*H*-pyrazol-2-ium 2,6-dicarboxybenzoate monohydrate are available from the authors.
