# Supplementary material for: Spectroscopic and Structural Study of a New Conducting Pyrazolium Salt
Source: Molecules. 2021 Jul 31;26(15):4657. doi: 10.3390/molecules26154657 (PMC8347911; doi:10.3390/molecules26154657)
Supplement: Supplementary file 1 [file molecules-26-04657-s001.zip › molecules-1294125-supplementary.pdf]

Supplementary Materials

Spectroscopic and structural study of a new conducting pyrazolium salt

Sylwia Zięba [1], Agata Piotrowska [2], Adam Mizera [1], Paweł Ławniczak [1], Karolina H. Markiewicz [3], Andrzej Gzella [4], Alina T. Dubis [3] and Andrzej Łapiński [1,*]

[1] Institute of Molecular Physics Polish Academy of Sciences, Smoluchowskiego 17, 60-179 Poznań, Poland
[2] Poznań University of Technology, Piotrowo 3, 60-965 Poznań Poland
[3] Faculty of Chemistry University of Bialystok, Ciołkowskiego 1K, 15-245 Białystok, Poland
[4] Departament of Organic Chemistry Poznan University of Medical Sciences, Grunwaldzka 6, 60-780 Poznań, Poland

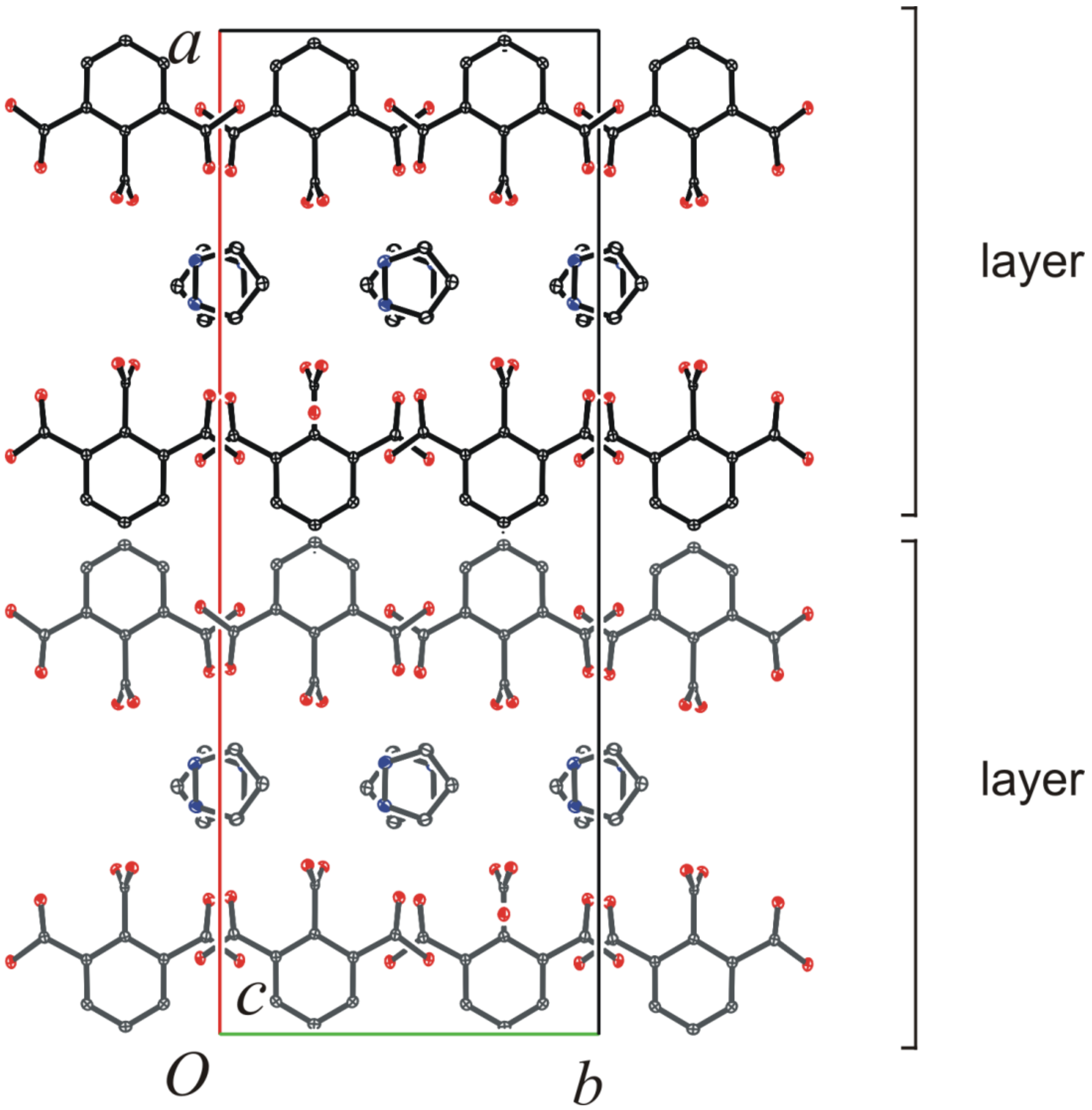

Figure S1. The molecular packing in the crystal PyrHemxH_2O, showing sheets (layers) parallel to the *bc* plane. The H atoms not involved in hydrogen bonds have been omitted for clarity.

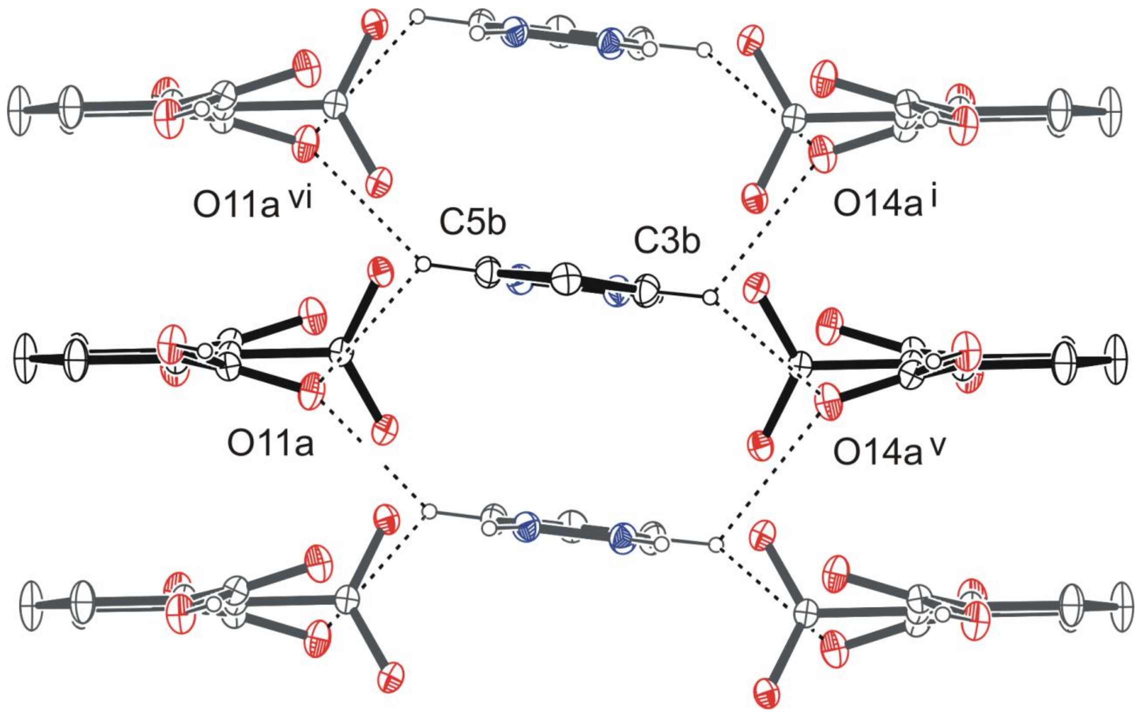

Figure S2. Non-classic C-H···O hydrogen bonds linking types into layers parallel to the *bc* plane. The symmetry codes are explained in Table 1

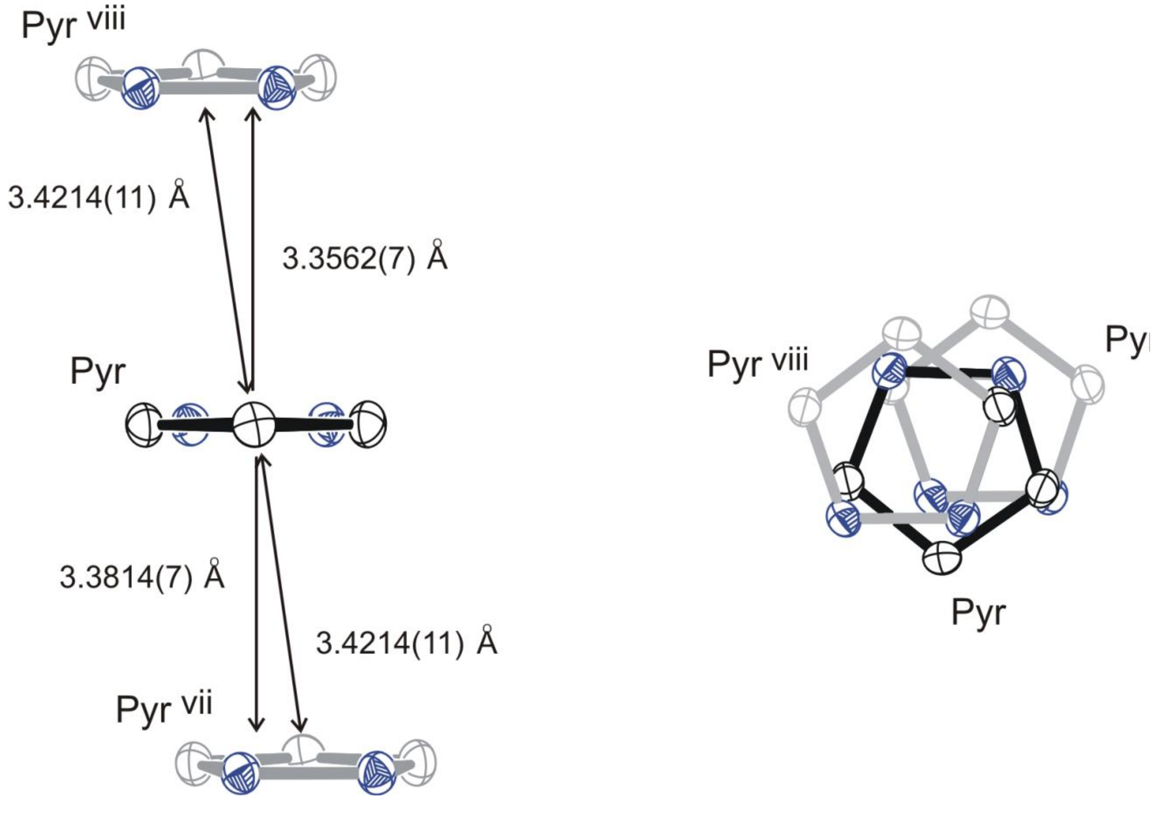

Figure S3. π ··· π interactions between pyrazolium rings. The molecules are shown in two orthogonal projections. Hydrogen atoms have been omitted for clarity.

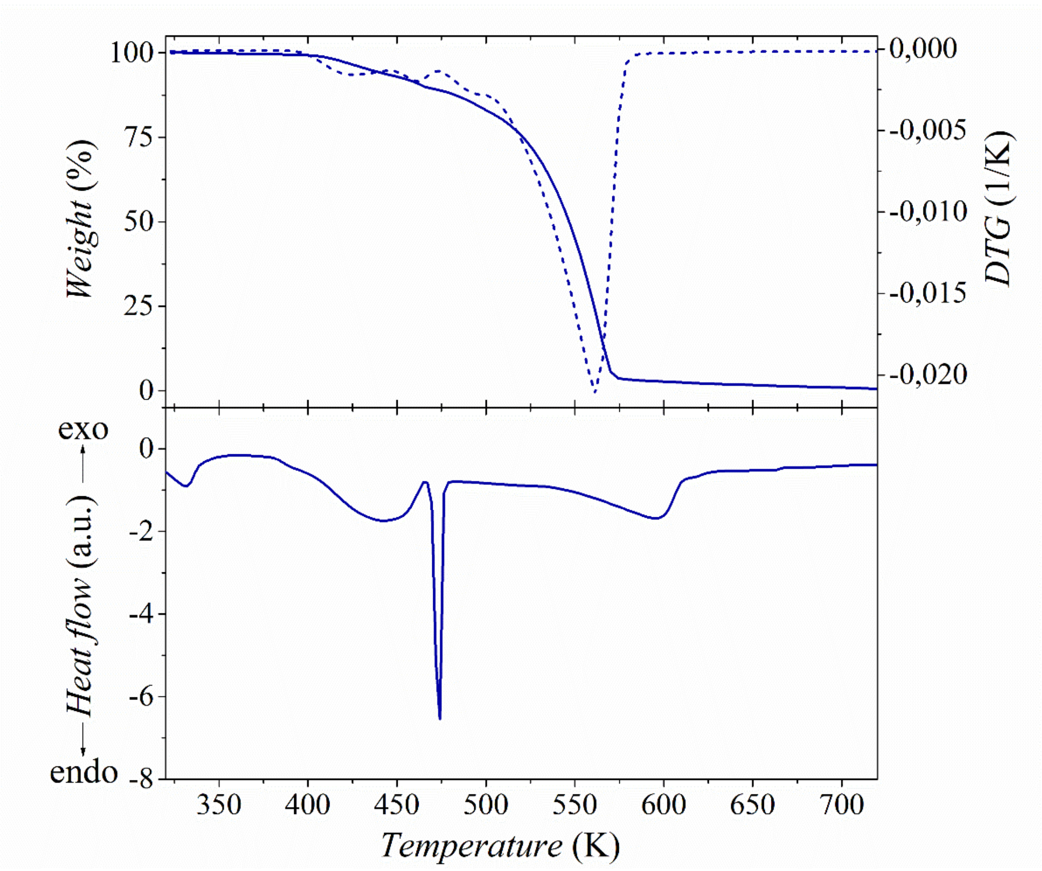

Figure S4. TGA/DTG curves (top panel) and DSC curves (bottom panel) of Hem×H_2O.

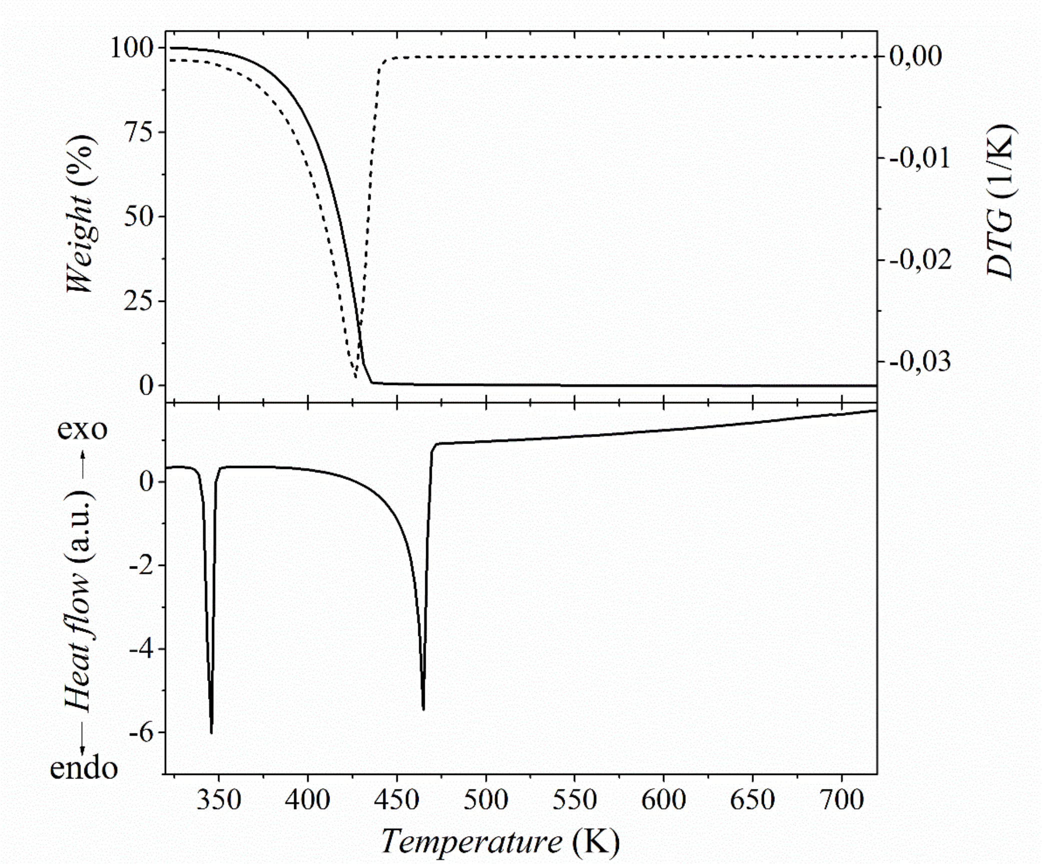

Figure S5. TGA/DTG curves (top panel) and DSC curves (bottom panel) of Pyr.

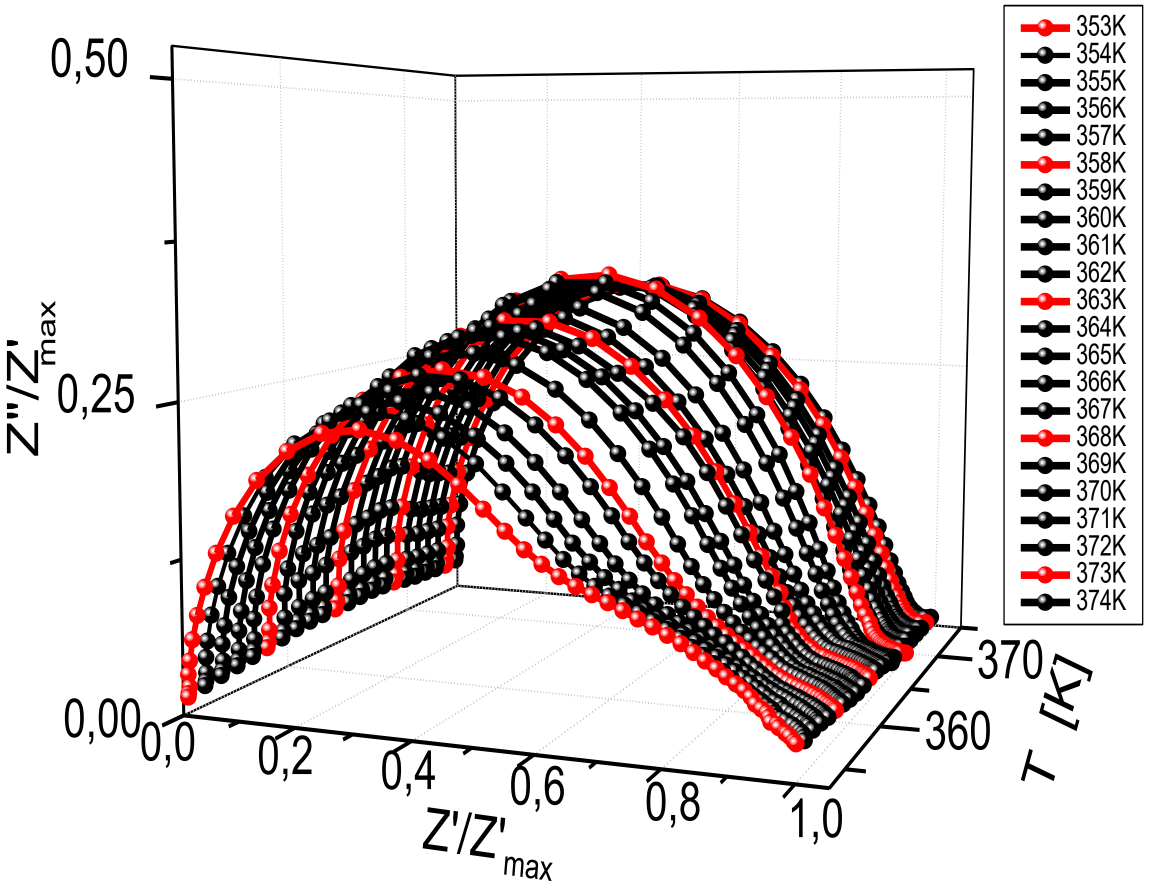

Figure S6. Scaled complex impedance plots –Z′′(Z′) plot for the measured pyrazolium hemimellitate.

Table S1. Experimental and calculated (WB97XD/6-311++G(d,p)) IR stretching and bending frequencies of the O-H, N-H, and C=O groups.

	Selected experimental and calculated IR frequencies [cm^{-1}]											
	Exp.	Calc.*	Exp.	Calc.*	Exp.	Calc.*	Exp.	Calc.*	Exp.	Calc.*	Exp.	Calc.*
	ν_{N-H}		ν_{O-H}		$\nu_{C=O\ or\ COO^-}$		δ_{O-H}		γ_{O-H}		γ_{N-H}	
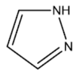	3425	3548										527
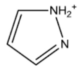		3501<br>3491										548
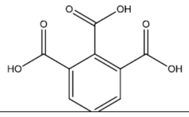				3716<br>3683<br>3673		1808<br>1779<br>1771						
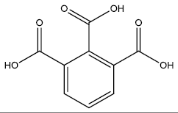 H2C			3400-<br>2500	3306<br>3583<br>3677<br>3684<br>3799	1727<br>1705<br>1701<br>1697<br>1685	1735 1771<br>1785		1552	898	889		
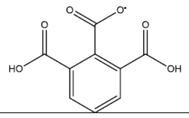				3695		1750<br>1747<br>1670						
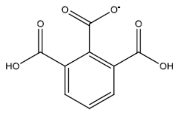 H2O				3395<br>3143		1747<br>1731<br>1668		1623	898	939		
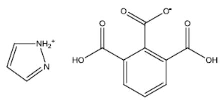		3327		3687<br>3686<br>2897		1780<br>1767<br>1723				964		

* scaling factor of 0.957 was used

Suplementary data – Theoretical Calculation of vibrational modes (wb97xd_6311++G_dp)

pyrazole PYR

Harmonic frequencies (cm**-1), IR intensities (KM/Mole), Raman scattering activities (A**4/AMU), depolarization ratios for plane and unpolarized incident light, reduced masses (AMU), force constants (mDyne/A), and normal coordinates:

	1	2	3
	A	A	A
Frequencies --	551.0021	639.8957	696.5277
Red. masses --	1.7096	2.3982	2.8376
Frc consts --	0.3058	0.5786	0.8111
IR Inten --	65.7180	0.0009	23.9088
Raman Activ --	0.3548	0.0017	0.2578
Depolar (P) --	0.7489	0.7491	0.7500
Depolar (U) --	0.8564	0.8566	0.8571

Atom	AN	X	Y	Z	X	Y	Z	X	Y	Z
1	6	0.00	0.00	0.08	0.00	0.00	0.20	0.00	0.00	0.08
2	1	0.00	0.00	0.22	0.00	0.00	0.53	0.00	0.00	0.16
3	6	0.00	0.00	-0.01	0.00	0.00	-0.25	0.00	0.00	0.04
4	1	0.00	0.00	-0.09	0.00	0.00	-0.28	0.00	0.00	0.26
5	6	0.00	0.00	0.00	0.00	0.00	0.14	0.00	0.00	-0.21
6	1	0.00	0.00	-0.02	0.00	0.00	0.47	0.00	0.00	-0.33
7	7	0.00	0.00	-0.21	0.00	0.00	-0.06	0.00	0.00	-0.14
8	7	0.00	0.00	0.08	0.00	0.00	-0.03	0.00	0.00	0.28
9	1	0.00	0.00	0.94	0.00	0.00	-0.54	0.00	0.00	-0.80

	4	5	6
	A	A	A
Frequencies --	769.7786	862.9843	906.9295
Red. masses --	1.2012	1.2435	1.3550
Frc consts --	0.4194	0.5457	0.6567
IR Inten --	104.4112	8.1462	2.3796
Raman Activ --	0.1196	0.2676	0.3782
Depolar (P) --	0.7492	0.7497	0.7498
Depolar (U) --	0.8566	0.8569	0.8570

Atom	AN	X	Y	Z	X	Y	Z	X	Y	Z
1	6	0.00	0.00	0.11	0.00	0.00	-0.10	0.00	0.00	0.06
2	1	0.00	0.00	-0.61	0.00	0.00	0.62	0.00	0.00	-0.38
3	6	0.00	0.00	0.06	0.00	0.00	0.05	0.00	0.00	-0.12
4	1	0.00	0.00	-0.73	0.00	0.00	-0.39	0.00	0.00	0.57
5	6	0.00	0.00	0.02	0.00	0.00	0.10	0.00	0.00	0.12
6	1	0.00	0.00	-0.22	0.00	0.00	-0.65	0.00	0.00	-0.71
7	7	0.00	0.00	-0.03	0.00	0.00	-0.01	0.00	0.00	-0.01
8	7	0.00	0.00	-0.01	0.00	0.00	0.00	0.00	0.00	-0.01
9	1	0.00	0.00	-0.13	0.00	0.00	-0.12	0.00	0.00	0.02

	7	8	9
	A	A	A
Frequencies --	930.8108	946.8630	1056.2637
Red. masses --	5.6542	3.6028	1.4588
Frc consts --	2.8863	1.9031	0.9589
IR Inten --	9.4544	3.5992	6.2431
Raman Activ --	3.3064	1.1856	1.5429
Depolar (P) --	0.7341	0.6833	0.5783
Depolar (U) --	0.8467	0.8119	0.7328

Atom	AN	X	Y	Z	X	Y	Z	X	Y	Z
1	6	0.22	0.12	0.00	0.13	0.23	0.00	-0.02	0.02	0.00
2	1	-0.04	0.39	0.00	0.40	-0.03	0.00	0.03	-0.02	0.00
3	6	0.17	-0.35	0.00	0.12	-0.04	0.00	0.06	0.08	0.00
4	1	0.34	-0.24	0.00	-0.44	-0.43	0.00	0.71	0.53	0.00

5	6	-0.32	-0.15	0.00	0.10	-0.06	0.00	-0.04	-0.11	0.00
6	1	-0.29	-0.25	0.00	-0.08	0.41	0.00	0.05	-0.36	0.00
7	7	0.02	0.10	0.00	-0.22	0.11	0.00	-0.07	0.06	0.00
8	7	-0.07	0.25	0.00	-0.06	-0.23	0.00	0.01	-0.08	0.00
9	1	-0.17	-0.30	0.00	-0.18	0.19	0.00	-0.01	0.20	0.00

	10	11	12
	A	A	A
Frequencies --	1071.6262	1159.9395	1205.3788
Red. masses --	1.5564	1.8191	2.9900
Frc consts --	1.0531	1.4421	2.5595
IR Inten --	44.6039	21.6112	2.6461
Raman Activ --	1.4128	7.5022	28.2985
Depolar (P) --	0.6358	0.0325	0.0797
Depolar (U) --	0.7774	0.0630	0.1476

Atom	AN	X	Y	Z	X	Y	Z	X	Y	Z
1	6	0.06	-0.04	0.00	-0.04	-0.15	0.00	0.10	0.01	0.00
2	1	0.58	-0.57	0.00	0.13	-0.35	0.00	0.52	-0.38	0.00
3	6	-0.05	0.03	0.00	0.01	-0.13	0.00	-0.10	0.10	0.00
4	1	-0.22	-0.08	0.00	0.27	0.02	0.00	-0.29	0.00	0.00
5	6	0.00	-0.03	0.00	-0.02	0.07	0.00	-0.20	-0.04	0.00
6	1	0.15	-0.44	0.00	-0.22	0.61	0.00	-0.23	-0.05	0.00
7	7	-0.11	0.00	0.00	-0.04	0.12	0.00	0.24	0.07	0.00
8	7	0.08	0.12	0.00	0.07	0.01	0.00	-0.10	-0.13	0.00
9	1	-0.13	-0.03	0.00	0.14	0.53	0.00	0.39	0.36	0.00

	13	14	15
	A	A	A
Frequencies --	1297.4867	1413.2508	1445.1333
Red. masses --	1.6875	2.7127	4.3175
Frc consts --	1.6738	3.1921	5.3125
IR Inten --	4.0680	3.0272	18.1955
Raman Activ --	11.5773	10.5409	28.0619
Depolar (P) --	0.0440	0.1020	0.5317
Depolar (U) --	0.0842	0.1851	0.6942

Atom	AN	X	Y	Z	X	Y	Z	X	Y	Z
1	6	0.00	0.10	0.00	-0.11	0.07	0.00	0.21	0.03	0.00
2	1	0.19	-0.08	0.00	0.43	-0.47	0.00	0.05	0.24	0.00
3	6	-0.09	0.03	0.00	-0.06	-0.11	0.00	-0.29	-0.20	0.00
4	1	0.18	0.24	0.00	0.39	0.17	0.00	0.40	0.28	0.00
5	6	-0.07	-0.06	0.00	0.21	-0.07	0.00	0.02	0.31	0.00
6	1	-0.34	0.62	0.00	0.15	0.14	0.00	0.33	-0.46	0.00
7	7	0.01	-0.07	0.00	0.11	0.08	0.00	-0.02	0.05	0.00
8	7	0.15	-0.02	0.00	-0.20	0.06	0.00	0.03	-0.16	0.00
9	1	-0.19	-0.52	0.00	-0.14	-0.45	0.00	-0.16	-0.25	0.00

	16	17	18
	A	A	A
Frequencies --	1513.4885	1592.1268	3261.7151
Red. masses --	2.7071	3.0669	1.0927
Frc consts --	3.6535	4.5804	6.8492
IR Inten --	7.1197	7.0465	2.7825
Raman Activ --	3.0395	2.4205	84.7030
Depolar (P) --	0.7487	0.0813	0.5784
Depolar (U) --	0.8563	0.1505	0.7329

Atom	AN	X	Y	Z	X	Y	Z	X	Y	Z
1	6	-0.10	0.23	0.00	0.23	-0.04	0.00	0.01	0.01	0.00
2	1	0.31	-0.16	0.00	-0.14	0.39	0.00	-0.10	-0.11	0.00
3	6	0.02	-0.11	0.00	-0.20	0.03	0.00	0.03	-0.03	0.00
4	1	0.24	0.01	0.00	0.08	0.27	0.00	-0.27	0.40	0.00
5	6	-0.02	0.09	0.00	0.14	-0.18	0.00	-0.07	-0.03	0.00
6	1	0.07	-0.12	0.00	-0.07	0.40	0.00	0.80	0.30	0.00
7	7	0.00	-0.24	0.00	-0.06	-0.08	0.00	0.00	0.00	0.00

```
    8   7     0.01   0.04   0.00    -0.09   0.12   0.00     0.00   0.00   0.00
    9   1     0.45   0.67   0.00     0.24   0.58   0.00     0.01   0.00   0.00
                 19                    20                    21
                  A                     A                     A
 Frequencies --   3276.3668             3294.5996             3708.9206
 Red. masses --      1.0964                1.1076                1.0821
 Frc consts  --      6.9346                7.0837                8.7699
 IR Inten    --      1.6933                0.1013               91.5755
 Raman Activ --     43.2397              132.4981               86.0580
 Depolar (P) --      0.3770                0.1323                0.2211
 Depolar (U) --      0.5476                0.2336                0.3621
  Atom  AN      X      Y      Z        X      Y      Z        X      Y      Z
    1   6     0.03   0.04   0.00    -0.06  -0.05   0.00     0.00   0.00   0.00
    2   1    -0.39  -0.39   0.00     0.58   0.57   0.00    -0.01  -0.01   0.00
    3   6     0.03  -0.05   0.00     0.03  -0.04   0.00     0.00   0.00   0.00
    4   1    -0.38   0.56   0.00    -0.31   0.45   0.00     0.00   0.00   0.00
    5   6     0.04   0.02   0.00     0.01   0.01   0.00     0.00   0.00   0.00
    6   1    -0.44  -0.17   0.00    -0.15  -0.06   0.00     0.00   0.00   0.00
    7   7     0.00   0.00   0.00     0.00   0.00   0.00     0.07  -0.03   0.00
    8   7     0.00   0.00   0.00     0.00   0.00   0.00     0.00   0.00   0.00
    9   1     0.01   0.00   0.00    -0.02   0.00   0.00    -0.90   0.42   0.00
```

2. PYRH+

```
Harmonic frequencies (cm**-1), IR intensities (KM/Mole), Raman scattering
 activities (A**4/AMU), depolarization ratios for plane and unpolarized
 incident light, reduced masses (AMU), force constants (mDyne/A),
 and normal coordinates:
                  1                      2                      3
                  A                      A                      A
 Frequencies --    453.2490               573.0164               621.2772
 Red. masses --      1.5759                 1.3541                 2.0727
 Frc consts  --      0.1907                 0.2620                 0.4714
 IR Inten    --      0.0024               231.0918                 8.2680
 Raman Activ --      0.2505                 0.0021                 0.0065
 Depolar (P) --      0.7493                 0.5953                 0.7424
 Depolar (U) --      0.8567                 0.7463                 0.8522
  Atom  AN      X      Y      Z        X      Y      Z        X      Y      Z
    1   6     0.00   0.00  -0.01     0.00   0.00   0.06     0.00   0.00   0.15
    2   1     0.00   0.00  -0.19     0.00   0.00   0.17     0.00   0.00   0.47
    3   6     0.00   0.00   0.00     0.00   0.00  -0.04     0.00   0.00  -0.23
    4   1     0.00   0.00   0.00     0.00   0.00  -0.11     0.00   0.00  -0.27
    5   6     0.00   0.00   0.01     0.00   0.00   0.06     0.00   0.00   0.15
    6   1     0.00   0.00   0.19     0.00   0.00   0.18     0.00   0.00   0.47
    7   7     0.00   0.00   0.15     0.00   0.00  -0.10     0.00   0.00  -0.02
    8   7     0.00   0.00  -0.15     0.00   0.00  -0.10     0.00   0.00  -0.02
    9   1     0.00   0.00  -0.67     0.00   0.00   0.67     0.00   0.00  -0.44
   10   1     0.00   0.00   0.66     0.00   0.00   0.67     0.00   0.00  -0.43
                  4                      5                      6
                  A                      A                      A
 Frequencies --    635.6544               800.3666               916.0982
 Red. masses --      2.2808                 1.1782                 1.2833
 Frc consts  --      0.5430                 0.4447                 0.6345
 IR Inten    --      0.0010               101.9502                 0.0006
 Raman Activ --      0.4315                 0.0130                 0.0818
 Depolar (P) --      0.7500                 0.7439                 0.7495
 Depolar (U) --      0.8571                 0.8531                 0.8568
  Atom  AN      X      Y      Z        X      Y      Z        X      Y      Z
```

1	6	0.00	0.00	0.15	0.00	0.00	-0.07	0.00	0.00	-0.11
2	1	0.00	0.00	0.21	0.00	0.00	0.42	0.00	0.00	0.69
3	6	0.00	0.00	0.00	0.00	0.00	-0.06	0.00	0.00	0.00
4	1	0.00	0.00	0.00	0.00	0.00	0.79	0.00	0.00	0.00
5	6	0.00	0.00	-0.15	0.00	0.00	-0.07	0.00	0.00	0.11
6	1	0.00	0.00	-0.22	0.00	0.00	0.42	0.00	0.00	-0.69
7	7	0.00	0.00	-0.18	0.00	0.00	0.02	0.00	0.00	0.01
8	7	0.00	0.00	0.18	0.00	0.00	0.02	0.00	0.00	-0.01
9	1	0.00	0.00	-0.63	0.00	0.00	0.07	0.00	0.00	-0.13
10	1	0.00	0.00	0.64	0.00	0.00	0.07	0.00	0.00	0.13

	7	8	9
	A	A	A
Frequencies --	917.0146	948.5643	949.3146
Red. masses --	5.7193	3.7439	1.3863
Frc consts --	2.8336	1.9848	0.7361
IR Inten --	2.8855	0.9460	0.0567
Raman Activ --	3.9288	0.6556	0.0882
Depolar (P) --	0.7148	0.7500	0.7493
Depolar (U) --	0.8337	0.8571	0.8567

Atom	AN	X	Y	Z	X	Y	Z	X	Y	Z
1	6	-0.01	-0.33	0.00	0.12	0.14	0.00	0.00	0.00	-0.09
2	1	-0.15	-0.31	0.01	-0.34	0.25	0.00	0.00	0.00	0.51
3	6	0.39	0.00	0.00	0.00	0.11	0.00	0.00	0.00	0.13
4	1	0.41	0.00	0.00	0.00	-0.47	0.00	0.00	0.00	-0.66
5	6	-0.01	0.33	0.00	-0.12	0.14	0.00	0.00	0.00	-0.09
6	1	-0.15	0.31	-0.01	0.34	0.25	-0.01	0.00	0.00	0.52
7	7	-0.16	-0.02	0.00	0.20	-0.17	0.00	0.00	0.00	0.01
8	7	-0.17	0.02	0.00	-0.20	-0.17	0.00	0.00	0.00	0.01
9	1	0.02	0.30	0.00	0.32	-0.01	0.00	0.00	0.00	-0.02
10	1	0.02	-0.30	0.00	-0.32	-0.01	0.00	0.00	0.00	-0.02

	10	11	12
	A	A	A
Frequencies --	1081.9582	1085.5749	1138.1878
Red. masses --	1.2973	1.4253	1.5495
Frc consts --	0.8948	0.9896	1.1826
IR Inten --	16.3727	47.1092	47.2144
Raman Activ --	2.0657	0.3760	0.1733
Depolar (P) --	0.7500	0.7486	0.7498
Depolar (U) --	0.8571	0.8562	0.8570

Atom	AN	X	Y	Z	X	Y	Z	X	Y	Z
1	6	0.00	-0.06	0.00	0.04	-0.04	0.00	-0.12	-0.04	0.00
2	1	0.09	-0.08	0.00	0.65	-0.17	0.00	-0.56	0.04	0.00
3	6	0.00	0.07	0.00	-0.09	0.00	0.00	0.00	-0.08	0.00
4	1	0.00	0.90	0.00	-0.10	0.01	0.00	0.00	-0.14	0.00
5	6	0.00	-0.06	0.00	0.04	0.04	0.00	0.12	-0.04	0.00
6	1	-0.11	-0.09	0.00	0.65	0.17	0.00	0.55	0.04	0.00
7	7	0.08	-0.02	0.00	-0.04	0.09	0.00	0.04	0.05	0.00
8	7	-0.07	-0.02	0.00	-0.04	-0.09	0.00	-0.04	0.05	0.00
9	1	0.19	0.16	0.00	0.00	0.17	0.00	0.21	0.33	0.00
10	1	-0.19	0.16	0.00	-0.01	-0.17	0.00	-0.22	0.33	0.00

	13	14	15
	A	A	A
Frequencies --	1212.8127	1268.5138	1351.7687
Red. masses --	5.0425	1.3069	1.3790
Frc consts --	4.3700	1.2390	1.4846
IR Inten --	6.4626	0.2188	8.8563
Raman Activ --	44.7853	9.6005	0.4346
Depolar (P) --	0.0562	0.3121	0.7500
Depolar (U) --	0.1064	0.4757	0.8571

Atom	AN	X	Y	Z	X	Y	Z	X	Y	Z

```
   1   6     0.06   0.25   0.00    -0.03  -0.04   0.00    -0.10   0.00   0.00
   2   1    -0.21   0.35   0.00    -0.27   0.01   0.00     0.47  -0.13   0.00
   3   6     0.26   0.00   0.00    -0.08   0.00   0.00     0.00   0.10   0.00
   4   1     0.31   0.00   0.00    -0.09   0.00   0.00     0.00  -0.46   0.00
   5   6     0.06  -0.25   0.00    -0.03   0.04   0.00     0.10   0.00   0.00
   6   1    -0.21  -0.36   0.00    -0.28  -0.01   0.00    -0.47  -0.13   0.00
   7   7    -0.15   0.22   0.00     0.06   0.06   0.00     0.01  -0.04   0.00
   8   7    -0.15  -0.22   0.00     0.06  -0.06   0.00    -0.01  -0.04   0.00
   9   1    -0.13   0.31   0.00     0.35   0.54   0.00     0.22   0.31   0.00
  10   1    -0.13  -0.31   0.00     0.35  -0.53   0.00    -0.22   0.31   0.00
                     16                     17                     18
                      A                      A                      A
 Frequencies --   1460.0448              1530.9064              1582.6399
 Red. masses --      4.1161                 2.6415                 3.8994
 Frc consts  --      5.1697                 3.6475                 5.7546
 IR Inten    --     71.6132                13.0320                 0.8406
 Raman Activ --     20.0942                 3.9957                 0.6933
 Depolar (P) --      0.7500                 0.1230                 0.1188
 Depolar (U) --      0.8571                 0.2190                 0.2124
 Atom  AN      X      Y      Z        X      Y      Z        X      Y      Z
   1   6     0.17  -0.19   0.00    -0.05  -0.10   0.00     0.30  -0.08   0.00
   2   1    -0.40  -0.11   0.00    -0.24  -0.08   0.00    -0.54   0.09   0.00
   3   6     0.00   0.35   0.00    -0.01   0.00   0.00    -0.21   0.00   0.00
   4   1     0.00  -0.44   0.00    -0.02   0.00   0.00    -0.26   0.00   0.00
   5   6    -0.17  -0.19   0.00    -0.05   0.10   0.00     0.30   0.08   0.00
   6   1     0.40  -0.11   0.00    -0.24   0.08   0.00    -0.54  -0.09   0.00
   7   7    -0.11   0.02   0.00     0.09   0.21   0.00    -0.11   0.00   0.00
   8   7     0.11   0.02   0.00     0.09  -0.21   0.00    -0.11   0.00   0.00
   9   1     0.05   0.30   0.00    -0.34  -0.50   0.00    -0.18  -0.08   0.00
  10   1    -0.05   0.30   0.00    -0.35   0.50   0.00    -0.18   0.08   0.00
                     19                     20                     21
                      A                      A                      A
 Frequencies --   1606.4810              3284.8434              3288.3133
 Red. masses --      2.2404                 1.0977                 1.1025
 Frc consts  --      3.4066                 6.9783                 7.0238
 IR Inten    --     33.4609                 0.1471                38.7585
 Raman Activ --      0.8277                90.0564                 8.2995
 Depolar (P) --      0.7500                 0.4286                 0.7459
 Depolar (U) --      0.8571                 0.6000                 0.8544
 Atom  AN      X      Y      Z        X      Y      Z        X      Y      Z
   1   6    -0.07  -0.09   0.00     0.01   0.06   0.00    -0.01  -0.07   0.00
   2   1     0.02  -0.14   0.00    -0.13  -0.61   0.00     0.15   0.71   0.00
   3   6     0.00   0.13   0.00    -0.03   0.00   0.00     0.00   0.01   0.00
   4   1     0.00  -0.27   0.00     0.39   0.00   0.00    -0.02  -0.01   0.00
   5   6     0.08  -0.09   0.00     0.01  -0.06   0.00     0.01  -0.06   0.00
   6   1    -0.02  -0.14   0.00    -0.14   0.66   0.00    -0.14   0.67   0.00
   7   7     0.15   0.08   0.00     0.00   0.00   0.00     0.00   0.00   0.00
   8   7    -0.15   0.08   0.00     0.00   0.00   0.00     0.00   0.00   0.00
   9   1    -0.24  -0.58   0.00    -0.01   0.01   0.00     0.00  -0.01   0.00
  10   1     0.24  -0.58   0.00    -0.01  -0.01   0.00     0.00  -0.01   0.00
                     22                     23                     24
                      A                      A                      A
 Frequencies --   3309.3330              3648.0377              3659.3064
 Red. masses --      1.1081                 1.0853                 1.0861
 Frc consts  --      7.1498                 8.5094                 8.5689
 IR Inten    --     26.9738               151.8665               347.0569
 Raman Activ --     97.4513                27.7952                98.7144
 Depolar (P) --      0.1005                 0.7494                 0.1151
 Depolar (U) --      0.1826                 0.8568                 0.2065
 Atom  AN      X      Y      Z        X      Y      Z        X      Y      Z
```

1	6	0.00	-0.03	0.00	0.00	0.00	0.00	0.00	0.00	0.00
2	1	0.05	0.27	0.00	0.00	0.00	0.00	0.00	0.01	0.00
3	6	-0.09	0.00	0.00	0.00	0.00	0.00	0.00	0.00	0.00
4	1	0.92	0.00	0.00	0.00	0.00	0.00	0.00	0.00	0.00
5	6	0.00	0.03	0.00	0.00	0.00	0.00	0.00	0.00	0.00
6	1	0.05	-0.27	0.00	0.00	-0.01	0.00	0.00	-0.01	0.00
7	7	0.00	0.00	0.00	-0.05	0.03	0.00	0.05	-0.03	0.00
8	7	0.00	0.00	0.00	0.05	0.03	0.00	0.05	0.03	0.00
9	1	0.01	-0.01	0.00	0.60	-0.36	0.00	-0.61	0.37	0.00
10	1	0.01	0.01	0.00	-0.61	-0.37	0.00	-0.59	-0.37	0.00

HEM

Harmonic frequencies (cm**-1), IR intensities (KM/Mole), Raman scattering activities (A**4/AMU), depolarization ratios for plane and unpolarized incident light, reduced masses (AMU), force constants (mDyne/A), and normal coordinates:

		1			2			3		
		A			A			A		
Frequencies --		36.0310			50.8398			69.3695		
Red. masses --		9.7882			7.4072			9.2713		
Frc consts --		0.0075			0.0113			0.0263		
IR Inten --		3.6050			1.6587			4.1927		
Raman Activ --		1.0499			0.8085			0.4063		
Depolar (P) --		0.7457			0.7370			0.7481		
Depolar (U) --		0.8543			0.8486			0.8559		
Atom	AN	X	Y	Z	X	Y	Z	X	Y	Z
1	6	0.00	0.00	-0.01	0.00	-0.01	-0.19	0.00	-0.01	-0.16
2	6	0.00	0.00	-0.04	0.00	0.00	-0.12	0.00	-0.02	-0.10
3	6	0.00	0.00	-0.02	-0.01	0.01	-0.05	0.01	-0.01	-0.03
4	6	0.00	0.00	0.00	-0.01	0.00	-0.06	0.01	-0.01	-0.03
5	6	0.00	0.00	0.01	-0.01	-0.01	-0.08	0.01	-0.01	-0.01
6	6	0.00	0.00	0.01	0.00	-0.01	-0.15	0.00	-0.01	-0.08
7	1	0.00	0.00	-0.01	0.01	-0.01	-0.25	0.00	-0.02	-0.23
8	1	0.00	0.00	-0.06	0.00	0.01	-0.12	0.00	-0.02	-0.11
9	1	0.01	-0.01	0.04	0.01	-0.02	-0.15	0.00	0.00	-0.09
10	6	0.01	0.01	-0.01	0.01	0.00	-0.03	-0.04	-0.02	-0.16
11	8	0.24	0.00	-0.12	0.00	0.03	-0.01	-0.01	-0.17	-0.27
12	8	-0.25	0.00	0.09	0.08	-0.02	-0.04	-0.17	0.14	-0.21
13	1	-0.25	0.00	0.08	0.11	-0.02	-0.03	-0.22	0.12	-0.34
14	6	0.00	0.01	0.03	0.00	0.01	0.05	0.02	0.00	0.11
15	8	-0.01	0.12	0.34	0.04	-0.05	-0.12	0.06	0.05	0.26
16	8	0.03	-0.12	-0.29	-0.05	0.09	0.37	0.00	-0.05	0.09
17	1	0.02	-0.09	-0.21	-0.03	0.08	0.42	0.01	-0.03	0.21
18	6	0.01	0.01	-0.01	0.00	-0.01	0.03	0.03	0.01	0.14
19	8	0.10	0.23	-0.22	0.14	0.13	-0.07	0.08	-0.15	0.33
20	8	-0.10	-0.21	0.22	-0.17	-0.16	0.29	0.00	0.22	0.08
21	1	-0.19	-0.39	0.32	-0.30	-0.26	0.30	-0.03	0.35	-0.05

		4			5			6		
		A			A			A		
Frequencies --		117.7184			136.8653			151.8997		
Red. masses --		10.4229			11.8515			6.2447		
Frc consts --		0.0851			0.1308			0.0849		
IR Inten --		2.6360			2.1048			2.3020		
Raman Activ --		1.5538			1.2329			3.3107		
Depolar (P) --		0.7188			0.7399			0.7436		
Depolar (U) --		0.8364			0.8505			0.8530		
Atom	AN	X	Y	Z	X	Y	Z	X	Y	Z
1	6	0.00	-0.04	0.06	-0.04	0.08	0.00	-0.01	-0.03	-0.21

2	6	0.01	-0.05	0.00	-0.05	0.07	-0.07	-0.01	-0.04	-0.23
3	6	-0.01	-0.05	-0.06	-0.04	0.07	-0.07	0.00	-0.02	0.04
4	6	0.00	-0.07	-0.04	-0.03	0.10	-0.04	0.01	0.00	0.25
5	6	0.00	-0.05	0.03	-0.04	0.10	0.03	0.01	0.01	0.26
6	6	0.00	-0.05	0.07	-0.02	0.10	0.06	-0.01	0.00	0.08
7	1	0.01	-0.04	0.10	-0.05	0.08	0.01	-0.03	-0.04	-0.44
8	1	0.01	-0.05	0.00	-0.05	0.07	-0.11	-0.02	-0.05	-0.47
9	1	0.00	-0.05	0.11	-0.03	0.11	0.13	-0.01	0.00	0.08
10	6	0.04	-0.06	-0.01	0.15	0.12	-0.06	0.01	-0.01	0.10
11	8	0.40	0.03	-0.12	0.40	0.21	-0.13	0.04	-0.19	-0.01
12	8	-0.34	-0.17	0.21	0.10	0.05	0.00	-0.03	0.18	0.01
13	1	-0.33	-0.16	0.29	0.34	0.07	-0.03	-0.03	0.16	-0.17
14	6	-0.07	0.03	-0.09	0.01	0.00	-0.03	-0.01	-0.02	-0.01
15	8	-0.17	-0.03	-0.27	0.14	-0.01	-0.02	-0.04	-0.04	-0.08
16	8	-0.02	0.21	0.12	-0.06	-0.09	0.04	0.00	0.02	0.04
17	1	-0.07	0.27	0.08	-0.01	-0.15	0.08	-0.01	0.02	-0.01
18	6	0.04	0.03	0.04	-0.15	-0.12	0.08	0.00	0.03	0.06
19	8	0.10	0.00	0.09	-0.40	-0.28	0.17	-0.14	0.13	-0.09
20	8	0.04	0.20	-0.04	-0.04	-0.28	0.02	0.16	-0.05	-0.07
21	1	0.01	0.17	-0.04	0.11	-0.10	-0.04	0.29	-0.12	0.06

	7	8	9
	A	A	A
Frequencies --	165.1556	171.8812	267.5933
Red. masses --	5.9677	6.0388	5.8185
Frc consts --	0.0959	0.1051	0.2455
IR Inten --	3.6620	2.2733	5.1453
Raman Activ --	1.3650	0.7248	0.6681
Depolar (P) --	0.7376	0.6384	0.7493
Depolar (U) --	0.8490	0.7793	0.8567

Atom	AN	X	Y	Z	X	Y	Z	X	Y	Z
1	6	0.02	0.03	0.07	-0.01	-0.04	0.06	-0.18	0.00	0.00
2	6	0.01	0.02	-0.17	-0.01	-0.05	-0.20	-0.08	-0.15	0.02
3	6	0.03	0.02	-0.20	-0.03	-0.05	-0.20	0.09	-0.15	-0.04
4	6	0.03	0.03	-0.14	-0.03	-0.07	-0.11	0.16	0.01	-0.11
5	6	0.04	0.03	0.04	-0.03	-0.05	0.08	0.09	0.16	-0.08
6	6	0.03	0.04	0.20	-0.02	-0.04	0.24	-0.08	0.16	-0.04
7	1	0.03	0.04	0.16	0.00	-0.03	0.14	-0.34	0.00	0.04
8	1	0.01	0.01	-0.25	-0.01	-0.05	-0.31	-0.13	-0.27	0.08
9	1	0.02	0.06	0.40	-0.01	-0.03	0.45	-0.16	0.29	-0.02
10	6	-0.10	0.03	-0.03	0.05	-0.06	-0.07	0.09	0.02	-0.05
11	8	-0.18	0.09	0.04	0.02	0.02	0.00	-0.09	0.01	0.03
12	8	-0.23	-0.05	0.06	0.22	-0.17	-0.08	-0.05	0.02	0.00
13	1	-0.44	-0.05	0.22	0.37	-0.15	-0.04	-0.36	0.01	0.12
14	6	0.09	-0.05	-0.06	-0.07	0.06	0.00	0.05	-0.09	0.01
15	8	0.24	-0.05	-0.03	-0.12	0.15	0.21	-0.18	-0.06	0.05
16	8	-0.02	-0.14	0.11	-0.05	0.12	0.05	0.19	0.13	0.01
17	1	0.06	-0.20	0.25	-0.09	0.23	0.26	0.07	0.31	0.06
18	6	0.03	0.02	0.02	0.00	0.01	0.02	0.06	0.08	0.00
19	8	-0.02	0.04	-0.02	-0.01	0.05	-0.03	-0.08	-0.01	0.05
20	8	0.08	-0.02	-0.02	0.04	0.05	-0.05	0.10	-0.14	0.06
21	1	0.12	-0.05	0.04	0.06	-0.04	0.03	0.21	0.05	-0.03

	10	11	12
	A	A	A
Frequencies --	291.5103	329.7312	367.3092
Red. masses --	10.7013	5.8229	6.7701
Frc consts --	0.5358	0.3730	0.5382
IR Inten --	0.9453	8.4404	5.7670
Raman Activ --	2.4055	0.8410	2.3919
Depolar (P) --	0.5569	0.0892	0.1566
Depolar (U) --	0.7154	0.1638	0.2708

Atom	AN	X	Y	Z	X	Y	Z	X	Y	Z
1	6	0.00	-0.11	0.04	-0.03	-0.01	0.19	0.12	-0.09	-0.12
2	6	-0.07	-0.04	-0.03	-0.02	-0.06	-0.17	0.10	-0.06	-0.07
3	6	-0.13	-0.03	-0.05	0.03	-0.04	-0.13	0.03	-0.06	0.17
4	6	-0.03	0.10	-0.01	0.04	-0.01	0.08	-0.01	0.00	-0.04
5	6	0.10	-0.06	-0.03	-0.01	0.00	-0.17	-0.01	-0.06	0.05
6	6	0.04	-0.06	0.02	-0.02	0.01	0.08	0.12	-0.08	0.18
7	1	0.01	-0.11	0.11	-0.05	0.00	0.43	0.13	-0.11	-0.30
8	1	-0.03	0.04	-0.06	-0.03	-0.09	-0.33	0.13	-0.01	-0.20
9	1	0.00	0.01	0.06	-0.03	0.04	0.18	0.15	-0.12	0.32
10	6	-0.02	0.21	0.02	0.06	0.01	0.16	-0.02	0.10	-0.09
11	8	0.02	0.28	0.04	0.05	-0.14	0.08	-0.02	0.27	0.00
12	8	0.00	0.29	-0.02	0.01	0.25	0.07	-0.03	0.06	-0.06
13	1	0.00	0.28	-0.15	-0.07	0.21	-0.17	-0.08	0.06	-0.03
14	6	-0.19	-0.09	-0.01	0.04	-0.01	-0.06	0.05	-0.13	0.13
15	8	-0.25	-0.07	0.03	0.04	0.01	0.00	-0.12	-0.16	0.02
16	8	-0.25	-0.13	0.03	0.06	0.05	0.02	0.22	0.02	-0.01
17	1	-0.20	-0.17	0.12	0.03	0.12	0.11	0.10	0.12	-0.25
18	6	0.19	-0.12	-0.02	-0.05	-0.04	-0.15	-0.10	-0.04	-0.01
19	8	0.32	-0.11	0.01	0.07	-0.12	-0.04	-0.07	0.00	-0.05
20	8	0.22	-0.11	-0.04	-0.23	0.05	-0.02	-0.19	0.14	-0.02
21	1	0.26	-0.13	0.00	-0.40	0.09	-0.15	-0.36	-0.11	0.08

	13	14	15
	A	A	A
Frequencies --	396.6048	421.5011	498.4035
Red. masses --	6.5824	5.0677	1.1473
Frc consts --	0.6100	0.5305	0.1679
IR Inten --	4.9527	8.4168	84.9252
Raman Activ --	2.7665	3.3556	0.6255
Depolar (P) --	0.7473	0.2139	0.3633
Depolar (U) --	0.8554	0.3524	0.5330

Atom	AN	X	Y	Z	X	Y	Z	X	Y	Z
1	6	-0.23	0.00	-0.06	0.04	0.14	-0.05	0.02	-0.01	0.02
2	6	-0.23	0.05	-0.06	0.06	0.08	-0.12	0.01	0.00	-0.01
3	6	-0.03	0.08	0.17	0.01	0.07	0.22	0.00	0.00	0.00
4	6	-0.09	0.01	0.07	0.05	0.05	0.00	-0.01	-0.01	0.02
5	6	-0.05	-0.07	0.01	0.05	0.13	-0.13	0.00	-0.02	0.02
6	6	-0.23	-0.04	0.13	0.06	0.17	0.17	0.01	-0.03	0.00
7	1	-0.15	0.00	-0.18	0.00	0.13	-0.14	0.03	-0.01	0.01
8	1	-0.27	-0.04	-0.22	0.05	0.07	-0.37	0.02	0.01	-0.04
9	1	-0.27	0.04	0.23	0.06	0.19	0.36	0.00	-0.02	-0.07
10	6	-0.12	0.01	-0.02	0.05	-0.04	-0.03	-0.02	0.00	0.00
11	8	0.01	0.07	-0.04	-0.03	-0.12	-0.04	0.00	0.02	0.00
12	8	-0.01	-0.07	-0.02	0.01	-0.13	0.03	-0.01	0.00	-0.01
13	1	0.30	-0.04	-0.06	-0.08	-0.12	0.21	0.09	0.00	-0.05
14	6	0.10	0.07	0.08	-0.07	0.02	0.12	0.00	-0.01	0.00
15	8	0.17	0.03	-0.03	-0.13	-0.04	-0.04	0.00	-0.01	0.00
16	8	0.18	0.12	-0.03	-0.11	-0.09	0.00	0.01	0.00	0.00
17	1	0.11	0.14	-0.33	-0.03	-0.30	-0.32	0.00	0.02	0.01
18	6	0.06	-0.09	-0.05	0.04	0.04	-0.11	0.00	0.02	0.00
19	8	0.22	-0.10	0.00	0.07	-0.07	0.02	0.00	0.03	-0.02
20	8	0.05	-0.08	-0.05	-0.04	-0.05	0.01	-0.02	-0.05	0.04
21	1	0.07	-0.05	-0.06	-0.04	0.16	-0.17	0.17	0.79	-0.56

	16	17	18
	A	A	A
Frequencies --	521.2878	565.7509	579.1314
Red. masses --	2.7003	2.0629	1.6520
Frc consts --	0.4323	0.3890	0.3264
IR Inten --	15.3017	50.8517	54.4733
Raman Activ --	0.8765	3.4419	1.3656

```
Depolar (P) --      0.2826                 0.3475                 0.6327
Depolar (U) --      0.4406                 0.5157                 0.7750
 Atom  AN      X      Y      Z        X      Y      Z        X      Y      Z
   1   6     0.04   0.08   0.10     0.00   0.06  -0.06    -0.01  -0.03  -0.09
   2   6     0.01   0.10  -0.09    -0.01   0.06   0.07     0.03  -0.06   0.08
   3   6    -0.03   0.05  -0.06     0.00   0.03  -0.04     0.03  -0.04   0.02
   4   6    -0.07   0.04   0.02     0.00   0.05   0.06     0.04  -0.02  -0.06
   5   6    -0.03   0.02  -0.06     0.01   0.04   0.12     0.03   0.02  -0.04
   6   6     0.02   0.01  -0.09     0.01   0.06   0.04     0.01   0.03   0.08
   7   1     0.09   0.09   0.29    -0.01   0.06  -0.21    -0.07  -0.04  -0.18
   8   1     0.03   0.14  -0.12    -0.01   0.05   0.11     0.01  -0.09   0.16
   9   1     0.05  -0.04  -0.09     0.00   0.06  -0.06     0.00   0.06   0.19
  10   6    -0.13   0.00   0.04     0.00   0.02  -0.01    -0.03  -0.01   0.03
  11   8     0.00  -0.03  -0.04    -0.01  -0.02  -0.03    -0.01   0.00   0.03
  12   8    -0.03  -0.04   0.03     0.01  -0.04   0.02    -0.07   0.03   0.03
  13   1     0.78   0.00  -0.21     0.00  -0.03   0.13     0.80   0.05  -0.40
  14   6     0.02  -0.10   0.03     0.02  -0.07  -0.09     0.00   0.03  -0.02
  15   8    -0.09  -0.11   0.03    -0.04  -0.03   0.02     0.02   0.03  -0.01
  16   8     0.12  -0.02   0.02     0.04  -0.03  -0.03    -0.04   0.00  -0.01
  17   1     0.03   0.10  -0.03    -0.04   0.32   0.84    -0.01   0.00   0.15
  18   6     0.00   0.03   0.03    -0.01  -0.06  -0.03     0.01   0.03  -0.03
  19   8    -0.01   0.03   0.03     0.07  -0.05  -0.02    -0.01  -0.01   0.00
  20   8     0.06  -0.01   0.00    -0.06   0.00  -0.03    -0.01   0.00   0.01
  21   1     0.10  -0.15   0.14    -0.11   0.02  -0.08    -0.04  -0.01   0.01
                    19                     20                     21
                     A                      A                      A
Frequencies --    593.4339               620.1620               650.2438
Red. masses --      4.0425                 1.9521                 4.5722
Frc consts  --      0.8388                 0.4423                 1.1390
IR Inten    --      9.6504                57.6246                52.9399
Raman Activ --      1.4982                 1.1518                 1.6954
Depolar (P) --      0.1365                 0.7469                 0.6599
Depolar (U) --      0.2402                 0.8551                 0.7951
 Atom  AN      X      Y      Z        X      Y      Z        X      Y      Z
   1   6    -0.02  -0.06   0.16     0.02  -0.02   0.03     0.03  -0.19  -0.02
   2   6     0.02  -0.14  -0.11     0.01   0.00  -0.05    -0.05  -0.06   0.02
   3   6     0.06  -0.07   0.10    -0.01   0.01   0.15    -0.16  -0.03   0.01
   4   6     0.09   0.02   0.25    -0.03  -0.04   0.00    -0.04   0.14  -0.02
   5   6     0.08   0.05   0.09    -0.03  -0.03  -0.15     0.04   0.04  -0.07
   6   6     0.04   0.06  -0.12    -0.01  -0.05   0.00     0.13   0.01   0.02
   7   1    -0.15  -0.07   0.15     0.05  -0.01   0.08    -0.03  -0.19   0.01
   8   1    -0.01  -0.20  -0.45     0.01   0.00  -0.20     0.04   0.10   0.05
   9   1    -0.01   0.11  -0.45     0.01  -0.07   0.12     0.08   0.11   0.12
  10   6    -0.04   0.04  -0.09    -0.01  -0.03   0.01     0.00   0.16   0.00
  11   8    -0.05   0.11  -0.06     0.02   0.03   0.03    -0.06  -0.10  -0.13
  12   8    -0.05  -0.11  -0.04     0.00   0.01  -0.03     0.06  -0.02   0.13
  13   1     0.39  -0.06   0.02    -0.20  -0.01  -0.05     0.14   0.04   0.52
  14   6    -0.01   0.04  -0.05    -0.01   0.02   0.02    -0.13  -0.07   0.02
  15   8     0.04   0.07  -0.01     0.03   0.00  -0.03     0.17  -0.09   0.04
  16   8    -0.07   0.01   0.00    -0.01  -0.01  -0.08    -0.03   0.14  -0.06
  17   1    -0.01  -0.05   0.12    -0.08   0.33   0.82    -0.29   0.49  -0.13
  18   6     0.04   0.00  -0.05     0.01   0.07   0.00     0.01   0.00   0.01
  19   8    -0.02  -0.04  -0.02    -0.04   0.04   0.03    -0.03   0.01  -0.01
  20   8    -0.04   0.03   0.02     0.06  -0.01   0.03     0.01   0.02   0.02
  21   1    -0.20  -0.14   0.08     0.12  -0.03   0.08     0.01   0.06  -0.02
                    22                     23                     24
                     A                      A                      A
Frequencies --    663.5443               725.8286               737.0709
Red. masses --      6.0002                 4.9317                 6.0663
Frc consts  --      1.5565                 1.5308                 1.9417
```

IR Inten -- 50.1226 61.1692 8.6291
Raman Activ -- 1.4252 17.9031 3.2206
Depolar (P) -- 0.1449 0.0452 0.0257
Depolar (U) -- 0.2531 0.0865 0.0501

Atom	AN	X	Y	Z	X	Y	Z	X	Y	Z
1	6	0.06	0.12	0.00	0.00	0.09	-0.02	-0.03	0.07	0.11
2	6	0.17	-0.12	0.01	-0.03	0.10	0.03	-0.05	0.05	-0.11
3	6	-0.07	-0.12	-0.01	-0.06	0.02	-0.06	-0.01	0.04	0.09
4	6	-0.08	-0.09	0.05	0.00	-0.03	0.06	0.06	0.01	-0.25
5	6	-0.18	0.11	0.05	0.09	-0.01	0.03	0.04	-0.01	0.17
6	6	0.05	0.16	-0.01	0.00	0.02	0.03	-0.02	0.02	-0.11
7	1	-0.18	0.12	-0.04	0.07	0.09	-0.13	0.02	0.08	0.16
8	1	0.18	-0.11	-0.02	-0.01	0.14	0.02	-0.07	0.02	-0.18
9	1	0.14	0.01	-0.09	-0.02	0.04	-0.09	-0.04	0.05	-0.26
10	6	0.02	-0.10	-0.01	-0.03	-0.14	0.04	0.28	-0.03	-0.09
11	8	0.03	0.06	0.09	0.06	0.04	0.11	-0.05	-0.04	0.09
12	8	-0.05	0.00	-0.09	-0.04	-0.03	-0.13	-0.09	0.03	0.04
13	1	-0.17	-0.04	-0.30	-0.23	-0.08	-0.40	0.35	0.02	-0.33
14	6	-0.12	-0.07	0.00	-0.11	-0.11	-0.02	-0.06	0.03	0.22
15	8	0.14	-0.08	0.04	0.08	-0.11	0.06	0.05	-0.07	-0.04
16	8	-0.04	0.12	-0.03	-0.05	0.10	-0.02	-0.04	0.03	-0.09
17	1	-0.24	0.34	-0.27	-0.28	0.35	-0.27	-0.16	0.29	0.23
18	6	-0.17	0.06	0.00	0.22	0.04	-0.14	-0.03	-0.23	0.17
19	8	0.18	0.05	0.12	-0.11	-0.12	-0.09	0.02	0.00	-0.10
20	8	-0.03	-0.14	-0.15	0.06	0.09	0.16	-0.01	0.06	-0.02
21	1	0.32	-0.11	0.02	-0.38	-0.02	0.00	-0.09	0.10	-0.10

25 26 27
A A A
Frequencies -- 772.9378 796.5451 801.4289
Red. masses -- 3.1258 4.3993 2.7506
Frc consts -- 1.1003 1.6446 1.0409
IR Inten -- 57.6221 35.2196 28.0627
Raman Activ -- 1.4994 0.9628 0.9992
Depolar (P) -- 0.1530 0.1079 0.7496
Depolar (U) -- 0.2654 0.1947 0.8569

Atom	AN	X	Y	Z	X	Y	Z	X	Y	Z
1	6	0.03	-0.03	0.07	0.02	-0.04	-0.06	0.00	0.04	-0.11
2	6	-0.05	0.09	0.04	0.01	0.01	-0.06	-0.01	0.04	-0.02
3	6	-0.01	0.05	0.04	-0.01	0.00	-0.14	0.00	0.01	0.01
4	6	0.00	0.00	-0.03	0.01	-0.02	0.07	0.03	0.00	0.20
5	6	-0.02	-0.04	0.00	-0.01	-0.02	0.15	-0.01	-0.03	-0.12
6	6	-0.04	-0.12	0.04	-0.05	-0.09	-0.02	-0.04	-0.03	-0.02
7	1	0.21	-0.06	-0.51	0.12	-0.01	0.49	0.09	0.08	0.55
8	1	-0.04	0.12	-0.46	0.04	0.06	0.58	-0.01	0.05	0.39
9	1	-0.02	-0.20	-0.39	-0.03	-0.11	0.12	-0.01	-0.01	0.58
10	6	0.22	0.02	-0.09	0.09	0.03	-0.06	0.18	0.01	-0.10
11	8	-0.06	-0.01	0.02	-0.04	0.01	-0.02	-0.05	0.03	0.02
12	8	-0.05	0.01	0.05	-0.02	0.00	0.03	-0.06	-0.03	-0.01
13	1	0.21	0.02	-0.05	0.11	0.02	0.08	0.13	-0.01	-0.03
14	6	0.01	-0.07	-0.15	-0.02	0.10	0.28	0.00	-0.05	-0.10
15	8	0.00	0.00	0.05	0.00	-0.03	-0.08	0.00	-0.01	0.04
16	8	0.00	0.02	0.04	0.01	-0.03	-0.07	-0.02	0.02	0.02
17	1	-0.01	-0.02	-0.14	-0.01	0.07	0.18	-0.03	0.01	-0.10
18	6	0.00	0.16	-0.10	0.06	0.23	-0.21	-0.03	-0.07	0.08
19	8	0.01	0.01	0.08	0.00	-0.04	0.08	0.00	0.03	-0.02
20	8	-0.02	-0.05	-0.02	-0.04	-0.06	0.00	0.01	0.01	-0.01
21	1	0.06	-0.12	0.09	-0.06	-0.12	0.03	0.06	0.07	-0.02

28 29 30
A A A
Frequencies -- 808.4555 859.2699 903.4019

Red. masses --	5.2266	3.7505	7.5643
Frc consts --	2.0127	1.6316	3.6373
IR Inten --	3.6265	3.3289	14.5839
Raman Activ --	2.2530	0.9744	1.7075
Depolar (P) --	0.1817	0.6181	0.7485
Depolar (U) --	0.3076	0.7640	0.8562

Atom	AN	X	Y	Z	X	Y	Z	X	Y	Z
1	6	-0.01	0.30	0.00	0.00	-0.01	-0.04	-0.02	0.02	0.00
2	6	0.23	-0.07	0.01	0.00	0.01	-0.10	-0.08	0.13	0.02
3	6	0.10	-0.10	0.02	0.03	0.01	0.24	0.27	0.06	-0.06
4	6	0.00	-0.12	-0.03	0.00	0.00	-0.19	0.32	0.01	0.03
5	6	-0.08	-0.10	-0.01	0.03	0.01	0.20	0.27	-0.06	-0.05
6	6	-0.23	-0.08	0.01	-0.01	-0.02	-0.12	-0.09	-0.13	0.02
7	1	-0.02	0.30	-0.15	0.04	0.04	0.73	0.37	0.02	-0.11
8	1	0.10	-0.32	-0.11	0.01	0.02	0.25	-0.19	-0.07	-0.06
9	1	-0.09	-0.31	-0.04	0.00	0.02	0.38	-0.18	0.01	0.00
10	6	-0.03	0.15	-0.03	-0.08	0.00	0.05	-0.20	0.00	0.08
11	8	-0.05	0.01	-0.13	0.02	-0.02	-0.01	0.04	0.02	-0.03
12	8	0.06	0.07	0.13	0.03	0.02	0.02	0.03	-0.02	-0.04
13	1	0.16	0.12	0.47	-0.04	0.01	-0.01	-0.03	-0.01	0.04
14	6	-0.05	-0.04	-0.01	0.01	-0.08	-0.21	-0.04	-0.08	0.08
15	8	0.03	-0.04	0.02	0.01	0.02	0.06	0.02	-0.13	0.04
16	8	-0.06	0.04	-0.01	-0.03	0.03	0.04	-0.18	0.10	-0.06
17	1	-0.12	0.11	-0.08	-0.02	-0.01	-0.07	-0.32	0.28	-0.12
18	6	0.07	-0.03	-0.02	0.02	0.08	-0.08	-0.10	0.02	0.11
19	8	-0.03	-0.04	-0.05	0.01	-0.02	0.03	0.01	0.12	0.10
20	8	0.06	0.03	0.07	-0.04	-0.03	-0.02	-0.15	-0.07	-0.16
21	1	-0.08	0.02	0.00	-0.06	-0.03	-0.03	0.15	-0.08	0.01

	31	32	33
	A	A	A
Frequencies --	967.2492	1017.9954	1095.2816
Red. masses --	1.3934	1.3457	4.2176
Frc consts --	0.7681	0.8216	2.9811
IR Inten --	0.1769	1.6356	27.2431
Raman Activ --	0.2015	0.1116	21.6552
Depolar (P) --	0.1315	0.0910	0.0821
Depolar (U) --	0.2324	0.1667	0.1517

Atom	AN	X	Y	Z	X	Y	Z	X	Y	Z
1	6	0.00	0.00	0.01	0.00	-0.01	-0.13	-0.01	0.07	0.00
2	6	0.01	0.01	0.11	0.00	0.01	0.10	-0.26	0.07	0.00
3	6	-0.01	0.00	-0.05	0.00	0.00	-0.01	0.10	-0.04	0.00
4	6	-0.01	0.00	0.00	0.00	0.00	-0.01	0.00	-0.23	0.02
5	6	0.00	0.00	0.04	0.00	0.00	0.00	-0.09	-0.04	0.01
6	6	0.00	-0.01	-0.13	0.01	0.01	0.07	0.27	0.08	-0.02
7	1	0.00	-0.01	-0.08	0.00	0.04	0.70	-0.01	0.09	-0.03
8	1	-0.01	-0.03	-0.64	-0.03	-0.04	-0.55	-0.49	-0.32	0.04
9	1	0.00	0.07	0.74	0.01	-0.04	-0.42	0.51	-0.28	0.09
10	6	0.00	0.00	0.00	0.00	0.00	0.00	-0.02	-0.04	-0.07
11	8	0.00	0.00	0.00	0.00	0.00	0.00	-0.03	0.05	-0.06
12	8	0.00	0.00	0.00	0.00	0.00	0.00	0.04	0.06	0.10
13	1	0.00	0.00	0.00	0.00	0.00	0.00	0.02	0.05	0.05
14	6	0.00	0.01	0.03	0.00	0.00	0.01	0.07	-0.01	0.02
15	8	0.00	0.00	-0.01	0.00	0.00	0.00	0.00	-0.02	0.01
16	8	0.01	-0.01	0.00	0.00	0.00	0.00	-0.06	0.04	-0.02
17	1	0.00	0.01	0.00	0.00	0.01	0.00	0.02	-0.06	0.02
18	6	0.01	0.01	-0.01	0.00	0.00	0.00	-0.05	0.02	-0.05
19	8	0.00	-0.01	0.00	0.00	0.00	0.00	0.00	-0.02	-0.01
20	8	-0.01	0.00	0.00	0.00	0.00	0.00	0.03	0.02	0.04
21	1	-0.02	0.02	-0.03	0.00	-0.01	0.01	0.10	0.06	0.05

	34	35	36

A A A

Frequencies -- 1141.3529 1162.0854 1184.1718

Red. masses -- 4.9331 2.4255 1.7790

Frc consts -- 3.7863 1.9299 1.4698

IR Inten -- 12.3168 50.3642 172.1896

Raman Activ -- 4.9784 2.5099 1.7496

Depolar (P) -- 0.2097 0.7052 0.7296

Depolar (U) -- 0.3467 0.8271 0.8437

Atom	AN	X	Y	Z	X	Y	Z	X	Y	Z
1	6	-0.03	0.22	-0.01	0.02	0.07	0.00	0.07	0.00	0.00
2	6	-0.09	-0.05	0.00	-0.05	-0.08	0.00	-0.06	-0.02	0.00
3	6	-0.19	-0.11	0.02	0.00	-0.01	0.00	-0.04	-0.05	0.01
4	6	-0.03	0.02	0.00	0.02	0.17	-0.02	0.06	-0.05	0.00
5	6	0.19	-0.13	-0.03	-0.03	0.02	0.02	0.00	0.02	0.01
6	6	0.12	-0.04	0.01	0.02	-0.07	0.00	-0.05	0.04	0.00
7	1	-0.17	0.24	-0.02	0.18	0.08	-0.01	0.79	0.02	-0.01
8	1	-0.23	-0.29	0.03	-0.28	-0.51	0.03	-0.12	-0.12	0.02
9	1	0.29	-0.29	0.00	0.27	-0.48	0.04	-0.19	0.28	-0.02
10	6	0.01	0.02	0.01	0.02	0.09	0.09	-0.02	-0.02	-0.01
11	8	0.00	-0.01	0.01	0.03	-0.05	0.06	0.00	0.01	-0.02
12	8	-0.01	-0.01	-0.02	-0.05	-0.06	-0.13	0.01	0.01	0.02
13	1	0.01	0.00	0.04	0.08	-0.01	0.19	0.00	0.01	-0.02
14	6	-0.13	0.05	-0.02	0.02	0.00	0.01	-0.11	0.03	-0.01
15	8	0.02	0.09	-0.03	0.00	0.00	0.00	0.01	0.06	-0.02
16	8	0.12	-0.09	0.04	-0.01	0.01	-0.01	0.08	-0.08	0.04
17	1	-0.04	0.12	-0.05	0.02	-0.03	0.02	-0.19	0.27	-0.12
18	6	0.19	0.05	0.10	-0.09	0.00	-0.06	-0.06	-0.01	-0.02
19	8	-0.04	0.09	0.08	0.01	-0.03	-0.03	0.01	-0.02	-0.02
20	8	-0.10	-0.07	-0.12	0.02	0.03	0.05	0.01	0.02	0.03
21	1	-0.42	-0.12	-0.27	0.34	0.09	0.18	0.17	0.03	0.11

37 38 39

A A A

Frequencies -- 1208.5482 1229.8966 1238.4330

Red. masses -- 1.5143 1.8399 1.6048

Frc consts -- 1.3032 1.6397 1.4501

IR Inten -- 123.0617 108.2180 214.7757

Raman Activ -- 4.2659 7.2294 20.9915

Depolar (P) -- 0.7361 0.2669 0.0467

Depolar (U) -- 0.8480 0.4213 0.0893

Atom	AN	X	Y	Z	X	Y	Z	X	Y	Z
1	6	0.05	0.00	0.00	0.00	-0.06	0.00	-0.01	0.03	0.00
2	6	-0.02	-0.03	0.00	-0.01	0.03	0.00	-0.03	0.04	0.00
3	6	-0.03	-0.04	0.00	0.10	0.06	0.00	-0.07	-0.11	0.01
4	6	-0.04	0.01	0.00	0.00	-0.09	-0.01	0.03	-0.10	-0.01
5	6	-0.05	0.04	-0.01	-0.09	0.01	0.00	0.06	-0.03	0.00
6	6	-0.02	0.02	0.00	0.02	0.03	0.00	0.00	0.02	0.00
7	1	0.61	0.02	0.00	-0.09	-0.07	0.00	0.03	0.04	0.00
8	1	-0.12	-0.21	0.00	-0.03	-0.01	0.01	0.19	0.48	-0.04
9	1	-0.11	0.17	-0.02	0.00	0.06	0.00	-0.06	0.14	-0.01
10	6	0.00	0.01	0.01	0.02	0.06	0.04	0.00	0.03	0.02
11	8	0.00	-0.01	0.01	0.02	-0.02	0.05	0.02	-0.01	0.03
12	8	-0.01	-0.01	-0.02	-0.05	0.01	-0.11	-0.03	0.01	-0.07
13	1	0.01	0.00	0.04	0.25	0.12	0.63	0.20	0.10	0.50
14	6	0.08	-0.01	0.01	-0.03	0.00	-0.01	-0.01	0.01	0.00
15	8	0.00	-0.04	0.01	-0.01	0.02	-0.01	0.01	-0.01	0.00
16	8	-0.05	0.08	-0.03	0.01	-0.05	0.02	0.00	0.04	-0.02
17	1	0.28	-0.35	0.15	-0.28	0.32	-0.14	0.27	-0.31	0.14
18	6	0.07	0.00	0.04	0.03	0.00	0.02	-0.01	0.00	-0.01
19	8	-0.01	0.02	0.02	0.00	0.02	0.02	0.00	-0.01	-0.01
20	8	0.01	-0.03	-0.03	0.03	-0.02	-0.01	-0.03	0.01	0.01

21	1	-0.44	-0.08	-0.24	-0.43	-0.07	-0.23	0.35	0.05	0.19

		40			41			42		
		A			A			A		
Frequencies --		1261.1684			1327.5501			1349.4504		
Red. masses --		1.7127			4.3469			3.5824		
Frc consts --		1.6050			4.5137			3.8436		
IR Inten --		56.6536			129.9620			372.3619		
Raman Activ --		3.1289			3.7375			7.5246		
Depolar (P) --		0.3708			0.4873			0.4210		
Depolar (U) --		0.5410			0.6553			0.5925		
Atom	AN	X	Y	Z	X	Y	Z	X	Y	Z
1	6	0.03	0.01	0.00	0.17	0.04	0.00	0.11	-0.05	0.00
2	6	0.03	-0.04	0.00	-0.09	-0.14	0.01	-0.06	-0.10	0.01
3	6	-0.01	0.12	-0.01	-0.13	0.15	0.00	-0.03	0.10	0.00
4	6	-0.08	-0.02	0.00	0.27	0.02	-0.01	0.11	-0.02	0.00
5	6	0.04	-0.16	0.00	-0.06	-0.24	0.01	-0.17	0.02	0.00
6	6	0.04	0.05	0.00	-0.07	0.13	-0.01	-0.09	0.12	0.00
7	1	-0.12	0.01	0.00	-0.39	0.03	0.01	0.09	-0.06	0.01
8	1	-0.20	-0.49	0.03	0.11	0.25	-0.04	0.09	0.18	-0.02
9	1	-0.30	0.62	-0.05	0.03	-0.04	0.02	0.27	-0.48	0.02
10	6	0.03	0.01	-0.01	-0.03	0.00	0.01	-0.02	0.00	0.00
11	8	0.00	-0.01	0.01	0.00	0.01	-0.01	0.00	0.01	0.00
12	8	-0.01	0.00	-0.01	0.01	-0.01	0.00	0.00	0.00	0.00
13	1	0.04	0.02	0.11	-0.01	-0.02	-0.07	0.00	0.00	-0.02
14	6	0.04	-0.01	0.00	-0.05	0.00	-0.01	0.06	-0.01	0.00
15	8	-0.01	-0.02	0.01	0.00	-0.03	0.01	-0.01	0.00	0.00
16	8	-0.02	0.01	0.00	0.03	0.03	-0.01	-0.02	-0.01	0.00
17	1	-0.02	0.01	-0.01	0.24	-0.25	0.11	-0.11	0.13	-0.06
18	6	0.03	0.03	-0.01	-0.15	0.01	-0.07	0.28	0.01	0.11
19	8	-0.01	0.00	0.00	0.00	0.03	0.04	-0.03	-0.03	-0.04
20	8	-0.04	0.00	0.00	0.11	-0.01	0.03	-0.14	-0.01	-0.06
21	1	0.34	0.03	0.19	-0.51	-0.06	-0.26	0.56	0.06	0.27

		43			44			45		
		A			A			A		
Frequencies --		1390.2970			1400.1353			1502.8715		
Red. masses --		2.4830			3.2260			2.5155		
Frc consts --		2.8278			3.7261			3.3474		
IR Inten --		93.8464			76.5178			7.6677		
Raman Activ --		11.7423			6.2536			0.2883		
Depolar (P) --		0.1190			0.4721			0.3540		
Depolar (U) --		0.2127			0.6414			0.5229		
Atom	AN	X	Y	Z	X	Y	Z	X	Y	Z
1	6	0.01	0.06	0.00	-0.01	-0.03	0.00	0.20	0.00	0.00
2	6	-0.05	-0.02	0.00	-0.01	0.07	0.00	-0.03	0.14	-0.01
3	6	-0.02	-0.12	0.01	0.13	0.01	0.00	-0.10	-0.11	0.01
4	6	-0.01	-0.01	0.00	0.01	-0.09	0.00	0.11	-0.01	0.00
5	6	-0.02	-0.09	0.00	-0.09	-0.01	0.00	-0.07	0.12	0.00
6	6	0.03	0.02	0.00	0.03	0.01	0.00	-0.03	-0.13	0.01
7	1	0.07	0.07	0.00	-0.09	-0.04	0.00	-0.68	-0.02	0.01
8	1	0.18	0.43	-0.03	-0.08	-0.06	0.01	-0.31	-0.35	0.03
9	1	-0.09	0.23	-0.02	-0.06	0.15	-0.01	-0.31	0.28	-0.02
10	6	0.04	0.16	0.10	0.06	0.24	0.14	-0.02	0.00	0.01
11	8	-0.01	-0.01	-0.03	-0.02	-0.01	-0.04	0.00	0.00	-0.01
12	8	-0.01	-0.07	-0.02	-0.01	-0.09	-0.02	0.00	0.00	0.00
13	1	-0.16	-0.12	-0.43	-0.24	-0.17	-0.62	0.01	0.00	0.00
14	6	0.21	-0.03	0.03	-0.23	0.03	-0.02	0.04	-0.01	0.01
15	8	-0.02	0.05	-0.02	0.03	-0.03	0.01	0.00	0.02	-0.01
16	8	-0.07	-0.03	0.00	0.07	0.02	0.00	-0.01	0.00	0.00
17	1	-0.39	0.41	-0.18	0.34	-0.36	0.16	-0.03	0.04	-0.02
18	6	-0.01	0.01	-0.02	0.06	0.00	0.02	0.02	-0.01	0.02

```
 19  8    0.00   0.01   0.01    -0.01  -0.01  -0.01     0.00  -0.01  -0.01
 20  8    0.01   0.00   0.00    -0.02   0.00  -0.01     0.00   0.00   0.00
 21  1   -0.04  -0.01  -0.02     0.08   0.00   0.04     0.01  -0.01   0.01
                    46                     47                     48
                     A                      A                      A
 Frequencies --   1517.2578              1660.4119              1664.3328
 Red. masses --      4.3903                 7.5967                 6.2507
 Frc consts  --      5.9547                12.3398                10.2014
 IR Inten    --      8.6855                36.5590                 5.8748
 Raman Activ --      5.5714                28.7092                19.0537
 Depolar (P) --      0.3187                 0.6951                 0.7434
 Depolar (U) --      0.4833                 0.8202                 0.8528
  Atom  AN      X      Y      Z        X      Y      Z        X      Y      Z
   1   6    0.00   0.10  -0.01     0.41   0.00  -0.01    -0.04  -0.18   0.01
   2   6   -0.10  -0.07   0.00    -0.23  -0.09   0.01     0.14   0.33  -0.02
   3   6    0.23  -0.13   0.00     0.28  -0.08   0.00     0.02  -0.28   0.02
   4   6    0.01   0.28   0.00    -0.37   0.01   0.01     0.02   0.18   0.00
   5   6   -0.23  -0.11   0.00     0.26   0.03   0.00    -0.04  -0.29   0.00
   6   6    0.12  -0.10   0.00    -0.25   0.14   0.00    -0.11   0.31  -0.01
   7   1   -0.05   0.12   0.00    -0.45  -0.03   0.01     0.05  -0.21   0.01
   8   1    0.17   0.48  -0.03    -0.06   0.28  -0.01    -0.27  -0.42   0.03
   9   1   -0.27   0.55  -0.04     0.02  -0.34   0.02     0.31  -0.36   0.03
  10   6   -0.03  -0.13  -0.08     0.03   0.01  -0.03    -0.02  -0.03  -0.03
  11   8    0.01   0.00   0.03     0.00  -0.01   0.02     0.01   0.00   0.02
  12   8    0.01   0.03   0.02     0.00   0.00   0.00     0.00   0.01   0.01
  13   1    0.06   0.04   0.16    -0.02   0.00   0.01     0.01   0.01   0.02
  14   6   -0.11   0.01   0.00    -0.06  -0.01   0.01    -0.01  -0.02   0.02
  15   8    0.01   0.00   0.00     0.01   0.01  -0.01     0.01   0.04  -0.01
  16   8    0.01  -0.01   0.00     0.00   0.00   0.00     0.00   0.00   0.00
  17   1    0.04  -0.05   0.02     0.02  -0.03   0.01     0.00  -0.01  -0.01
  18   6    0.08   0.02   0.01    -0.05  -0.01   0.00     0.01   0.01  -0.02
  19   8   -0.01  -0.01   0.00     0.00   0.00   0.00    -0.01   0.01   0.02
  20   8   -0.01  -0.01  -0.01     0.00   0.00   0.00     0.00   0.00   0.00
  21   1    0.06  -0.01   0.04    -0.02   0.00  -0.01     0.02   0.01   0.00
                    49                     50                     51
                     A                      A                      A
 Frequencies --   1851.5188              1859.3550              1888.1898
 Red. masses --     10.6255                10.9588                11.1433
 Frc consts  --     21.4613                22.3223                23.4075
 IR Inten    --    239.4496               424.8398               341.6463
 Raman Activ --     42.3414                23.6627                40.9328
 Depolar (P) --      0.3102                 0.1765                 0.1405
 Depolar (U) --      0.4735                 0.3000                 0.2464
  Atom  AN      X      Y      Z        X      Y      Z        X      Y      Z
   1   6    0.02   0.00   0.00     0.00   0.00   0.00    -0.01   0.00   0.00
   2   6   -0.01   0.02   0.00     0.00   0.00   0.00     0.00   0.01   0.00
   3   6   -0.02  -0.10   0.01     0.01   0.01   0.00    -0.01  -0.02   0.00
   4   6   -0.01   0.04  -0.01    -0.02   0.06  -0.02     0.00   0.01   0.00
   5   6    0.01  -0.01   0.00     0.01  -0.02   0.00     0.03  -0.06  -0.02
   6   6   -0.01   0.01   0.00    -0.01   0.00   0.00     0.01   0.01   0.00
   7   1   -0.02  -0.01   0.00     0.00   0.00   0.00     0.00   0.00   0.00
   8   1   -0.04  -0.01   0.00     0.00  -0.01   0.00    -0.01  -0.01   0.00
   9   1    0.01  -0.02   0.00     0.01  -0.02   0.00     0.02  -0.01   0.00
  10   6    0.06  -0.06   0.11     0.28  -0.38   0.57    -0.04   0.06  -0.07
  11   8   -0.03   0.04  -0.07    -0.18   0.23  -0.37     0.03  -0.03   0.05
  12   8   -0.01   0.00  -0.01    -0.03   0.01  -0.07     0.00  -0.01   0.00
  13   1    0.04   0.01   0.05     0.14   0.07   0.38    -0.04  -0.02  -0.06
  14   6    0.09   0.68  -0.24    -0.02  -0.12   0.04     0.02   0.09  -0.03
  15   8   -0.04  -0.43   0.15     0.01   0.07  -0.03    -0.01  -0.06   0.02
  16   8    0.01  -0.06   0.02     0.00   0.01   0.00     0.00  -0.01   0.00
```

17	1	-0.28	0.32	-0.14	0.05	-0.05	0.03	-0.04	0.04	-0.02
18	6	0.02	-0.06	-0.05	-0.02	0.08	0.08	-0.18	0.55	0.49
19	8	-0.01	0.04	0.04	0.02	-0.05	-0.05	0.11	-0.35	-0.31
20	8	0.00	0.00	0.00	0.00	0.00	-0.01	0.03	-0.03	-0.03
21	1	0.03	0.01	0.01	-0.05	-0.02	-0.01	-0.35	-0.06	-0.17

	52	53	54
	A	A	A
Frequencies --	3197.2236	3219.9720	3243.3981
Red. masses --	1.0897	1.0934	1.0949
Frc consts --	6.5629	6.6793	6.7864
IR Inten --	1.1114	4.2330	1.3453
Raman Activ --	61.3935	125.7513	121.4530
Depolar (P) --	0.4796	0.2932	0.1767
Depolar (U) --	0.6483	0.4535	0.3004

Atom	AN	X	Y	Z	X	Y	Z	X	Y	Z
1	6	0.00	0.03	0.00	0.00	-0.08	0.00	0.00	0.03	0.00
2	6	0.00	0.00	0.00	-0.02	0.01	0.00	-0.07	0.04	0.00
3	6	0.00	0.00	0.00	0.00	0.00	0.00	0.00	0.00	0.00
4	6	0.00	0.00	0.00	0.00	0.00	0.00	0.00	0.00	0.00
5	6	0.00	0.00	0.00	0.00	0.00	0.00	0.00	0.00	0.00
6	6	-0.07	-0.04	0.00	-0.03	-0.01	0.00	0.01	0.00	0.00
7	1	0.01	-0.35	0.02	-0.03	0.88	-0.05	0.00	-0.30	0.02
8	1	-0.05	0.03	0.00	0.27	-0.15	0.00	0.84	-0.44	0.00
9	1	0.79	0.48	-0.05	0.30	0.18	-0.02	-0.05	-0.03	0.00
10	6	0.00	0.00	0.00	0.00	0.00	0.00	0.00	0.00	0.00
11	8	0.00	0.00	0.00	0.00	0.00	0.00	0.00	0.00	0.00
12	8	0.00	0.00	0.00	0.00	0.00	0.00	0.00	0.00	0.00
13	1	0.00	0.00	0.00	0.00	0.00	0.00	0.00	0.00	0.00
14	6	0.00	0.00	0.00	0.00	0.00	0.00	0.00	0.00	0.00
15	8	0.00	0.00	0.00	0.00	0.00	0.00	0.00	0.00	0.00
16	8	0.00	0.00	0.00	0.00	0.00	0.00	0.00	0.00	0.00
17	1	0.00	0.00	0.00	0.00	0.00	0.00	0.00	0.00	0.00
18	6	0.00	0.00	0.00	0.00	0.00	0.00	0.00	0.00	0.00
19	8	0.00	0.00	0.00	0.00	0.00	0.00	0.00	0.00	0.00
20	8	0.00	0.00	0.00	0.00	0.00	0.00	0.00	0.00	0.00
21	1	0.00	0.00	0.00	0.00	0.00	0.00	0.00	0.00	0.00

	55	56	57
	A	A	A
Frequencies --	3839.4755	3849.4091	3884.8297
Red. masses --	1.0643	1.0648	1.0662
Frc consts --	9.2439	9.2959	9.4804
IR Inten --	112.5035	128.7161	59.7886
Raman Activ --	123.1417	131.3467	46.3328
Depolar (P) --	0.2735	0.2408	0.1464
Depolar (U) --	0.4295	0.3882	0.2555

Atom	AN	X	Y	Z	X	Y	Z	X	Y	Z
1	6	0.00	0.00	0.00	0.00	0.00	0.00	0.00	0.00	0.00
2	6	0.00	0.00	0.00	0.00	0.00	0.00	0.00	0.00	0.00
3	6	0.00	0.00	0.00	0.00	0.00	0.00	0.00	0.00	0.00
4	6	0.00	0.00	0.00	0.00	0.00	0.00	0.00	0.00	0.00
5	6	0.00	0.00	0.00	0.00	0.00	0.00	0.00	0.00	0.00
6	6	0.00	0.00	0.00	0.00	0.00	0.00	0.00	0.00	0.00
7	1	0.00	0.00	0.00	0.00	0.00	0.00	0.00	0.00	0.00
8	1	0.00	0.00	0.00	0.00	0.00	0.00	0.00	0.00	0.00
9	1	0.00	0.00	0.00	0.00	0.00	0.00	0.00	0.00	0.00
10	6	0.00	0.00	0.00	0.00	0.00	0.00	0.00	0.00	0.00
11	8	0.00	0.00	0.00	0.00	0.00	0.00	0.00	0.00	0.00
12	8	0.01	-0.06	0.01	0.00	0.00	0.00	0.00	0.00	0.00
13	1	-0.08	0.99	-0.11	0.00	0.04	0.00	0.00	0.00	0.00
14	6	0.00	0.00	0.00	0.00	0.00	0.00	0.00	0.00	0.00

15	8	0.00	0.00	0.00	0.00	0.00	0.00	0.00	0.00	0.00
16	8	0.00	0.00	0.00	-0.05	-0.03	0.01	0.00	0.00	0.00
17	1	-0.03	-0.02	0.01	0.81	0.55	-0.15	0.00	0.00	0.00
18	6	0.00	0.00	0.00	0.00	0.00	0.00	0.00	0.00	0.00
19	8	0.00	0.00	0.00	0.00	0.00	0.00	0.00	0.00	0.00
20	8	0.00	0.00	0.00	0.00	0.00	0.00	0.02	-0.04	-0.04
21	1	0.00	0.00	0.00	0.00	0.00	0.00	-0.39	0.59	0.70

HEM-

Harmonic frequencies (cm**-1), IR intensities (KM/Mole), Raman scattering activities (A**4/AMU), depolarization ratios for plane and unpolarized incident light, reduced masses (AMU), force constants (mDyne/A), and normal coordinates:

		1			2			3		
		A			A			A		
Frequencies --		29.8182			49.3968			98.5185		
Red. masses --		12.3349			9.8566			11.2020		
Frc consts --		0.0065			0.0142			0.0641		
IR Inten --		0.4852			1.4278			8.4491		
Raman Activ --		0.8370			0.6831			1.2867		
Depolar (P) --		0.7500			0.7500			0.7500		
Depolar (U) --		0.8571			0.8571			0.8571		
Atom	AN	X	Y	Z	X	Y	Z	X	Y	Z
1	6	0.00	0.01	0.00	0.02	0.00	0.05	-0.06	0.00	0.12
2	6	0.00	0.02	0.01	0.01	-0.01	0.05	-0.05	0.02	0.06
3	6	0.00	0.02	0.00	0.00	-0.01	0.16	0.00	0.02	-0.05
4	1	0.00	0.02	0.01	0.00	-0.01	0.18	0.01	0.04	-0.11
5	6	0.00	0.03	0.00	0.00	0.00	0.24	0.01	0.00	-0.09
6	1	0.00	0.03	0.00	-0.01	0.00	0.34	0.03	0.00	-0.17
7	6	0.00	0.02	0.00	0.00	0.01	0.16	0.00	-0.02	-0.05
8	8	-0.16	-0.27	0.10	-0.21	-0.08	-0.12	-0.09	0.19	-0.18
9	1	-0.17	-0.27	0.13	-0.18	-0.07	-0.23	-0.10	0.13	-0.30
10	8	-0.19	0.01	0.40	0.00	0.02	0.18	0.24	0.20	0.14
11	6	0.00	0.03	0.03	0.02	-0.02	-0.13	-0.08	0.10	-0.01
12	8	-0.10	0.20	0.14	0.19	-0.10	-0.30	-0.08	0.32	0.01
13	6	0.00	0.02	-0.01	0.01	0.01	0.05	-0.05	-0.02	0.06
14	6	0.00	0.01	0.00	0.01	0.00	0.14	0.12	0.00	0.14
15	6	0.00	0.03	-0.03	0.02	0.02	-0.13	-0.08	-0.10	-0.01
16	8	0.19	0.01	-0.40	0.00	-0.02	0.18	0.24	-0.20	0.14
17	1	0.00	0.02	-0.01	0.00	0.01	0.18	0.01	-0.04	-0.11
18	8	0.10	0.20	-0.14	0.19	0.10	-0.30	-0.08	-0.32	0.01
19	8	0.16	-0.27	-0.10	-0.21	0.08	-0.12	-0.09	-0.19	-0.18
20	1	0.17	-0.27	-0.13	-0.18	0.07	-0.23	-0.10	-0.13	-0.30

		4			5			6		
		A			A			A		
Frequencies --		125.0010			146.3385			149.8282		
Red. masses --		9.4156			6.8782			5.4551		
Frc consts --		0.0867			0.0868			0.0722		
IR Inten --		0.1607			6.9506			3.0346		
Raman Activ --		1.4554			2.2779			3.0303		
Depolar (P) --		0.7116			0.7500			0.7486		
Depolar (U) --		0.8315			0.8571			0.8562		
Atom	AN	X	Y	Z	X	Y	Z	X	Y	Z
1	6	0.00	-0.11	0.00	0.04	0.00	0.25	0.00	0.06	0.00
2	6	0.00	-0.09	0.06	0.03	-0.01	0.17	-0.01	0.05	0.18
3	6	0.01	-0.09	0.07	0.01	-0.02	-0.08	-0.01	0.05	0.27
4	1	0.00	-0.09	0.11	0.01	-0.04	-0.23	-0.02	0.06	0.47
5	6	0.00	-0.08	0.00	0.01	0.00	-0.23	0.00	0.04	0.00
6	1	0.00	-0.08	0.00	0.00	0.00	-0.48	0.00	0.04	0.00

7	6	-0.01	-0.09	-0.07	0.01	0.02	-0.08	0.01	0.05	-0.27
8	8	0.21	-0.05	0.08	-0.05	0.18	-0.05	0.09	-0.13	0.03
9	1	0.35	0.17	0.16	0.02	0.25	-0.21	0.00	-0.22	0.20
10	8	0.07	-0.13	-0.13	-0.14	-0.20	-0.02	0.00	0.07	0.00
11	6	-0.09	0.08	0.02	0.05	-0.06	0.04	0.03	-0.03	0.05
12	8	-0.07	0.37	0.04	0.13	0.03	-0.02	0.15	-0.04	-0.06
13	6	0.00	-0.09	-0.06	0.03	0.01	0.17	0.01	0.05	-0.18
14	6	0.00	-0.13	0.00	-0.07	0.00	0.08	0.00	0.07	0.00
15	6	0.09	0.08	-0.02	0.05	0.06	0.04	-0.03	-0.03	-0.05
16	8	-0.07	-0.13	0.13	-0.14	0.20	-0.02	0.00	0.07	0.00
17	1	0.00	-0.09	-0.11	0.01	0.04	-0.23	0.02	0.06	-0.48
18	8	0.07	0.37	-0.04	0.13	-0.03	-0.02	-0.15	-0.04	0.06
19	8	-0.21	-0.05	-0.08	-0.05	-0.18	-0.05	-0.09	-0.13	-0.03
20	1	-0.35	0.17	-0.16	0.02	-0.25	-0.21	0.00	-0.22	-0.20

	7	8	9
	A	A	A
Frequencies --	172.8208	179.6967	250.8685
Red. masses --	6.1474	6.3499	4.6950
Frc consts --	0.1082	0.1208	0.1741
IR Inten --	7.8566	20.3580	0.0709
Raman Activ --	1.6212	0.0026	3.2981
Depolar (P) --	0.7449	0.7500	0.7500
Depolar (U) --	0.8538	0.8571	0.8571

Atom	AN	X	Y	Z	X	Y	Z	X	Y	Z
1	6	0.00	0.06	0.00	0.01	0.00	0.15	-0.15	0.00	0.10
2	6	0.00	0.05	-0.05	-0.01	-0.03	0.11	-0.08	0.13	0.09
3	6	0.00	0.05	-0.09	-0.03	-0.03	-0.03	0.09	0.14	0.01
4	1	0.00	0.05	-0.16	-0.03	-0.05	-0.11	0.16	0.26	-0.02
5	6	0.00	0.05	0.00	-0.04	0.00	-0.13	0.18	0.00	-0.05
6	1	0.00	0.05	0.00	-0.07	0.00	-0.29	0.32	0.00	-0.14
7	6	0.00	0.05	0.09	-0.03	0.03	-0.03	0.09	-0.14	0.01
8	8	-0.16	-0.20	0.11	-0.20	-0.14	0.10	0.01	0.02	0.01
9	1	-0.31	-0.40	0.18	-0.34	-0.35	0.11	0.25	0.35	-0.10
10	8	0.11	0.05	-0.24	0.18	-0.02	-0.19	0.01	0.12	-0.07
11	6	0.04	-0.02	-0.04	-0.03	0.02	0.07	-0.06	0.07	0.03
12	8	-0.02	0.04	0.01	0.05	-0.01	-0.01	0.00	-0.15	-0.04
13	6	0.00	0.05	0.05	-0.01	0.03	0.11	-0.08	-0.13	0.09
14	6	0.00	0.06	0.00	0.13	0.00	-0.07	-0.09	0.00	-0.01
15	6	-0.04	-0.02	0.04	-0.03	-0.02	0.07	-0.06	-0.07	0.03
16	8	-0.11	0.05	0.24	0.18	0.02	-0.18	0.01	-0.12	-0.07
17	1	0.00	0.05	0.16	-0.03	0.05	-0.11	0.16	-0.26	-0.02
18	8	0.02	0.04	-0.01	0.05	0.01	-0.01	0.00	0.15	-0.04
19	8	0.16	-0.20	-0.11	-0.20	0.14	0.10	0.01	-0.02	0.01
20	1	0.32	-0.40	-0.18	-0.34	0.35	0.11	0.25	-0.35	-0.10

	10	11	12
	A	A	A
Frequencies --	280.6779	339.8337	368.5589
Red. masses --	9.8108	5.2317	10.5263
Frc consts --	0.4554	0.3560	0.8424
IR Inten --	0.5797	0.0492	1.8985
Raman Activ --	4.3147	0.6420	0.0087
Depolar (P) --	0.5359	0.2337	0.7500
Depolar (U) --	0.6978	0.3789	0.8571

Atom	AN	X	Y	Z	X	Y	Z	X	Y	Z
1	6	0.00	0.10	0.00	0.00	0.00	0.00	-0.02	0.00	0.40
2	6	-0.10	-0.04	-0.03	0.02	0.01	0.10	-0.05	0.02	-0.04
3	6	-0.05	-0.05	-0.09	0.01	0.01	-0.26	0.03	0.02	-0.05
4	1	-0.01	0.02	-0.17	0.01	-0.01	-0.55	0.06	0.06	-0.21
5	6	0.00	-0.11	0.00	0.00	0.02	0.00	0.05	0.00	0.19
6	1	0.00	-0.10	0.00	0.00	0.02	0.00	0.07	0.00	0.33

7	6	0.05	-0.05	0.09	-0.01	0.01	0.26	0.03	-0.02	-0.05
8	8	0.23	-0.12	0.00	0.11	-0.07	-0.08	0.11	-0.08	-0.09
9	1	0.37	0.08	0.04	0.04	-0.11	0.11	0.14	0.01	0.08
10	8	0.02	0.29	0.01	-0.01	0.01	0.02	0.13	-0.28	-0.01
11	6	-0.15	-0.12	0.00	0.06	0.00	0.14	-0.09	0.04	-0.15
12	8	-0.23	-0.10	0.07	0.20	0.04	0.02	-0.24	-0.08	-0.04
13	6	0.10	-0.04	0.03	-0.02	0.01	-0.10	-0.05	-0.02	-0.04
14	6	0.00	0.25	0.00	0.00	0.01	0.00	0.14	0.00	0.24
15	6	0.15	-0.12	0.00	-0.06	0.00	-0.14	-0.09	-0.04	-0.15
16	8	-0.02	0.29	-0.01	0.01	0.01	-0.02	0.13	0.28	-0.01
17	1	0.01	0.02	0.17	-0.01	-0.01	0.55	0.06	-0.06	-0.21
18	8	0.23	-0.10	-0.07	-0.20	0.04	-0.02	-0.24	0.08	-0.04
19	8	-0.23	-0.12	0.00	-0.11	-0.07	0.08	0.11	0.08	-0.09
20	1	-0.37	0.08	-0.04	-0.04	-0.11	-0.11	0.14	-0.01	0.08

	13	14	15
	A	A	A
Frequencies --	372.9349	398.2893	483.2768
Red. masses --	6.3347	5.9428	1.4901
Frc consts --	0.5191	0.5554	0.2050
IR Inten --	3.0463	3.1098	63.9376
Raman Activ --	0.3561	5.0904	0.3263
Depolar (P) --	0.7500	0.2022	0.7500
Depolar (U) --	0.8571	0.3364	0.8571

Atom	AN	X	Y	Z	X	Y	Z	X	Y	Z
1	6	0.08	0.00	-0.18	0.00	0.06	0.00	0.02	0.00	-0.08
2	6	0.04	-0.05	-0.06	-0.04	0.14	0.04	-0.01	-0.01	-0.03
3	6	0.23	-0.03	0.01	0.00	0.17	-0.04	0.00	-0.01	0.00
4	1	0.28	0.07	0.11	0.01	0.19	-0.09	-0.01	-0.02	0.05
5	6	0.25	0.00	-0.02	0.00	0.19	0.00	-0.01	0.00	0.00
6	1	0.19	0.00	0.02	0.00	0.20	0.00	-0.03	0.00	0.04
7	6	0.23	0.03	0.01	0.00	0.17	0.04	0.00	0.01	0.00
8	8	-0.18	0.12	-0.01	0.12	-0.03	0.02	-0.05	-0.03	0.04
9	1	-0.37	-0.17	-0.09	-0.16	-0.40	0.24	0.38	0.56	-0.16
10	8	0.00	-0.06	0.05	-0.03	-0.19	-0.01	0.02	-0.05	0.01
11	6	-0.06	-0.08	0.03	-0.13	0.09	0.04	-0.03	0.03	0.03
12	8	-0.13	-0.10	0.08	-0.13	-0.12	0.02	0.01	0.02	-0.01
13	6	0.04	0.05	-0.06	0.04	0.14	-0.04	-0.01	0.01	-0.03
14	6	0.07	0.00	-0.04	0.00	-0.13	0.00	0.06	0.00	-0.01
15	6	-0.06	0.08	0.03	0.13	0.09	-0.04	-0.03	-0.03	0.03
16	8	0.00	0.06	0.05	0.03	-0.19	0.01	0.02	0.05	0.01
17	1	0.28	-0.07	0.11	-0.01	0.19	0.09	-0.01	0.02	0.05
18	8	-0.13	0.10	0.08	0.13	-0.12	-0.02	0.01	-0.02	-0.01
19	8	-0.18	-0.12	-0.01	-0.12	-0.03	-0.02	-0.05	0.03	0.04
20	1	-0.37	0.17	-0.09	0.16	-0.40	-0.24	0.38	-0.56	-0.16

	16	17	18
	A	A	A
Frequencies --	485.5103	531.5817	571.8407
Red. masses --	1.3870	4.1566	4.9356
Frc consts --	0.1926	0.6920	0.9509
IR Inten --	96.2767	0.9099	20.2317
Raman Activ --	1.0209	0.1797	0.6298
Depolar (P) --	0.4918	0.7500	0.7500
Depolar (U) --	0.6593	0.8571	0.8571

Atom	AN	X	Y	Z	X	Y	Z	X	Y	Z
1	6	0.00	-0.02	0.00	0.03	0.00	-0.10	-0.08	0.00	-0.19
2	6	-0.01	-0.04	0.00	-0.01	-0.02	0.12	-0.06	0.15	-0.07
3	6	0.00	-0.05	0.00	0.00	-0.03	0.22	-0.13	0.18	0.01
4	1	0.00	-0.04	0.01	-0.01	-0.04	0.28	-0.11	0.23	0.23
5	6	0.00	-0.07	0.00	0.00	0.00	-0.27	-0.03	0.00	-0.05
6	1	0.00	-0.07	0.00	-0.03	0.00	-0.73	0.23	0.00	0.08

7	6	0.00	-0.05	0.00	0.00	0.03	0.22	-0.13	-0.18	0.01
8	8	0.02	0.05	-0.03	0.08	-0.05	-0.05	0.00	0.07	-0.01
9	1	-0.41	-0.55	0.10	0.06	-0.03	0.11	-0.27	-0.34	-0.02
10	8	0.00	0.03	0.01	0.00	-0.04	0.04	0.07	-0.15	0.04
11	6	-0.04	0.03	0.00	-0.04	0.02	-0.06	0.02	0.07	0.08
12	8	-0.01	0.03	-0.02	-0.08	-0.01	-0.04	0.08	-0.02	0.01
13	6	0.01	-0.04	0.00	-0.01	0.02	0.12	-0.06	-0.15	-0.07
14	6	0.00	0.02	0.00	0.07	0.00	-0.06	0.11	0.00	0.04
15	6	0.04	0.03	0.00	-0.04	-0.02	-0.06	0.02	-0.07	0.08
16	8	0.00	0.03	-0.01	0.00	0.04	0.04	0.07	0.15	0.04
17	1	0.00	-0.04	-0.01	-0.01	0.04	0.28	-0.11	-0.23	0.23
18	8	0.01	0.03	0.02	-0.08	0.01	-0.04	0.08	0.02	0.01
19	8	-0.02	0.05	0.03	0.08	0.05	-0.05	0.00	-0.07	-0.01
20	1	0.41	-0.55	-0.10	0.06	0.03	0.11	-0.27	0.34	-0.02

19 20 21
A A A
Frequencies -- 647.0405 660.3370 684.1683
Red. masses -- 5.3789 4.3745 5.8317
Frc consts -- 1.3268 1.1239 1.6083
IR Inten -- 0.6527 141.1826 0.2017
Raman Activ -- 1.2128 0.8279 9.8004
Depolar (P) -- 0.5851 0.7500 0.0474
Depolar (U) -- 0.7382 0.8571 0.0905

Atom	AN	X	Y	Z	X	Y	Z	X	Y	Z
1	6	0.00	0.01	0.00	0.12	0.00	0.05	0.00	0.21	0.00
2	6	-0.06	-0.01	0.34	0.14	0.06	0.02	-0.14	0.05	-0.09
3	6	-0.02	-0.04	-0.04	-0.08	0.05	0.02	-0.15	0.06	0.00
4	1	0.01	0.01	-0.38	-0.14	-0.06	-0.07	-0.06	0.23	0.11
5	6	0.00	-0.10	0.00	-0.07	0.00	-0.01	0.00	-0.12	0.00
6	1	0.00	-0.10	0.00	0.04	0.00	-0.12	0.00	-0.12	0.00
7	6	0.02	-0.04	0.04	-0.08	-0.05	0.02	0.15	0.06	0.00
8	8	-0.13	0.01	0.04	-0.07	-0.06	-0.13	-0.01	-0.05	-0.14
9	1	0.06	0.20	-0.34	0.08	0.04	-0.57	0.03	-0.07	-0.47
10	8	0.00	0.00	-0.01	-0.02	0.03	0.00	-0.10	-0.11	-0.04
11	6	-0.13	0.07	-0.02	0.13	0.08	-0.01	-0.08	-0.15	-0.02
12	8	-0.06	-0.01	-0.11	-0.06	-0.09	0.15	0.04	0.11	-0.11
13	6	0.06	-0.01	-0.34	0.14	-0.06	0.02	0.14	0.05	0.09
14	6	0.00	0.01	0.00	-0.02	0.00	-0.02	0.00	0.10	0.00
15	6	0.13	0.07	0.02	0.13	-0.08	-0.01	0.08	-0.15	0.02
16	8	0.00	0.00	0.01	-0.02	-0.03	0.00	0.10	-0.11	0.04
17	1	-0.01	0.01	0.38	-0.14	0.06	-0.07	0.06	0.23	-0.11
18	8	0.06	-0.01	0.11	-0.06	0.09	0.15	-0.04	0.11	0.11
19	8	0.13	0.01	-0.04	-0.07	0.06	-0.13	0.01	-0.05	0.14
20	1	-0.06	0.20	0.34	0.08	-0.04	-0.57	-0.03	-0.07	0.47

22 23 24
A A A
Frequencies -- 713.4706 755.3530 756.2336
Red. masses -- 3.2409 3.8886 5.7220
Frc consts -- 0.9720 1.3072 1.9280
IR Inten -- 17.7895 71.3189 38.6994
Raman Activ -- 0.1798 10.1870 17.9019
Depolar (P) -- 0.7500 0.7500 0.0177
Depolar (U) -- 0.8571 0.8571 0.0348

Atom	AN	X	Y	Z	X	Y	Z	X	Y	Z
1	6	-0.03	0.00	0.12	0.02	0.00	0.02	0.00	-0.16	0.00
2	6	0.01	-0.01	-0.16	0.00	-0.06	0.00	0.03	-0.05	-0.04
3	6	0.01	0.00	0.08	0.05	-0.14	-0.04	0.12	0.04	0.00
4	1	0.00	0.03	0.60	0.01	-0.18	0.35	0.04	-0.09	0.03
5	6	0.02	0.00	-0.18	-0.04	0.00	-0.06	0.00	0.32	0.00
6	1	0.00	0.00	0.25	-0.32	0.00	0.49	0.00	0.32	0.00

7	6	0.01	0.00	0.08	0.05	0.14	-0.04	-0.12	0.04	0.00
8	8	-0.03	-0.01	0.02	0.05	0.03	-0.06	-0.01	-0.08	-0.14
9	1	0.03	0.07	-0.05	-0.08	-0.17	-0.12	0.04	-0.08	-0.49
10	8	0.02	0.02	-0.07	-0.01	0.00	-0.01	0.13	0.07	0.06
11	6	0.05	-0.07	-0.03	-0.09	0.24	0.12	-0.13	-0.07	-0.02
12	8	0.01	0.02	0.03	0.03	-0.07	-0.02	-0.02	0.05	-0.13
13	6	0.01	0.01	-0.16	0.00	0.06	0.00	-0.03	-0.05	0.04
14	6	-0.12	0.00	0.21	-0.04	0.00	0.02	0.00	-0.15	0.00
15	6	0.05	0.07	-0.03	-0.09	-0.24	0.12	0.13	-0.07	0.02
16	8	0.02	-0.02	-0.07	-0.01	0.00	-0.01	-0.13	0.07	-0.06
17	1	0.00	-0.03	0.60	0.01	0.18	0.35	-0.04	-0.09	-0.03
18	8	0.01	-0.02	0.03	0.03	0.07	-0.02	0.02	0.05	0.13
19	8	-0.03	0.01	0.02	0.05	-0.03	-0.06	0.01	-0.08	0.14
20	1	0.03	-0.07	-0.05	-0.08	0.17	-0.12	-0.04	-0.09	0.49

25 26 27
A A A
Frequencies -- 765.7123 802.9427 836.1162
Red. masses -- 7.4742 1.9307 9.2668
Frc consts -- 2.5819 0.7334 3.8169
IR Inten -- 33.4063 23.1462 60.2118
Raman Activ -- 5.0691 1.5225 6.4126
Depolar (P) -- 0.3316 0.7500 0.1392
Depolar (U) -- 0.4980 0.8571 0.2444

Atom	AN	X	Y	Z	X	Y	Z	X	Y	Z
1	6	0.00	-0.02	0.00	0.02	0.00	0.06	0.00	-0.06	0.00
2	6	-0.06	0.01	-0.19	0.01	0.02	0.08	0.08	-0.09	-0.03
3	6	-0.03	-0.05	-0.02	-0.02	0.03	-0.07	0.24	-0.11	0.01
4	1	0.03	0.08	0.32	-0.02	0.05	0.40	0.12	-0.34	0.00
5	6	0.00	-0.18	0.00	0.01	0.00	-0.08	0.00	0.24	0.00
6	1	0.00	-0.18	0.00	0.05	0.00	0.77	0.00	0.23	0.00
7	6	0.03	-0.05	0.02	-0.02	-0.03	-0.07	-0.24	-0.11	-0.01
8	8	-0.05	-0.11	-0.01	0.00	-0.02	0.00	-0.01	-0.03	-0.01
9	1	0.18	0.24	0.01	0.02	0.03	0.05	0.05	0.04	-0.09
10	8	0.04	0.03	0.01	-0.02	0.01	0.04	-0.29	-0.06	-0.14
11	6	-0.17	0.33	0.20	0.01	-0.05	-0.04	-0.07	0.07	0.04
12	8	0.08	-0.07	-0.08	-0.02	0.01	0.00	-0.01	-0.02	-0.04
13	6	0.06	0.01	0.19	0.01	-0.02	0.08	-0.08	-0.09	0.03
14	6	0.00	-0.05	0.00	0.07	0.00	-0.15	0.00	0.43	0.00
15	6	0.17	0.33	-0.20	0.01	0.05	-0.04	0.07	0.07	-0.04
16	8	-0.04	0.03	-0.01	-0.02	-0.01	0.04	0.29	-0.06	0.14
17	1	-0.03	0.08	-0.32	-0.02	-0.05	0.40	-0.12	-0.34	0.00
18	8	-0.08	-0.07	0.08	-0.02	-0.01	0.00	0.01	-0.02	0.04
19	8	0.05	-0.11	0.01	0.00	0.02	0.00	0.01	-0.03	0.01
20	1	-0.18	0.24	-0.01	0.02	-0.03	0.05	-0.05	0.04	0.09

28 29 30
A A A
Frequencies -- 856.5647 912.1283 934.3767
Red. masses -- 9.0805 6.6700 1.4255
Frc consts -- 3.9253 3.2696 0.7332
IR Inten -- 9.5871 4.7119 0.2116
Raman Activ -- 0.9087 0.6796 0.3289
Depolar (P) -- 0.7500 0.7500 0.6311
Depolar (U) -- 0.8571 0.8571 0.7738

Atom	AN	X	Y	Z	X	Y	Z	X	Y	Z
1	6	0.00	0.00	0.44	0.33	0.00	0.02	0.00	0.00	0.00
2	6	-0.02	0.00	-0.31	0.26	0.06	-0.02	0.00	0.00	0.05
3	6	-0.01	0.01	0.13	-0.09	0.11	0.01	0.02	0.00	-0.12
4	1	0.00	0.03	-0.10	-0.19	-0.07	-0.04	0.01	0.03	0.69
5	6	0.01	0.00	-0.03	-0.03	0.00	-0.02	0.00	0.01	0.00
6	1	0.05	0.00	-0.49	0.36	0.00	0.09	0.00	0.01	0.00

7 6 -0.01 -0.01 0.13 -0.09 -0.11 0.01 -0.02 0.00 0.12
8 8 -0.01 0.05 0.04 -0.02 0.11 0.13 0.00 0.01 0.01
9 1 -0.05 -0.03 -0.05 -0.07 0.08 0.37 -0.01 0.00 -0.01
10 8 -0.06 0.01 0.11 -0.01 0.03 -0.03 0.00 0.00 0.00
11 6 -0.03 0.07 0.05 -0.13 0.08 -0.02 0.00 -0.01 -0.01
12 8 0.04 0.00 -0.01 -0.09 0.00 -0.14 -0.01 0.00 0.00
13 6 -0.02 0.00 -0.31 0.26 -0.06 -0.02 0.00 0.00 -0.05
14 6 0.20 0.00 -0.45 -0.07 0.00 0.07 0.00 0.00 0.00
15 6 -0.03 -0.07 0.05 -0.13 -0.08 -0.02 0.00 -0.01 0.01
16 8 -0.06 -0.01 0.11 -0.01 -0.03 -0.03 0.00 0.00 0.00
17 1 0.00 -0.03 -0.10 -0.19 0.07 -0.04 -0.01 0.03 -0.69
18 8 0.04 0.00 -0.01 -0.09 0.00 -0.14 0.01 0.00 0.00
19 8 -0.01 -0.05 0.04 -0.02 -0.11 0.13 0.00 0.01 -0.01
20 1 -0.05 0.03 -0.05 -0.07 -0.08 0.37 0.01 0.00 0.01
31 32 33
A A A
Frequencies -- 980.0617 1093.8620 1129.8806
Red. masses -- 1.3278 3.2547 9.2957
Frc consts -- 0.7514 2.2945 6.9920
IR Inten -- 1.2252 2.2527 59.2358
Raman Activ -- 0.0809 28.4781 1.6411
Depolar (P) -- 0.7500 0.0499 0.5155
Depolar (U) -- 0.8571 0.0951 0.6803
Atom AN X Y Z X Y Z X Y Z
1 6 -0.01 0.00 -0.02 0.00 0.18 0.00 0.00 -0.26 0.00
2 6 -0.01 0.00 -0.01 -0.03 0.06 0.00 0.29 0.13 0.04
3 6 0.00 0.00 0.08 0.25 -0.05 0.02 -0.03 0.11 -0.01
4 1 0.01 -0.03 -0.51 0.50 0.36 -0.04 0.04 0.21 0.03
5 6 0.01 0.00 -0.12 0.00 -0.13 0.00 0.00 -0.27 0.00
6 1 -0.02 0.00 0.67 0.00 -0.16 0.00 0.00 -0.28 0.00
7 6 0.00 0.00 0.08 -0.25 -0.05 -0.02 0.03 0.11 0.01
8 8 0.00 0.00 0.00 0.01 -0.01 -0.02 0.04 -0.12 -0.22
9 1 0.00 0.00 -0.01 0.01 0.01 0.02 0.09 0.07 0.24
10 8 0.00 0.00 0.00 0.04 -0.02 0.02 -0.08 0.03 -0.04
11 6 0.00 0.00 0.00 0.02 -0.02 -0.03 0.12 0.12 -0.19
12 8 0.00 0.00 0.00 0.00 0.00 0.00 -0.11 0.00 -0.09
13 6 -0.01 0.00 -0.01 0.03 0.06 0.00 -0.29 0.13 -0.04
14 6 0.00 0.00 0.01 0.00 -0.04 0.00 0.00 0.02 0.00
15 6 0.00 0.00 0.00 -0.02 -0.02 0.03 -0.12 0.12 0.19
16 8 0.00 0.00 0.00 -0.04 -0.02 -0.02 0.08 0.03 0.04
17 1 0.01 0.03 -0.51 -0.50 0.36 0.04 -0.04 0.21 -0.03
18 8 0.00 0.00 0.00 0.00 0.00 0.00 0.11 0.00 0.09
19 8 0.00 0.00 0.00 -0.01 -0.01 0.02 -0.04 -0.12 0.22
20 1 0.00 0.00 -0.01 -0.01 0.01 -0.02 -0.09 0.07 -0.24
34 35 36
A A A
Frequencies -- 1158.6981 1180.4602 1201.9547
Red. masses -- 1.8622 2.1585 1.6115
Frc consts -- 1.4730 1.7722 1.3717
IR Inten -- 197.7005 6.3102 247.7359
Raman Activ -- 5.4119 4.8932 0.4576
Depolar (P) -- 0.7500 0.3487 0.7500
Depolar (U) -- 0.8571 0.5171 0.8571
Atom AN X Y Z X Y Z X Y Z
1 6 0.06 0.00 0.01 0.00 0.22 0.00 -0.05 0.00 -0.01
2 6 -0.03 -0.07 -0.01 0.02 0.04 0.01 -0.03 0.01 0.01
3 6 -0.06 -0.01 0.00 0.00 -0.09 -0.01 -0.01 -0.06 0.00
4 1 -0.13 -0.12 -0.01 -0.28 -0.58 0.04 -0.19 -0.37 0.02
5 6 0.08 0.00 0.00 0.00 0.04 0.00 0.06 0.00 0.00
6 1 0.88 0.00 0.02 0.00 0.04 0.00 0.56 0.00 0.01

7	6	-0.06	0.01	0.00	0.00	-0.09	0.01	-0.01	0.06	0.00
8	8	0.01	-0.04	-0.09	0.00	-0.03	-0.06	0.01	0.03	0.08
9	1	0.03	0.06	0.20	0.02	0.06	0.19	-0.02	-0.12	-0.36
10	8	0.00	0.01	-0.01	0.04	-0.02	0.02	0.00	0.00	0.00
11	6	-0.06	-0.04	0.08	0.03	0.04	-0.04	0.05	0.03	-0.06
12	8	0.03	0.00	0.03	-0.03	0.00	-0.02	-0.02	0.00	-0.02
13	6	-0.03	0.07	-0.01	-0.02	0.04	-0.01	-0.03	-0.01	0.01
14	6	-0.01	0.00	0.01	0.00	-0.03	0.00	0.01	0.00	0.00
15	6	-0.06	0.04	0.08	-0.03	0.04	0.04	0.05	-0.03	-0.06
16	8	0.00	-0.01	-0.01	-0.04	-0.02	-0.02	0.00	0.00	0.00
17	1	-0.13	0.12	-0.01	0.28	-0.58	-0.04	-0.19	0.37	0.02
18	8	0.03	0.00	0.03	0.03	0.00	0.02	-0.02	0.00	-0.02
19	8	0.01	0.04	-0.09	0.00	-0.03	0.06	0.01	-0.03	0.08
20	1	0.03	-0.06	0.20	-0.02	0.06	-0.19	-0.02	0.12	-0.36

	37	38	39
	A	A	A
Frequencies --	1249.9782	1254.6831	1329.5143
Red. masses --	1.7376	1.5981	6.4143
Frc consts --	1.5996	1.4823	6.6802
IR Inten --	45.3489	71.9387	1.0246
Raman Activ --	14.6617	1.6171	0.1776
Depolar (P) --	0.1223	0.7500	0.7500
Depolar (U) --	0.2180	0.8571	0.8571

Atom	AN	X	Y	Z	X	Y	Z	X	Y	Z
1	6	0.00	0.03	0.00	0.06	0.00	0.00	0.36	0.00	0.00
2	6	0.12	0.07	-0.01	-0.02	-0.13	0.01	-0.16	0.23	0.00
3	6	0.01	0.00	0.00	-0.04	0.03	0.00	-0.14	-0.21	0.00
4	1	-0.10	-0.21	0.01	0.22	0.50	-0.01	0.17	0.35	-0.02
5	6	0.00	-0.07	0.00	-0.02	0.00	0.00	0.25	0.00	0.01
6	1	0.00	-0.08	0.00	0.21	0.00	0.02	-0.38	0.00	-0.02
7	6	-0.01	0.00	0.00	-0.04	-0.03	0.00	-0.14	0.21	0.00
8	8	0.03	0.00	0.07	0.03	0.00	0.04	0.01	-0.01	0.00
9	1	0.00	-0.20	-0.61	0.01	-0.12	-0.38	-0.01	-0.07	-0.16
10	8	0.00	0.00	0.00	0.00	0.00	0.00	-0.01	0.02	-0.01
11	6	-0.02	0.00	0.02	0.00	0.02	0.00	-0.01	-0.05	0.02
12	8	0.01	-0.01	0.02	-0.01	0.00	-0.02	0.01	0.01	0.00
13	6	-0.12	0.07	0.01	-0.02	0.13	0.01	-0.16	-0.23	0.00
14	6	0.00	-0.01	0.00	-0.01	0.00	0.01	0.00	0.00	0.02
15	6	0.02	0.00	-0.02	0.00	-0.02	0.00	-0.01	0.05	0.02
16	8	0.00	0.00	0.00	0.00	0.00	0.00	-0.01	-0.02	-0.01
17	1	0.10	-0.21	-0.01	0.22	-0.50	-0.01	0.17	-0.35	-0.02
18	8	-0.01	-0.01	-0.02	-0.01	0.00	-0.02	0.01	-0.01	0.00
19	8	-0.03	0.00	-0.07	0.03	0.00	0.04	0.01	0.01	0.00
20	1	0.00	-0.20	0.61	0.01	0.12	-0.38	-0.01	0.07	-0.16

	40	41	42
	A	A	A
Frequencies --	1376.2439	1379.5972	1407.9687
Red. masses --	2.2125	9.7059	3.6549
Frc consts --	2.4690	10.8840	4.2689
IR Inten --	99.8047	169.3887	198.6204
Raman Activ --	1.7676	15.0445	15.1656
Depolar (P) --	0.7500	0.1311	0.0622
Depolar (U) --	0.8571	0.2318	0.1172

Atom	AN	X	Y	Z	X	Y	Z	X	Y	Z
1	6	-0.01	0.00	0.00	0.00	-0.11	0.00	0.00	-0.01	0.00
2	6	-0.03	-0.02	0.01	0.11	0.04	0.00	0.08	0.09	-0.01
3	6	-0.03	-0.06	0.00	0.01	0.06	0.00	0.05	0.04	0.00
4	1	0.15	0.26	-0.01	-0.05	-0.06	0.01	-0.11	-0.26	0.01
5	6	-0.01	0.00	0.00	0.00	-0.08	0.00	0.00	-0.09	0.00
6	1	0.30	0.00	0.01	0.00	-0.08	0.00	0.00	-0.10	0.00

7	6	-0.03	0.06	0.00	-0.01	0.06	0.00	-0.05	0.04	0.00
8	8	-0.05	0.04	0.01	-0.03	0.02	0.01	-0.05	0.05	0.02
9	1	-0.03	0.17	0.53	0.00	0.14	0.35	-0.04	0.16	0.52
10	8	0.00	0.00	0.00	0.28	-0.19	0.14	-0.07	0.05	-0.03
11	6	0.10	0.12	-0.12	-0.08	-0.09	0.09	-0.12	-0.16	0.15
12	8	0.01	-0.02	0.04	-0.01	0.01	-0.04	-0.02	0.03	-0.05
13	6	-0.03	0.02	0.01	-0.11	0.04	0.00	-0.08	0.09	0.01
14	6	-0.01	0.00	-0.01	0.00	0.57	0.00	0.00	-0.14	0.00
15	6	0.10	-0.12	-0.12	0.08	-0.09	-0.09	0.12	-0.16	-0.15
16	8	0.00	0.00	0.00	-0.28	-0.19	-0.14	0.07	0.05	0.03
17	1	0.15	-0.26	-0.01	0.05	-0.06	-0.01	0.11	-0.26	-0.01
18	8	0.01	0.02	0.04	0.01	0.01	0.04	0.02	0.03	0.05
19	8	-0.05	-0.04	0.01	0.03	0.02	-0.01	0.05	0.05	-0.02
20	1	-0.03	-0.17	0.53	0.00	0.14	-0.35	0.04	0.16	-0.52

	43	44	45
	A	A	A
Frequencies --	1478.0701	1503.5741	1655.1204
Red. masses --	3.8986	2.7869	7.8144
Frc consts --	5.0182	3.7121	12.6126
IR Inten --	1.0955	19.4600	14.5200
Raman Activ --	1.8337	0.0011	11.7233
Depolar (P) --	0.3600	0.7500	0.7500
Depolar (U) --	0.5294	0.8571	0.8571

Atom	AN	X	Y	Z	X	Y	Z	X	Y	Z
1	6	0.00	0.24	0.00	0.11	0.00	0.00	-0.38	0.00	-0.01
2	6	0.23	-0.15	0.01	-0.09	-0.13	0.01	0.27	-0.06	0.00
3	6	-0.11	-0.05	0.00	-0.04	0.13	0.00	-0.24	-0.12	-0.01
4	1	0.22	0.55	-0.01	-0.31	-0.30	0.00	-0.01	0.32	-0.01
5	6	0.00	0.09	0.00	0.21	0.00	0.01	0.41	0.00	0.01
6	1	0.00	0.12	0.00	-0.67	0.00	-0.02	-0.43	0.00	-0.02
7	6	0.11	-0.05	0.00	-0.04	-0.13	0.00	-0.24	0.12	-0.01
8	8	-0.01	0.01	0.01	-0.01	0.01	0.01	0.00	0.00	-0.01
9	1	-0.02	0.01	0.08	0.00	0.03	0.09	0.01	0.01	-0.02
10	8	0.00	0.00	0.00	-0.01	0.01	0.00	0.03	-0.03	0.02
11	6	-0.04	0.00	0.05	0.04	0.05	-0.04	-0.01	-0.01	0.05
12	8	-0.02	0.00	-0.03	0.01	-0.01	0.02	-0.02	0.00	-0.03
13	6	-0.23	-0.15	-0.01	-0.09	0.13	0.01	0.27	0.06	0.00
14	6	0.00	-0.03	0.00	0.00	0.00	0.01	-0.06	0.00	-0.05
15	6	0.04	0.00	-0.05	0.04	-0.05	-0.04	-0.01	0.01	0.05
16	8	0.00	0.00	0.00	-0.01	-0.01	0.00	0.03	0.03	0.02
17	1	-0.22	0.55	0.01	-0.31	0.30	0.00	-0.01	-0.31	-0.01
18	8	0.02	0.00	0.03	0.01	0.01	0.02	-0.02	0.00	-0.03
19	8	0.01	0.01	-0.01	-0.01	-0.01	0.01	0.00	0.00	-0.01
20	1	0.02	0.01	-0.08	0.00	-0.03	0.09	0.01	-0.01	-0.02

	46	47	48
	A	A	A
Frequencies --	1659.3615	1746.9636	1826.4354
Red. masses --	6.1093	12.7077	10.3134
Frc consts --	9.9111	22.8499	20.2703
IR Inten --	2.9921	625.9244	833.2033
Raman Activ --	24.8404	5.7289	0.0626
Depolar (P) --	0.6920	0.7500	0.7497
Depolar (U) --	0.8180	0.8571	0.8569

Atom	AN	X	Y	Z	X	Y	Z	X	Y	Z
1	6	0.00	0.15	0.00	-0.10	0.00	-0.01	0.02	0.00	0.00
2	6	0.00	-0.29	0.01	0.05	0.00	0.00	-0.07	-0.02	-0.01
3	6	0.12	0.32	0.00	-0.03	-0.02	0.00	0.01	0.00	0.00
4	1	-0.29	-0.40	0.00	0.01	0.06	0.00	0.02	-0.01	-0.01
5	6	0.00	-0.17	0.00	0.04	0.00	0.00	-0.01	0.00	0.00
6	1	0.00	-0.20	0.00	-0.04	0.00	0.00	0.04	0.00	0.01

7	6	-0.12	0.32	0.00	-0.03	0.02	0.00	0.01	0.00	0.00
8	8	0.00	0.00	0.00	0.00	0.01	-0.01	-0.03	0.00	-0.04
9	1	0.00	-0.01	-0.02	-0.02	-0.01	0.06	0.02	0.12	0.31
10	8	0.00	0.00	0.00	-0.28	0.13	-0.13	0.00	0.00	0.00
11	6	0.01	0.04	-0.02	0.01	0.03	0.01	0.39	-0.01	0.35
12	8	0.00	-0.01	0.01	-0.01	-0.01	-0.01	-0.23	0.02	-0.23
13	6	0.00	-0.29	-0.01	0.05	0.00	0.00	-0.07	0.02	-0.01
14	6	0.00	-0.02	0.00	0.78	0.00	0.36	-0.02	0.00	-0.01
15	6	-0.01	0.04	0.02	0.01	-0.03	0.01	0.39	0.01	0.35
16	8	0.00	0.00	0.00	-0.28	-0.13	-0.13	0.00	0.00	0.00
17	1	0.29	-0.40	0.00	0.01	-0.06	0.00	0.02	0.01	-0.01
18	8	0.00	-0.01	-0.01	-0.01	0.01	-0.01	-0.23	-0.02	-0.23
19	8	0.00	0.00	0.00	0.00	-0.01	-0.01	-0.03	0.00	-0.04
20	1	0.00	-0.01	0.02	-0.02	0.01	0.06	0.02	-0.12	0.31

	49	50	51
	A	A	A
Frequencies --	1829.0666	3183.4546	3196.7958
Red. masses --	10.3439	1.0854	1.0915
Frc consts --	20.3888	6.4809	6.5723
IR Inten --	6.0133	1.1731	15.6303
Raman Activ --	68.4120	55.7302	77.8313
Depolar (P) --	0.1827	0.7084	0.7500
Depolar (U) --	0.3089	0.8293	0.8571

Atom	AN	X	Y	Z	X	Y	Z	X	Y	Z
1	6	0.00	0.01	0.00	0.00	0.00	0.00	0.00	0.00	0.00
2	6	0.07	0.02	0.02	0.00	0.00	0.00	0.00	0.00	0.00
3	6	-0.01	0.00	0.00	-0.04	0.02	0.00	-0.05	0.03	0.00
4	1	-0.02	0.00	0.01	0.43	-0.25	0.02	0.61	-0.35	0.02
5	6	0.00	-0.01	0.00	0.00	-0.06	0.00	0.00	0.00	0.00
6	1	0.00	-0.01	0.00	0.00	0.70	0.00	-0.01	0.00	0.00
7	6	0.01	0.00	0.00	0.04	0.02	0.00	-0.05	-0.03	0.00
8	8	-0.03	0.00	-0.04	0.00	0.00	0.00	0.00	0.00	0.00
9	1	0.01	0.13	0.31	0.00	0.00	0.00	0.00	0.00	0.00
10	8	0.00	0.00	0.00	0.00	0.00	0.00	0.00	0.00	0.00
11	6	-0.39	0.00	-0.34	0.00	0.00	0.00	0.00	0.00	0.00
12	8	0.23	-0.02	0.23	0.00	0.00	0.00	0.00	0.00	0.00
13	6	-0.07	0.02	-0.02	0.00	0.00	0.00	0.00	0.00	0.00
14	6	0.00	-0.01	0.00	0.00	0.00	0.00	0.00	0.00	0.00
15	6	0.39	0.00	0.34	0.00	0.00	0.00	0.00	0.00	0.00
16	8	0.00	0.00	0.00	0.00	0.00	0.00	0.00	0.00	0.00
17	1	0.02	0.00	-0.01	-0.43	-0.25	-0.02	0.61	0.35	0.02
18	8	-0.23	-0.02	-0.23	0.00	0.00	0.00	0.00	0.00	0.00
19	8	0.03	0.00	0.04	0.00	0.00	0.00	0.00	0.00	0.00
20	1	-0.01	0.13	-0.31	0.00	0.00	0.00	0.00	0.00	0.00

	52	53	54
	A	A	A
Frequencies --	3210.4055	3862.5827	3862.7678
Red. masses --	1.0979	1.0641	1.0641
Frc consts --	6.6668	9.3536	9.3547
IR Inten --	35.7688	82.8969	48.3145
Raman Activ --	240.5150	100.0759	204.3170
Depolar (P) --	0.1319	0.7493	0.1145
Depolar (U) --	0.2331	0.8567	0.2055

Atom	AN	X	Y	Z	X	Y	Z	X	Y	Z
1	6	0.00	0.00	0.00	0.00	0.00	0.00	0.00	0.00	0.00
2	6	0.00	0.00	0.00	0.00	0.00	0.00	0.00	0.00	0.00
3	6	0.04	-0.02	0.00	0.00	0.00	0.00	0.00	0.00	0.00
4	1	-0.43	0.24	-0.02	0.00	0.00	0.00	0.00	0.00	0.00
5	6	0.00	-0.06	0.00	0.00	0.00	0.00	0.00	0.00	0.00
6	1	0.00	0.71	0.00	0.00	0.00	0.00	0.00	0.00	0.00

7	6	-0.04	-0.02	0.00	0.00	0.00	0.00	0.00	0.00	0.00
8	8	0.00	0.00	0.00	0.04	-0.02	0.01	-0.03	0.02	-0.01
9	1	0.00	0.00	0.00	-0.59	0.40	-0.09	0.57	-0.39	0.09
10	8	0.00	0.00	0.00	0.00	0.00	0.00	0.00	0.00	0.00
11	6	0.00	0.00	0.00	0.00	0.00	0.00	0.00	0.00	0.00
12	8	0.00	0.00	0.00	0.00	0.00	0.00	0.00	0.00	0.00
13	6	0.00	0.00	0.00	0.00	0.00	0.00	0.00	0.00	0.00
14	6	0.00	0.00	0.00	0.00	0.00	0.00	0.00	0.00	0.00
15	6	0.00	0.00	0.00	0.00	0.00	0.00	0.00	0.00	0.00
16	8	0.00	0.00	0.00	0.00	0.00	0.00	0.00	0.00	0.00
17	1	0.43	0.24	0.02	0.00	0.00	0.00	0.00	0.00	0.00
18	8	0.00	0.00	0.00	0.00	0.00	0.00	0.00	0.00	0.00
19	8	0.00	0.00	0.00	0.03	0.02	0.01	0.04	0.02	0.01
20	1	0.00	0.00	0.00	-0.57	-0.39	-0.09	-0.59	-0.40	-0.09

HEM-xH20

Harmonic frequencies (cm**-1), IR intensities (KM/Mole), Raman scattering activities (A**4/AMU), depolarization ratios for plane and unpolarized incident light, reduced masses (AMU), force constants (mDyne/A), and normal coordinates:

	1	2	3
	A	A	A
Frequencies --	23.3572	44.0834	55.0620
Red. masses --	11.7221	6.2124	9.8660
Frc consts --	0.0038	0.0071	0.0176
IR Inten --	0.1256	6.4946	0.9012
Raman Activ --	0.4832	0.4008	0.8601
Depolar (P) --	0.6074	0.6776	0.7480
Depolar (U) --	0.7557	0.8078	0.8558

Atom	AN	X	Y	Z	X	Y	Z	X	Y	Z
1	6	-0.01	0.02	0.06	0.01	-0.04	0.04	0.01	0.03	-0.03
2	6	0.01	0.01	0.04	-0.01	-0.03	0.05	0.02	0.02	-0.04
3	6	-0.02	0.01	0.03	0.03	-0.03	-0.03	-0.01	0.02	-0.16
4	6	0.00	0.04	0.06	0.01	-0.04	0.07	0.01	0.06	0.15
5	6	0.00	0.00	-0.01	-0.01	0.00	-0.01	0.02	0.01	-0.07
6	6	0.03	0.02	0.08	-0.06	-0.05	0.12	-0.01	0.01	-0.08
7	6	-0.02	0.01	-0.01	0.03	0.00	-0.09	-0.01	0.01	-0.27
8	1	-0.02	0.02	0.04	0.04	-0.04	-0.04	-0.01	0.03	-0.17
9	8	-0.01	0.17	0.14	0.01	-0.02	0.08	0.11	0.23	0.37
10	8	0.00	-0.12	-0.08	0.02	-0.05	0.05	-0.11	-0.13	0.05
11	6	-0.01	0.00	-0.03	0.01	0.01	-0.09	0.00	0.00	-0.19
12	6	0.00	0.01	-0.06	-0.03	0.02	-0.02	0.05	-0.03	0.11
13	8	0.28	0.14	0.30	-0.17	-0.02	0.10	0.01	-0.01	-0.09
14	8	-0.19	-0.10	-0.13	-0.02	-0.11	0.17	-0.07	-0.01	-0.14
15	1	-0.03	0.00	-0.03	0.05	0.01	-0.15	-0.02	0.01	-0.38
16	1	-0.02	-0.11	-0.16	0.04	0.01	0.03	-0.08	-0.09	0.06
17	1	-0.01	-0.02	-0.08	0.01	0.03	-0.14	0.00	0.00	-0.22
18	8	-0.18	0.14	-0.31	-0.04	0.04	-0.03	0.20	-0.07	0.32
19	8	0.26	-0.18	0.21	-0.05	0.02	-0.03	-0.10	-0.02	0.05
20	1	0.19	-0.14	0.18	-0.07	0.03	-0.04	-0.02	-0.06	0.19
21	8	-0.14	-0.12	-0.23	0.22	0.24	-0.32	-0.09	-0.08	-0.08
22	1	-0.15	-0.09	-0.21	0.08	0.14	-0.09	-0.07	-0.03	-0.11
23	1	-0.17	-0.07	-0.19	0.59	-0.02	-0.44	-0.14	-0.12	-0.13

	4	5	6
	A	A	A
Frequencies --	81.0612	111.9226	135.3788
Red. masses --	10.2675	9.0248	8.3322
Frc consts --	0.0398	0.0666	0.0900
IR Inten --	3.3734	1.2731	5.5422

Raman Activ -- 0.3214 1.3653 1.5253
Depolar (P) -- 0.7324 0.5759 0.7500
Depolar (U) -- 0.8455 0.7309 0.8571

Atom	AN	X	Y	Z	X	Y	Z	X	Y	Z
1	6	0.01	0.06	0.02	0.00	-0.04	-0.04	-0.03	0.03	0.20
2	6	0.03	0.05	0.05	0.03	-0.06	-0.04	-0.04	0.03	0.23
3	6	-0.02	0.05	0.02	-0.01	-0.04	0.00	-0.04	0.03	-0.01
4	6	0.01	0.09	-0.06	0.01	0.12	-0.06	-0.03	-0.02	0.05
5	6	0.03	0.03	0.02	0.02	-0.06	0.00	-0.04	0.02	0.08
6	6	-0.08	0.01	0.15	0.03	-0.07	-0.01	0.04	0.07	0.11
7	6	-0.02	0.02	0.05	-0.01	-0.05	0.04	-0.05	0.02	-0.21
8	1	-0.04	0.07	0.01	-0.01	-0.04	-0.01	-0.04	0.03	-0.07
9	8	-0.05	0.19	-0.05	-0.06	0.40	0.08	-0.14	-0.06	-0.07
10	8	0.08	-0.01	-0.18	0.11	-0.01	-0.20	0.12	0.02	0.03
11	6	-0.01	0.00	0.03	0.01	-0.06	0.04	-0.05	0.03	-0.16
12	6	0.11	-0.04	-0.06	-0.09	0.03	0.00	-0.02	0.00	0.02
13	8	-0.18	0.14	0.23	-0.11	-0.09	-0.09	0.06	-0.13	-0.06
14	8	-0.12	-0.20	0.15	0.15	-0.06	0.11	0.10	0.34	0.13
15	1	-0.04	0.01	0.06	-0.02	-0.05	0.08	-0.05	0.03	-0.44
16	1	0.06	0.01	-0.25	0.20	0.16	-0.23	0.11	0.07	-0.06
17	1	-0.01	-0.01	0.02	0.01	-0.08	0.08	-0.06	0.04	-0.35
18	8	0.20	-0.28	0.03	-0.15	0.21	-0.05	-0.04	-0.05	-0.02
19	8	0.10	0.18	-0.29	-0.18	-0.07	0.07	0.04	0.05	0.00
20	1	0.22	0.08	-0.37	-0.33	0.04	0.06	0.07	0.02	-0.06
21	8	-0.07	-0.22	-0.01	0.21	-0.19	0.10	0.05	-0.31	-0.15
22	1	-0.12	-0.22	0.08	0.19	-0.19	0.15	0.01	-0.08	-0.07
23	1	-0.04	-0.06	0.12	0.18	0.02	0.26	-0.04	-0.26	-0.13

7 8 9
A A A
Frequencies -- 158.1831 164.1456 169.2964
Red. masses -- 5.5672 7.6690 7.4004
Frc consts -- 0.0821 0.1217 0.1250
IR Inten -- 3.9327 5.6761 2.8746
Raman Activ -- 1.4863 2.6172 1.0911
Depolar (P) -- 0.7467 0.7500 0.6908
Depolar (U) -- 0.8550 0.8571 0.8171

Atom	AN	X	Y	Z	X	Y	Z	X	Y	Z
1	6	0.02	-0.06	-0.04	-0.01	-0.02	0.09	0.06	-0.04	0.19
2	6	0.02	-0.07	0.14	0.01	-0.03	-0.05	0.05	-0.04	0.12
3	6	0.02	-0.05	-0.23	-0.02	-0.02	0.19	0.06	-0.04	0.16
4	6	0.02	0.00	-0.01	-0.01	-0.02	0.04	0.07	0.06	0.05
5	6	0.02	-0.05	0.22	0.00	-0.04	-0.16	0.06	-0.02	-0.04
6	6	0.05	-0.06	0.06	0.11	0.02	0.03	-0.06	-0.09	0.00
7	6	0.03	-0.04	-0.11	-0.02	-0.04	0.03	0.06	-0.02	-0.06
8	1	0.03	-0.07	-0.45	-0.02	-0.02	0.34	0.07	-0.04	0.22
9	8	0.02	0.07	0.04	-0.07	-0.05	-0.04	-0.11	0.09	-0.09
10	8	0.02	0.01	0.00	0.06	0.01	0.02	0.28	0.12	0.00
11	6	0.04	-0.04	0.15	-0.01	-0.04	-0.17	0.05	-0.02	-0.18
12	6	-0.05	0.02	0.04	-0.08	0.01	-0.08	0.04	-0.01	-0.01
13	8	0.17	-0.12	0.05	0.30	0.13	0.21	-0.11	-0.17	-0.09
14	8	-0.04	0.00	-0.05	0.02	-0.03	-0.05	-0.12	-0.08	-0.06
15	1	0.04	-0.03	-0.24	-0.04	-0.04	0.07	0.06	-0.02	-0.15
16	1	-0.01	-0.04	-0.01	0.06	0.03	-0.02	0.11	-0.07	-0.17
17	1	0.04	-0.04	0.22	-0.01	-0.05	-0.28	0.05	-0.01	-0.37
18	8	-0.11	-0.03	-0.06	0.05	0.07	0.11	0.04	0.06	0.00
19	8	-0.08	0.19	-0.14	-0.35	0.01	-0.19	0.00	-0.07	0.04
20	1	-0.22	0.26	-0.40	-0.50	0.13	-0.16	0.00	-0.06	0.10
21	8	-0.09	0.11	0.04	0.05	-0.02	0.01	-0.26	0.18	0.05
22	1	-0.06	0.07	-0.03	0.08	-0.01	-0.03	-0.23	0.09	-0.03
23	1	-0.13	0.13	0.05	-0.04	0.08	0.06	-0.30	0.29	0.12

	10	11	12
	A	A	A
Frequencies --	218.7295	235.3750	273.1174
Red. masses --	5.9932	6.2415	5.9586
Frc consts --	0.1689	0.2037	0.2619
IR Inten --	11.3615	37.2179	0.3104
Raman Activ --	1.0542	0.8971	0.4350
Depolar (P) --	0.7361	0.7479	0.6699
Depolar (U) --	0.8480	0.8558	0.8023

Atom	AN	X	Y	Z	X	Y	Z	X	Y	Z
1	6	0.04	0.03	-0.09	0.03	-0.01	-0.06	-0.02	-0.19	-0.13
2	6	0.07	0.01	-0.05	-0.01	0.01	-0.03	-0.16	-0.08	-0.15
3	6	-0.03	0.00	-0.08	0.03	-0.02	0.01	0.16	-0.13	-0.04
4	6	0.07	0.11	0.02	0.04	-0.07	-0.06	-0.01	-0.06	-0.01
5	6	0.06	-0.08	-0.01	0.00	-0.02	-0.05	-0.16	0.07	-0.12
6	6	0.03	0.02	0.03	0.05	0.08	0.09	-0.08	-0.03	-0.02
7	6	-0.04	-0.08	0.05	0.03	-0.03	0.06	0.19	0.05	0.06
8	1	-0.09	0.04	-0.10	0.03	-0.01	0.03	0.28	-0.22	0.00
9	8	0.12	-0.01	-0.01	0.05	0.05	0.03	-0.08	0.12	0.06
10	8	0.04	0.29	0.21	-0.01	-0.20	-0.16	0.11	0.13	0.10
11	6	0.01	-0.11	0.09	0.01	-0.02	-0.01	0.02	0.16	0.00
12	6	0.02	-0.05	-0.02	-0.01	-0.04	-0.03	-0.12	0.02	-0.03
13	8	-0.07	0.05	0.01	0.00	0.03	0.02	0.07	-0.01	0.06
14	8	0.06	-0.02	0.07	0.21	0.19	0.22	0.01	0.08	0.04
15	1	-0.08	-0.10	0.13	0.03	-0.03	0.13	0.33	0.11	0.18
16	1	-0.02	0.18	0.27	-0.23	-0.53	-0.25	0.13	0.17	0.12
17	1	0.01	-0.16	0.20	0.01	0.00	0.00	0.03	0.32	0.06
18	8	-0.01	0.05	-0.04	0.01	-0.01	0.00	-0.05	-0.17	0.05
19	8	-0.10	-0.09	-0.03	-0.06	-0.08	-0.01	0.03	0.01	0.02
20	1	-0.25	0.02	-0.05	-0.10	-0.04	0.02	0.30	-0.16	0.17
21	8	-0.18	-0.14	-0.17	-0.29	0.15	-0.03	-0.02	-0.03	-0.03
22	1	-0.24	0.02	-0.07	-0.42	0.01	0.12	-0.05	0.04	0.03
23	1	-0.08	-0.39	-0.36	0.07	-0.05	-0.10	-0.03	-0.01	-0.02

	13	14	15
	A	A	A
Frequencies --	292.7045	338.9078	345.8926
Red. masses --	6.3701	3.3129	2.2297
Frc consts --	0.3216	0.2242	0.1572
IR Inten --	2.5740	43.5063	37.5194
Raman Activ --	1.6770	0.5800	2.1074
Depolar (P) --	0.5825	0.6513	0.0820
Depolar (U) --	0.7362	0.7888	0.1516

Atom	AN	X	Y	Z	X	Y	Z	X	Y	Z
1	6	0.08	0.02	0.03	0.00	-0.01	0.08	0.02	0.02	-0.05
2	6	-0.02	0.08	0.03	0.00	0.01	-0.11	0.00	0.01	0.04
3	6	0.04	0.00	-0.02	-0.02	-0.01	0.01	0.01	0.01	0.11
4	6	0.14	-0.03	0.02	0.00	-0.03	0.08	0.05	0.04	-0.08
5	6	-0.05	-0.06	0.07	-0.01	-0.01	0.07	-0.02	-0.01	0.05
6	6	-0.09	0.16	-0.04	0.04	0.04	-0.12	-0.01	-0.01	0.02
7	6	0.02	-0.06	-0.05	-0.02	-0.01	-0.11	0.01	0.00	0.01
8	1	-0.02	0.04	-0.06	-0.03	-0.01	0.01	0.00	0.02	0.22
9	8	0.18	-0.04	0.04	-0.12	-0.01	0.00	0.13	0.03	-0.02
10	8	0.21	-0.06	-0.03	0.12	-0.03	0.04	-0.05	0.04	-0.04
11	6	-0.03	-0.05	0.04	-0.02	-0.02	0.05	-0.01	0.01	-0.11
12	6	-0.07	-0.12	0.02	-0.02	0.01	0.06	-0.04	-0.02	0.04
13	8	-0.13	0.22	0.00	-0.01	0.17	-0.04	0.03	-0.04	0.00
14	8	-0.13	0.15	-0.08	0.11	-0.10	-0.04	-0.06	0.01	-0.03
15	1	0.01	-0.06	-0.11	-0.02	-0.01	-0.25	0.01	0.00	0.02
16	1	0.25	0.05	-0.12	-0.06	-0.26	-0.06	-0.04	-0.01	0.10
17	1	-0.02	0.01	0.05	-0.02	-0.03	0.11	-0.01	0.04	-0.26

18	8	-0.12	-0.16	-0.05	-0.06	-0.03	0.00	-0.08	-0.08	0.00
19	8	-0.11	-0.12	0.01	0.03	0.07	0.03	-0.01	0.02	0.02
20	1	-0.23	-0.04	-0.05	0.01	0.07	-0.08	-0.04	0.03	-0.05
21	8	0.09	0.02	0.03	-0.05	0.02	0.03	0.00	0.01	0.05
22	1	0.19	0.08	-0.11	-0.19	-0.17	0.24	-0.10	-0.06	0.23
23	1	-0.30	0.46	0.30	0.54	-0.45	-0.21	0.52	-0.59	-0.31

	16	17	18
	A	A	A
Frequencies --	358.3918	386.3949	414.4478
Red. masses --	2.8667	5.7351	6.0334
Frc consts --	0.2169	0.5045	0.6106
IR Inten --	56.3762	13.7352	11.1621
Raman Activ --	0.5343	1.6719	6.6212
Depolar (P) --	0.7385	0.5756	0.1056
Depolar (U) --	0.8496	0.7306	0.1910

Atom	AN	X	Y	Z	X	Y	Z	X	Y	Z
1	6	-0.01	-0.01	-0.06	0.00	0.06	0.04	-0.02	0.16	0.10
2	6	0.00	-0.02	-0.08	0.07	0.02	0.06	-0.01	0.08	0.02
3	6	-0.05	-0.02	0.13	0.19	0.11	0.06	-0.05	0.19	-0.08
4	6	0.02	0.01	-0.03	-0.11	0.04	-0.01	0.05	0.09	0.08
5	6	-0.02	0.00	0.06	0.07	-0.05	0.11	-0.08	0.11	-0.09
6	6	0.04	-0.03	-0.03	0.09	0.03	-0.02	0.07	-0.08	0.01
7	6	-0.05	-0.02	-0.04	0.21	0.08	-0.04	-0.06	0.20	0.01
8	1	-0.06	-0.01	0.30	0.27	0.05	0.07	-0.06	0.20	-0.21
9	8	0.10	-0.02	0.02	-0.15	0.04	-0.05	0.11	-0.07	0.00
10	8	-0.01	0.02	-0.01	-0.22	0.02	0.01	0.10	-0.01	-0.02
11	6	-0.05	-0.02	-0.10	0.22	0.04	-0.03	-0.04	0.16	0.08
12	6	-0.05	0.03	0.09	-0.02	-0.10	0.04	-0.12	0.00	-0.07
13	8	0.00	0.01	-0.01	-0.05	0.08	-0.03	0.10	-0.15	-0.05
14	8	0.12	-0.08	0.05	0.08	-0.08	-0.01	0.08	-0.18	0.04
15	1	-0.05	-0.02	-0.06	0.15	0.06	-0.13	-0.08	0.19	0.03
16	1	0.03	0.09	-0.03	-0.12	0.12	0.13	-0.06	-0.17	-0.22
17	1	-0.05	-0.04	-0.21	0.22	0.15	-0.13	-0.04	0.17	0.20
18	8	-0.09	-0.03	0.02	-0.09	-0.16	-0.06	-0.07	-0.17	-0.01
19	8	0.06	0.13	0.04	-0.09	-0.11	0.02	-0.10	-0.10	0.02
20	1	0.09	0.08	-0.12	-0.35	0.07	-0.10	0.08	-0.18	0.32
21	8	-0.03	-0.01	-0.07	-0.01	-0.01	-0.03	-0.02	-0.01	-0.03
22	1	0.06	0.08	-0.22	0.11	0.13	-0.21	-0.10	-0.21	0.07
23	1	-0.52	0.53	0.25	-0.39	0.11	-0.02	0.15	0.23	0.21

	19	20	21
	A	A	A
Frequencies --	475.2256	524.6552	537.6940
Red. masses --	1.1465	1.4204	3.2047
Frc consts --	0.1525	0.2304	0.5459
IR Inten --	38.8947	74.0798	42.2589
Raman Activ --	1.2640	0.9396	0.3841
Depolar (P) --	0.5632	0.7306	0.6575
Depolar (U) --	0.7206	0.8443	0.7933

Atom	AN	X	Y	Z	X	Y	Z	X	Y	Z
1	6	0.01	0.00	0.03	0.01	-0.01	0.06	-0.02	-0.05	0.08
2	6	0.00	0.00	0.00	0.01	-0.01	0.05	-0.07	-0.03	-0.06
3	6	-0.02	0.00	-0.01	0.02	-0.02	0.02	0.00	-0.06	0.15
4	6	0.02	0.00	0.00	0.00	0.02	-0.02	0.00	0.01	-0.05
5	6	-0.01	0.01	-0.02	0.00	-0.04	0.01	-0.04	0.06	0.08
6	6	0.02	-0.01	0.01	0.02	0.02	-0.01	-0.06	-0.05	-0.04
7	6	-0.02	0.01	0.00	0.02	-0.04	-0.02	-0.01	0.05	-0.19
8	1	-0.02	0.01	-0.03	0.01	-0.02	-0.04	0.04	-0.09	0.19
9	8	0.00	0.01	-0.01	0.03	0.00	-0.01	0.08	0.00	0.00
10	8	0.02	-0.02	-0.02	-0.05	0.01	-0.02	-0.05	0.02	-0.06
11	6	-0.02	0.01	0.01	0.03	-0.04	0.03	-0.06	0.08	0.17

12	6	-0.02	0.01	-0.01	-0.06	0.01	-0.04	0.07	0.00	-0.05
13	8	-0.01	0.00	0.01	-0.01	0.04	0.00	0.03	-0.01	0.02
14	8	0.02	-0.02	0.01	0.01	0.00	-0.01	0.02	0.01	0.03
15	1	-0.01	0.01	-0.01	0.01	-0.04	-0.11	0.07	0.09	-0.51
16	1	0.05	-0.03	0.08	0.07	0.16	0.05	0.27	0.46	0.12
17	1	-0.02	0.00	0.04	0.03	-0.03	0.00	-0.05	0.10	0.22
18	8	-0.01	-0.01	0.00	-0.02	0.03	0.02	0.09	0.03	-0.03
19	8	-0.01	0.00	0.00	-0.03	0.02	-0.06	-0.02	-0.10	-0.02
20	1	0.09	-0.05	0.08	0.69	-0.42	0.48	-0.28	0.10	-0.04
21	8	0.01	0.02	0.05	-0.01	0.00	-0.01	-0.03	0.00	-0.01
22	1	0.24	0.36	-0.31	-0.06	-0.01	0.08	-0.10	0.14	0.13
23	1	-0.50	-0.45	-0.47	0.08	0.05	0.06	0.02	-0.02	-0.01

	22	23	24
	A	A	A
Frequencies --	575.3289	593.2736	621.9502
Red. masses --	1.3997	4.7210	4.1030
Frc consts --	0.2730	0.9790	0.9351
IR Inten --	137.4525	7.2780	21.8910
Raman Activ --	2.7752	0.6239	0.7958
Depolar (P) --	0.5379	0.7375	0.7500
Depolar (U) --	0.6995	0.8489	0.8571

Atom	AN	X	Y	Z	X	Y	Z	X	Y	Z
1	6	-0.02	-0.01	0.05	-0.01	-0.11	0.01	0.06	-0.05	0.28
2	6	0.01	-0.01	0.03	-0.06	-0.02	0.26	-0.01	-0.03	0.05
3	6	-0.01	0.00	-0.07	-0.04	-0.14	-0.07	0.03	-0.12	-0.03
4	6	-0.03	0.00	0.02	0.01	-0.07	-0.09	0.07	0.13	-0.01
5	6	0.00	0.00	-0.05	-0.07	0.09	0.18	-0.04	0.01	-0.23
6	6	0.03	0.00	-0.01	0.06	0.03	-0.08	-0.01	0.00	-0.02
7	6	0.00	0.00	0.07	-0.02	0.03	0.15	0.04	-0.10	0.00
8	1	0.00	0.00	-0.16	0.00	-0.17	-0.36	0.01	-0.11	-0.32
9	8	0.00	-0.03	0.03	0.04	0.03	0.01	0.08	0.04	-0.09
10	8	0.01	-0.03	-0.05	-0.01	0.04	0.03	-0.13	0.04	-0.02
11	6	0.01	-0.01	-0.04	-0.15	0.12	-0.09	-0.03	-0.02	0.04
12	6	-0.02	0.00	0.01	0.06	0.02	-0.06	-0.09	0.05	-0.04
13	8	-0.01	0.02	-0.01	-0.01	0.12	-0.05	-0.02	0.03	0.00
14	8	0.02	-0.02	-0.01	0.10	-0.10	-0.04	0.02	0.01	-0.01
15	1	0.00	-0.01	0.14	0.14	0.11	0.03	0.11	-0.07	-0.06
16	1	0.50	0.70	0.12	-0.17	-0.23	0.03	-0.35	-0.34	0.01
17	1	0.01	-0.01	-0.05	-0.16	0.17	-0.55	-0.02	0.01	0.22
18	8	-0.01	-0.01	0.01	0.09	0.02	-0.03	-0.03	-0.04	0.06
19	8	0.02	0.02	0.03	-0.03	-0.08	-0.02	0.07	0.05	0.07
20	1	-0.13	0.10	-0.15	-0.13	0.02	0.13	-0.29	0.24	-0.37
21	8	-0.04	0.01	0.00	0.00	0.00	-0.01	0.02	-0.01	-0.01
22	1	-0.15	0.20	0.21	0.03	-0.09	-0.07	0.05	-0.14	-0.09
23	1	0.07	-0.05	-0.03	-0.02	0.06	0.04	0.00	0.09	0.07

	25	26	27
	A	A	A
Frequencies --	670.7300	697.3731	725.5748
Red. masses --	4.3485	5.4917	5.9700
Frc consts --	1.1526	1.5736	1.8518
IR Inten --	119.3275	67.6311	3.6037
Raman Activ --	3.0987	2.1780	0.3877
Depolar (P) --	0.1638	0.5100	0.4055
Depolar (U) --	0.2815	0.6755	0.5770

Atom	AN	X	Y	Z	X	Y	Z	X	Y	Z
1	6	-0.08	0.05	0.02	0.19	0.08	-0.05	-0.04	-0.02	-0.15
2	6	-0.14	0.04	0.03	-0.01	0.22	-0.01	0.04	0.01	0.25
3	6	0.09	0.13	0.00	0.10	0.01	-0.01	-0.02	0.01	0.12
4	6	-0.05	-0.01	-0.02	0.15	-0.06	0.00	-0.06	-0.22	-0.17
5	6	-0.15	-0.11	0.03	-0.09	0.07	0.00	-0.01	0.00	-0.18

6	6	-0.03	0.03	-0.03	-0.02	0.12	0.03	0.23	0.12	0.16
7	6	0.07	-0.01	0.01	0.05	-0.20	-0.02	-0.03	0.01	-0.18
8	1	0.10	0.11	-0.08	-0.12	0.18	0.15	-0.02	0.01	0.44
9	8	0.05	-0.02	0.05	-0.10	0.11	-0.12	-0.01	0.03	0.08
10	8	0.04	0.05	-0.04	-0.07	-0.12	0.12	0.05	0.05	0.03
11	6	0.02	-0.01	0.01	-0.22	-0.01	0.01	0.01	-0.02	0.11
12	6	-0.09	-0.18	0.00	0.02	-0.03	0.03	-0.12	0.09	-0.13
13	8	0.05	0.00	-0.04	0.10	-0.06	-0.10	-0.06	0.03	-0.07
14	8	0.01	-0.07	0.04	-0.04	-0.12	0.08	-0.01	-0.06	-0.08
15	1	-0.03	-0.05	-0.08	0.15	-0.15	0.08	-0.05	0.00	0.09
16	1	-0.10	-0.11	-0.15	0.26	0.21	0.48	0.03	0.00	0.08
17	1	0.03	0.18	-0.09	-0.21	0.12	0.08	0.02	-0.08	0.53
18	8	-0.03	0.18	0.15	-0.01	0.02	-0.01	0.01	-0.03	0.05
19	8	0.15	-0.08	-0.13	-0.01	0.00	-0.01	0.05	0.01	0.05
20	1	0.10	-0.15	-0.75	0.02	-0.02	-0.01	-0.14	0.11	-0.14
21	8	0.01	0.00	0.01	0.00	-0.01	-0.02	-0.01	0.00	0.00
22	1	0.04	0.00	-0.05	-0.11	0.17	0.17	0.00	-0.08	-0.02
23	1	-0.05	-0.06	-0.06	-0.02	0.15	0.11	0.05	0.03	0.03

	28	29	30
	A	A	A
Frequencies --	762.6021	771.5174	788.0806
Red. masses --	2.3968	4.7758	5.5225
Frc consts --	0.8213	1.6749	2.0208
IR Inten --	53.6681	22.5366	14.3634
Raman Activ --	2.9389	15.9243	1.6622
Depolar (P) --	0.3966	0.0327	0.3062
Depolar (U) --	0.5680	0.0633	0.4689

Atom	AN	X	Y	Z	X	Y	Z	X	Y	Z
1	6	0.02	-0.05	-0.04	0.05	-0.02	0.04	-0.05	-0.02	0.22
2	6	0.01	-0.03	0.05	0.08	-0.07	-0.03	-0.04	0.03	0.01
3	6	-0.02	-0.11	-0.04	-0.10	0.02	0.01	0.00	0.02	-0.02
4	6	0.02	0.15	0.12	0.17	-0.13	-0.11	-0.06	-0.22	-0.21
5	6	-0.01	0.03	0.00	0.05	-0.01	-0.03	0.03	0.01	-0.14
6	6	0.08	0.00	0.04	0.02	-0.11	-0.01	-0.08	0.04	-0.05
7	6	0.00	0.05	-0.08	-0.12	0.23	0.04	-0.02	0.05	-0.04
8	1	0.04	-0.14	0.44	-0.05	-0.01	-0.20	0.06	-0.02	-0.09
9	8	-0.01	-0.03	-0.04	-0.01	0.13	-0.12	0.05	0.03	0.08
10	8	-0.02	-0.03	-0.02	-0.02	-0.11	0.15	0.00	0.10	-0.02
11	6	-0.06	0.09	-0.04	0.06	0.06	0.01	0.01	0.01	-0.07
12	6	0.03	-0.10	0.09	-0.07	-0.09	0.04	0.22	-0.15	0.27
13	8	-0.03	0.01	0.00	-0.06	0.00	0.08	0.06	0.01	-0.04
14	8	0.01	0.01	-0.04	0.03	0.07	-0.07	-0.01	-0.04	0.06
15	1	0.18	0.11	0.60	-0.15	0.23	-0.18	-0.03	0.04	0.43
16	1	-0.01	0.03	-0.10	0.17	-0.01	0.53	-0.08	-0.09	0.10
17	1	-0.04	0.08	0.44	0.04	-0.09	-0.08	0.02	-0.08	0.56
18	8	-0.04	0.04	0.01	-0.07	0.04	0.08	-0.04	0.01	-0.11
19	8	0.00	-0.03	-0.06	0.05	-0.06	-0.08	-0.09	0.07	-0.02
20	1	0.11	-0.12	-0.13	0.13	-0.16	-0.35	0.17	-0.09	0.17
21	8	0.00	0.00	0.00	-0.01	0.00	-0.02	0.00	0.00	0.00
22	1	0.01	-0.03	-0.02	-0.08	0.07	0.11	0.02	-0.03	-0.02
23	1	0.02	-0.02	-0.01	0.04	0.14	0.11	-0.03	0.02	0.00

	31	32	33
	A	A	A
Frequencies --	809.1850	838.5643	858.5974
Red. masses --	2.7195	8.2293	6.5579
Frc consts --	1.0492	3.4095	2.8484
IR Inten --	22.1258	53.8884	4.3495
Raman Activ --	1.4374	6.1586	1.1012
Depolar (P) --	0.7147	0.1361	0.7461
Depolar (U) --	0.8336	0.2396	0.8546

Atom	AN	X	Y	Z	X	Y	Z	X	Y	Z
1	6	0.00	-0.03	-0.02	-0.03	-0.11	-0.05	0.02	0.02	-0.28
2	6	0.03	0.02	-0.10	0.02	-0.04	0.02	-0.01	-0.01	0.35
3	6	-0.01	-0.08	0.05	-0.16	-0.19	0.01	0.02	0.02	0.11
4	6	-0.03	0.02	0.02	0.05	0.08	0.06	0.01	0.10	0.10
5	6	0.00	0.03	-0.09	0.10	-0.05	0.01	0.02	0.02	-0.29
6	6	0.17	0.09	0.17	-0.17	0.35	0.00	-0.19	-0.11	-0.22
7	6	0.01	0.02	0.09	-0.09	0.21	0.01	-0.01	-0.01	0.01
8	1	0.02	-0.11	-0.39	0.04	-0.35	-0.01	-0.01	0.03	-0.14
9	8	-0.01	-0.03	0.02	0.01	0.00	-0.04	-0.03	-0.02	-0.02
10	8	0.00	0.02	-0.03	0.00	-0.04	0.01	0.02	-0.05	0.01
11	6	-0.06	0.05	0.06	0.25	0.00	0.00	0.01	0.00	0.12
12	6	0.06	-0.08	0.11	-0.07	0.02	-0.06	0.09	-0.08	0.15
13	8	-0.03	-0.03	-0.04	0.19	0.04	-0.24	0.04	0.06	0.04
14	8	-0.05	-0.01	-0.03	-0.16	-0.13	0.24	0.08	0.00	0.03
15	1	0.12	0.09	-0.71	-0.09	0.21	-0.03	-0.04	0.00	-0.64
16	1	-0.02	0.03	-0.10	-0.01	-0.07	0.01	0.02	0.02	-0.11
17	1	-0.07	0.08	-0.33	0.23	-0.25	-0.03	0.00	0.00	-0.17
18	8	-0.04	0.02	-0.02	-0.01	-0.02	0.03	-0.04	0.00	-0.04
19	8	-0.02	0.01	-0.03	0.02	-0.02	0.01	-0.03	0.05	0.00
20	1	0.08	-0.06	-0.05	-0.02	-0.01	-0.11	0.07	-0.03	-0.03
21	8	0.00	0.00	0.01	0.03	-0.01	0.00	0.00	0.00	0.00
22	1	0.04	-0.10	-0.06	-0.07	0.25	0.16	-0.04	0.13	0.07
23	1	0.03	-0.02	0.00	-0.10	0.02	-0.01	-0.04	-0.02	-0.03

	34	35	36
	A	A	A
Frequencies --	924.7406	947.7576	982.0838
Red. masses --	6.6514	1.4193	1.0996
Frc consts --	3.3512	0.7511	0.6248
IR Inten --	14.1831	0.1794	212.3327
Raman Activ --	0.4551	0.2663	1.2660
Depolar (P) --	0.6660	0.7154	0.2499
Depolar (U) --	0.7995	0.8341	0.3998

Atom	AN	X	Y	Z	X	Y	Z	X	Y	Z
1	6	0.24	0.04	0.01	0.00	0.00	-0.05	0.00	-0.01	-0.01
2	6	0.29	0.14	-0.01	0.00	0.00	0.00	-0.02	0.00	0.01
3	6	-0.04	-0.13	-0.01	0.01	0.01	0.11	0.00	0.01	0.01
4	6	-0.13	-0.12	-0.03	0.00	0.02	0.02	0.02	-0.01	0.01
5	6	0.21	0.16	0.02	0.00	0.00	0.05	-0.01	-0.01	0.00
6	6	-0.14	-0.06	-0.10	0.00	0.00	0.00	-0.01	0.01	-0.01
7	6	-0.01	-0.01	0.02	0.00	-0.01	0.02	-0.01	0.01	-0.01
8	1	-0.20	-0.01	0.04	0.03	-0.01	-0.64	0.01	0.00	-0.05
9	8	-0.08	-0.07	0.15	0.00	0.00	0.00	0.00	0.01	-0.02
10	8	-0.03	0.13	-0.12	0.00	-0.01	0.01	-0.01	-0.02	0.01
11	6	-0.11	0.06	-0.01	-0.01	0.00	-0.13	0.01	-0.01	0.00
12	6	-0.14	0.01	-0.03	-0.01	0.01	-0.02	0.00	0.00	0.00
13	8	0.04	-0.01	0.03	0.00	0.00	0.00	0.01	0.00	-0.01
14	8	0.00	0.03	0.05	0.00	0.00	0.00	0.00	0.00	0.03
15	1	0.32	0.14	-0.10	0.00	0.00	-0.13	-0.04	0.00	0.04
16	1	-0.08	0.12	-0.22	0.01	0.00	-0.02	0.17	0.26	0.05
17	1	-0.13	-0.15	0.07	0.01	-0.07	0.73	0.01	-0.01	-0.02
18	8	-0.11	-0.02	0.11	0.01	0.00	0.00	0.00	0.00	0.00
19	8	0.05	-0.11	-0.07	0.00	-0.01	0.00	0.00	0.00	0.00
20	1	0.09	-0.19	-0.33	-0.01	0.00	0.01	-0.01	0.01	0.01
21	8	0.00	0.01	0.01	0.00	0.00	0.00	-0.03	0.03	0.02
22	1	0.09	-0.21	-0.15	-0.01	0.02	0.01	0.27	-0.74	-0.50
23	1	0.00	-0.06	-0.05	0.00	0.00	0.00	0.08	-0.07	-0.05

	37	38	39
	A	A	A
Frequencies --	988.3143	1092.7331	1143.1877

Red. masses -- 1.3288 2.8110 7.8368
Frc consts -- 0.7647 1.9776 6.0342
IR Inten -- 1.2576 12.3398 60.2942
Raman Activ -- 0.1033 31.3201 0.1926
Depolar (P) -- 0.7498 0.0578 0.3888
Depolar (U) -- 0.8570 0.1092 0.5599

Atom	AN	X	Y	Z	X	Y	Z	X	Y	Z
1	6	-0.01	-0.01	0.02	0.00	-0.05	0.00	0.28	0.02	-0.02
2	6	-0.01	-0.01	0.02	0.06	-0.13	0.00	-0.12	0.29	0.00
3	6	0.00	0.01	-0.10	0.20	0.12	-0.01	0.01	-0.14	0.00
4	6	0.00	0.00	-0.01	0.01	0.01	0.02	0.10	-0.08	0.10
5	6	-0.01	0.00	0.01	0.05	-0.04	-0.01	-0.19	-0.22	0.02
6	6	0.00	0.00	0.00	-0.01	0.03	0.00	-0.01	-0.02	0.01
7	6	0.01	0.00	0.11	-0.06	0.11	0.00	-0.10	0.23	0.01
8	1	-0.01	0.02	0.61	0.63	-0.18	0.05	0.26	-0.31	-0.01
9	8	0.01	0.01	-0.01	0.00	0.00	0.00	-0.08	-0.04	0.06
10	8	0.00	0.00	0.00	-0.01	0.01	-0.01	-0.06	0.11	-0.13
11	6	0.01	0.00	-0.07	-0.23	-0.05	0.01	0.08	-0.09	-0.01
12	6	0.00	0.00	0.00	-0.01	0.00	-0.03	-0.01	-0.16	-0.13
13	8	0.00	0.00	0.00	0.01	0.03	-0.03	-0.04	-0.07	0.08
14	8	0.00	0.00	0.00	-0.03	0.00	0.03	0.08	-0.01	-0.09
15	1	-0.02	0.01	-0.64	-0.07	0.13	0.00	-0.04	0.27	0.00
16	1	0.01	0.01	0.02	-0.02	-0.01	-0.01	-0.07	-0.04	0.09
17	1	0.02	-0.03	0.42	-0.29	-0.55	-0.06	0.05	-0.34	0.00
18	8	0.01	0.00	0.00	0.00	0.00	0.00	0.11	0.03	-0.06
19	8	0.00	0.00	0.00	0.00	0.02	0.02	-0.07	0.14	0.16
20	1	-0.01	0.01	0.01	0.03	-0.01	-0.03	0.14	-0.06	-0.17
21	8	0.00	0.00	0.00	0.00	0.00	0.00	0.01	-0.01	-0.01
22	1	0.02	-0.06	-0.04	0.00	0.01	0.01	-0.03	0.04	0.03
23	1	0.01	0.00	0.00	0.00	0.00	0.00	0.00	0.03	0.02

40 41 42
A A A
Frequencies -- 1170.7083 1185.8448 1209.2535
Red. masses -- 1.7511 2.2051 1.6843
Frc consts -- 1.4140 1.8270 1.4511
IR Inten -- 99.0806 29.2824 127.8603
Raman Activ -- 3.2400 12.0701 5.6055
Depolar (P) -- 0.7488 0.2304 0.3295
Depolar (U) -- 0.8563 0.3745 0.4957

Atom	AN	X	Y	Z	X	Y	Z	X	Y	Z
1	6	-0.06	0.03	0.00	0.11	-0.03	-0.01	-0.05	0.05	0.01
2	6	0.06	-0.03	0.00	0.07	-0.18	0.00	0.05	0.05	-0.01
3	6	-0.06	0.01	0.00	0.00	0.06	0.00	0.01	-0.05	-0.01
4	6	-0.05	0.02	-0.05	0.05	-0.04	0.04	-0.07	0.03	-0.06
5	6	0.00	-0.07	0.00	-0.02	-0.08	0.00	0.06	0.00	0.01
6	6	-0.01	0.00	-0.01	0.00	0.02	0.00	-0.01	-0.01	-0.01
7	6	0.09	0.03	0.00	-0.04	-0.01	0.00	-0.03	-0.04	0.00
8	1	-0.30	0.19	-0.01	-0.36	0.34	-0.03	0.45	-0.39	0.02
9	8	0.02	0.01	-0.01	-0.04	-0.02	0.03	0.02	0.01	-0.02
10	8	0.02	-0.04	0.05	-0.03	0.05	-0.06	0.03	-0.05	0.06
11	6	-0.06	-0.01	0.00	-0.03	0.09	0.00	-0.02	0.04	0.00
12	6	-0.01	-0.07	-0.06	0.00	-0.05	-0.03	-0.02	-0.05	-0.05
13	8	0.01	0.01	0.00	0.01	0.03	-0.03	0.00	0.00	0.01
14	8	-0.01	0.01	0.02	-0.03	0.01	0.03	0.00	0.00	0.00
15	1	0.79	0.34	0.00	-0.27	-0.11	0.00	-0.34	-0.18	-0.01
16	1	0.02	0.03	-0.07	-0.03	-0.05	0.10	0.02	0.07	-0.15
17	1	-0.07	-0.08	0.00	0.02	0.73	0.07	0.02	0.45	0.04
18	8	0.04	0.01	-0.02	0.03	0.01	-0.02	0.03	0.00	-0.02
19	8	-0.04	0.06	0.08	-0.02	0.04	0.05	-0.05	0.03	0.07
20	1	0.12	-0.10	-0.19	0.08	-0.06	-0.12	0.19	-0.21	-0.35

21	8	0.00	0.00	0.00	0.00	0.00	0.00	0.00	0.00	0.00
22	1	0.01	-0.02	-0.01	-0.01	0.01	0.01	0.01	-0.01	-0.01
23	1	0.00	-0.02	-0.01	0.00	0.02	0.01	0.00	-0.02	-0.02

	43	44	45
	A	A	A
Frequencies --	1248.7550	1284.7217	1326.0940
Red. masses --	1.6586	1.9124	7.5200
Frc consts --	1.5239	1.8597	7.7914
IR Inten --	69.2520	224.2532	7.4497
Raman Activ --	9.8707	8.8980	2.7859
Depolar (P) --	0.1788	0.1351	0.7476
Depolar (U) --	0.3034	0.2380	0.8556

Atom	AN	X	Y	Z	X	Y	Z	X	Y	Z
1	6	-0.01	-0.07	0.00	-0.16	0.05	-0.02	0.04	0.35	0.00
2	6	-0.07	-0.02	0.00	0.03	0.02	0.00	-0.34	-0.14	0.00
3	6	0.02	0.03	0.00	-0.06	0.02	0.00	0.21	-0.17	-0.01
4	6	0.02	0.00	0.02	0.11	-0.04	0.08	0.00	-0.03	-0.02
5	6	0.03	0.16	0.02	0.05	0.01	0.00	0.28	-0.17	-0.01
6	6	0.01	0.01	0.02	-0.01	0.01	-0.01	0.02	0.02	0.03
7	6	0.02	-0.04	0.00	0.03	-0.05	0.00	-0.24	-0.12	0.01
8	1	-0.25	0.23	-0.01	0.28	-0.25	0.00	-0.17	0.11	0.00
9	8	0.00	0.00	0.00	-0.02	-0.02	0.02	-0.02	0.00	0.02
10	8	-0.01	0.01	-0.02	0.00	0.05	-0.11	0.01	0.00	0.00
11	6	0.04	0.01	0.00	-0.02	-0.01	0.00	0.04	0.27	0.01
12	6	-0.01	-0.02	-0.01	0.00	0.02	0.00	-0.01	0.05	0.00
13	8	0.00	0.00	0.00	0.00	-0.01	0.01	-0.01	0.01	-0.01
14	8	0.01	0.00	-0.01	0.01	0.00	0.00	0.01	-0.02	-0.02
15	1	-0.19	-0.13	0.01	0.26	0.05	-0.01	0.45	0.19	-0.02
16	1	-0.01	-0.03	0.05	0.10	-0.28	0.75	0.04	0.01	0.07
17	1	0.01	-0.50	-0.04	0.00	0.16	0.01	-0.01	-0.34	-0.03
18	8	0.03	-0.01	-0.03	0.00	-0.01	0.00	-0.02	-0.02	0.01
19	8	-0.06	0.00	0.06	-0.01	-0.01	0.01	0.00	-0.01	0.00
20	1	0.27	-0.34	-0.57	0.06	-0.08	-0.12	0.04	-0.04	-0.06
21	8	0.00	0.00	0.00	0.00	0.00	-0.01	0.00	0.00	0.00
22	1	0.00	-0.01	-0.01	0.00	-0.05	-0.03	0.00	-0.01	-0.01
23	1	0.00	0.01	0.01	0.02	0.07	0.06	0.01	0.00	0.01

	46	47	48
	A	A	A
Frequencies --	1392.9636	1394.6359	1430.1191
Red. masses --	7.4099	2.2593	2.6216
Frc consts --	8.4711	2.5891	3.1591
IR Inten --	204.6828	142.4347	195.4763
Raman Activ --	8.5925	7.2362	18.3737
Depolar (P) --	0.2269	0.1743	0.0435
Depolar (U) --	0.3699	0.2969	0.0834

Atom	AN	X	Y	Z	X	Y	Z	X	Y	Z
1	6	-0.10	-0.07	0.01	0.01	-0.01	0.00	0.07	-0.04	0.00
2	6	0.04	-0.06	-0.01	-0.02	-0.02	0.00	0.01	0.02	0.00
3	6	0.01	0.04	0.00	-0.03	0.05	0.00	0.06	-0.03	0.00
4	6	0.04	-0.02	0.02	0.08	-0.07	0.04	-0.18	0.15	-0.09
5	6	0.14	0.05	0.00	-0.01	-0.06	-0.01	-0.02	-0.08	-0.01
6	6	-0.27	0.40	0.06	-0.05	0.07	0.02	-0.06	0.08	0.01
7	6	0.02	-0.03	0.00	-0.03	0.01	0.00	-0.01	0.07	0.00
8	1	-0.14	0.14	0.01	0.13	-0.07	0.00	-0.20	0.17	0.00
9	8	0.01	0.01	-0.01	0.00	0.02	-0.02	0.01	-0.03	0.03
10	8	-0.02	0.00	0.01	-0.04	0.02	0.02	0.07	-0.05	0.00
11	6	-0.04	0.07	0.01	-0.01	-0.08	0.00	-0.03	-0.04	0.00
12	6	-0.02	-0.19	-0.10	0.02	0.21	0.11	0.01	0.14	0.07
13	8	-0.05	-0.19	0.17	-0.01	-0.03	0.02	-0.01	-0.04	0.04
14	8	0.23	-0.07	-0.21	0.04	-0.02	-0.04	0.05	-0.01	-0.04

15	1	-0.11	-0.09	0.00	0.28	0.16	-0.01	-0.11	0.03	0.00
16	1	0.01	0.14	-0.22	-0.09	0.23	-0.56	0.16	-0.29	0.75
17	1	-0.04	0.02	-0.01	0.03	0.31	0.02	-0.02	0.14	0.01
18	8	-0.04	0.02	0.04	0.03	-0.03	-0.03	0.02	-0.02	-0.02
19	8	0.03	0.06	0.01	-0.03	-0.07	-0.02	-0.01	-0.04	-0.01
20	1	-0.21	0.29	0.46	0.18	-0.27	-0.43	0.09	-0.14	-0.22
21	8	0.01	0.00	0.00	0.01	0.00	0.00	-0.01	0.00	0.00
22	1	-0.02	-0.07	-0.02	-0.02	0.03	0.03	0.02	-0.07	-0.06
23	1	-0.04	-0.02	-0.03	-0.03	-0.04	-0.04	0.03	0.05	0.04

	49	50	51
	A	A	A
Frequencies --	1471.9108	1507.7199	1645.1571
Red. masses --	4.0322	2.8423	7.3551
Frc consts --	5.1471	3.8068	11.7288
IR Inten --	14.0555	21.8650	10.1564
Raman Activ --	4.8163	0.6270	18.8633
Depolar (P) --	0.1933	0.5394	0.7398
Depolar (U) --	0.3240	0.7008	0.8505

Atom	AN	X	Y	Z	X	Y	Z	X	Y	Z
1	6	-0.15	-0.22	0.00	-0.13	0.10	0.00	0.20	0.14	0.00
2	6	-0.10	0.24	0.01	0.11	0.03	0.00	-0.33	-0.13	0.00
3	6	0.14	-0.03	0.00	0.02	-0.14	0.00	-0.26	0.01	0.01
4	6	0.05	-0.01	0.05	0.06	-0.05	0.03	-0.02	0.01	-0.03
5	6	0.26	-0.07	-0.01	-0.05	-0.17	-0.01	0.26	0.04	0.00
6	6	0.05	-0.08	-0.01	-0.02	0.00	-0.01	0.01	-0.01	0.05
7	6	-0.04	0.10	0.00	0.18	0.07	0.00	0.38	0.17	-0.01
8	1	-0.46	0.43	0.00	-0.34	0.10	0.00	0.08	-0.28	0.00
9	8	0.02	0.01	-0.02	0.00	0.01	-0.01	-0.02	-0.01	0.02
10	8	-0.02	0.01	-0.01	-0.01	0.01	0.00	0.00	0.00	0.00
11	6	-0.08	-0.08	0.00	-0.08	0.13	0.01	-0.17	-0.20	0.00
12	6	-0.01	-0.01	-0.04	0.01	0.08	0.04	0.01	-0.01	-0.03
13	8	0.00	0.02	-0.01	0.00	-0.01	0.01	0.00	0.02	-0.02
14	8	-0.02	0.01	0.02	0.00	0.00	0.00	0.01	-0.01	-0.01
15	1	-0.09	0.10	0.00	-0.61	-0.29	0.01	-0.46	-0.20	0.01
16	1	-0.04	0.02	-0.12	-0.02	0.06	-0.13	0.01	0.00	0.03
17	1	-0.04	0.54	0.03	-0.15	-0.44	-0.02	-0.16	0.26	0.02
18	8	-0.03	-0.01	0.02	0.01	-0.01	-0.01	-0.03	-0.01	0.02
19	8	0.01	0.01	0.00	0.00	-0.02	-0.01	0.00	0.00	0.00
20	1	0.00	0.02	0.06	0.04	-0.05	-0.09	0.01	0.00	0.02
21	8	0.00	0.00	0.00	0.00	0.00	0.00	0.00	0.00	0.00
22	1	0.00	0.02	0.01	-0.01	0.01	0.02	0.01	0.00	-0.03
23	1	0.00	0.00	0.00	-0.01	-0.01	-0.01	0.02	0.00	0.01

	52	53	54
	A	A	A
Frequencies --	1664.5125	1696.1064	1729.9781
Red. masses --	6.5067	1.0938	10.6006
Frc consts --	10.6215	1.8539	18.6923
IR Inten --	9.3714	40.5394	556.0511
Raman Activ --	32.6496	0.5858	12.2737
Depolar (P) --	0.7477	0.6102	0.7417
Depolar (U) --	0.8556	0.7580	0.8517

Atom	AN	X	Y	Z	X	Y	Z	X	Y	Z
1	6	0.13	-0.29	0.00	0.00	0.00	0.00	-0.02	0.00	0.00
2	6	-0.07	0.15	0.00	0.00	0.00	0.00	0.05	0.03	-0.01
3	6	-0.24	0.26	0.01	0.00	0.00	0.00	0.02	0.00	0.00
4	6	-0.04	0.04	-0.01	0.00	0.01	-0.01	0.00	0.02	-0.01
5	6	0.12	-0.27	-0.02	0.00	0.00	0.00	-0.02	-0.01	0.00
6	6	0.01	-0.02	0.00	0.02	0.01	-0.02	-0.41	-0.31	0.58
7	6	0.06	-0.17	0.00	0.00	0.00	0.00	-0.01	0.00	0.00
8	1	0.42	-0.23	-0.01	0.00	0.00	0.00	-0.02	0.02	0.00

9 8 0.01 -0.01 0.00 0.00 0.00 0.00 0.00 -0.01 0.00
10 8 0.00 -0.01 0.00 0.00 0.00 0.00 0.00 0.00 0.00
11 6 -0.01 0.35 0.01 0.00 0.00 0.00 0.01 0.01 0.00
12 6 0.01 0.05 0.00 0.00 0.00 0.00 0.01 -0.02 0.00
13 8 0.00 0.00 0.00 -0.01 -0.01 0.01 0.12 0.24 -0.26
14 8 0.00 0.00 0.00 -0.01 0.01 0.00 0.16 -0.02 -0.17
15 1 0.11 -0.18 -0.01 0.00 0.00 0.00 0.02 0.01 0.00
16 1 0.00 -0.02 0.04 0.01 0.00 0.03 0.06 0.02 0.08
17 1 -0.09 -0.47 -0.01 0.00 0.00 0.00 0.01 -0.03 0.00
18 8 -0.01 -0.01 0.01 0.00 0.00 0.00 0.00 0.00 0.00
19 8 0.00 0.00 0.00 0.00 0.00 0.00 0.00 0.01 0.00
20 1 0.02 -0.02 -0.02 0.00 0.00 0.00 0.02 0.00 0.03
21 8 0.00 0.00 0.00 0.05 0.03 -0.02 0.03 0.01 0.00
22 1 0.00 -0.01 0.00 -0.32 -0.52 0.51 -0.19 -0.29 0.08
23 1 -0.01 0.00 0.00 -0.56 -0.08 -0.21 -0.22 -0.02 -0.08
55 56 57
A A A
Frequencies -- 1817.7707 1821.1027 3185.9082
Red. masses -- 10.5429 11.1484 1.0862
Frc consts -- 20.5252 21.7836 6.4959
IR Inten -- 805.6491 105.5664 0.9816
Raman Activ -- 10.2915 74.4742 53.8738
Depolar (P) -- 0.3404 0.1954 0.6575
Depolar (U) -- 0.5079 0.3269 0.7933
Atom AN X Y Z X Y Z X Y Z
1 6 -0.04 -0.01 0.01 0.07 0.03 -0.02 0.00 0.00 0.00
2 6 0.02 -0.01 0.00 0.01 -0.02 0.00 0.00 0.00 0.00
3 6 0.01 0.00 0.00 0.00 -0.01 0.00 -0.03 -0.05 0.00
4 6 0.23 0.17 -0.24 -0.42 -0.30 0.42 0.00 0.00 0.00
5 6 -0.08 -0.03 0.01 -0.05 -0.02 0.01 0.00 0.00 0.00
6 6 0.00 0.01 -0.01 -0.01 0.01 0.01 0.00 0.00 0.00
7 6 -0.01 0.00 0.00 0.00 0.01 0.00 -0.02 0.05 0.00
8 1 0.01 0.01 0.01 -0.04 0.00 -0.01 0.42 0.56 -0.01
9 8 -0.14 -0.11 0.15 0.25 0.20 -0.27 0.00 0.00 0.00
10 8 -0.01 -0.02 0.03 0.01 0.03 -0.04 0.00 0.00 0.00
11 6 0.02 -0.01 0.00 0.01 -0.01 0.00 0.03 0.00 0.00
12 6 0.54 0.10 -0.33 0.33 0.06 -0.20 0.00 0.00 0.00
13 8 0.00 0.00 0.01 0.00 0.00 0.00 0.00 0.00 0.00
14 8 0.00 0.00 0.00 0.01 0.00 -0.01 0.00 0.00 0.00
15 1 0.04 0.02 0.00 -0.01 0.01 0.00 0.25 -0.57 -0.02
16 1 -0.02 0.08 -0.14 0.04 -0.15 0.24 0.00 0.00 0.00
17 1 0.03 0.02 0.02 0.02 0.02 0.01 -0.34 0.03 0.01
18 8 -0.33 -0.04 0.22 -0.20 -0.02 0.13 0.00 0.00 0.00
19 8 -0.05 0.00 0.04 -0.03 0.00 0.02 0.00 0.00 0.00
20 1 0.18 -0.21 -0.32 0.11 -0.12 -0.19 0.00 0.00 0.00
21 8 0.00 0.00 0.00 0.01 0.00 -0.01 0.00 0.00 0.00
22 1 0.03 0.02 -0.03 -0.05 -0.05 0.07 0.01 0.00 0.00
23 1 0.02 -0.01 0.00 -0.06 0.01 0.00 0.00 0.00 0.00
58 59 60
A A A
Frequencies -- 3198.5906 3213.2942 3285.4193
Red. masses -- 1.0914 1.0977 1.0678
Frc consts -- 6.5786 6.6776 6.7907
IR Inten -- 9.7796 33.3432 907.2109
Raman Activ -- 77.6538 229.5056 181.1209
Depolar (P) -- 0.6784 0.1431 0.3121
Depolar (U) -- 0.8084 0.2504 0.4757
Atom AN X Y Z X Y Z X Y Z
1 6 0.00 0.00 0.00 0.00 0.00 0.00 0.00 0.00 0.00
2 6 0.00 0.00 0.00 0.00 0.00 0.00 0.00 0.00 0.00

3	6	-0.03	-0.04	0.00	-0.02	-0.02	0.00	0.00	0.00	0.00
4	6	0.00	0.00	0.00	0.00	0.00	0.00	0.00	0.00	0.00
5	6	0.00	0.00	0.00	0.00	0.00	0.00	0.00	0.00	0.00
6	6	0.00	0.00	0.00	0.00	0.00	0.00	0.01	0.00	-0.01
7	6	0.02	-0.02	0.00	0.03	-0.06	0.00	0.00	0.00	0.00
8	1	0.37	0.50	-0.01	0.20	0.27	0.00	0.00	0.01	0.00
9	8	0.00	0.00	0.00	0.00	0.00	0.00	0.00	0.00	0.00
10	8	0.00	0.00	0.00	0.00	0.00	0.00	-0.01	0.00	0.00
11	6	-0.06	0.01	0.00	0.05	0.00	0.00	0.00	0.00	0.00
12	6	0.00	0.00	0.00	0.00	0.00	0.00	0.00	0.00	0.00
13	8	0.00	0.00	0.00	0.00	0.00	0.00	0.00	0.00	0.00
14	8	0.00	0.00	0.00	0.00	0.00	0.00	-0.01	0.00	0.00
15	1	-0.13	0.28	0.01	-0.29	0.65	0.02	0.00	0.00	0.00
16	1	0.00	0.00	0.00	0.00	0.00	0.00	0.13	-0.06	-0.04
17	1	0.71	-0.06	-0.02	-0.60	0.05	0.02	-0.01	0.00	0.00
18	8	0.00	0.00	0.00	0.00	0.00	0.00	0.00	0.00	0.00
19	8	0.00	0.00	0.00	0.00	0.00	0.00	0.00	0.00	0.00
20	1	0.00	0.00	0.00	0.00	0.00	0.00	0.00	0.00	0.00
21	8	0.00	0.00	0.00	0.00	0.00	0.00	0.05	0.00	0.03
22	1	0.00	0.00	0.00	0.01	0.00	0.01	-0.83	0.04	-0.53
23	1	0.00	0.00	0.00	0.00	0.00	0.00	0.02	-0.03	0.05

	61	62	63
	A	A	A
Frequencies --	3548.9336	3863.6176	3940.0062
Red. masses --	1.0662	1.0642	1.0648
Frc consts --	7.9120	9.3600	9.7388
IR Inten --	807.8973	72.5934	37.6174
Raman Activ --	101.7664	141.6030	46.8750
Depolar (P) --	0.2257	0.2582	0.1830
Depolar (U) --	0.3683	0.4104	0.3094

Atom	AN	X	Y	Z	X	Y	Z	X	Y	Z
1	6	0.00	0.00	0.00	0.00	0.00	0.00	0.00	0.00	0.00
2	6	0.00	0.00	0.00	0.00	0.00	0.00	0.00	0.00	0.00
3	6	0.00	0.00	0.00	0.00	0.00	0.00	0.00	0.00	0.00
4	6	0.00	0.00	0.00	0.00	0.00	0.00	0.00	0.00	0.00
5	6	0.00	0.00	0.00	0.00	0.00	0.00	0.00	0.00	0.00
6	6	0.00	0.00	0.00	0.00	0.00	0.00	0.00	0.00	0.00
7	6	0.00	0.00	0.00	0.00	0.00	0.00	0.00	0.00	0.00
8	1	0.00	0.00	0.00	0.00	0.00	0.00	0.00	0.00	0.00
9	8	0.00	0.00	0.00	0.00	0.00	0.00	0.00	0.00	0.00
10	8	-0.05	0.03	0.02	0.00	0.00	0.00	0.00	0.00	0.00
11	6	0.00	0.00	0.00	0.00	0.00	0.00	0.00	0.00	0.00
12	6	0.00	0.00	0.00	0.00	0.00	0.00	0.00	0.00	0.00
13	8	0.00	0.00	0.00	0.00	0.00	0.00	0.00	0.00	0.00
14	8	0.00	0.00	0.00	0.00	0.00	0.00	0.00	0.00	0.00
15	1	0.00	0.00	0.00	0.00	0.00	0.00	0.00	0.00	0.00
16	1	0.81	-0.49	-0.27	0.00	0.00	0.00	-0.01	0.01	0.00
17	1	0.00	0.00	0.00	0.00	0.00	0.00	0.00	0.00	0.00
18	8	0.00	0.00	0.00	0.00	0.00	0.00	0.00	0.00	0.00
19	8	0.00	0.00	0.00	0.04	0.05	-0.01	0.00	0.00	0.00
20	1	0.00	0.00	0.00	-0.58	-0.80	0.12	0.00	0.00	0.00
21	8	-0.01	0.00	-0.01	0.00	0.00	0.00	0.01	0.04	-0.05
22	1	0.13	-0.01	0.08	0.00	0.00	0.00	0.04	0.02	0.00
23	1	0.00	-0.01	0.00	0.00	0.00	0.00	-0.17	-0.62	0.76

HEM H20

Harmonic frequencies (cm**-1), IR intensities (KM/Mole), Raman scattering
activities (A**4/AMU), depolarization ratios for plane and unpolarized

incident light, reduced masses (AMU), force constants (mDyne/A),
and normal coordinates:

		1			2			3		
		A			A			A		
Frequencies --		21.7495			39.2740			59.6302		
Red. masses --		10.6412			6.9155			6.9831		
Frc consts --		0.0030			0.0063			0.0146		
IR Inten --		1.9610			1.3895			0.9815		
Raman Activ --		0.6445			0.1404			0.0999		
Depolar (P) --		0.7496			0.7495			0.7212		
Depolar (U) --		0.8569			0.8568			0.8380		
Atom	AN	X	Y	Z	X	Y	Z	X	Y	Z
1	8	0.00	0.02	0.05	0.01	0.02	0.39	0.00	0.05	0.22
2	1	0.04	-0.19	0.11	-0.15	0.05	0.54	-0.11	-0.13	0.41
3	1	0.00	0.03	0.30	0.01	0.01	0.20	0.00	0.04	0.30
4	6	0.00	0.00	-0.03	0.00	0.00	-0.12	0.00	-0.01	-0.02
5	6	0.00	0.00	-0.01	0.00	0.00	-0.08	0.01	-0.01	0.02
6	6	0.00	0.00	-0.04	0.00	0.00	-0.14	0.00	-0.01	0.06
7	6	0.00	0.00	0.01	0.00	0.00	-0.10	0.00	-0.02	-0.12
8	6	0.00	0.00	0.02	0.00	0.00	-0.01	0.00	0.00	0.07
9	6	0.02	0.00	-0.02	0.00	0.00	-0.10	-0.01	-0.01	-0.03
10	6	0.00	0.00	0.00	0.00	0.00	-0.11	-0.01	0.00	0.17
11	1	0.00	0.00	-0.07	0.00	-0.01	-0.17	-0.01	-0.01	0.03
12	8	0.00	0.03	0.37	0.00	-0.01	-0.20	-0.01	0.00	0.05
13	8	0.00	-0.04	-0.37	-0.01	0.01	0.06	0.01	-0.03	-0.39
14	6	0.00	0.00	0.04	0.00	0.00	-0.03	0.00	0.00	0.16
15	6	0.00	0.00	-0.02	0.00	0.00	0.11	0.01	0.01	0.02
16	8	0.23	-0.04	-0.05	-0.06	-0.02	-0.11	-0.06	-0.04	-0.05
17	8	-0.21	0.04	0.00	0.07	0.01	-0.12	0.03	0.03	-0.06
18	1	0.00	0.00	0.01	0.00	0.00	-0.14	-0.01	0.00	0.25
19	1	0.00	-0.03	-0.29	-0.01	0.01	0.15	0.01	-0.02	-0.31
20	1	0.00	0.00	0.07	0.00	0.00	0.02	-0.01	0.01	0.21
21	8	0.02	0.07	-0.35	0.04	0.01	0.06	-0.02	-0.03	0.21
22	8	-0.04	-0.07	0.35	-0.03	-0.01	0.30	0.05	0.05	-0.27
23	1	-0.03	-0.05	0.28	-0.02	0.00	0.37	0.04	0.04	-0.27
24	1	-0.19	0.03	-0.01	0.07	0.01	-0.14	0.01	0.03	-0.09

		4			5			6		
		A			A			A		
Frequencies --		85.6143			90.7158			152.3458		
Red. masses --		8.1127			5.9929			8.5829		
Frc consts --		0.0350			0.0291			0.1174		
IR Inten --		3.5635			5.4212			2.8234		
Raman Activ --		1.5031			0.3454			1.7312		
Depolar (P) --		0.7459			0.7496			0.7480		
Depolar (U) --		0.8545			0.8569			0.8559		
Atom	AN	X	Y	Z	X	Y	Z	X	Y	Z
1	8	0.07	-0.15	-0.09	-0.22	0.35	-0.05	0.03	-0.02	0.00
2	1	0.22	-0.25	-0.18	-0.20	0.53	-0.16	-0.01	0.07	0.00
3	1	0.14	-0.07	0.07	-0.40	0.17	0.03	0.05	-0.01	-0.15
4	6	0.01	0.04	0.04	-0.01	-0.10	0.02	0.01	-0.02	-0.10
5	6	0.00	0.02	0.04	0.02	-0.06	0.02	0.01	-0.02	-0.04
6	6	0.02	0.03	-0.08	-0.05	-0.09	-0.02	0.01	-0.02	-0.09
7	6	0.01	0.03	0.16	-0.02	-0.08	0.06	0.01	0.00	-0.08
8	6	0.00	0.00	-0.01	0.00	-0.01	0.00	0.01	0.00	0.06
9	6	-0.04	0.02	-0.10	0.08	-0.07	-0.03	0.00	-0.01	-0.02
10	6	0.03	0.01	-0.19	-0.07	-0.05	-0.06	0.00	-0.01	0.02
11	1	0.03	0.04	-0.10	-0.07	-0.12	-0.01	0.00	-0.02	-0.13
12	8	0.04	0.04	0.32	-0.08	-0.07	0.09	-0.01	-0.01	-0.17
13	8	-0.01	0.01	0.06	0.03	-0.04	0.04	0.01	0.02	0.10
14	6	0.02	0.00	-0.13	-0.04	-0.01	-0.05	0.00	0.00	0.10

15	6	-0.01	-0.02	0.11	0.05	0.05	0.02	0.03	0.02	0.08
16	8	-0.08	-0.15	-0.20	0.11	-0.13	-0.06	0.36	-0.03	-0.04
17	8	-0.05	0.20	-0.20	0.09	-0.03	-0.05	-0.49	0.01	0.04
18	1	0.04	0.01	-0.31	-0.11	-0.04	-0.10	-0.01	-0.01	0.05
19	1	0.01	-0.02	0.06	-0.01	0.03	0.04	0.01	0.02	0.11
20	1	0.02	-0.01	-0.19	-0.06	0.03	-0.07	0.00	0.00	0.19
21	8	-0.03	-0.05	0.29	0.13	0.04	0.07	0.03	0.00	0.19
22	8	0.00	0.00	0.00	0.02	0.13	0.00	0.03	0.07	-0.07
23	1	-0.01	-0.02	0.10	0.05	0.17	0.02	0.04	0.07	-0.05
24	1	-0.08	0.18	-0.35	0.12	-0.04	-0.10	-0.60	0.03	0.08

7 8 9

A A A

Frequencies -- 168.1038 169.9075 183.2099

Red. masses -- 6.6738 7.7652 5.2673

Frc consts -- 0.1111 0.1321 0.1042

IR Inten -- 2.3987 0.9194 1.6729

Raman Activ -- 1.5019 0.5646 0.9048

Depolar (P) -- 0.7492 0.7391 0.7443

Depolar (U) -- 0.8566 0.8500 0.8534

Atom	AN	X	Y	Z	X	Y	Z	X	Y	Z
1	8	0.02	0.01	0.01	-0.20	-0.04	0.01	0.25	0.09	0.00
2	1	-0.11	0.01	0.15	-0.17	0.02	-0.05	0.02	-0.02	0.28
3	1	0.01	-0.01	-0.09	-0.25	-0.09	0.02	0.29	0.11	-0.14
4	6	0.01	-0.01	0.12	-0.03	0.06	-0.01	-0.04	-0.01	0.18
5	6	0.01	0.00	0.31	-0.01	0.08	0.03	-0.04	-0.01	0.07
6	6	0.00	-0.02	-0.15	-0.01	0.06	-0.05	-0.05	0.00	0.24
7	6	0.00	-0.02	-0.02	-0.02	0.01	-0.02	-0.03	0.02	0.00
8	6	0.01	0.00	0.26	-0.01	0.06	0.05	-0.05	-0.01	-0.10
9	6	-0.02	-0.01	0.16	0.16	0.06	0.00	0.03	-0.03	0.03
10	6	0.00	-0.01	-0.19	0.00	0.05	-0.03	-0.05	0.00	-0.02
11	1	0.00	-0.03	-0.36	0.00	0.07	-0.10	-0.05	-0.01	0.40
12	8	-0.01	-0.03	-0.15	0.03	0.00	-0.03	-0.05	0.00	-0.12
13	8	0.01	-0.01	-0.03	-0.06	-0.02	0.01	0.01	0.04	-0.10
14	6	0.00	0.00	0.05	0.01	0.06	0.04	-0.05	-0.01	-0.25
15	6	0.02	0.02	0.02	-0.11	-0.07	0.03	-0.06	-0.01	-0.01
16	8	-0.06	-0.23	0.04	0.36	0.03	-0.02	0.15	-0.09	0.00
17	8	-0.02	0.24	0.04	0.21	0.09	-0.01	0.02	0.02	0.01
18	1	-0.01	-0.02	-0.43	0.00	0.05	-0.06	-0.05	0.00	-0.07
19	1	0.01	-0.01	-0.08	-0.04	-0.06	0.02	-0.01	0.06	-0.17
20	1	0.00	0.00	-0.02	0.02	0.05	0.05	-0.05	-0.01	-0.47
21	8	-0.01	0.06	-0.18	-0.30	-0.07	0.06	-0.04	-0.03	0.07
22	8	0.05	0.00	-0.07	-0.01	-0.24	-0.04	-0.08	0.01	0.05
23	1	0.05	0.02	-0.32	-0.12	-0.36	-0.05	-0.08	0.01	0.14
24	1	-0.07	0.21	-0.17	0.49	0.05	-0.07	0.13	0.00	-0.04

10 11 12

A A A

Frequencies -- 187.0155 201.4397 257.6152

Red. masses -- 5.1730 6.7406 1.1180

Frc consts -- 0.1066 0.1612 0.0437

IR Inten -- 1.0684 18.7280 128.5121

Raman Activ -- 0.6379 0.2912 0.0926

Depolar (P) -- 0.7195 0.4571 0.7114

Depolar (U) -- 0.8369 0.6274 0.8314

Atom	AN	X	Y	Z	X	Y	Z	X	Y	Z
1	8	0.32	0.12	-0.03	-0.12	-0.20	0.01	-0.03	0.00	0.05
2	1	0.35	0.10	-0.05	-0.12	-0.46	0.13	0.64	0.09	-0.65
3	1	0.38	0.18	-0.02	0.09	0.03	-0.02	0.10	0.14	0.34
4	6	-0.04	-0.03	-0.12	-0.04	0.03	0.00	0.00	0.01	0.01
5	6	-0.05	-0.03	-0.03	-0.07	-0.05	0.03	-0.01	0.00	0.01
6	6	-0.06	-0.03	-0.19	0.05	0.02	-0.02	0.00	0.01	0.02

7 6 -0.04 0.02 0.01 -0.10 0.18 -0.01 0.00 0.01 -0.02
8 6 -0.07 0.00 0.09 -0.04 -0.10 0.03 -0.01 -0.01 0.00
9 6 0.02 -0.05 -0.02 0.02 -0.07 0.01 -0.01 -0.01 0.01
10 6 -0.07 -0.01 0.01 0.08 -0.05 0.00 0.00 0.00 0.00
11 1 -0.07 -0.05 -0.32 0.09 0.07 -0.05 0.00 0.01 0.02
12 8 -0.04 0.02 0.10 -0.20 0.21 0.00 0.01 0.00 -0.03
13 8 0.00 0.05 0.08 -0.06 0.25 -0.01 -0.01 0.00 -0.02
14 6 -0.07 0.00 0.19 0.02 -0.11 0.03 0.00 -0.01 -0.02
15 6 -0.07 0.01 0.02 0.03 -0.03 0.00 -0.01 0.00 0.00
16 8 0.05 -0.03 -0.01 0.13 -0.11 -0.01 0.01 -0.02 0.00
17 8 0.11 -0.10 -0.01 0.11 -0.09 0.00 0.00 0.00 0.00
18 1 -0.08 -0.01 0.03 0.14 -0.06 -0.02 0.01 0.00 0.00
19 1 -0.02 0.08 0.11 -0.06 0.27 -0.03 0.00 -0.02 0.03
20 1 -0.07 0.00 0.37 0.04 -0.16 0.04 0.00 -0.01 -0.04
21 8 -0.08 0.02 -0.03 0.19 -0.03 -0.02 0.00 0.00 0.00
22 8 -0.05 0.00 -0.05 -0.04 0.12 -0.02 -0.01 0.01 0.00
23 1 -0.06 0.01 -0.12 0.04 0.21 -0.06 -0.01 0.02 0.00
24 1 0.25 -0.11 0.01 0.36 -0.11 -0.02 0.02 0.00 -0.01
13 14 15
A A A
Frequencies -- 285.5712 302.7144 358.5527
Red. masses -- 5.3863 9.7603 5.1087
Frc consts -- 0.2588 0.5270 0.3870
IR Inten -- 47.5487 1.5542 6.3156
Raman Activ -- 2.6586 2.0303 1.1682
Depolar (P) -- 0.7172 0.7326 0.2290
Depolar (U) -- 0.8353 0.8456 0.3726
Atom AN X Y Z X Y Z X Y Z
1 8 0.04 0.07 -0.01 -0.11 -0.03 0.00 0.04 0.00 0.03
2 1 -0.17 0.37 0.06 -0.23 0.02 0.10 0.04 0.34 -0.14
3 1 -0.20 -0.19 -0.13 -0.21 -0.13 -0.05 -0.03 -0.09 -0.20
4 6 -0.05 0.13 -0.01 0.12 0.06 0.04 -0.04 0.05 0.18
5 6 -0.14 0.04 0.02 0.00 -0.08 0.01 -0.03 0.02 -0.01
6 6 0.05 0.12 -0.03 0.11 0.08 0.03 -0.01 0.05 0.12
7 6 0.01 0.02 0.00 0.18 0.09 0.01 -0.07 0.03 0.06
8 6 -0.09 -0.12 0.01 -0.07 0.07 0.04 0.01 0.00 0.23
9 6 -0.11 0.05 0.02 -0.03 -0.21 -0.02 -0.04 0.01 -0.15
10 6 0.11 -0.02 0.01 0.07 0.13 -0.04 0.01 0.01 -0.20
11 1 0.10 0.20 -0.06 0.07 0.03 0.03 0.01 0.07 0.15
12 8 0.25 -0.03 0.00 0.28 0.07 -0.01 -0.02 0.01 -0.02
13 8 -0.13 -0.14 0.00 0.14 0.04 -0.01 -0.13 -0.04 -0.05
14 6 0.02 -0.13 0.03 0.00 0.07 -0.01 0.01 0.00 0.04
15 6 -0.07 -0.09 -0.01 -0.13 0.14 0.02 0.06 0.01 0.11
16 8 0.06 0.03 0.01 -0.05 -0.28 -0.05 0.01 0.14 -0.08
17 8 0.02 0.06 0.00 -0.04 -0.33 0.02 -0.03 -0.21 -0.06
18 1 0.24 -0.04 0.03 0.08 0.13 -0.11 0.04 0.00 -0.52
19 1 -0.08 -0.27 0.04 0.16 0.02 -0.02 -0.10 -0.12 -0.01
20 1 0.05 -0.22 0.05 0.04 -0.03 -0.04 0.01 -0.01 -0.01
21 8 0.14 -0.10 -0.01 -0.25 0.15 0.00 0.03 0.04 -0.02
22 8 -0.18 0.11 -0.02 -0.13 0.12 0.00 0.15 -0.06 -0.02
23 1 -0.06 0.24 -0.03 -0.12 0.13 -0.02 0.09 -0.10 -0.28
24 1 0.31 0.02 -0.05 -0.04 -0.30 0.19 0.06 -0.18 0.16
16 17 18
A A A
Frequencies -- 364.7360 386.1403 415.4099
Red. masses -- 1.3734 4.8129 6.8224
Frc consts -- 0.1076 0.4228 0.6937
IR Inten -- 68.3696 33.2210 3.6415
Raman Activ -- 0.5819 2.8792 3.2406
Depolar (P) -- 0.4206 0.1505 0.5889

Depolar (U) --	0.5921	0.2617	0.7413

Atom	AN	X	Y	Z	X	Y	Z	X	Y	Z
1	8	0.03	-0.05	0.05	-0.01	-0.08	0.05	0.06	0.05	-0.02
2	1	-0.05	0.70	-0.23	0.00	0.44	-0.20	0.06	-0.13	0.07
3	1	-0.09	-0.20	-0.56	0.02	-0.07	-0.48	0.03	0.02	0.22
4	6	0.00	0.00	-0.05	0.04	-0.09	0.09	-0.02	-0.03	-0.07
5	6	0.01	0.00	0.00	0.03	-0.02	-0.01	0.03	0.01	-0.02
6	6	0.00	0.00	-0.01	0.07	-0.10	0.03	0.24	-0.03	0.01
7	6	-0.02	0.02	-0.02	0.06	-0.08	0.04	-0.17	-0.05	-0.02
8	6	0.01	0.01	-0.04	-0.02	-0.03	0.07	0.04	0.05	-0.02
9	6	0.01	-0.02	0.03	0.05	0.06	-0.06	0.07	-0.02	0.02
10	6	-0.01	0.01	0.04	0.07	-0.08	-0.08	0.28	-0.03	0.04
11	1	-0.01	0.00	0.01	0.09	-0.08	0.01	0.33	0.09	0.05
12	8	-0.02	0.03	0.03	-0.11	-0.05	0.01	-0.19	-0.06	0.01
13	8	-0.04	0.01	-0.03	0.20	0.04	-0.05	-0.25	-0.14	0.03
14	6	0.00	0.02	-0.03	0.06	-0.06	0.06	0.26	0.00	-0.05
15	6	0.02	0.01	-0.01	-0.11	-0.02	0.02	-0.03	0.11	0.01
16	8	-0.01	-0.06	0.01	0.01	0.18	0.01	-0.02	-0.06	0.00
17	8	0.01	0.01	0.02	0.01	0.06	-0.05	0.01	0.00	0.02
18	1	-0.02	0.02	0.11	0.04	-0.08	-0.21	0.24	-0.02	0.10
19	1	-0.01	-0.04	0.20	0.16	0.16	0.10	-0.22	-0.23	-0.02
20	1	0.00	0.02	-0.03	0.07	-0.09	0.08	0.31	-0.12	-0.07
21	8	0.00	0.01	0.01	-0.09	-0.01	-0.02	-0.16	0.13	0.00
22	8	0.04	-0.02	0.01	-0.21	0.13	-0.01	-0.01	0.10	0.01
23	1	0.01	-0.05	0.04	-0.08	0.28	-0.05	-0.02	0.10	0.02
24	1	-0.01	0.01	0.00	-0.11	0.07	-0.06	-0.21	0.02	0.04

	19	20	21
	A	A	A
Frequencies --	451.8998	510.2872	534.6194
Red. masses --	3.9234	1.9306	7.1356
Frc consts --	0.4721	0.2962	1.2016
IR Inten --	4.9023	39.4496	38.7950
Raman Activ --	0.7993	1.2566	4.8504
Depolar (P) --	0.4561	0.7233	0.2002
Depolar (U) --	0.6265	0.8395	0.3337

Atom	AN	X	Y	Z	X	Y	Z	X	Y	Z
1	8	0.01	0.00	0.01	0.01	0.01	-0.01	0.00	-0.01	0.01
2	1	0.04	0.06	-0.05	-0.01	0.01	0.01	0.03	-0.05	-0.01
3	1	0.02	0.01	-0.04	-0.05	-0.05	0.06	0.13	0.13	-0.12
4	6	0.00	0.02	0.28	0.01	-0.02	-0.01	0.00	0.12	-0.08
5	6	0.03	0.02	0.02	0.07	-0.01	0.00	0.02	0.15	0.05
6	6	0.01	0.02	-0.14	-0.02	-0.04	0.03	0.01	0.21	0.00
7	6	-0.02	0.00	0.14	-0.01	0.06	-0.02	0.01	-0.16	-0.03
8	6	0.02	0.04	-0.22	0.03	0.02	0.04	0.03	0.13	0.10
9	6	0.03	-0.01	-0.01	0.14	-0.02	-0.01	0.00	0.04	-0.01
10	6	0.01	0.05	-0.03	-0.04	0.00	-0.02	0.04	0.25	0.03
11	1	0.01	0.01	-0.41	-0.05	-0.07	0.05	0.01	0.22	0.02
12	8	-0.04	-0.01	-0.06	0.05	0.06	0.00	-0.22	-0.12	0.03
13	8	-0.04	-0.03	-0.05	-0.09	0.01	0.00	0.13	-0.08	0.02
14	6	0.02	0.06	0.17	-0.01	0.04	0.01	0.05	0.20	-0.05
15	6	0.01	0.00	-0.16	-0.02	-0.07	0.01	-0.05	-0.14	0.03
16	8	-0.01	-0.03	-0.02	-0.01	0.00	0.00	-0.02	-0.11	-0.10
17	8	-0.01	-0.04	0.01	0.06	-0.01	0.00	-0.01	-0.11	0.07
18	1	-0.01	0.05	-0.09	-0.08	0.01	-0.06	0.04	0.25	0.04
19	1	-0.03	-0.06	-0.15	-0.05	-0.07	0.01	0.07	0.06	0.06
20	1	0.02	0.07	0.45	-0.03	0.09	-0.02	0.06	0.20	-0.19
21	8	0.03	-0.05	0.04	0.03	-0.07	-0.01	0.17	-0.16	-0.02
22	8	-0.03	-0.01	0.00	-0.08	0.01	-0.01	-0.14	-0.05	-0.01
23	1	-0.06	-0.08	0.53	0.00	0.10	-0.07	-0.02	0.11	-0.37
24	1	-0.10	-0.02	0.07	-0.92	0.11	0.10	0.06	-0.08	0.33

	22			23			24		
	A			A			A		
Frequencies --	582.1818			584.2399			595.6869		
Red. masses --	1.9802			1.7888			1.4133		
Frc consts --	0.3954			0.3598			0.2955		
IR Inten --	43.2998			21.5761			218.2129		
Raman Activ --	1.3828			1.8297			4.2806		
Depolar (P) --	0.7446			0.4352			0.4183		
Depolar (U) --	0.8536			0.6064			0.5899		

Atom	AN	X	Y	Z	X	Y	Z	X	Y	Z
1	8	-0.03	0.00	0.02	0.04	-0.01	-0.01	-0.04	0.01	0.01
2	1	0.03	-0.08	-0.01	-0.04	0.13	-0.01	0.03	-0.12	0.01
3	1	0.26	0.28	-0.28	-0.35	-0.38	0.36	0.33	0.37	-0.34
4	6	-0.03	0.04	0.07	0.03	-0.04	0.06	0.01	-0.03	0.02
5	6	-0.06	0.01	0.07	0.05	0.00	0.09	0.04	-0.01	-0.06
6	6	-0.01	0.05	-0.11	0.00	-0.07	-0.09	0.01	-0.04	0.01
7	6	0.00	-0.02	0.04	0.00	0.03	0.01	-0.01	0.02	0.02
8	6	-0.04	-0.04	-0.03	0.04	0.02	0.00	0.03	0.02	-0.06
9	6	-0.01	0.01	-0.03	0.01	0.02	-0.04	0.02	-0.01	0.02
10	6	0.02	-0.02	0.12	-0.02	-0.01	0.10	-0.01	0.00	-0.03
11	1	0.02	0.08	-0.30	-0.02	-0.10	-0.26	-0.01	-0.06	0.06
12	8	0.01	-0.04	0.00	0.00	0.04	-0.02	0.05	0.01	0.00
13	8	0.03	-0.01	-0.01	-0.03	0.02	0.00	-0.05	-0.01	-0.01
14	6	-0.02	-0.07	-0.08	0.02	0.04	-0.07	0.02	0.04	0.03
15	6	0.01	0.03	0.03	0.00	-0.02	0.01	0.00	-0.02	0.02
16	8	0.02	0.03	-0.02	-0.01	0.04	-0.02	-0.02	-0.02	0.02
17	8	0.05	-0.04	-0.03	-0.05	-0.03	-0.02	-0.03	0.02	0.01
18	1	0.09	-0.03	0.22	-0.09	0.00	0.15	-0.06	0.01	-0.02
19	1	-0.01	0.10	-0.01	0.03	-0.12	-0.07	-0.07	0.04	0.02
20	1	-0.01	-0.11	-0.19	0.00	0.08	-0.22	0.01	0.07	0.14
21	8	-0.02	0.04	0.01	-0.01	-0.03	0.00	0.00	-0.02	0.01
22	8	0.05	-0.01	0.03	-0.03	0.01	0.03	-0.02	0.00	0.04
23	1	0.04	0.01	-0.41	0.06	0.15	-0.41	0.08	0.16	-0.64
24	1	-0.54	0.04	0.09	0.37	-0.07	0.02	0.31	-0.02	-0.07

	25			26			27		
	A			A			A		
Frequencies --	596.4792			650.5803			676.2064		
Red. masses --	1.6517			4.5969			4.8299		
Frc consts --	0.3462			1.1464			1.3012		
IR Inten --	104.2863			46.3312			111.0115		
Raman Activ --	2.2073			1.5969			5.6544		
Depolar (P) --	0.7221			0.4556			0.0647		
Depolar (U) --	0.8387			0.6260			0.1216		

Atom	AN	X	Y	Z	X	Y	Z	X	Y	Z
1	8	0.04	-0.01	-0.01	0.00	0.00	0.00	0.00	-0.01	0.00
2	1	-0.03	0.13	-0.01	0.00	-0.01	0.00	0.00	0.05	-0.02
3	1	-0.32	-0.37	0.34	0.03	0.04	-0.03	-0.10	-0.11	0.07
4	6	-0.01	0.03	0.01	0.05	-0.06	0.02	-0.14	-0.07	0.00
5	6	-0.04	-0.01	-0.10	-0.04	-0.14	0.02	-0.07	0.09	0.01
6	6	0.00	0.05	0.03	0.13	-0.05	-0.02	0.02	-0.15	0.02
7	6	0.01	-0.02	0.03	0.01	0.01	0.00	-0.09	-0.03	-0.01
8	6	-0.04	-0.02	-0.09	-0.13	0.06	0.04	-0.07	0.10	-0.03
9	6	-0.02	-0.02	0.03	0.00	-0.17	0.00	0.02	0.12	-0.01
10	6	0.01	0.02	-0.06	0.05	0.19	0.03	0.03	-0.14	-0.02
11	1	0.01	0.06	0.12	0.06	-0.15	-0.09	0.12	-0.04	0.04
12	8	-0.05	-0.01	-0.02	-0.01	0.01	0.00	0.14	-0.08	0.01
13	8	0.05	0.00	-0.01	-0.02	-0.02	0.00	0.03	0.14	-0.01
14	6	-0.03	-0.03	0.05	-0.03	0.09	-0.03	0.14	0.05	0.01
15	6	-0.01	0.02	0.03	-0.12	0.08	0.01	-0.12	0.10	0.00
16	8	0.01	-0.03	0.03	0.02	0.10	0.15	-0.02	-0.05	-0.12

17	8	0.03	0.03	0.01	-0.02	0.02	-0.15	0.01	-0.01	0.10
18	1	0.06	0.01	-0.03	-0.05	0.20	0.01	-0.16	-0.12	-0.01
19	1	0.07	-0.05	-0.06	-0.01	-0.05	0.00	-0.04	0.31	0.02
20	1	-0.01	-0.07	0.24	0.03	-0.07	-0.11	0.15	0.04	0.06
21	8	0.01	0.03	0.01	0.17	0.06	0.03	0.15	0.09	0.03
22	8	0.02	-0.02	0.04	-0.05	-0.14	-0.04	-0.05	-0.12	-0.03
23	1	0.04	0.04	-0.64	-0.32	-0.43	-0.02	-0.33	-0.42	-0.15
24	1	-0.25	0.06	-0.05	-0.03	-0.04	-0.57	-0.01	0.04	0.42

	28	29	30
	A	A	A
Frequencies --	730.1326	747.6678	767.6829
Red. masses --	5.6082	4.5371	5.3482
Frc consts --	1.7615	1.4943	1.8570
IR Inten --	29.9748	27.7035	30.3879
Raman Activ --	0.8547	19.8487	0.9986
Depolar (P) --	0.1366	0.0252	0.2068
Depolar (U) --	0.2403	0.0491	0.3427

Atom	AN	X	Y	Z	X	Y	Z	X	Y	Z
1	8	0.00	0.00	0.01	-0.01	0.01	0.00	0.01	0.00	0.00
2	1	0.02	0.04	-0.03	0.00	-0.08	0.03	-0.01	0.04	0.00
3	1	0.00	-0.01	-0.03	0.13	0.15	-0.10	-0.08	-0.09	0.07
4	6	0.01	0.01	0.04	0.14	0.01	-0.01	-0.06	0.09	0.04
5	6	0.02	0.01	-0.29	0.06	-0.02	0.05	0.03	0.02	-0.03
6	6	-0.02	0.00	-0.13	0.00	-0.01	0.02	-0.08	0.17	0.01
7	6	0.01	0.04	0.33	0.16	0.08	-0.06	-0.06	-0.06	-0.08
8	6	0.00	0.00	0.11	-0.04	-0.06	-0.03	-0.02	-0.05	0.01
9	6	0.09	0.01	0.02	0.09	0.11	-0.04	0.47	-0.10	-0.03
10	6	-0.01	-0.01	0.07	-0.02	-0.05	-0.01	0.03	-0.03	0.04
11	1	-0.02	0.00	0.10	-0.07	-0.10	0.00	-0.01	0.26	-0.23
12	8	-0.01	0.01	-0.09	-0.11	0.17	0.00	0.03	-0.08	0.03
13	8	0.00	-0.02	-0.06	0.00	-0.19	0.03	0.00	0.08	0.01
14	6	0.00	-0.01	-0.14	-0.11	-0.12	0.02	-0.05	-0.11	0.02
15	6	-0.05	-0.04	0.30	-0.08	0.10	-0.02	0.00	0.04	-0.03
16	8	-0.03	-0.06	-0.01	-0.03	0.00	-0.12	-0.12	0.01	0.05
17	8	-0.02	0.05	0.07	-0.01	0.02	0.11	-0.13	0.02	-0.01
18	1	0.00	-0.01	0.49	0.10	-0.07	-0.05	0.27	-0.07	-0.27
19	1	0.02	-0.09	-0.32	0.11	-0.46	0.02	-0.05	0.20	0.08
20	1	-0.01	0.01	0.04	-0.10	-0.17	0.03	-0.05	-0.14	-0.21
21	8	0.03	0.03	-0.09	0.07	0.10	0.04	0.01	0.02	0.02
22	8	-0.02	0.00	-0.11	-0.06	-0.08	-0.01	-0.01	-0.01	0.01
23	1	-0.10	-0.13	0.42	-0.27	-0.31	-0.13	-0.04	-0.05	-0.04
24	1	0.14	0.03	0.04	0.16	0.04	0.41	0.42	-0.06	-0.17

	31	32	33
	A	A	A
Frequencies --	787.9756	806.5394	809.5489
Red. masses --	1.7313	6.4568	5.4793
Frc consts --	0.6334	2.4747	2.1157
IR Inten --	60.8168	7.4905	3.2008
Raman Activ --	0.2061	0.6072	1.3710
Depolar (P) --	0.7500	0.5311	0.4279
Depolar (U) --	0.8571	0.6938	0.5994

Atom	AN	X	Y	Z	X	Y	Z	X	Y	Z
1	8	0.00	0.00	0.00	0.00	0.00	-0.01	0.00	0.00	0.00
2	1	0.00	0.01	0.00	-0.02	-0.04	0.03	0.00	0.03	-0.02
3	1	-0.01	-0.01	0.01	0.00	0.00	0.04	-0.04	-0.04	0.02
4	6	0.00	0.00	-0.04	0.02	-0.04	0.24	0.04	-0.11	-0.06
5	6	0.00	0.00	0.18	-0.01	-0.04	-0.04	-0.03	-0.12	0.00
6	6	0.00	0.01	-0.02	0.07	-0.04	0.02	0.21	-0.09	0.00
7	6	-0.01	-0.01	0.02	-0.02	-0.04	-0.38	-0.08	-0.03	0.12
8	6	-0.01	-0.01	-0.06	-0.04	-0.03	-0.19	-0.11	-0.08	0.06

```
   9   6     0.06  -0.01  -0.03    -0.01   0.05  -0.01     0.07   0.13  -0.04
  10   6     0.01   0.01  -0.11     0.01   0.12   0.02     0.05   0.29   0.00
  11   1     0.00   0.02   0.46     0.01  -0.14  -0.41     0.06  -0.29   0.07
  12   8     0.01  -0.01  -0.01     0.01  -0.01   0.10     0.05  -0.09  -0.02
  13   8     0.00   0.01  -0.01    -0.02   0.03   0.06    -0.04   0.09  -0.03
  14   6    -0.01  -0.01  -0.04    -0.06   0.01  -0.03    -0.22  -0.04   0.01
  15   6     0.00  -0.01   0.05    -0.02  -0.10   0.39     0.06  -0.02  -0.13
  16   8    -0.01   0.03   0.00     0.00   0.00  -0.04    -0.02   0.02  -0.13
  17   8    -0.02  -0.03  -0.03     0.01   0.02   0.05     0.01   0.06   0.13
  18   1     0.02   0.02   0.65    -0.02   0.13  -0.09     0.05   0.29  -0.06
  19   1    -0.01   0.03  -0.01    -0.03   0.09   0.37    -0.09   0.18  -0.08
  20   1    -0.01  -0.02   0.54    -0.03  -0.07   0.26    -0.12  -0.31  -0.12
  21   8     0.00   0.00  -0.02    -0.01   0.00  -0.11    -0.04  -0.06   0.02
  22   8     0.00   0.00  -0.01     0.04   0.04  -0.09     0.07   0.03   0.05
  23   1     0.01   0.01   0.03     0.05   0.02   0.26     0.17   0.15  -0.02
  24   1     0.04  -0.03   0.01     0.00   0.04   0.14     0.10   0.09   0.44
                    34                     35                     36
                     A                      A                      A
Frequencies --    864.6483               910.8725               929.2615
Red. masses --      3.5403                 5.9223                 1.1690
Frc consts  --      1.5595                 2.8951                 0.5948
IR Inten    --      1.2989                18.6974               139.4543
Raman Activ --      0.6053                 3.8827                 0.4488
Depolar (P) --      0.6666                 0.7434                 0.6523
Depolar (U) --      0.8000                 0.8528                 0.7895
 Atom  AN      X      Y      Z        X      Y      Z        X      Y      Z
   1   8     0.00   0.00   0.01     0.01   0.00  -0.01     0.00  -0.01   0.03
   2   1     0.01   0.03  -0.02     0.00  -0.03   0.02     0.02   0.13  -0.07
   3   1     0.00   0.00  -0.02    -0.04  -0.04   0.08     0.02   0.01  -0.13
   4   6    -0.01  -0.01  -0.21     0.22   0.01  -0.01     0.01   0.00   0.00
   5   6     0.00   0.01   0.18     0.28  -0.04   0.01     0.02   0.00   0.00
   6   6     0.00   0.00   0.09    -0.05   0.12   0.00     0.00   0.01   0.01
   7   6     0.00   0.02   0.18    -0.09  -0.08   0.04    -0.01  -0.01  -0.06
   8   6    -0.01   0.00  -0.22     0.23  -0.09  -0.02     0.02  -0.01   0.00
   9   6     0.02   0.00  -0.01    -0.19   0.03   0.02    -0.01   0.00   0.00
  10   6     0.00   0.01   0.05    -0.01   0.02   0.00     0.00   0.00  -0.01
  11   1     0.00  -0.01  -0.24    -0.16  -0.01   0.00    -0.01   0.00  -0.01
  12   8     0.00   0.00  -0.04     0.01  -0.15   0.00     0.00  -0.02   0.03
  13   8     0.01   0.00  -0.02    -0.13   0.15  -0.04    -0.01   0.03   0.06
  14   6     0.00   0.01   0.11    -0.10  -0.10   0.01    -0.01  -0.01   0.00
  15   6    -0.02  -0.04   0.19    -0.02   0.08   0.04     0.00   0.01   0.00
  16   8     0.00   0.02   0.00     0.04   0.00  -0.01     0.00   0.00   0.00
  17   8    -0.01  -0.02  -0.03     0.03  -0.01  -0.01     0.00   0.00   0.00
  18   1    -0.01  -0.01  -0.75     0.34  -0.03  -0.02     0.03   0.00   0.02
  19   1     0.01  -0.03  -0.22    -0.20   0.32   0.37     0.00  -0.08  -0.97
  20   1     0.00   0.00  -0.30    -0.16   0.05  -0.03    -0.01   0.00   0.02
  21   8     0.00   0.01  -0.05     0.02   0.11   0.02     0.00   0.01   0.00
  22   8     0.02   0.01  -0.04    -0.16  -0.07  -0.04    -0.01  -0.01   0.00
  23   1     0.01  -0.01   0.06    -0.29  -0.22  -0.07    -0.02  -0.01  -0.01
  24   1     0.01  -0.02   0.02    -0.03   0.00   0.01     0.00   0.00   0.00
                    37                     38                     39
                     A                      A                      A
Frequencies --    984.4402              1026.6729              1095.5690
Red. masses --      1.4039                 1.3585                 4.2668
Frc consts  --      0.8016                 0.8437                 3.0174
IR Inten    --      0.2146                 1.0517                47.4473
Raman Activ --      0.0296                 0.0499                23.9898
Depolar (P) --      0.3700                 0.5038                 0.1006
Depolar (U) --      0.5402                 0.6700                 0.1828
 Atom  AN      X      Y      Z        X      Y      Z        X      Y      Z
```

1	8	0.00	0.00	0.00	0.00	0.00	0.00	0.00	0.00	0.00
2	1	0.00	0.00	0.00	0.00	0.00	0.00	0.00	0.00	0.00
3	1	0.00	0.00	0.00	0.00	0.00	0.00	0.00	0.00	0.00
4	6	0.00	0.00	0.04	0.00	0.00	0.02	-0.09	0.05	0.00
5	6	0.00	0.00	0.00	0.00	0.00	0.01	0.03	0.24	-0.02
6	6	0.00	0.00	-0.10	0.00	0.00	-0.12	0.25	-0.12	0.01
7	6	0.00	0.00	-0.03	0.00	0.00	-0.02	-0.04	0.01	0.00
8	6	0.00	0.00	-0.04	0.00	0.00	0.00	0.09	0.03	0.00
9	6	0.00	0.00	0.00	0.00	0.00	0.00	0.01	0.05	0.08
10	6	0.00	0.00	-0.04	0.00	0.00	0.12	-0.01	-0.07	0.00
11	1	0.00	0.01	0.57	0.00	0.02	0.67	0.55	0.25	-0.01
12	8	0.00	0.00	0.01	0.00	0.00	0.00	0.00	0.02	0.00
13	8	0.00	0.00	0.00	0.00	0.00	0.00	0.04	-0.04	0.00
14	6	0.00	0.00	0.14	0.00	0.00	-0.06	-0.27	-0.04	0.00
15	6	0.00	-0.01	0.03	0.00	0.00	0.00	0.06	0.01	0.01
16	8	0.00	0.00	0.00	0.00	0.00	0.00	0.00	-0.05	0.07
17	8	0.00	0.00	0.00	0.00	0.00	0.00	-0.02	-0.06	-0.12
18	1	0.00	0.00	0.20	0.00	-0.01	-0.63	-0.03	-0.09	0.00
19	1	0.00	0.00	0.00	0.00	0.00	-0.01	0.02	0.00	0.01
20	1	-0.01	0.01	-0.77	0.00	-0.01	0.34	-0.45	0.36	0.00
21	8	0.00	0.00	-0.01	0.00	0.00	0.00	0.00	0.02	0.00
22	8	0.00	0.00	0.00	0.00	0.00	0.00	-0.06	-0.04	-0.01
23	1	0.00	-0.01	0.00	0.00	0.00	0.00	0.03	0.06	0.01
24	1	0.00	0.00	0.00	0.00	0.00	0.01	-0.01	-0.05	-0.02

	40	41	42
	A	A	A
Frequencies --	1151.5350	1164.9419	1199.2483
Red. masses --	2.5728	4.9639	1.4345
Frc consts --	2.0101	3.9690	1.2156
IR Inten --	54.2140	86.4685	90.1978
Raman Activ --	2.1168	4.9828	5.1184
Depolar (P) --	0.0568	0.2015	0.5074
Depolar (U) --	0.1076	0.3354	0.6732

Atom	AN	X	Y	Z	X	Y	Z	X	Y	Z
1	8	0.00	0.00	0.00	0.00	0.00	0.00	0.00	0.00	0.00
2	1	0.00	-0.01	0.00	-0.01	0.01	0.00	0.00	0.00	0.00
3	1	0.00	0.00	0.00	0.00	-0.01	0.01	0.00	0.00	0.00
4	6	-0.10	-0.04	0.01	-0.19	-0.07	0.00	-0.09	-0.04	0.00
5	6	0.01	0.13	-0.02	-0.07	-0.16	0.02	0.03	-0.01	0.00
6	6	-0.08	-0.06	0.00	0.00	0.04	0.00	-0.05	-0.03	0.00
7	6	-0.03	0.02	0.00	-0.07	0.05	0.00	-0.05	0.02	0.00
8	6	0.07	-0.06	0.00	0.16	-0.12	-0.01	0.02	0.00	0.00
9	6	0.02	0.08	0.07	-0.01	-0.09	-0.09	-0.02	-0.01	-0.01
10	6	0.02	0.16	0.00	-0.02	0.09	0.00	0.09	0.02	0.00
11	1	-0.41	-0.48	0.01	0.29	0.44	-0.01	-0.19	-0.20	0.01
12	8	0.01	0.04	0.00	0.03	0.06	0.00	0.02	0.03	0.00
13	8	0.04	-0.03	0.00	0.08	-0.07	0.01	0.04	-0.04	0.00
14	6	0.07	-0.09	0.01	0.05	0.02	0.00	-0.04	0.04	0.00
15	6	0.06	0.01	0.00	0.23	0.05	0.03	-0.03	0.00	0.00
16	8	0.00	-0.04	0.05	0.00	0.05	-0.06	0.00	0.01	-0.01
17	8	-0.02	-0.04	-0.11	0.02	0.05	0.14	0.00	0.00	0.02
18	1	0.01	0.17	0.00	-0.23	0.13	0.00	0.82	-0.08	0.00
19	1	0.02	0.00	0.00	0.05	0.00	0.00	0.01	0.03	-0.01
20	1	0.27	-0.57	-0.01	-0.04	0.28	0.00	-0.18	0.38	0.01
21	8	0.00	0.05	0.01	-0.02	0.12	0.02	0.00	-0.02	0.00
22	8	-0.05	-0.03	-0.01	-0.18	-0.14	-0.05	0.02	0.02	0.01
23	1	0.03	0.06	0.02	0.26	0.35	0.09	-0.11	-0.11	-0.04
24	1	0.01	-0.01	0.16	-0.03	0.01	-0.22	0.00	-0.01	-0.06

43	44	45
A	A	A

Frequencies -- 1223.1585 1231.2894 1252.5771
Red. masses -- 1.9104 1.7795 1.7619
Frc consts -- 1.6840 1.5895 1.6287
IR Inten -- 327.2955 177.4466 1.7580
Raman Activ -- 14.4886 7.2092 0.4402
Depolar (P) -- 0.2701 0.0786 0.7160
Depolar (U) -- 0.4253 0.1457 0.8345

Atom	AN	X	Y	Z	X	Y	Z	X	Y	Z
1	8	0.00	0.00	0.00	0.00	0.00	0.00	0.00	0.00	0.00
2	1	0.00	0.01	0.00	0.00	0.00	0.00	0.00	0.00	0.00
3	1	0.00	0.00	0.00	0.00	0.00	0.00	0.00	0.00	0.00
4	6	-0.06	0.00	0.00	0.02	0.05	0.00	0.05	0.12	0.00
5	6	0.05	0.04	0.01	0.00	0.14	0.02	-0.12	0.00	0.00
6	6	0.01	-0.01	0.00	0.02	-0.05	0.00	0.05	-0.02	0.00
7	6	-0.05	0.02	0.00	0.03	-0.01	0.00	0.04	-0.02	0.00
8	6	0.11	-0.11	0.00	-0.01	0.05	0.00	0.01	-0.14	0.00
9	6	0.00	-0.03	-0.02	-0.01	-0.07	-0.05	0.04	0.00	0.00
10	6	-0.03	0.07	0.00	0.00	-0.01	0.00	0.01	0.00	0.00
11	1	0.14	0.17	0.00	-0.19	-0.34	0.01	-0.32	-0.53	0.01
12	8	0.01	0.02	0.00	-0.01	-0.01	0.00	-0.02	-0.03	0.00
13	8	0.03	-0.03	0.00	-0.02	0.02	0.00	-0.02	0.02	0.00
14	6	0.01	-0.03	0.00	-0.03	-0.04	0.00	0.06	0.02	0.00
15	6	-0.09	0.00	-0.01	0.01	0.00	0.00	0.05	0.01	0.00
16	8	0.00	0.01	-0.02	0.00	0.02	-0.06	-0.01	0.00	0.01
17	8	0.00	0.00	0.05	0.01	-0.02	0.13	-0.01	0.00	-0.01
18	1	-0.41	0.13	-0.01	0.05	-0.02	0.00	-0.22	0.03	-0.01
19	1	0.00	0.04	-0.01	0.00	-0.01	0.00	-0.02	0.02	0.00
20	1	0.02	-0.04	0.02	0.04	-0.22	0.00	-0.19	0.66	0.00
21	8	-0.01	-0.04	-0.01	0.00	0.01	0.00	-0.01	0.02	0.00
22	8	0.05	0.11	0.03	-0.01	-0.02	-0.01	-0.02	0.01	0.00
23	1	-0.54	-0.55	-0.16	0.12	0.13	0.04	-0.12	-0.12	-0.04
24	1	-0.03	-0.04	-0.26	-0.10	-0.13	-0.82	0.00	0.02	0.08

46 47 48
A A A

Frequencies -- 1313.8896 1341.3875 1385.3437
Red. masses -- 3.4570 5.5469 2.4375
Frc consts -- 3.5161 5.8805 2.7562
IR Inten -- 290.4599 127.7007 93.8642
Raman Activ -- 21.6411 25.5062 5.9275
Depolar (P) -- 0.1412 0.4668 0.1185
Depolar (U) -- 0.2474 0.6365 0.2119

Atom	AN	X	Y	Z	X	Y	Z	X	Y	Z
1	8	0.00	0.01	0.00	0.00	-0.01	0.00	0.00	0.00	0.00
2	1	0.02	-0.07	0.01	-0.02	0.06	-0.01	0.00	0.01	0.00
3	1	0.03	0.05	-0.06	-0.02	-0.03	0.05	0.00	0.00	0.00
4	6	-0.07	-0.21	0.00	0.19	-0.15	0.00	-0.03	0.09	0.00
5	6	-0.15	0.06	0.00	-0.24	0.00	0.00	0.00	0.02	0.00
6	6	0.02	0.05	0.00	0.15	0.18	0.00	0.02	-0.04	0.00
7	6	0.25	-0.06	0.01	-0.21	0.04	-0.01	0.02	0.00	0.00
8	6	0.10	0.05	0.00	0.15	0.24	0.00	0.00	0.10	0.00
9	6	0.02	0.01	0.01	0.03	-0.02	-0.01	-0.04	-0.17	-0.12
10	6	-0.06	0.07	0.00	-0.21	-0.02	0.00	0.01	-0.05	0.00
11	1	0.13	0.20	-0.01	-0.26	-0.37	0.01	-0.07	-0.17	0.00
12	8	-0.02	-0.04	0.00	0.04	0.06	-0.01	-0.01	-0.01	0.00
13	8	-0.11	0.14	-0.01	0.07	-0.11	0.01	0.00	0.01	0.00
14	6	0.03	-0.07	0.00	0.06	-0.19	0.00	-0.06	0.03	0.00
15	6	0.04	0.00	0.01	-0.02	0.00	0.01	0.19	0.01	0.03
16	8	0.00	0.00	0.00	-0.01	0.00	0.00	0.01	0.01	0.04
17	8	0.00	-0.01	0.00	0.00	0.01	0.00	0.01	0.07	0.02
18	1	0.37	0.02	0.00	0.17	-0.08	0.00	0.09	-0.07	0.00

19	1	0.24	-0.61	0.09	-0.23	0.52	-0.07	0.00	0.00	0.00
20	1	-0.06	0.17	0.00	-0.04	0.07	0.01	0.12	-0.43	0.00
21	8	-0.01	-0.02	-0.01	0.01	-0.04	-0.01	-0.03	-0.05	-0.01
22	8	-0.02	0.03	0.00	0.00	0.02	0.00	-0.06	0.04	0.00
23	1	-0.24	-0.22	-0.07	-0.09	-0.07	-0.02	-0.41	-0.37	-0.11
24	1	-0.02	-0.01	-0.05	-0.01	0.02	0.07	0.07	0.13	0.54

	49	50	51
	A	A	A
Frequencies --	1393.7735	1474.7242	1506.0820
Red. masses --	2.9930	1.5411	2.5369
Frc consts --	3.4256	1.9747	3.3904
IR Inten --	93.4967	32.0490	10.5286
Raman Activ --	10.2949	2.8087	0.4914
Depolar (P) --	0.3860	0.7469	0.6945
Depolar (U) --	0.5570	0.8551	0.8197

Atom	AN	X	Y	Z	X	Y	Z	X	Y	Z
1	8	0.00	0.00	0.00	0.01	-0.01	0.00	0.00	0.00	0.00
2	1	0.00	0.02	0.00	-0.04	0.12	-0.01	-0.01	0.03	0.00
3	1	-0.01	-0.01	0.01	-0.02	-0.02	0.10	0.00	0.00	0.02
4	6	0.07	-0.01	0.00	0.01	-0.03	0.00	-0.10	-0.12	0.00
5	6	-0.02	-0.08	0.00	-0.02	0.05	0.00	0.11	0.00	0.00
6	6	-0.03	0.01	0.00	-0.03	-0.05	0.00	-0.01	0.13	0.00
7	6	-0.03	0.00	0.00	0.13	-0.08	0.01	0.05	-0.03	0.00
8	6	-0.12	0.03	0.00	-0.01	-0.04	0.00	-0.09	0.12	0.00
9	6	0.04	0.20	0.13	0.00	-0.02	-0.02	-0.03	-0.01	0.00
10	6	0.01	-0.04	0.00	-0.01	0.03	0.00	0.18	-0.02	0.00
11	1	0.06	0.12	0.00	0.15	0.19	0.00	-0.31	-0.22	0.00
12	8	0.00	0.01	0.00	-0.01	0.06	-0.01	0.01	0.04	0.00
13	8	0.00	-0.02	0.00	-0.06	-0.04	0.00	-0.02	-0.01	0.00
14	6	0.01	0.06	0.00	0.02	0.01	0.00	-0.04	-0.13	0.00
15	6	0.25	0.01	0.03	0.00	0.00	0.00	0.04	0.01	0.01
16	8	-0.01	-0.01	-0.04	0.00	0.00	0.01	0.00	0.00	0.00
17	8	-0.01	-0.08	-0.02	0.00	0.01	0.00	0.00	0.00	0.00
18	1	0.09	-0.05	0.00	0.14	0.02	0.00	-0.69	0.10	0.00
19	1	-0.07	0.14	-0.02	-0.42	0.81	-0.11	-0.08	0.16	-0.02
20	1	0.08	-0.10	0.00	0.01	0.03	0.00	-0.26	0.36	0.00
21	8	-0.04	-0.04	-0.01	0.00	0.01	0.00	0.00	-0.03	-0.01
22	8	-0.07	0.03	0.00	0.00	0.00	0.00	-0.01	0.00	0.00
23	1	-0.45	-0.41	-0.12	0.01	0.00	0.00	-0.04	-0.03	-0.01
24	1	-0.08	-0.14	-0.59	0.00	0.01	0.05	0.01	0.00	0.01

	52	53	54
	A	A	A
Frequencies --	1516.3480	1622.7208	1657.9637
Red. masses --	4.5152	1.1008	7.2488
Frc consts --	6.1168	1.7079	11.7399
IR Inten --	13.3307	136.1542	70.1285
Raman Activ --	14.4089	12.1743	42.4548
Depolar (P) --	0.3591	0.6449	0.7448
Depolar (U) --	0.5284	0.7841	0.8538

Atom	AN	X	Y	Z	X	Y	Z	X	Y	Z
1	8	0.00	0.00	0.00	0.00	-0.06	-0.04	0.00	-0.01	0.00
2	1	0.01	-0.04	0.00	-0.37	0.54	0.09	-0.03	0.05	0.01
3	1	0.01	0.01	-0.04	0.37	0.40	0.51	0.04	0.04	0.04
4	6	0.23	-0.13	0.00	0.00	0.00	0.00	0.24	-0.08	0.00
5	6	0.03	0.26	0.00	0.00	0.00	0.00	-0.34	0.04	0.00
6	6	-0.12	-0.07	0.00	0.00	0.00	0.00	-0.26	-0.08	0.00
7	6	-0.16	0.05	0.00	0.00	0.03	0.00	-0.06	0.00	0.00
8	6	-0.24	-0.08	0.00	0.00	0.00	0.00	0.26	0.03	0.00
9	6	-0.02	-0.11	-0.08	0.00	0.00	0.00	0.04	0.00	0.00
10	6	0.00	0.10	0.00	0.00	0.00	0.00	0.41	-0.05	0.00

11	1	0.26	0.44	-0.01	0.00	-0.01	0.00	0.00	0.30	-0.01
12	8	0.02	-0.01	0.00	0.00	-0.03	0.00	0.01	0.01	0.00
13	8	0.04	0.00	0.00	0.00	0.00	0.00	0.00	0.00	0.00
14	6	0.11	-0.10	0.00	0.00	-0.01	0.00	-0.22	0.13	0.00
15	6	0.11	0.00	0.00	0.00	0.00	0.00	-0.05	0.02	0.00
16	8	0.00	0.00	0.03	0.00	0.00	0.00	-0.01	0.00	0.00
17	8	0.00	0.03	0.02	0.00	0.00	0.00	0.00	0.00	0.00
18	1	0.05	0.12	0.00	0.01	0.00	0.00	-0.50	0.08	0.00
19	1	0.15	-0.29	0.04	0.02	-0.07	0.00	0.02	-0.06	0.01
20	1	-0.12	0.50	-0.01	0.00	0.01	0.00	-0.09	-0.26	0.00
21	8	-0.01	0.00	0.00	0.00	0.00	0.00	0.01	-0.01	0.00
22	8	-0.02	0.00	0.00	0.00	0.00	0.00	0.00	0.00	0.00
23	1	-0.05	-0.05	-0.02	0.00	0.00	0.00	0.02	0.03	0.01
24	1	0.02	0.04	0.15	0.00	0.00	0.00	-0.02	0.00	0.00

	55	56	57
	A	A	A
Frequencies --	1665.2671	1814.3088	1851.9212
Red. masses --	6.5834	7.2789	10.5397
Frc consts --	10.7564	14.1168	21.2972
IR Inten --	7.0804	208.4408	360.4421
Raman Activ --	16.8914	48.8726	45.8119
Depolar (P) --	0.7183	0.2853	0.2876
Depolar (U) --	0.8360	0.4440	0.4467

Atom	AN	X	Y	Z	X	Y	Z	X	Y	Z
1	8	0.00	0.00	0.00	0.00	0.01	0.01	0.00	0.00	0.00
2	1	-0.03	0.04	0.01	0.09	-0.13	-0.03	0.01	-0.01	0.00
3	1	0.03	0.04	0.03	-0.16	-0.22	-0.11	-0.02	-0.02	-0.01
4	6	0.01	-0.30	0.01	-0.03	-0.09	0.00	-0.01	-0.03	0.00
5	6	0.02	0.19	0.00	-0.01	0.03	0.00	0.01	0.06	-0.01
6	6	0.16	0.30	-0.01	-0.01	0.02	0.00	0.01	0.01	0.00
7	6	-0.03	-0.04	0.00	0.17	0.58	-0.05	0.03	0.09	-0.01
8	6	-0.07	-0.29	0.00	0.00	0.00	0.00	0.01	-0.10	0.00
9	6	-0.01	-0.03	-0.04	0.01	-0.01	0.03	0.00	-0.14	0.24
10	6	-0.02	-0.18	0.00	0.03	-0.01	0.00	-0.02	0.00	0.00
11	1	-0.32	-0.32	0.01	-0.04	0.00	0.00	-0.02	-0.02	0.00
12	8	0.02	0.05	0.00	-0.09	-0.34	0.03	-0.01	-0.05	0.00
13	8	0.00	0.00	0.00	0.01	-0.07	0.01	0.00	-0.01	0.00
14	6	-0.09	0.35	-0.01	-0.01	0.02	0.00	0.01	0.02	0.00
15	6	0.01	-0.03	-0.01	0.00	-0.10	-0.02	-0.03	0.67	0.12
16	8	0.00	-0.01	0.02	0.00	0.01	-0.02	0.00	0.09	-0.16
17	8	0.00	0.01	0.01	-0.01	0.00	0.00	0.00	0.00	-0.03
18	1	-0.03	-0.22	0.00	-0.03	0.00	0.00	0.02	-0.01	0.00
19	1	0.02	-0.04	0.00	-0.30	0.52	-0.06	-0.05	0.07	-0.01
20	1	0.23	-0.42	0.00	0.00	-0.02	0.00	0.04	-0.02	0.00
21	8	-0.01	0.04	0.01	0.00	0.06	0.01	0.00	-0.42	-0.08
22	8	0.00	0.00	0.00	0.00	0.01	0.00	-0.01	-0.06	-0.01
23	1	0.00	-0.01	0.00	-0.04	-0.04	-0.01	0.30	0.29	0.09
24	1	0.00	0.01	0.02	0.02	0.00	0.00	-0.01	0.02	0.14

	58	59	60
	A	A	A
Frequencies --	1866.7975	3215.5293	3238.4079
Red. masses --	10.7597	1.0893	1.0928
Frc consts --	22.0925	6.6360	6.7522
IR Inten --	449.1747	4.7027	0.8067
Raman Activ --	41.3596	76.7417	66.9736
Depolar (P) --	0.0595	0.5263	0.4722
Depolar (U) --	0.1122	0.6897	0.6415

Atom	AN	X	Y	Z	X	Y	Z	X	Y	Z
1	8	0.00	0.00	0.00	0.00	0.00	0.00	0.00	0.00	0.00
2	1	-0.01	0.01	0.01	0.00	0.00	0.00	0.00	0.00	0.00

3	1	0.01	0.02	0.02	0.00	0.00	0.00	0.00	0.00	0.00
4	6	0.01	0.01	0.00	0.00	0.00	0.00	0.00	0.00	0.00
5	6	0.00	0.05	-0.02	0.00	0.00	0.00	0.00	0.00	0.00
6	6	0.00	-0.01	0.00	-0.02	0.02	0.00	-0.06	0.05	0.00
7	6	-0.03	-0.08	0.01	0.00	0.00	0.00	0.00	0.00	0.00
8	6	-0.01	0.03	0.00	0.00	0.00	0.00	0.00	0.00	0.00
9	6	0.01	-0.38	0.58	0.00	0.00	0.00	0.00	0.00	0.00
10	6	0.01	0.00	0.00	-0.01	-0.08	0.00	0.01	0.02	0.00
11	1	0.00	-0.01	0.00	0.25	-0.19	0.00	0.70	-0.53	0.01
12	8	0.01	0.05	-0.01	0.00	0.00	0.00	0.00	0.00	0.00
13	8	0.00	0.01	0.00	0.00	0.00	0.00	0.00	0.00	0.00
14	6	-0.01	0.00	0.00	0.02	0.01	0.00	-0.03	-0.01	0.00
15	6	0.02	-0.27	-0.05	0.00	0.00	0.00	0.00	0.00	0.00
16	8	-0.01	0.23	-0.38	0.00	0.00	0.00	0.00	0.00	0.00
17	8	-0.01	0.02	-0.06	0.00	0.00	0.00	0.00	0.00	0.00
18	1	-0.01	0.00	0.00	0.12	0.90	-0.02	-0.03	-0.17	0.00
19	1	0.05	-0.07	0.01	0.00	0.00	0.00	0.00	0.00	0.00
20	1	-0.01	-0.01	0.00	-0.25	-0.10	0.00	0.40	0.16	0.00
21	8	0.00	0.17	0.03	0.00	0.00	0.00	0.00	0.00	0.00
22	8	0.00	0.02	0.00	0.00	0.00	0.00	0.00	0.00	0.00
23	1	-0.12	-0.12	-0.03	0.00	0.00	0.00	0.00	0.00	0.00
24	1	0.05	0.07	0.39	0.00	0.00	0.00	0.00	0.00	0.00

	61	62	63
	A	A	A
Frequencies --	3244.2487	3455.4462	3764.8722
Red. masses --	1.0961	1.0674	1.0550
Frc consts --	6.7972	7.5091	8.8106
IR Inten --	1.9560	1046.3991	266.7669
Raman Activ --	160.5245	202.9629	103.8981
Depolar (P) --	0.1201	0.2479	0.1674
Depolar (U) --	0.2145	0.3973	0.2868

Atom	AN	X	Y	Z	X	Y	Z	X	Y	Z
1	8	0.00	0.00	0.00	0.00	0.00	0.00	-0.03	0.04	0.01
2	1	0.00	0.00	0.00	0.02	0.02	0.02	-0.19	-0.06	-0.17
3	1	0.00	0.00	0.00	-0.06	0.07	0.00	0.70	-0.65	0.05
4	6	0.00	0.00	0.00	0.00	0.00	0.00	0.00	0.00	0.00
5	6	0.00	0.00	0.00	0.00	0.00	0.00	0.00	0.00	0.00
6	6	0.03	-0.02	0.00	0.00	0.00	0.00	0.00	0.00	0.00
7	6	0.00	0.00	0.00	0.00	0.01	0.00	0.00	0.00	0.00
8	6	0.00	0.00	0.00	0.00	0.00	0.00	0.00	0.00	0.00
9	6	0.00	0.00	0.00	0.00	0.00	0.00	0.00	0.00	0.00
10	6	0.00	-0.03	0.00	0.00	0.00	0.00	0.00	0.00	0.00
11	1	-0.28	0.21	0.00	0.00	0.00	0.00	0.00	0.00	0.00
12	8	0.00	0.00	0.00	0.00	0.00	0.00	0.00	0.00	0.00
13	8	0.00	0.00	0.00	-0.06	-0.03	0.00	-0.01	0.00	0.00
14	6	-0.07	-0.03	0.00	0.00	0.00	0.00	0.00	0.00	0.00
15	6	0.00	0.00	0.00	0.00	0.00	0.00	0.00	0.00	0.00
16	8	0.00	0.00	0.00	0.00	0.00	0.00	0.00	0.00	0.00
17	8	0.00	0.00	0.00	0.00	0.00	0.00	0.00	0.00	0.00
18	1	0.05	0.36	-0.01	0.00	0.00	0.00	0.00	0.00	0.00
19	1	0.00	0.00	0.00	0.91	0.39	-0.04	0.09	0.04	0.00
20	1	0.80	0.32	0.00	0.00	0.00	0.00	0.00	0.00	0.00
21	8	0.00	0.00	0.00	0.00	0.00	0.00	0.00	0.00	0.00
22	8	0.00	0.00	0.00	0.00	0.00	0.00	0.00	0.00	0.00
23	1	0.00	0.00	0.00	0.00	0.00	0.00	0.00	0.00	0.00
24	1	0.00	0.00	0.00	0.00	0.00	0.00	0.00	-0.01	0.00

	64	65	66
	A	A	A
Frequencies --	3843.0819	3850.7727	3970.9454
Red. masses --	1.0642	1.0648	1.0735

Frc consts -- 9.2601 9.3025 9.9735
IR Inten -- 100.2021 124.7358 125.3352
Raman Activ -- 120.9480 133.8817 68.3900
Depolar (P) -- 0.2779 0.2384 0.3001
Depolar (U) -- 0.4350 0.3850 0.4617

Atom	AN	X	Y	Z	X	Y	Z	X	Y	Z
1	8	0.00	0.00	0.00	0.00	0.00	0.00	-0.05	-0.01	-0.04
2	1	0.00	0.00	0.00	0.00	0.00	0.00	0.65	0.29	0.65
3	1	0.00	0.00	0.00	0.00	0.00	0.00	0.17	-0.18	-0.01
4	6	0.00	0.00	0.00	0.00	0.00	0.00	0.00	0.00	0.00
5	6	0.00	0.00	0.00	0.00	0.00	0.00	0.00	0.00	0.00
6	6	0.00	0.00	0.00	0.00	0.00	0.00	0.00	0.00	0.00
7	6	0.00	0.00	0.00	0.00	0.00	0.00	0.00	0.00	0.00
8	6	0.00	0.00	0.00	0.00	0.00	0.00	0.00	0.00	0.00
9	6	0.00	0.00	0.00	0.00	0.00	0.00	0.00	0.00	0.00
10	6	0.00	0.00	0.00	0.00	0.00	0.00	0.00	0.00	0.00
11	1	0.00	0.00	0.00	0.00	0.00	0.00	0.00	0.00	0.00
12	8	0.00	0.00	0.00	0.00	0.00	0.00	0.00	0.00	0.00
13	8	0.00	0.00	0.00	0.00	0.00	0.00	0.00	0.00	0.00
14	6	0.00	0.00	0.00	0.00	0.00	0.00	0.00	0.00	0.00
15	6	0.00	0.00	0.00	0.00	0.00	0.00	0.00	0.00	0.00
16	8	0.00	0.00	0.00	0.00	0.00	0.00	0.00	0.00	0.00
17	8	0.01	0.06	-0.01	0.00	0.00	0.00	0.00	0.00	0.00
18	1	0.00	0.00	0.00	0.00	0.00	0.00	0.00	0.00	0.00
19	1	0.00	0.00	0.00	0.00	0.00	0.00	-0.01	0.00	0.00
20	1	0.00	0.00	0.00	0.00	0.00	0.00	0.00	0.00	0.00
21	8	0.00	0.00	0.00	0.00	0.00	0.00	0.00	0.00	0.00
22	8	0.00	0.00	0.00	-0.05	0.04	0.00	0.00	0.00	0.00
23	1	-0.03	0.03	0.00	0.75	-0.66	-0.05	0.00	0.00	0.00
24	1	-0.10	-0.98	0.13	0.00	-0.05	0.01	0.00	0.00	0.00

PYRHEM H2O

Harmonic frequencies (cm**-1), IR intensities (KM/Mole), Raman scattering activities (A**4/AMU), depolarization ratios for plane and unpolarized incident light, reduced masses (AMU), force constants (mDyne/A), and normal coordinates:

	1	2	3
	A	A	A
Frequencies --	18.3350	25.5591	37.1620
Red. masses --	6.6994	8.1349	7.2656
Frc consts --	0.0013	0.0031	0.0059
IR Inten --	1.8844	2.0622	0.8942
Raman Activ --	1.4893	1.2673	2.0050
Depolar (P) --	0.7499	0.7344	0.7443
Depolar (U) --	0.8571	0.8469	0.8534

Atom	AN	X	Y	Z	X	Y	Z	X	Y	Z
1	6	-0.05	0.01	0.00	0.02	0.03	0.02	-0.03	-0.01	0.02
2	6	0.00	0.05	0.00	0.02	0.03	-0.01	-0.02	0.00	0.01
3	6	-0.05	-0.06	0.01	0.02	0.03	0.03	-0.03	-0.02	0.01
4	6	-0.12	0.04	-0.02	0.02	0.03	0.02	-0.05	0.00	0.04
5	6	0.07	0.02	0.01	0.02	0.04	-0.05	-0.01	0.00	-0.01
6	6	0.00	0.12	-0.02	0.02	0.04	-0.01	-0.02	0.01	0.01
7	6	0.01	-0.09	0.02	0.02	0.03	-0.02	-0.02	-0.02	-0.02
8	1	-0.10	-0.09	0.00	0.02	0.03	0.06	-0.04	-0.03	0.02
9	8	-0.12	0.09	0.00	-0.05	0.06	-0.13	-0.11	0.05	-0.07
10	8	-0.17	0.02	-0.04	0.11	-0.02	0.21	0.01	-0.06	0.21
11	6	0.07	-0.06	0.02	0.02	0.04	-0.06	-0.01	-0.02	-0.03
12	6	0.13	0.05	0.02	0.02	0.04	-0.07	0.00	0.00	0.03
13	8	0.00	0.13	-0.02	0.01	0.11	-0.01	-0.02	0.15	-0.02

14	8	0.00	0.13	-0.02	0.03	-0.02	0.01	-0.03	-0.13	0.03
15	1	0.01	-0.15	0.03	0.02	0.03	-0.02	-0.02	-0.03	-0.04
16	1	-0.21	0.04	-0.05	0.10	-0.01	0.19	-0.01	-0.05	0.21
17	1	0.11	-0.08	0.04	0.02	0.04	-0.10	0.00	-0.02	-0.05
18	8	0.14	0.11	0.01	0.00	0.02	0.03	-0.05	-0.01	0.24
19	8	0.18	0.01	0.03	0.06	0.07	-0.21	0.08	0.03	-0.18
20	1	0.22	0.04	0.03	0.05	0.06	-0.21	0.07	0.03	-0.12
21	8	-0.06	0.11	-0.01	-0.39	-0.37	0.00	0.31	0.07	-0.06
22	1	-0.03	0.12	-0.03	-0.23	-0.28	0.03	0.23	0.02	-0.09
23	7	0.00	-0.07	0.00	0.01	0.05	0.03	-0.01	0.12	-0.05
24	1	0.01	0.01	-0.01	0.01	0.09	0.02	0.00	0.21	-0.05
25	7	0.00	-0.02	-0.01	0.03	-0.02	0.04	-0.03	-0.11	-0.01
26	1	0.00	0.11	-0.02	0.03	-0.02	0.02	-0.02	-0.14	0.02
27	6	0.00	-0.17	0.01	0.04	-0.08	0.07	-0.05	-0.21	-0.02
28	1	-0.01	-0.16	0.01	0.05	-0.13	0.08	-0.07	-0.40	0.00
29	6	0.00	-0.32	0.03	0.02	-0.05	0.08	-0.03	-0.04	-0.07
30	1	0.00	-0.46	0.04	0.03	-0.08	0.09	-0.03	-0.06	-0.09
31	6	0.01	-0.25	0.02	0.01	0.03	0.05	0.00	0.17	-0.09
32	1	0.01	-0.31	0.03	0.00	0.07	0.05	0.02	0.35	-0.12
33	1	-0.09	0.11	0.00	-0.34	-0.26	-0.12	0.14	-0.03	0.11

	4	5	6
	A	A	A
Frequencies --	47.0392	50.9376	59.1803
Red. masses --	6.4150	7.5464	4.9898
Frc consts --	0.0084	0.0115	0.0103
IR Inten --	5.5976	3.4451	0.5078
Raman Activ --	3.2536	0.1783	2.4845
Depolar (P) --	0.7475	0.7021	0.7494
Depolar (U) --	0.8555	0.8250	0.8568

Atom	AN	X	Y	Z	X	Y	Z	X	Y	Z
1	6	-0.01	-0.01	0.04	0.01	-0.01	-0.07	-0.01	0.01	0.01
2	6	-0.01	-0.01	-0.04	0.00	-0.01	-0.07	-0.01	0.01	0.03
3	6	0.00	-0.02	0.22	0.01	0.01	-0.12	-0.01	0.00	-0.01
4	6	0.00	-0.01	-0.06	0.01	-0.01	0.04	-0.01	0.01	-0.01
5	6	-0.01	-0.01	0.02	-0.01	-0.01	-0.05	-0.01	0.01	0.02
6	6	-0.01	0.01	-0.09	0.00	0.01	-0.06	-0.01	0.01	0.04
7	6	-0.01	-0.02	0.32	0.00	0.01	-0.16	-0.01	0.00	-0.02
8	1	0.00	-0.02	0.30	0.01	0.01	-0.12	-0.02	0.00	-0.02
9	8	-0.06	-0.02	-0.20	-0.08	0.08	-0.11	-0.05	0.02	-0.08
10	8	0.07	0.00	0.03	0.12	-0.13	0.36	0.02	0.00	0.05
11	6	-0.02	-0.02	0.20	-0.01	0.00	-0.10	0.00	0.00	-0.01
12	6	-0.01	0.00	-0.09	-0.01	-0.02	0.06	0.00	0.01	0.04
13	8	-0.02	0.02	-0.08	-0.02	0.03	-0.06	-0.01	0.03	0.03
14	8	0.01	0.02	-0.08	0.01	0.03	-0.06	-0.01	-0.01	0.04
15	1	-0.01	-0.03	0.47	0.00	0.02	-0.22	-0.01	0.00	-0.03
16	1	0.07	0.00	-0.05	0.10	-0.12	0.43	0.01	0.00	0.03
17	1	-0.02	-0.02	0.26	-0.01	0.01	-0.08	0.00	0.00	-0.02
18	8	0.02	-0.01	-0.20	0.03	0.04	-0.09	-0.03	0.00	0.16
19	8	-0.05	0.00	-0.05	-0.07	-0.09	0.38	0.05	0.03	-0.09
20	1	-0.04	0.00	-0.13	-0.06	-0.08	0.43	0.04	0.03	-0.07
21	8	0.14	0.01	-0.19	0.02	0.05	-0.09	0.14	-0.13	-0.08
22	1	0.13	-0.01	-0.23	0.04	0.04	-0.15	0.10	-0.10	0.04
23	7	-0.04	0.01	0.05	-0.01	0.00	0.00	0.00	-0.17	0.02
24	1	-0.07	0.01	0.01	-0.02	0.00	-0.02	0.02	-0.25	0.05
25	7	0.01	0.03	0.04	0.01	0.06	-0.01	-0.03	0.16	-0.03
26	1	0.00	0.02	-0.05	0.01	0.05	-0.04	-0.01	0.07	0.02
27	6	0.03	0.02	0.09	0.02	0.04	0.01	-0.04	0.28	-0.08
28	1	0.08	0.03	0.09	0.04	0.07	0.01	-0.07	0.53	-0.12
29	6	-0.01	0.00	0.14	0.00	-0.03	0.04	-0.02	0.03	-0.06
30	1	0.00	-0.01	0.18	0.00	-0.05	0.06	-0.02	0.05	-0.09

31	6	-0.06	-0.01	0.11	-0.02	-0.05	0.03	0.01	-0.26	0.00
32	1	-0.10	-0.02	0.12	-0.04	-0.09	0.04	0.04	-0.50	0.03
33	1	0.01	-0.05	-0.09	-0.08	-0.01	0.01	0.03	-0.08	-0.09

	7	8	9
	A	A	A
Frequencies --	80.2923	89.0854	113.8383
Red. masses --	7.6674	6.0257	7.1191
Frc consts --	0.0291	0.0282	0.0544
IR Inten --	3.1125	0.5650	0.5590
Raman Activ --	0.6107	2.0937	0.2363
Depolar (P) --	0.6984	0.7494	0.7482
Depolar (U) --	0.8224	0.8568	0.8560

Atom	AN	X	Y	Z	X	Y	Z	X	Y	Z
1	6	0.03	-0.02	-0.05	-0.02	0.03	0.00	0.04	0.01	0.00
2	6	0.01	-0.03	-0.05	0.00	0.05	-0.01	0.02	0.01	-0.01
3	6	0.03	-0.01	-0.01	-0.02	0.01	0.01	0.04	0.01	0.00
4	6	0.04	-0.03	-0.07	-0.06	0.05	0.00	0.11	-0.02	0.04
5	6	0.00	-0.02	0.01	0.02	0.04	-0.01	0.05	0.00	-0.01
6	6	0.01	-0.04	-0.10	0.00	0.03	-0.01	0.00	0.02	-0.04
7	6	0.01	0.00	0.04	0.00	0.00	0.01	0.05	0.00	0.00
8	1	0.04	0.00	-0.01	-0.04	0.00	0.01	0.03	0.00	0.01
9	8	0.03	-0.03	-0.12	-0.08	0.09	-0.02	0.16	-0.10	0.11
10	8	0.09	-0.03	-0.01	-0.07	0.02	0.04	0.15	0.02	0.01
11	6	0.00	-0.02	0.05	0.02	0.01	0.00	0.05	0.00	0.00
12	6	-0.01	-0.03	0.09	0.04	0.05	-0.01	0.11	0.04	0.02
13	8	0.02	0.02	-0.12	0.00	-0.02	0.00	0.00	-0.02	-0.03
14	8	-0.01	-0.11	-0.10	0.00	0.02	0.00	-0.03	0.05	-0.05
15	1	0.01	0.00	0.07	0.00	-0.02	0.01	0.05	0.00	0.00
16	1	0.10	-0.04	-0.02	-0.10	0.04	0.04	0.22	-0.01	0.06
17	1	-0.01	-0.02	0.09	0.04	0.00	0.00	0.05	-0.01	0.00
18	8	-0.06	-0.05	0.30	0.05	0.07	-0.06	0.11	0.10	0.04
19	8	0.05	-0.01	-0.07	0.04	0.03	0.04	0.18	0.00	0.01
20	1	0.03	-0.02	0.02	0.06	0.04	0.03	0.22	0.03	0.03
21	8	-0.25	0.37	0.02	0.02	-0.04	-0.02	0.03	-0.06	-0.03
22	1	-0.23	0.20	-0.41	0.05	-0.01	0.01	-0.01	-0.01	0.14
23	7	-0.03	-0.11	0.00	0.01	-0.34	0.05	-0.28	-0.03	-0.06
24	1	-0.06	-0.14	-0.05	0.02	-0.40	0.06	-0.33	-0.04	-0.13
25	7	0.05	-0.04	-0.02	0.01	-0.37	0.05	-0.19	-0.05	-0.07
26	1	-0.02	-0.09	-0.09	0.01	-0.14	0.02	-0.01	0.01	-0.09
27	6	0.08	0.08	0.04	0.01	-0.02	0.00	-0.15	-0.02	0.00
28	1	0.15	0.15	0.04	0.02	0.03	-0.01	-0.09	-0.02	0.01
29	6	0.02	0.09	0.10	0.01	0.27	-0.05	-0.23	0.03	0.06
30	1	0.03	0.17	0.16	0.01	0.60	-0.09	-0.22	0.06	0.13
31	6	-0.05	-0.04	0.07	0.01	0.06	-0.01	-0.31	0.02	0.01
32	1	-0.12	-0.07	0.10	0.01	0.16	-0.03	-0.38	0.04	0.03
33	1	-0.07	0.08	0.23	-0.05	-0.01	-0.02	0.06	0.06	-0.16

	10	11	12
	A	A	A
Frequencies --	119.7274	133.0046	152.6129
Red. masses --	6.4523	6.9995	6.2847
Frc consts --	0.0545	0.0730	0.0862
IR Inten --	5.5793	8.7054	8.1280
Raman Activ --	0.3566	0.4360	0.1315
Depolar (P) --	0.5262	0.6842	0.6707
Depolar (U) --	0.6896	0.8125	0.8029

Atom	AN	X	Y	Z	X	Y	Z	X	Y	Z
1	6	-0.01	0.02	-0.02	-0.04	0.00	0.05	-0.01	0.00	0.06
2	6	0.00	0.02	-0.10	-0.03	0.01	0.02	-0.01	-0.01	0.15
3	6	-0.01	0.01	0.05	-0.04	0.00	0.04	-0.01	0.00	-0.05
4	6	-0.04	0.04	0.05	-0.02	-0.01	0.11	-0.02	0.00	-0.05

5	6	-0.01	0.02	-0.07	-0.03	0.01	0.00	0.00	-0.01	0.10
6	6	0.01	0.00	-0.14	-0.02	0.00	0.00	-0.01	-0.01	0.18
7	6	-0.01	0.01	0.06	-0.04	0.01	-0.02	-0.01	0.00	-0.07
8	1	0.00	0.00	0.11	-0.04	0.00	0.07	-0.01	0.01	-0.13
9	8	-0.01	0.09	0.17	0.03	-0.05	0.22	-0.08	-0.01	-0.19
10	8	-0.14	0.00	0.00	-0.07	0.01	0.03	0.05	0.02	0.00
11	6	-0.01	0.02	0.01	-0.03	0.01	-0.03	0.00	0.00	0.01
12	6	-0.03	0.01	0.00	-0.03	0.01	0.05	0.01	0.00	0.00
13	8	0.01	0.10	-0.17	0.02	-0.16	0.01	-0.02	-0.04	0.20
14	8	0.02	-0.14	-0.12	-0.04	0.15	-0.04	-0.02	0.04	0.17
15	1	-0.01	0.01	0.13	-0.04	0.01	-0.05	0.00	0.01	-0.16
16	1	-0.16	0.02	0.07	-0.04	0.00	0.09	0.03	0.02	-0.11
17	1	-0.01	0.01	0.04	-0.03	0.01	-0.06	0.00	0.00	-0.04
18	8	-0.04	0.01	0.04	-0.06	0.02	0.15	0.03	-0.01	-0.09
19	8	-0.04	0.00	0.05	0.00	0.01	-0.01	0.00	0.02	-0.03
20	1	-0.06	-0.01	0.12	0.00	0.01	0.04	0.01	0.03	-0.14
21	8	0.10	-0.16	0.22	-0.03	-0.04	-0.42	0.01	0.05	0.07
22	1	0.14	-0.13	0.25	-0.04	0.07	-0.13	-0.04	-0.04	-0.08
23	7	-0.05	-0.02	-0.13	0.04	0.02	-0.08	-0.09	-0.02	-0.21
24	1	-0.11	-0.02	-0.23	0.01	-0.01	-0.13	-0.16	-0.04	-0.30
25	7	0.12	0.02	-0.16	0.13	-0.02	-0.09	0.11	-0.03	-0.24
26	1	0.01	-0.10	-0.15	-0.06	0.09	-0.08	-0.02	0.01	0.02
27	6	0.19	0.05	0.00	0.18	-0.03	0.01	0.20	-0.01	-0.04
28	1	0.33	0.08	0.01	0.26	-0.06	0.03	0.37	-0.02	-0.02
29	6	0.06	0.01	0.13	0.10	0.00	0.08	0.04	0.01	0.11
30	1	0.07	0.01	0.27	0.11	0.00	0.16	0.05	0.02	0.28
31	6	-0.09	-0.03	0.04	0.01	0.03	0.02	-0.15	0.00	-0.01
32	1	-0.24	-0.06	0.09	-0.07	0.06	0.04	-0.32	0.01	0.05
33	1	0.14	-0.11	0.15	-0.15	0.16	-0.56	0.03	-0.10	0.21

	13	14	15
	A	A	A
Frequencies --	162.6090	170.4141	179.4523
Red. masses --	7.1698	7.6017	5.4787
Frc consts --	0.1117	0.1301	0.1039
IR Inten --	2.7701	2.5910	0.5201
Raman Activ --	1.7756	0.6961	0.7754
Depolar (P) --	0.7491	0.7424	0.7168
Depolar (U) --	0.8565	0.8522	0.8350

Atom	AN	X	Y	Z	X	Y	Z	X	Y	Z
1	6	0.09	0.03	-0.16	-0.11	0.00	-0.15	0.03	0.00	0.14
2	6	0.07	0.00	-0.04	-0.11	0.01	-0.11	0.03	0.01	-0.03
3	6	0.09	0.03	-0.16	-0.11	0.00	-0.08	0.03	-0.02	0.28
4	6	-0.01	0.09	-0.07	0.00	-0.06	-0.08	-0.03	0.03	0.00
5	6	0.05	0.00	0.10	-0.11	0.01	-0.01	0.02	0.02	-0.18
6	6	0.06	-0.09	-0.03	-0.10	0.00	-0.06	0.03	-0.07	-0.02
7	6	0.07	0.03	0.05	-0.11	-0.02	0.07	0.03	0.01	0.01
8	1	0.09	0.04	-0.28	-0.12	0.01	-0.09	0.03	-0.04	0.50
9	8	-0.05	0.29	-0.04	0.03	-0.16	-0.07	-0.09	0.05	-0.15
10	8	-0.13	-0.08	0.06	0.15	-0.03	0.05	-0.02	0.07	-0.05
11	6	0.05	0.00	0.20	-0.11	-0.02	0.10	0.02	0.04	-0.27
12	6	0.00	-0.03	0.05	-0.01	0.08	0.02	0.01	0.01	-0.02
13	8	0.06	-0.32	0.02	-0.13	-0.20	0.00	0.02	-0.24	0.02
14	8	0.07	0.10	-0.05	-0.04	0.20	-0.07	0.04	0.04	-0.03
15	1	0.07	0.04	0.11	-0.11	-0.03	0.16	0.03	0.02	0.04
16	1	-0.24	-0.01	0.16	0.26	-0.09	0.14	-0.07	0.09	-0.22
17	1	0.04	0.00	0.36	-0.10	-0.04	0.21	0.02	0.05	-0.47
18	8	-0.01	-0.12	0.07	-0.01	0.22	0.07	-0.03	0.04	0.12
19	8	-0.04	0.04	-0.07	0.14	0.00	-0.03	0.03	-0.04	0.08
20	1	-0.08	0.01	-0.12	0.23	0.07	-0.01	0.02	-0.04	0.25
21	8	0.01	-0.03	0.03	0.06	-0.05	0.22	0.02	0.00	0.07

22	1	0.07	0.11	0.27	0.11	0.03	0.38	0.08	0.01	0.02
23	7	-0.06	0.06	0.00	0.10	0.03	0.00	-0.02	0.04	0.00
24	1	-0.07	0.00	0.00	0.11	-0.01	0.03	-0.02	-0.01	0.01
25	7	-0.06	0.02	0.00	0.07	0.00	0.01	-0.02	0.02	0.00
26	1	0.09	0.05	-0.03	-0.05	0.13	-0.01	0.04	0.02	-0.01
27	6	-0.06	-0.03	0.01	0.07	-0.03	0.00	-0.03	-0.01	0.00
28	1	-0.06	-0.05	0.01	0.06	-0.05	0.00	-0.03	-0.03	0.00
29	6	-0.06	-0.03	0.01	0.09	-0.01	-0.02	-0.02	-0.02	0.00
30	1	-0.06	-0.07	0.01	0.09	-0.03	-0.03	-0.02	-0.05	0.00
31	6	-0.06	0.02	0.00	0.11	0.02	-0.01	-0.02	0.01	0.00
32	1	-0.06	0.03	0.00	0.12	0.04	-0.02	-0.01	0.01	-0.01
33	1	-0.05	0.19	-0.17	0.05	0.08	0.09	-0.06	-0.03	0.13

	16	17	18
	A	A	A
Frequencies --	199.5587	213.6309	228.2911
Red. masses --	2.2344	1.7702	6.9915
Frc consts --	0.0524	0.0476	0.2147
IR Inten --	27.4594	31.6942	19.1316
Raman Activ --	0.4247	0.4655	1.9438
Depolar (P) --	0.4392	0.5837	0.6914
Depolar (U) --	0.6103	0.7371	0.8175

Atom	AN	X	Y	Z	X	Y	Z	X	Y	Z
1	6	0.01	-0.02	0.00	0.00	0.00	-0.04	-0.03	-0.02	0.18
2	6	0.02	-0.01	-0.02	0.02	0.01	-0.05	0.00	0.00	0.20
3	6	0.01	-0.03	0.03	0.00	-0.01	0.00	-0.03	-0.01	-0.03
4	6	0.01	-0.02	-0.02	0.00	-0.01	0.00	-0.04	-0.01	0.05
5	6	0.02	-0.02	-0.03	0.02	0.00	-0.04	0.02	-0.01	0.21
6	6	0.01	0.08	0.00	0.01	0.06	-0.01	0.02	0.04	-0.08
7	6	0.02	-0.03	0.01	0.01	-0.02	0.03	-0.01	-0.01	-0.18
8	1	0.01	-0.03	0.06	0.00	-0.02	0.02	-0.04	0.00	-0.15
9	8	0.01	-0.03	-0.04	0.02	-0.01	0.04	-0.08	-0.10	-0.07
10	8	0.03	-0.02	-0.01	0.00	-0.02	0.00	0.03	0.06	0.00
11	6	0.02	-0.02	-0.03	0.02	-0.01	0.00	0.02	0.00	0.01
12	6	-0.03	-0.05	0.00	-0.01	-0.02	0.00	0.00	-0.01	0.07
13	8	-0.01	0.11	0.01	-0.04	0.03	0.03	0.26	-0.05	-0.21
14	8	0.04	0.15	0.00	0.06	0.15	0.01	-0.18	0.16	-0.21
15	1	0.02	-0.04	0.03	0.01	-0.03	0.07	-0.01	-0.01	-0.44
16	1	0.04	-0.03	-0.01	0.01	-0.02	0.02	0.02	0.05	-0.16
17	1	0.03	-0.01	-0.04	0.03	-0.01	0.02	0.03	0.00	-0.07
18	8	-0.04	-0.11	0.05	-0.03	-0.06	0.04	0.02	-0.08	-0.01
19	8	-0.08	-0.02	0.00	-0.05	0.00	0.00	-0.05	0.06	-0.03
20	1	-0.12	-0.05	0.03	-0.08	-0.03	0.04	-0.08	0.04	-0.19
21	8	0.01	-0.01	0.04	0.01	-0.02	0.01	0.06	-0.05	0.09
22	1	0.13	0.29	0.61	-0.07	-0.30	-0.59	-0.04	-0.16	-0.07
23	7	-0.01	-0.03	-0.01	-0.01	-0.01	-0.01	0.01	0.00	0.05
24	1	-0.02	-0.01	-0.02	-0.01	-0.01	-0.01	0.01	0.00	0.04
25	7	0.00	-0.03	-0.01	-0.01	-0.02	-0.01	-0.01	-0.01	0.05
26	1	0.05	0.13	0.00	0.06	0.12	0.03	-0.19	0.11	-0.22
27	6	0.01	-0.01	0.00	-0.01	-0.01	-0.01	-0.01	0.00	0.05
28	1	0.02	-0.01	0.00	-0.01	-0.01	-0.01	-0.02	-0.01	0.05
29	6	0.00	0.02	0.00	-0.01	0.01	-0.01	0.00	0.01	0.03
30	1	0.00	0.04	0.01	-0.01	0.02	-0.01	0.00	0.02	0.02
31	6	-0.02	0.01	0.00	-0.01	0.00	-0.01	0.01	0.02	0.04
32	1	-0.03	0.02	0.00	-0.01	0.01	-0.01	0.01	0.02	0.04
33	1	-0.18	0.47	-0.35	0.20	-0.50	0.42	0.11	-0.22	0.24

	19	20	21
	A	A	A
Frequencies --	279.3894	306.5745	322.7540
Red. masses --	6.3331	2.5196	1.7798
Frc consts --	0.2913	0.1395	0.1092

IR Inten -- 3.8274 1.8379 1.5651
Raman Activ -- 0.8482 1.5572 1.4850
Depolar (P) -- 0.7495 0.7080 0.4994
Depolar (U) -- 0.8568 0.8290 0.6661

Atom	AN	X	Y	Z	X	Y	Z	X	Y	Z
1	6	0.15	-0.09	0.06	0.01	-0.05	0.01	0.01	0.02	0.04
2	6	0.01	-0.17	0.09	0.05	-0.01	0.00	-0.03	-0.02	-0.01
3	6	0.14	0.06	0.04	0.01	-0.01	-0.01	0.01	0.03	-0.01
4	6	0.10	-0.06	-0.01	-0.03	-0.08	0.01	0.01	0.05	0.04
5	6	-0.15	-0.10	0.04	-0.01	0.04	0.02	-0.01	-0.04	0.02
6	6	0.01	-0.05	0.03	0.09	-0.01	-0.01	-0.05	-0.01	-0.03
7	6	-0.01	0.15	0.01	-0.03	0.02	-0.02	0.01	0.02	-0.03
8	1	0.26	0.13	0.03	0.05	0.02	-0.02	0.00	0.02	-0.03
9	8	0.05	0.14	-0.03	-0.03	-0.11	0.00	0.01	0.06	0.02
10	8	-0.09	-0.19	-0.06	-0.05	-0.10	-0.02	0.03	0.10	-0.01
11	6	-0.15	0.05	-0.01	-0.01	0.04	0.02	-0.02	-0.01	0.03
12	6	-0.11	-0.06	-0.01	-0.03	0.07	0.01	0.01	-0.06	0.00
13	8	0.04	0.12	-0.02	0.08	0.00	0.00	-0.08	0.00	-0.01
14	8	-0.03	0.14	-0.02	0.14	-0.01	0.02	-0.01	0.02	-0.02
15	1	-0.02	0.31	-0.02	-0.03	0.03	-0.05	0.01	0.04	-0.08
16	1	-0.29	-0.08	-0.11	-0.08	-0.08	-0.01	0.06	0.08	-0.07
17	1	-0.25	0.10	-0.07	0.00	0.03	0.04	-0.05	0.00	0.05
18	8	-0.08	0.18	-0.02	-0.03	0.11	0.00	0.01	-0.06	-0.01
19	8	0.11	-0.19	-0.04	-0.04	0.09	0.00	0.05	-0.09	-0.01
20	1	0.28	-0.06	-0.08	-0.05	0.08	-0.03	0.10	-0.06	-0.02
21	8	0.01	-0.01	0.01	-0.01	0.00	0.00	0.00	-0.02	0.00
22	1	-0.17	-0.11	-0.04	0.58	0.20	-0.18	0.60	0.18	-0.21
23	7	0.00	-0.03	0.01	-0.02	0.00	0.00	0.01	0.00	0.00
24	1	0.00	0.02	0.00	-0.03	0.00	-0.01	0.01	0.00	0.00
25	7	0.00	-0.02	0.01	-0.01	0.00	0.00	0.01	0.00	0.00
26	1	-0.04	0.17	-0.05	0.16	-0.01	0.05	-0.02	0.01	0.02
27	6	0.00	-0.01	0.01	-0.02	0.00	-0.01	0.01	0.00	0.00
28	1	0.00	-0.01	0.01	-0.02	0.00	-0.01	0.01	0.00	0.00
29	6	0.00	0.01	0.00	-0.02	0.00	-0.01	0.01	0.00	0.00
30	1	0.00	0.03	0.00	-0.02	0.00	0.00	0.01	0.00	0.00
31	6	0.00	0.00	0.00	-0.03	0.00	-0.01	0.01	0.00	0.00
32	1	0.00	0.02	0.00	-0.03	0.00	-0.01	0.01	0.00	0.00
33	1	0.24	-0.08	0.00	-0.61	0.01	0.21	-0.63	-0.02	0.25

22 23 24
A A A
Frequencies -- 377.5132 380.2575 406.5875
Red. masses -- 5.4231 4.5273 7.4407
Frc consts -- 0.4554 0.3857 0.7247
IR Inten -- 13.6809 37.6769 14.6823
Raman Activ -- 3.6100 0.2077 2.8652
Depolar (P) -- 0.1736 0.7495 0.7497
Depolar (U) -- 0.2958 0.8568 0.8569

Atom	AN	X	Y	Z	X	Y	Z	X	Y	Z
1	6	-0.03	0.00	0.08	-0.02	0.02	-0.14	-0.07	0.04	-0.04
2	6	0.04	0.00	-0.03	0.00	0.02	0.09	0.00	0.08	-0.02
3	6	-0.04	-0.02	-0.11	-0.04	-0.05	-0.10	-0.05	0.28	-0.05
4	6	-0.09	0.07	0.09	0.01	0.04	-0.06	-0.10	-0.08	0.00
5	6	-0.05	-0.01	-0.06	0.00	0.01	-0.16	0.07	0.05	-0.11
6	6	0.14	-0.01	-0.02	0.02	0.02	0.14	0.00	0.09	0.04
7	6	-0.07	-0.02	-0.02	-0.02	-0.09	0.17	-0.01	0.28	0.09
8	1	-0.04	0.00	-0.23	-0.08	-0.07	-0.16	0.05	0.34	-0.08
9	8	-0.11	-0.05	0.01	0.05	0.06	0.03	-0.09	-0.22	-0.02
10	8	0.05	0.24	0.04	0.04	0.02	0.00	-0.12	-0.10	0.02
11	6	-0.05	-0.02	0.12	0.01	-0.05	-0.07	0.04	0.27	0.03
12	6	-0.10	-0.06	-0.06	-0.05	0.00	-0.09	0.10	-0.09	-0.06

13	8	0.13	0.00	-0.01	0.26	0.01	0.01	0.08	-0.03	0.02
14	8	0.24	0.00	0.03	-0.18	0.01	0.04	-0.07	-0.03	0.02
15	1	-0.07	-0.01	-0.04	-0.02	-0.14	0.43	-0.01	0.19	0.20
16	1	0.18	0.15	-0.14	0.12	-0.02	0.15	-0.16	-0.08	0.09
17	1	-0.04	-0.05	0.27	0.05	-0.07	-0.09	-0.06	0.31	0.10
18	8	-0.11	0.06	0.00	-0.07	0.05	0.01	0.08	-0.21	0.00
19	8	0.05	-0.20	-0.02	-0.01	-0.07	0.01	0.14	-0.15	0.01
20	1	0.19	-0.10	0.12	-0.01	-0.06	0.20	0.18	-0.12	0.21
21	8	-0.01	0.02	-0.01	0.01	-0.01	0.00	0.01	0.00	0.00
22	1	-0.35	-0.09	0.13	0.29	0.07	-0.12	-0.10	-0.02	0.07
23	7	-0.02	0.00	0.00	0.00	0.00	0.00	0.00	0.01	0.00
24	1	-0.04	0.00	-0.02	0.00	0.01	0.00	0.00	-0.01	0.00
25	7	-0.01	0.00	0.00	-0.01	0.00	0.00	0.00	0.00	0.00
26	1	0.26	0.02	0.08	-0.19	-0.01	-0.06	-0.07	-0.06	-0.02
27	6	-0.02	0.00	-0.01	0.00	0.00	0.01	0.00	0.00	0.00
28	1	-0.02	0.00	-0.01	0.01	0.01	0.01	0.00	0.00	0.00
29	6	-0.03	0.00	-0.01	0.00	0.00	0.02	0.00	0.00	0.01
30	1	-0.03	0.00	0.00	0.00	0.00	0.02	0.00	0.00	0.01
31	6	-0.03	0.00	-0.01	-0.01	0.00	0.01	0.00	0.00	0.00
32	1	-0.03	0.00	-0.01	-0.02	0.00	0.01	0.00	0.00	0.01
33	1	0.37	0.04	-0.18	-0.44	-0.06	0.23	-0.11	-0.02	0.07

	25	26	27
	A	A	A
Frequencies --	435.5577	489.7577	542.9081
Red. masses --	3.7376	1.1063	5.6903
Frc consts --	0.4178	0.1564	0.9882
IR Inten --	14.9364	225.2207	14.2879
Raman Activ --	3.7616	0.2824	2.3025
Depolar (P) --	0.2696	0.5947	0.2831
Depolar (U) --	0.4246	0.7459	0.4412

Atom	AN	X	Y	Z	X	Y	Z	X	Y	Z
1	6	-0.08	0.04	-0.19	-0.01	0.00	0.04	-0.02	0.09	0.01
2	6	-0.02	0.02	0.01	-0.01	0.01	0.02	0.05	0.17	-0.03
3	6	-0.10	0.00	0.14	-0.01	0.00	-0.01	-0.03	-0.02	0.13
4	6	-0.04	0.04	-0.13	0.01	0.00	0.00	0.03	0.02	-0.07
5	6	-0.05	-0.01	0.21	0.00	0.01	-0.02	0.13	0.09	0.10
6	6	0.05	0.02	0.00	-0.02	0.01	0.00	-0.01	0.16	-0.02
7	6	-0.10	0.01	-0.01	0.00	0.00	0.00	0.13	-0.05	-0.15
8	1	-0.10	-0.01	0.37	-0.01	0.00	-0.06	-0.14	-0.09	0.22
9	8	0.04	0.05	0.04	0.00	0.00	-0.01	0.07	-0.03	-0.03
10	8	0.02	0.01	0.01	0.00	-0.01	0.01	-0.04	-0.09	-0.01
11	6	-0.06	0.03	-0.13	0.01	0.00	0.01	0.23	0.01	0.12
12	6	-0.02	-0.05	0.12	0.00	0.01	-0.02	-0.17	-0.03	-0.04
13	8	0.09	0.00	-0.02	0.00	-0.01	0.00	-0.04	-0.05	0.04
14	8	0.10	-0.01	0.03	-0.01	0.00	0.00	-0.06	-0.05	-0.01
15	1	-0.10	-0.01	-0.03	0.00	-0.01	-0.01	0.14	-0.18	-0.40
16	1	0.21	-0.08	0.36	-0.04	0.00	-0.02	-0.07	-0.06	0.33
17	1	-0.05	0.05	-0.36	0.01	0.00	0.03	0.32	-0.04	0.15
18	8	0.03	-0.07	-0.05	0.00	0.00	0.00	-0.18	0.12	-0.02
19	8	0.05	-0.06	-0.01	0.00	0.00	0.00	-0.02	-0.20	-0.04
20	1	0.21	0.05	-0.38	-0.01	-0.01	0.09	0.21	-0.03	0.02
21	8	-0.02	0.00	0.00	-0.04	-0.01	0.03	0.01	0.00	0.00
22	1	0.18	0.04	-0.11	0.61	0.13	-0.35	-0.04	-0.01	0.03
23	7	-0.01	0.00	0.00	0.00	0.00	0.00	0.00	0.01	0.00
24	1	-0.02	0.00	-0.01	0.00	0.00	0.00	0.01	-0.04	0.01
25	7	0.00	0.00	0.00	0.00	0.00	0.00	0.00	0.00	0.00
26	1	0.11	-0.01	0.07	-0.02	-0.04	0.04	-0.07	-0.13	-0.04
27	6	0.00	0.00	0.00	0.00	0.00	0.00	0.00	0.01	0.00
28	1	-0.01	0.00	0.00	0.00	0.00	0.00	0.00	0.01	0.00
29	6	-0.01	0.00	0.00	0.00	0.00	0.00	0.00	0.00	0.00

30	1	-0.01	0.00	0.00	0.00	0.00	0.00	0.00	-0.01	0.00
31	6	-0.01	0.00	0.00	0.00	0.00	0.00	0.01	0.00	0.00
32	1	-0.02	0.00	0.00	0.00	-0.01	0.00	0.01	-0.01	0.00
33	1	0.15	0.04	-0.10	0.57	0.13	-0.36	-0.09	-0.02	0.06

28 29 30
A A A
Frequencies -- 553.9933 585.0921 599.3549
Red. masses -- 3.6821 1.4401 2.7931
Frc consts -- 0.6658 0.2905 0.5912
IR Inten -- 40.3853 116.5593 25.1692
Raman Activ -- 6.3673 0.6860 0.4074
Depolar (P) -- 0.2228 0.6648 0.7216
Depolar (U) -- 0.3645 0.7986 0.8383

Atom	AN	X	Y	Z	X	Y	Z	X	Y	Z
1	6	0.09	-0.02	-0.12	0.03	-0.02	-0.04	-0.02	0.02	0.09
2	6	0.07	-0.05	0.00	0.01	-0.04	-0.02	0.00	0.02	0.21
3	6	0.17	0.00	-0.06	0.05	-0.01	0.06	-0.03	0.02	-0.10
4	6	-0.12	0.02	0.01	-0.05	0.01	-0.04	0.00	0.02	-0.04
5	6	0.04	-0.03	0.06	-0.01	-0.03	0.01	0.02	0.02	0.10
6	6	-0.01	-0.05	0.01	0.00	-0.04	0.02	0.00	-0.01	-0.08
7	6	0.16	0.02	0.09	0.02	0.03	-0.06	0.01	-0.02	0.15
8	1	0.19	0.02	-0.03	0.07	0.00	0.15	-0.06	0.03	-0.43
9	8	-0.11	-0.14	0.02	-0.03	-0.01	0.01	0.02	0.00	-0.01
10	8	-0.05	0.12	0.04	-0.02	0.04	-0.03	-0.02	-0.02	-0.01
11	6	0.07	0.02	-0.09	-0.02	-0.01	0.05	0.04	0.02	-0.11
12	6	-0.05	-0.03	0.08	0.02	0.00	-0.04	-0.01	0.01	-0.04
13	8	-0.07	0.02	0.02	0.01	0.01	0.01	-0.11	-0.01	-0.03
14	8	-0.06	0.01	-0.04	-0.03	0.01	0.00	0.10	-0.01	-0.05
15	1	0.16	0.06	0.24	0.02	0.08	-0.13	0.01	-0.07	0.11
16	1	0.18	0.00	0.21	0.35	-0.13	0.55	0.11	-0.07	0.36
17	1	0.03	0.05	-0.19	-0.05	0.00	0.10	0.07	0.04	-0.44
18	8	-0.03	0.09	0.00	0.02	0.01	0.00	-0.03	0.01	0.00
19	8	-0.05	-0.03	0.01	0.00	0.03	-0.04	0.01	-0.02	-0.03
20	1	0.11	0.07	-0.67	-0.23	-0.12	0.62	-0.08	-0.07	0.51
21	8	-0.01	0.00	0.01	0.00	0.00	0.00	0.01	0.00	0.00
22	1	0.16	0.03	-0.09	0.02	0.00	-0.02	-0.09	-0.02	0.05
23	7	0.00	0.00	0.00	0.00	0.00	0.00	0.00	0.00	0.00
24	1	0.01	0.01	0.01	0.00	0.01	0.00	-0.01	-0.01	0.00
25	7	0.00	0.00	0.00	0.00	0.00	0.00	0.01	0.00	0.00
26	1	-0.07	0.02	-0.07	-0.03	0.02	-0.02	0.11	0.01	0.03
27	6	0.00	0.00	0.00	0.00	0.00	0.00	0.00	0.00	0.00
28	1	0.00	0.00	0.00	0.00	0.00	0.00	0.00	0.00	-0.01
29	6	0.00	0.00	0.00	0.00	0.00	0.00	0.00	0.00	0.00
30	1	0.00	0.00	0.00	0.00	0.00	0.00	0.00	0.00	0.00
31	6	0.01	0.00	0.00	0.00	0.00	0.00	0.00	0.00	0.00
32	1	0.01	0.00	0.00	0.00	0.00	0.00	0.01	0.00	0.00
33	1	0.15	0.03	-0.08	0.01	0.00	-0.01	-0.05	-0.01	0.03

31 32 33
A A A
Frequencies -- 617.3343 627.6689 655.1367
Red. masses -- 1.4303 3.4666 4.6503
Frc consts -- 0.3212 0.8047 1.1760
IR Inten -- 15.5382 12.1212 129.4001
Raman Activ -- 3.7552 0.1964 0.2982
Depolar (P) -- 0.7326 0.7498 0.7215
Depolar (U) -- 0.8457 0.8570 0.8382

Atom	AN	X	Y	Z	X	Y	Z	X	Y	Z
1	6	-0.01	-0.01	0.11	0.00	0.00	0.00	-0.10	-0.12	0.00
2	6	-0.01	0.00	0.01	0.00	0.00	0.00	0.01	-0.07	0.02
3	6	-0.02	0.00	-0.01	0.00	0.00	0.00	-0.14	0.10	0.01

4	6	0.01	0.00	-0.02	0.00	0.00	0.00	-0.06	-0.15	0.00
5	6	0.00	0.01	-0.10	0.00	0.00	0.00	0.11	-0.10	0.00
6	6	0.00	0.00	0.00	0.00	0.00	0.00	0.00	0.03	-0.01
7	6	-0.02	0.00	-0.01	0.00	0.00	0.00	-0.01	0.05	0.01
8	1	-0.02	0.01	-0.12	0.01	0.00	0.00	-0.07	0.14	-0.01
9	8	0.01	0.03	-0.02	0.00	0.00	0.00	-0.09	0.16	0.08
10	8	-0.02	-0.01	-0.06	0.00	0.00	0.00	0.14	-0.02	-0.07
11	6	-0.01	0.00	0.03	0.00	0.00	0.00	0.13	0.12	0.01
12	6	0.01	0.01	-0.01	0.00	0.00	0.00	0.07	-0.13	-0.01
13	8	0.00	0.00	-0.01	0.00	0.00	0.00	-0.01	-0.02	0.00
14	8	0.01	0.00	0.00	0.00	0.00	0.00	0.00	-0.02	-0.01
15	1	-0.02	0.00	-0.04	0.00	0.01	0.01	0.00	-0.16	-0.03
16	1	0.33	-0.17	0.66	-0.01	0.00	-0.01	0.41	-0.20	-0.34
17	1	-0.02	-0.01	0.10	-0.01	0.00	0.00	0.07	0.15	-0.01
18	8	0.01	-0.02	0.02	0.00	0.00	0.00	0.08	0.17	0.05
19	8	-0.01	0.01	0.05	0.00	0.00	0.00	-0.14	-0.04	-0.04
20	1	0.16	0.11	-0.57	-0.01	0.00	0.02	-0.43	-0.27	-0.22
21	8	0.00	0.00	0.00	0.00	0.00	0.00	0.00	0.00	0.00
22	1	-0.02	-0.01	0.00	-0.01	0.00	0.00	0.00	0.00	0.00
23	7	0.00	0.00	0.00	0.00	0.24	-0.04	0.00	0.00	0.00
24	1	0.00	-0.01	0.00	0.00	-0.11	0.02	0.00	0.01	0.00
25	7	0.00	0.00	0.00	0.00	-0.07	0.01	0.00	0.00	0.00
26	1	0.01	0.01	0.01	0.00	-0.03	0.00	0.00	-0.01	0.00
27	6	0.00	0.00	0.00	0.00	-0.12	0.02	0.00	0.00	0.00
28	1	0.00	0.00	0.00	0.00	-0.35	0.05	0.00	0.01	0.00
29	6	0.00	0.00	0.00	0.00	0.24	-0.04	0.00	0.00	0.00
30	1	0.00	0.00	0.00	0.00	0.37	-0.06	0.00	0.00	0.00
31	6	0.00	0.00	0.00	0.00	-0.27	0.04	0.00	0.00	0.00
32	1	0.00	-0.01	0.00	0.00	-0.71	0.11	0.00	0.00	0.00
33	1	0.00	-0.01	0.01	-0.01	0.00	0.01	0.04	0.01	-0.03

	34	35	36
	A	A	A
Frequencies --	671.9190	673.1302	735.1766
Red. masses --	5.1876	4.7117	6.1087
Frc consts --	1.3799	1.2579	1.9453
IR Inten --	0.6950	10.5498	34.7723
Raman Activ --	0.7358	5.0386	0.0776
Depolar (P) --	0.5418	0.1263	0.7089
Depolar (U) --	0.7028	0.2242	0.8297

Atom	AN	X	Y	Z	X	Y	Z	X	Y	Z
1	6	0.02	-0.03	0.00	0.04	-0.13	-0.02	0.04	-0.02	0.12
2	6	0.03	0.00	0.00	0.16	0.01	0.00	-0.01	0.06	-0.27
3	6	0.02	-0.03	-0.01	0.03	-0.12	-0.02	0.06	-0.03	-0.12
4	6	-0.01	-0.03	0.01	-0.07	-0.12	0.01	0.10	-0.07	0.22
5	6	0.00	0.02	0.01	0.03	0.14	0.02	-0.05	-0.02	0.09
6	6	0.02	0.02	0.00	0.09	0.00	0.01	-0.02	0.33	-0.02
7	6	-0.03	0.00	0.01	-0.15	-0.01	0.00	-0.01	-0.01	0.09
8	1	0.05	-0.01	-0.01	0.18	-0.02	0.01	0.06	-0.03	-0.12
9	8	-0.02	0.02	0.01	-0.09	0.10	0.07	-0.05	0.04	-0.06
10	8	0.02	0.00	-0.01	0.11	-0.01	-0.06	-0.01	-0.01	-0.10
11	6	-0.01	0.02	0.00	0.02	0.12	0.01	-0.07	-0.05	-0.12
12	6	-0.02	0.02	0.01	-0.09	0.12	0.00	-0.05	-0.06	0.25
13	8	-0.01	0.01	0.02	-0.08	0.02	0.10	0.06	-0.07	0.03
14	8	-0.02	0.00	-0.02	-0.08	-0.02	-0.11	-0.05	-0.05	0.06
15	1	-0.03	0.02	0.01	-0.15	-0.01	0.01	-0.01	0.08	0.30
16	1	0.09	-0.04	-0.03	0.40	-0.19	-0.23	0.26	-0.14	0.34
17	1	0.02	0.00	-0.01	0.18	0.04	-0.01	-0.07	-0.05	-0.08
18	8	-0.02	-0.03	-0.01	-0.10	-0.12	-0.05	0.04	0.02	-0.07
19	8	0.03	0.01	0.00	0.12	0.03	0.04	0.00	-0.01	-0.09
20	1	0.07	0.05	0.05	0.43	0.26	0.16	-0.16	-0.11	0.33

21	8	0.00	0.00	0.00	0.00	0.00	0.00	0.00	0.00	0.00
22	1	0.00	0.00	0.00	0.05	0.01	-0.02	-0.01	-0.01	0.00
23	7	0.00	0.25	-0.04	0.00	-0.05	0.01	0.00	-0.01	0.00
24	1	0.01	-0.17	0.03	0.02	0.04	0.02	0.01	-0.14	0.02
25	7	0.00	-0.37	0.06	-0.01	0.07	-0.01	0.00	0.03	-0.01
26	1	-0.01	-0.18	-0.03	-0.08	-0.02	-0.24	-0.05	-0.20	0.04
27	6	0.00	0.30	-0.05	0.00	-0.05	0.02	0.00	-0.02	0.00
28	1	0.00	0.70	-0.11	0.00	-0.13	0.03	0.00	-0.02	0.01
29	6	0.00	-0.18	0.03	0.01	0.03	0.00	0.00	0.00	0.00
30	1	0.00	-0.27	0.04	0.01	0.05	-0.01	0.00	0.04	0.00
31	6	0.00	-0.02	0.00	0.01	0.00	0.00	0.00	0.01	0.00
32	1	0.00	0.00	0.00	0.01	0.00	0.00	0.00	0.02	0.00
33	1	0.01	0.00	-0.01	0.04	0.02	-0.03	0.02	0.01	-0.01

	37	38	39
	A	A	A
Frequencies --	745.7333	766.0880	768.3737
Red. masses --	5.0787	2.5731	1.4034
Frc consts --	1.6641	0.8898	0.4882
IR Inten --	8.8557	62.8615	34.7169
Raman Activ --	17.4790	0.7763	0.2782
Depolar (P) --	0.0287	0.4559	0.3893
Depolar (U) --	0.0557	0.6263	0.5604

Atom	AN	X	Y	Z	X	Y	Z	X	Y	Z
1	6	-0.04	0.01	-0.03	0.04	-0.01	-0.02	-0.02	0.00	0.00
2	6	-0.14	-0.01	0.00	-0.01	0.01	0.04	0.00	0.00	-0.01
3	6	0.02	0.11	0.01	0.10	-0.03	0.04	-0.04	0.01	-0.01
4	6	-0.05	-0.13	0.06	-0.08	0.00	-0.10	0.03	0.00	0.04
5	6	-0.02	-0.01	0.03	-0.04	-0.02	0.01	0.02	0.01	-0.01
6	6	-0.16	-0.06	-0.01	-0.02	0.22	-0.04	0.01	-0.09	0.02
7	6	0.26	0.01	0.00	0.02	0.02	0.03	-0.01	-0.01	-0.01
8	1	-0.09	0.03	0.06	0.12	0.00	-0.21	-0.05	0.00	0.10
9	8	-0.11	0.06	0.05	-0.02	0.00	0.05	0.01	0.00	-0.02
10	8	0.08	-0.05	-0.07	0.04	-0.01	0.02	-0.01	0.01	-0.01
11	6	0.07	-0.08	-0.01	-0.09	-0.06	0.04	0.04	0.02	-0.01
12	6	-0.09	0.11	-0.02	0.05	0.02	-0.13	-0.02	-0.01	0.05
13	8	0.06	-0.01	-0.17	0.00	-0.05	0.00	0.00	0.02	0.00
14	8	0.06	0.04	0.17	0.01	-0.04	0.02	0.00	0.02	-0.01
15	1	0.27	-0.03	0.04	0.02	0.22	-0.33	-0.01	-0.09	0.14
16	1	0.35	-0.21	-0.21	0.03	-0.02	-0.18	-0.02	0.01	0.07
17	1	-0.02	-0.03	0.00	-0.13	-0.01	-0.24	0.05	0.01	0.10
18	8	-0.12	-0.07	-0.04	0.01	-0.01	0.04	0.00	0.00	-0.02
19	8	0.10	0.07	0.04	-0.02	0.00	0.04	0.01	0.00	-0.01
20	1	0.37	0.28	0.17	0.00	0.00	-0.14	0.00	0.00	0.05
21	8	0.00	0.00	-0.01	0.00	0.00	0.00	0.00	0.00	0.00
22	1	-0.07	-0.01	0.03	-0.03	-0.01	0.02	0.01	0.00	-0.01
23	7	0.00	0.00	-0.01	0.00	0.02	0.00	0.00	0.03	0.00
24	1	-0.01	0.00	-0.03	0.00	-0.28	0.04	0.00	-0.18	0.03
25	7	0.01	0.00	0.00	0.00	0.04	-0.01	0.00	0.02	0.00
26	1	0.05	0.10	0.35	0.01	-0.10	0.04	0.00	0.06	-0.02
27	6	0.00	0.00	-0.01	0.00	-0.04	0.00	0.00	-0.04	0.01
28	1	0.00	0.01	-0.01	0.00	0.15	-0.02	0.00	0.24	-0.04
29	6	-0.01	0.00	0.00	0.00	-0.06	0.01	0.00	-0.09	0.01
30	1	0.00	0.00	0.01	0.00	0.56	-0.08	0.00	0.78	-0.11
31	6	-0.01	0.00	0.00	0.00	-0.03	0.00	0.00	-0.06	0.01
32	1	-0.02	0.00	0.00	0.00	0.29	-0.04	0.00	0.42	-0.07
33	1	-0.01	0.00	0.01	-0.03	0.00	0.01	0.01	0.00	-0.01

	40	41	42
	A	A	A
Frequencies --	794.8206	809.1634	813.7251
Red. masses --	1.8306	5.8056	1.2235

Frc consts -- 0.6814 2.2396 0.4773
IR Inten -- 42.4888 12.4725 80.0501
Raman Activ -- 0.9458 0.2163 0.1002
Depolar (P) -- 0.6736 0.7432 0.6123
Depolar (U) -- 0.8050 0.8527 0.7596

Atom	AN	X	Y	Z	X	Y	Z	X	Y	Z
1	6	0.01	0.00	-0.05	0.00	0.00	0.19	0.00	0.00	-0.02
2	6	0.00	0.00	0.19	0.00	0.00	0.02	0.00	0.01	0.01
3	6	0.02	-0.01	-0.03	0.03	0.01	0.02	0.01	0.00	-0.01
4	6	0.00	-0.01	0.02	-0.18	0.06	-0.34	0.01	-0.01	0.04
5	6	-0.01	0.00	-0.04	-0.01	0.00	-0.20	0.00	0.00	0.02
6	6	-0.01	0.11	-0.04	0.01	0.02	0.00	0.00	0.03	-0.01
7	6	0.00	0.02	-0.11	0.08	0.01	-0.01	0.00	0.00	-0.01
8	1	0.04	-0.05	0.50	0.00	0.02	-0.29	0.01	-0.01	0.07
9	8	-0.01	0.00	0.00	0.03	0.00	0.11	-0.01	0.00	-0.01
10	8	0.00	0.00	-0.01	0.06	-0.04	0.07	0.00	0.00	-0.01
11	6	-0.02	-0.01	-0.03	-0.01	-0.02	-0.02	-0.01	-0.01	0.00
12	6	0.00	0.00	0.01	-0.10	-0.05	0.35	0.01	0.01	-0.04
13	8	-0.03	-0.03	-0.01	0.00	-0.01	0.01	0.00	-0.02	0.00
14	8	0.03	-0.02	-0.02	0.00	0.00	-0.01	0.00	0.00	0.00
15	1	0.00	-0.01	0.64	0.08	0.05	0.04	-0.01	0.01	0.05
16	1	0.02	-0.01	0.00	-0.06	0.01	-0.26	0.02	-0.01	0.03
17	1	-0.04	-0.05	0.47	-0.06	-0.03	0.33	-0.01	-0.01	0.00
18	8	0.01	0.00	0.00	0.02	0.00	-0.10	0.00	0.00	0.01
19	8	0.00	0.00	0.00	0.03	0.03	-0.08	-0.01	-0.01	0.01
20	1	-0.01	0.00	-0.01	-0.03	-0.01	0.21	0.00	0.00	-0.03
21	8	0.00	0.00	0.00	0.00	0.00	0.00	0.00	0.00	0.00
22	1	-0.02	0.00	0.01	-0.01	0.00	0.02	0.00	0.00	0.00
23	7	0.00	0.00	0.00	0.00	-0.01	0.00	0.00	-0.02	0.00
24	1	-0.01	-0.15	0.02	0.00	0.46	-0.07	0.00	0.91	-0.14
25	7	0.00	0.01	0.00	0.00	-0.01	0.00	0.00	-0.03	0.00
26	1	0.04	-0.04	0.03	0.01	-0.07	0.00	0.00	-0.09	0.01
27	6	0.00	-0.01	0.00	0.00	0.02	0.00	0.00	0.04	-0.01
28	1	0.00	-0.01	0.00	0.00	-0.03	0.00	0.00	-0.06	0.01
29	6	0.00	0.00	0.00	0.00	0.01	0.00	0.00	0.02	0.00
30	1	0.00	-0.04	0.01	0.00	0.07	-0.01	0.00	0.12	-0.02
31	6	0.00	0.02	0.00	0.00	-0.05	0.01	0.00	-0.09	0.01
32	1	0.00	-0.05	0.01	0.00	0.16	-0.02	0.00	0.31	-0.05
33	1	-0.01	0.00	0.00	-0.05	0.00	0.02	0.00	0.00	0.00

43 44 45
A A A
Frequencies -- 833.9881 865.2331 887.4566
Red. masses -- 6.1752 3.8645 1.2025
Frc consts -- 2.5306 1.7046 0.5580
IR Inten -- 12.0325 1.2872 12.4111
Raman Activ -- 4.9415 0.7337 0.2984
Depolar (P) -- 0.0515 0.7401 0.7500
Depolar (U) -- 0.0980 0.8507 0.8571

Atom	AN	X	Y	Z	X	Y	Z	X	Y	Z
1	6	-0.10	0.08	-0.01	0.00	0.00	0.22	0.00	0.00	0.00
2	6	-0.06	0.00	0.00	0.00	0.03	-0.20	0.00	-0.01	0.00
3	6	-0.12	0.22	0.02	0.01	0.01	-0.11	0.00	0.00	0.00
4	6	0.01	-0.04	0.05	-0.08	0.03	-0.17	0.00	0.00	0.00
5	6	-0.09	-0.09	0.00	-0.01	0.00	0.22	0.00	0.00	0.00
6	6	0.25	0.01	0.04	0.01	-0.06	0.03	0.00	0.00	0.00
7	6	0.24	0.01	0.00	0.01	0.01	-0.05	0.00	0.00	0.00
8	1	-0.34	0.08	0.00	0.01	-0.03	0.29	0.00	0.00	0.00
9	8	-0.03	0.02	0.00	0.02	0.00	0.05	0.00	0.00	0.00
10	8	0.01	-0.03	-0.03	0.03	-0.03	0.02	0.00	0.00	0.00
11	6	-0.09	-0.22	-0.02	-0.01	0.00	-0.10	0.00	0.00	0.00

12	6	-0.01	0.03	-0.03	0.05	0.03	-0.19	0.00	0.00	0.00
13	8	0.00	0.05	0.24	0.02	0.02	0.01	0.00	0.01	0.00
14	8	0.01	-0.04	-0.26	-0.03	0.01	0.01	0.00	0.00	0.00
15	1	0.24	0.01	-0.01	0.00	-0.04	0.76	0.00	-0.01	0.01
16	1	0.07	-0.06	-0.03	-0.01	-0.01	-0.06	-0.01	0.00	0.00
17	1	-0.31	-0.12	-0.01	-0.02	-0.03	0.27	0.00	0.00	0.00
18	8	-0.03	-0.02	0.00	-0.01	0.00	0.05	0.00	0.00	0.00
19	8	0.02	0.04	0.02	-0.02	-0.03	0.03	0.00	0.00	0.00
20	1	0.06	0.07	0.03	0.00	-0.01	-0.06	0.00	0.00	0.00
21	8	0.00	0.00	0.00	0.00	0.00	0.00	0.00	0.00	0.00
22	1	0.05	0.01	-0.03	0.00	0.00	0.00	0.00	0.00	0.00
23	7	0.00	0.00	0.01	0.00	0.00	0.00	0.00	0.02	0.00
24	1	0.02	-0.05	0.05	0.01	-0.02	0.01	0.01	-0.38	0.06
25	7	-0.01	0.00	0.00	0.00	0.00	0.00	0.00	0.01	0.00
26	1	0.03	-0.10	-0.50	-0.03	0.03	-0.03	0.00	0.00	0.00
27	6	-0.01	0.00	0.01	0.00	0.00	0.00	0.00	0.06	-0.01
28	1	-0.01	0.01	0.01	0.00	0.02	0.00	0.01	-0.46	0.07
29	6	0.01	0.00	0.00	0.00	0.00	0.00	0.00	0.05	-0.01
30	1	0.00	0.00	-0.02	0.00	0.01	-0.01	0.00	-0.34	0.04
31	6	0.01	0.01	0.00	0.00	0.01	0.00	0.00	-0.10	0.02
32	1	0.01	-0.02	0.00	0.00	-0.03	0.01	-0.01	0.70	-0.10
33	1	0.03	0.00	-0.02	-0.01	0.00	0.01	0.00	0.00	0.00

	46	47	48
	A	A	A
Frequencies --	905.9519	921.9482	941.8188
Red. masses --	6.3828	1.3426	6.3162
Frc consts --	3.0865	0.6724	3.3010
IR Inten --	6.2833	9.2884	3.9464
Raman Activ --	3.9095	0.0956	5.2660
Depolar (P) --	0.7499	0.7430	0.7423
Depolar (U) --	0.8571	0.8525	0.8521

Atom	AN	X	Y	Z	X	Y	Z	X	Y	Z
1	6	0.05	0.25	-0.02	0.00	0.00	0.00	0.00	0.00	0.00
2	6	-0.01	0.30	0.06	0.00	0.00	0.00	0.00	0.00	0.00
3	6	0.12	-0.08	0.01	0.00	0.00	0.00	0.00	0.00	0.00
4	6	-0.05	-0.06	0.08	0.00	0.00	0.00	0.00	0.00	0.00
5	6	-0.07	0.25	-0.01	0.00	0.00	0.00	0.00	0.00	0.00
6	6	0.01	-0.21	0.03	0.00	0.00	0.00	0.00	0.00	-0.01
7	6	0.00	-0.02	-0.01	0.00	0.00	0.00	0.00	0.00	0.00
8	1	-0.03	-0.17	-0.04	0.00	0.00	0.00	0.00	0.00	0.00
9	8	-0.12	0.00	0.04	0.00	0.00	0.00	0.00	0.00	0.00
10	8	0.09	-0.14	-0.09	0.00	0.00	0.00	0.00	0.00	0.00
11	6	-0.12	-0.09	0.01	0.00	0.00	0.00	0.00	0.00	0.00
12	6	0.08	-0.04	0.05	0.00	0.00	0.00	0.00	0.00	0.00
13	8	-0.02	0.04	-0.02	0.00	0.00	0.00	0.00	0.00	-0.01
14	8	0.01	0.03	-0.02	0.00	0.00	0.00	0.01	0.00	0.02
15	1	-0.02	0.36	-0.01	0.00	0.00	0.00	0.00	0.00	0.00
16	1	0.27	-0.25	-0.19	0.00	0.00	0.00	0.00	0.00	0.00
17	1	0.04	-0.16	-0.03	0.00	0.00	0.00	0.00	0.00	0.00
18	8	0.12	0.02	0.03	0.00	0.00	0.00	0.00	0.00	0.00
19	8	-0.09	-0.16	-0.06	0.00	0.00	0.00	0.00	0.00	0.00
20	1	-0.26	-0.29	-0.11	0.00	0.00	0.00	0.00	0.00	0.00
21	8	0.00	0.00	0.00	0.00	0.00	0.00	0.00	0.00	0.00
22	1	0.01	0.01	0.00	0.00	0.00	0.00	0.00	0.00	0.00
23	7	0.00	0.00	0.00	0.00	-0.01	0.00	-0.18	0.00	0.00
24	1	-0.01	-0.06	0.01	0.00	0.00	0.00	-0.03	0.04	0.23
25	7	0.00	0.00	0.00	0.00	-0.01	0.00	-0.15	0.01	0.05
26	1	0.01	0.10	-0.01	0.00	-0.06	0.01	0.01	0.00	-0.01
27	6	0.00	0.00	0.00	0.00	0.13	-0.02	0.02	0.05	0.32
28	1	0.00	-0.01	0.00	0.00	-0.82	0.12	-0.12	0.04	0.31

29	6	0.00	0.01	0.00	0.00	-0.10	0.01	0.42	-0.01	-0.04
30	1	0.00	-0.04	0.01	0.00	0.42	-0.06	0.43	0.00	0.00
31	6	0.00	-0.01	0.00	0.00	0.05	-0.01	-0.07	-0.05	-0.35
32	1	0.01	0.06	-0.01	0.01	-0.31	0.05	-0.28	-0.05	-0.30
33	1	0.02	0.01	-0.01	0.00	0.00	0.00	0.00	0.00	0.00

	49	50	51
	A	A	A
Frequencies --	960.2324	983.1945	1023.8086
Red. masses --	3.1185	1.3973	1.3531
Frc consts --	1.6941	0.7958	0.8357
IR Inten --	38.0605	0.1171	1.7155
Raman Activ --	2.3770	0.0449	0.0413
Depolar (P) --	0.3528	0.2882	0.2550
Depolar (U) --	0.5216	0.4474	0.4063

Atom	AN	X	Y	Z	X	Y	Z	X	Y	Z
1	6	0.00	0.00	0.00	0.00	0.00	0.04	0.00	0.00	-0.01
2	6	0.00	0.00	-0.01	0.00	0.00	0.00	0.00	0.00	-0.02
3	6	0.00	0.00	0.00	0.00	0.02	-0.13	0.00	-0.01	0.08
4	6	0.00	0.00	0.00	-0.01	0.01	-0.02	0.00	0.00	0.01
5	6	0.00	0.00	0.00	0.00	0.00	-0.04	0.00	0.00	-0.01
6	6	0.00	0.00	0.00	0.00	0.00	0.00	0.00	0.00	0.00
7	6	0.00	0.00	0.00	0.00	0.00	0.01	0.00	0.01	-0.12
8	1	0.00	0.00	0.01	0.02	-0.05	0.73	-0.02	0.03	-0.45
9	8	0.00	0.00	0.00	0.00	0.00	0.01	0.00	0.00	0.00
10	8	0.00	0.00	0.00	0.01	-0.01	0.00	0.00	0.00	0.00
11	6	0.00	0.00	0.00	0.00	-0.02	0.11	0.00	-0.01	0.10
12	6	0.00	0.00	0.00	-0.01	-0.01	0.03	0.00	0.00	0.01
13	8	0.01	0.00	0.01	0.00	0.00	0.00	0.00	0.00	0.00
14	8	-0.02	0.00	-0.01	0.00	0.00	0.00	0.00	0.00	0.00
15	1	0.00	0.00	0.00	0.00	0.00	-0.07	0.00	-0.06	0.66
16	1	0.00	0.00	0.00	-0.01	0.00	0.01	0.01	0.00	0.00
17	1	0.00	0.00	0.00	0.01	0.05	-0.65	0.01	0.05	-0.57
18	8	0.00	0.00	0.00	0.00	0.00	-0.01	0.00	0.00	0.00
19	8	0.00	0.00	0.00	0.00	0.00	0.00	0.00	0.00	0.00
20	1	0.00	0.00	0.00	-0.01	0.00	0.00	-0.01	0.00	0.00
21	8	0.00	0.00	0.00	0.00	0.00	0.00	0.00	0.00	0.00
22	1	0.00	0.00	0.00	0.00	0.00	0.00	0.00	0.00	0.00
23	7	-0.16	0.03	0.16	0.00	0.00	0.00	0.00	0.00	0.00
24	1	-0.33	-0.03	-0.04	0.00	0.00	0.00	0.00	0.00	0.00
25	7	0.20	0.02	0.13	0.00	0.00	0.00	0.00	0.00	0.00
26	1	0.00	0.00	-0.03	0.00	-0.01	0.00	0.00	0.01	0.00
27	6	0.11	-0.02	-0.14	0.00	0.00	0.00	0.00	0.00	0.00
28	1	-0.27	-0.03	-0.20	0.00	0.00	0.00	0.00	0.00	0.00
29	6	-0.02	-0.01	-0.07	0.00	0.00	0.00	0.00	0.00	0.00
30	1	0.05	0.06	0.56	0.00	0.00	0.00	0.00	0.00	0.00
31	6	-0.11	-0.02	-0.12	0.00	0.00	0.00	0.00	0.00	0.00
32	1	0.44	-0.02	-0.29	0.00	0.00	0.00	0.00	0.00	0.00
33	1	0.00	0.00	0.00	0.00	0.00	0.00	0.00	0.00	0.00

	52	53	54
	A	A	A
Frequencies --	1033.0952	1071.6360	1078.9726
Red. masses --	1.0870	1.3832	1.5098
Frc consts --	0.6835	0.9359	1.0356
IR Inten --	96.4999	11.4722	94.6032
Raman Activ --	0.2378	2.7042	1.3611
Depolar (P) --	0.6946	0.6495	0.7336
Depolar (U) --	0.8198	0.7875	0.8463

Atom	AN	X	Y	Z	X	Y	Z	X	Y	Z
1	6	0.00	0.00	0.00	0.00	0.00	0.00	0.00	0.00	0.00
2	6	0.00	0.00	0.00	0.00	0.00	0.00	0.01	0.00	0.00

3	6	0.00	0.00	0.00	0.00	0.00	0.00	0.00	0.01	0.00
4	6	0.00	0.00	0.00	0.00	0.00	0.00	0.00	0.00	0.00
5	6	0.00	0.00	0.00	0.00	0.00	0.00	0.00	0.01	0.00
6	6	0.00	0.03	-0.01	0.00	0.00	0.00	0.00	0.00	0.00
7	6	0.00	0.00	0.00	0.00	0.00	0.00	0.00	0.00	0.00
8	1	0.00	0.00	0.01	0.00	0.00	0.00	0.01	0.02	0.00
9	8	0.00	0.00	0.00	0.00	0.00	0.00	0.00	0.00	0.00
10	8	0.00	0.00	0.00	0.00	0.00	0.00	0.00	0.00	0.00
11	6	0.00	0.00	0.00	0.00	0.00	0.00	0.00	-0.01	0.00
12	6	0.00	0.00	0.00	0.00	0.00	0.00	0.00	0.00	0.00
13	8	0.00	-0.01	0.01	0.00	0.00	0.00	0.00	0.00	-0.01
14	8	0.00	-0.06	0.01	0.00	0.00	0.01	0.00	0.00	0.01
15	1	0.00	0.00	-0.01	0.00	0.00	0.00	0.00	0.00	0.00
16	1	0.00	0.00	0.00	0.00	0.00	0.00	0.00	0.00	0.00
17	1	0.00	0.00	0.00	0.00	0.00	0.00	0.01	-0.01	0.00
18	8	0.00	0.00	0.00	0.00	0.00	0.00	0.00	0.00	0.00
19	8	0.00	0.00	0.00	0.00	0.00	0.00	0.00	0.00	0.00
20	1	0.01	0.01	0.01	0.00	0.00	0.00	0.01	0.00	0.00
21	8	0.00	0.00	0.00	0.00	0.00	0.00	0.00	0.00	0.00
22	1	0.03	0.00	-0.02	0.00	0.00	0.00	0.00	0.00	0.00
23	7	0.00	0.00	0.00	-0.08	0.01	0.06	0.01	0.00	0.03
24	1	0.00	0.05	-0.01	-0.18	-0.01	-0.05	0.08	0.02	0.12
25	7	0.00	-0.03	0.01	0.02	-0.01	-0.04	-0.10	-0.02	-0.11
26	1	-0.02	0.98	-0.14	0.01	0.01	-0.04	0.00	0.00	0.00
27	6	0.00	0.01	0.00	0.07	0.01	0.08	-0.02	0.00	0.02
28	1	0.00	-0.04	0.01	0.53	0.03	0.14	0.25	0.01	0.05
29	6	0.00	0.00	0.00	-0.06	-0.01	-0.06	-0.03	0.01	0.08
30	1	0.00	0.03	0.00	-0.13	-0.10	-0.68	0.01	0.08	0.58
31	6	0.00	0.00	0.00	0.01	0.00	0.01	0.07	-0.01	-0.04
32	1	0.00	0.01	0.00	0.37	-0.01	-0.10	0.69	-0.03	-0.23
33	1	0.01	0.01	-0.01	0.00	0.00	0.00	0.00	0.00	0.00

	55	56	57
	A	A	A
Frequencies --	1102.2668	1153.2260	1178.1302
Red. masses --	3.9008	5.2867	1.8073
Frc consts --	2.7924	4.1425	1.4780
IR Inten --	28.4775	41.1193	65.5582
Raman Activ --	25.4420	5.7932	16.1640
Depolar (P) --	0.0767	0.0702	0.1362
Depolar (U) --	0.1425	0.1312	0.2397

Atom	AN	X	Y	Z	X	Y	Z	X	Y	Z
1	6	0.04	-0.09	-0.01	-0.11	-0.22	0.00	0.00	0.01	0.00
2	6	0.21	0.01	0.01	0.04	-0.02	0.00	-0.04	0.00	0.00
3	6	-0.08	0.25	0.03	-0.06	-0.11	-0.02	0.02	0.01	0.00
4	6	0.01	-0.05	-0.03	0.06	-0.13	-0.05	0.00	0.00	0.00
5	6	0.04	0.10	0.01	-0.13	0.20	0.00	0.00	-0.01	0.00
6	6	0.01	-0.01	-0.04	0.02	0.00	-0.02	-0.01	0.00	0.01
7	6	-0.08	-0.01	0.00	0.24	0.01	0.00	-0.02	-0.01	0.00
8	1	0.35	0.54	0.02	-0.40	-0.35	0.00	0.11	0.07	0.00
9	8	0.02	0.00	-0.01	0.09	0.04	-0.04	0.00	0.00	0.00
10	8	-0.04	0.05	0.03	-0.10	0.11	0.08	0.00	0.00	0.00
11	6	-0.06	-0.26	-0.03	-0.07	0.11	0.02	0.01	0.00	0.00
12	6	0.01	0.07	0.02	0.06	0.17	0.04	0.00	0.01	0.00
13	8	-0.04	-0.01	-0.06	-0.01	0.00	-0.01	0.01	0.00	0.01
14	8	-0.05	0.01	0.09	-0.02	0.00	0.02	0.01	0.00	-0.03
15	1	-0.09	-0.05	0.00	0.26	0.03	0.00	-0.02	-0.04	0.00
16	1	0.05	-0.01	-0.03	0.13	-0.02	-0.08	0.01	-0.01	0.00
17	1	0.30	-0.45	-0.03	-0.34	0.27	0.00	0.07	-0.03	0.00
18	8	0.02	0.00	0.01	0.11	-0.03	0.03	0.00	0.00	0.00
19	8	-0.05	-0.06	-0.02	-0.11	-0.14	-0.06	-0.01	0.00	0.00

20	1	0.08	0.03	0.02	0.22	0.10	0.08	0.03	0.02	0.01
21	8	0.00	0.00	0.00	0.00	0.00	0.00	0.00	0.00	0.00
22	1	0.00	0.00	-0.01	-0.01	-0.01	0.00	0.00	0.00	0.01
23	7	0.00	0.00	0.00	0.01	0.00	0.01	0.11	0.00	0.02
24	1	-0.01	0.00	0.00	0.04	0.01	0.06	0.36	0.06	0.35
25	7	0.01	0.00	0.01	0.00	0.00	0.01	-0.02	0.01	0.07
26	1	-0.04	0.01	0.07	0.00	-0.01	0.01	0.01	0.01	0.07
27	6	0.01	0.00	-0.01	0.02	0.00	-0.01	0.08	0.00	0.01
28	1	-0.01	0.00	-0.01	0.06	0.00	0.00	0.65	0.02	0.09
29	6	0.00	0.00	-0.01	-0.01	0.00	-0.01	-0.12	-0.01	-0.06
30	1	0.00	-0.01	-0.06	-0.01	0.00	0.00	-0.10	0.04	0.25
31	6	-0.01	0.00	0.01	-0.02	0.00	-0.01	-0.10	-0.02	-0.11
32	1	-0.08	0.00	0.03	-0.08	0.00	0.01	-0.38	-0.01	-0.05
33	1	-0.01	0.01	-0.01	-0.02	0.01	0.00	0.00	-0.01	0.00

	58	59	60
	A	A	A
Frequencies --	1181.6654	1190.1389	1212.0644
Red. masses --	2.2735	1.8810	1.6135
Frc consts --	1.8704	1.5697	1.3966
IR Inten --	71.0474	232.7069	391.1369
Raman Activ --	1.8015	1.9928	9.1245
Depolar (P) --	0.4843	0.5419	0.6876
Depolar (U) --	0.6526	0.7029	0.8149

Atom	AN	X	Y	Z	X	Y	Z	X	Y	Z
1	6	0.02	0.01	0.00	-0.04	-0.04	0.01	-0.04	-0.03	0.00
2	6	0.20	0.03	0.01	-0.09	0.06	0.00	0.00	-0.04	-0.01
3	6	-0.09	-0.02	-0.01	0.00	-0.04	-0.01	-0.02	-0.02	0.00
4	6	-0.01	0.01	0.01	0.03	-0.10	-0.04	-0.01	0.07	0.02
5	6	0.03	-0.02	0.00	0.02	0.00	0.00	0.08	-0.06	0.00
6	6	0.05	-0.01	-0.05	-0.02	-0.01	0.03	0.00	-0.01	0.00
7	6	0.04	0.04	0.01	-0.02	0.07	0.01	-0.02	0.05	0.00
8	1	-0.61	-0.35	0.00	0.04	-0.03	0.01	-0.14	-0.10	-0.02
9	8	0.00	0.00	0.00	0.05	0.02	-0.02	-0.03	0.00	0.02
10	8	0.01	-0.01	-0.01	-0.08	0.06	0.06	0.07	-0.03	-0.04
11	6	-0.06	-0.02	0.00	0.06	-0.05	-0.01	0.02	-0.03	0.00
12	6	-0.03	-0.10	-0.02	-0.01	-0.05	-0.01	0.01	0.07	0.02
13	8	-0.04	-0.01	-0.05	0.02	0.01	0.02	0.00	0.00	0.00
14	8	-0.05	0.01	0.08	0.02	0.00	-0.04	0.00	0.00	0.00
15	1	0.02	0.41	0.03	-0.05	0.74	0.06	-0.05	0.65	0.06
16	1	0.00	-0.01	-0.01	0.26	-0.14	-0.17	-0.32	0.20	0.21
17	1	-0.28	0.08	0.01	0.41	-0.23	-0.01	0.12	-0.08	-0.02
18	8	-0.04	0.01	-0.01	-0.03	0.00	-0.01	0.03	0.00	0.01
19	8	0.07	0.07	0.03	0.04	0.03	0.02	-0.08	-0.04	-0.03
20	1	-0.24	-0.16	-0.10	-0.16	-0.11	-0.07	0.40	0.32	0.16
21	8	0.00	0.00	0.00	0.00	0.00	0.00	0.00	0.00	0.00
22	1	0.01	0.00	-0.01	-0.01	0.00	0.00	0.00	0.00	0.00
23	7	0.02	0.00	0.02	0.00	0.00	-0.01	0.00	0.00	0.00
24	1	0.07	0.01	0.08	-0.01	0.00	-0.02	0.00	0.00	0.00
25	7	-0.01	0.00	0.01	0.00	0.00	0.00	0.00	0.00	0.00
26	1	-0.05	0.02	0.04	0.02	0.02	-0.01	0.00	-0.01	0.00
27	6	0.02	0.00	-0.01	-0.01	0.00	0.01	0.00	0.00	0.00
28	1	0.15	0.00	0.00	-0.04	0.00	0.00	0.00	0.00	0.00
29	6	-0.02	0.00	-0.02	0.00	0.00	0.00	0.00	0.00	0.00
30	1	-0.01	0.01	0.06	0.00	0.00	-0.01	0.00	0.00	0.00
31	6	-0.02	0.00	-0.02	0.00	0.00	0.00	0.00	0.00	0.00
32	1	-0.12	0.00	0.01	0.04	0.00	-0.01	0.00	0.00	0.00
33	1	0.01	0.01	-0.01	-0.01	-0.01	0.01	0.01	0.00	0.00

	61	62	63
	A	A	A
Frequencies --	1217.1969	1237.2319	1263.3396

Red. masses -- 3.9350 1.7320 1.7329
Frc consts -- 3.4349 1.5621 1.6296
IR Inten -- 48.0871 21.6943 1.0907
Raman Activ -- 40.3397 23.8231 1.5723
Depolar (P) -- 0.0625 0.1129 0.6621
Depolar (U) -- 0.1177 0.2030 0.7967

Atom	AN	X	Y	Z	X	Y	Z	X	Y	Z
1	6	0.00	0.02	0.00	0.09	0.12	0.01	0.13	0.01	0.00
2	6	-0.03	0.00	0.00	0.00	-0.01	0.00	-0.01	-0.11	-0.01
3	6	0.01	0.00	0.00	0.00	0.02	0.00	-0.03	0.05	0.00
4	6	0.00	-0.01	0.00	0.00	-0.03	-0.01	-0.02	0.04	0.00
5	6	0.01	-0.02	0.00	0.07	-0.10	-0.01	-0.14	0.02	0.00
6	6	0.01	0.00	-0.01	0.01	0.00	-0.01	-0.01	0.03	0.00
7	6	-0.01	0.00	0.00	-0.07	-0.01	0.00	-0.01	0.02	0.00
8	1	0.05	0.02	0.00	-0.27	-0.14	0.01	-0.54	-0.26	-0.01
9	8	0.00	0.00	0.00	0.03	-0.01	-0.02	-0.02	-0.02	0.01
10	8	-0.01	0.00	0.00	-0.08	0.00	0.04	0.01	-0.02	-0.01
11	6	0.01	0.00	0.00	0.00	0.00	0.00	0.05	0.03	0.00
12	6	0.00	0.00	0.00	0.00	0.02	0.00	0.02	0.05	0.00
13	8	0.01	0.00	0.00	0.00	0.00	0.00	0.01	0.00	0.01
14	8	0.00	0.00	0.00	-0.01	0.00	0.01	0.00	0.00	0.00
15	1	-0.02	0.02	0.00	-0.07	-0.08	-0.01	-0.01	-0.09	-0.02
16	1	0.04	-0.03	-0.03	0.51	-0.34	-0.34	0.05	-0.04	-0.03
17	1	0.04	-0.01	0.00	-0.19	0.09	-0.01	0.67	-0.27	-0.01
18	8	0.00	0.00	0.00	0.02	0.01	0.01	0.02	-0.02	0.00
19	8	-0.01	0.00	0.00	-0.07	-0.01	-0.02	0.00	-0.02	0.00
20	1	0.04	0.03	0.02	0.41	0.33	0.17	-0.13	-0.11	-0.06
21	8	0.00	0.00	0.00	0.00	0.00	0.00	0.00	0.00	0.00
22	1	0.00	0.00	0.00	0.00	0.00	0.00	0.00	0.00	0.00
23	7	-0.07	0.04	0.28	0.00	0.00	-0.01	0.00	0.00	0.00
24	1	0.02	0.07	0.44	0.00	0.00	-0.01	0.00	0.00	0.00
25	7	-0.14	-0.03	-0.17	0.01	0.00	0.01	0.00	0.00	0.00
26	1	0.02	0.00	-0.01	0.00	0.00	-0.01	0.00	-0.03	0.00
27	6	0.05	-0.04	-0.23	0.00	0.00	0.01	0.00	0.00	0.00
28	1	0.30	-0.03	-0.23	-0.02	0.00	0.01	0.00	0.00	0.00
29	6	0.18	-0.01	-0.05	-0.01	0.00	0.00	0.00	0.00	0.00
30	1	0.21	-0.01	-0.05	-0.01	0.00	-0.01	0.00	0.00	0.00
31	6	0.00	0.02	0.12	0.00	0.00	0.00	0.00	0.00	0.00
32	1	-0.51	0.04	0.30	0.02	0.00	-0.01	0.00	0.00	0.00
33	1	0.00	0.00	0.00	-0.01	0.00	0.00	0.00	0.00	0.00

64 65 66
A A A
Frequencies -- 1319.6539 1335.3566 1367.0584
Red. masses -- 1.6643 5.9516 4.6977
Frc consts -- 1.7077 6.2528 5.1726
IR Inten -- 30.6308 0.4660 299.2068
Raman Activ -- 28.5263 10.6937 6.7020
Depolar (P) -- 0.2087 0.7485 0.0712
Depolar (U) -- 0.3453 0.8561 0.1329

Atom	AN	X	Y	Z	X	Y	Z	X	Y	Z
1	6	0.00	0.00	0.00	0.25	-0.14	-0.02	-0.09	0.07	0.01
2	6	0.00	0.00	0.00	0.00	0.33	0.02	-0.13	0.00	0.02
3	6	0.00	0.00	0.00	-0.19	-0.13	-0.01	0.03	-0.06	-0.01
4	6	0.00	0.00	0.00	0.00	-0.04	-0.03	0.00	0.00	0.01
5	6	0.01	0.00	0.00	-0.24	-0.16	-0.02	-0.10	-0.08	-0.01
6	6	0.00	0.00	0.01	-0.01	-0.04	0.01	0.36	-0.02	-0.21
7	6	0.00	0.00	0.00	-0.01	0.23	0.02	0.02	0.01	0.00
8	1	-0.01	-0.01	0.00	0.19	0.11	-0.01	0.38	0.15	0.01
9	8	0.00	0.00	0.00	-0.04	-0.01	0.02	0.01	0.00	-0.01
10	8	0.00	0.00	0.00	0.03	0.03	0.00	-0.01	-0.01	0.00

11	6	0.00	0.00	0.00	0.19	-0.11	-0.01	0.06	0.05	0.00
12	6	0.00	0.00	0.00	-0.01	-0.05	-0.02	0.00	0.02	0.00
13	8	0.00	0.00	-0.01	0.00	0.01	0.00	-0.07	-0.01	-0.05
14	8	0.00	0.00	0.00	0.01	0.00	-0.01	-0.11	0.03	0.22
15	1	0.00	0.00	0.00	0.02	-0.42	-0.04	0.02	0.06	0.01
16	1	-0.01	0.01	0.01	-0.23	0.18	0.15	0.06	-0.05	-0.04
17	1	-0.01	0.00	0.00	-0.24	0.11	-0.01	0.34	-0.08	0.00
18	8	0.00	0.00	0.00	0.04	0.00	0.01	0.01	-0.01	0.01
19	8	0.00	0.00	0.00	-0.03	0.03	0.00	-0.01	0.00	0.00
20	1	-0.01	-0.01	-0.01	0.27	0.25	0.11	0.01	0.01	0.00
21	8	0.00	0.00	0.00	0.00	0.00	0.00	0.00	0.00	0.01
22	1	0.00	0.00	0.00	0.01	0.00	0.00	0.02	-0.01	-0.06
23	7	0.05	0.01	0.07	0.00	0.00	0.00	0.03	0.00	0.01
24	1	0.40	0.08	0.51	0.01	0.01	0.02	-0.03	-0.01	-0.05
25	7	0.07	-0.02	-0.13	0.00	0.00	0.00	0.02	0.00	0.00
26	1	-0.01	0.00	-0.02	0.00	0.05	0.01	-0.03	-0.08	-0.59
27	6	0.03	0.01	0.06	0.00	0.00	0.00	0.00	0.01	0.04
28	1	-0.63	-0.01	-0.02	-0.02	0.00	0.00	-0.08	0.00	0.03
29	6	-0.05	0.01	0.06	0.00	0.00	0.00	-0.03	0.00	-0.02
30	1	-0.10	-0.04	-0.30	0.00	0.00	-0.01	-0.03	0.01	0.08
31	6	-0.11	-0.01	-0.04	0.00	0.00	0.00	0.01	0.00	-0.01
32	1	0.09	-0.02	-0.11	0.00	0.00	0.00	-0.08	0.00	0.01
33	1	0.00	0.00	0.00	0.00	-0.01	0.00	-0.01	0.05	-0.05

	67	68	69
	A	A	A
Frequencies --	1388.1626	1400.9350	1414.9839
Red. masses --	2.5738	2.9427	2.9004
Frc consts --	2.9222	3.4028	3.4214
IR Inten --	248.8886	17.5980	65.8697
Raman Activ --	1.6944	19.3325	11.7977
Depolar (P) --	0.3804	0.1896	0.1498
Depolar (U) --	0.5511	0.3187	0.2606

Atom	AN	X	Y	Z	X	Y	Z	X	Y	Z
1	6	0.01	-0.05	0.00	-0.09	-0.08	0.00	0.01	-0.02	0.00
2	6	0.00	0.00	0.00	0.07	-0.01	0.00	0.01	0.00	0.00
3	6	-0.07	-0.04	0.00	-0.04	-0.03	0.00	0.00	0.01	0.00
4	6	-0.04	0.18	0.06	-0.05	0.24	0.08	0.00	0.00	0.00
5	6	0.03	-0.07	0.00	-0.10	0.07	0.00	0.01	0.01	0.00
6	6	-0.01	0.00	0.01	-0.03	0.00	0.02	-0.05	0.00	0.03
7	6	-0.01	0.04	0.00	0.08	0.00	0.00	0.00	0.00	0.00
8	1	0.19	0.12	0.00	0.29	0.17	0.00	-0.04	-0.02	0.00
9	8	0.03	-0.02	-0.02	0.05	-0.02	-0.03	0.00	0.00	0.00
10	8	-0.01	-0.06	-0.01	-0.02	-0.08	-0.01	0.00	0.00	0.00
11	6	0.07	-0.04	0.00	-0.02	0.02	0.00	-0.01	-0.01	0.00
12	6	0.03	0.24	0.05	-0.02	-0.18	-0.04	0.00	0.00	0.00
13	8	0.00	0.00	0.00	0.00	0.00	0.00	0.00	0.00	-0.01
14	8	0.00	0.00	-0.01	0.01	0.00	-0.02	0.02	0.00	0.00
15	1	-0.02	0.17	0.02	0.09	0.06	0.01	0.00	-0.01	0.00
16	1	0.33	-0.26	-0.23	0.44	-0.34	-0.30	0.00	0.00	0.00
17	1	-0.33	0.16	0.00	0.26	-0.11	0.00	-0.05	0.01	0.00
18	8	-0.04	-0.03	-0.02	0.05	0.02	0.02	0.00	0.00	0.00
19	8	0.03	-0.07	-0.01	-0.02	0.06	0.00	0.00	0.00	0.00
20	1	-0.46	-0.41	-0.20	0.32	0.30	0.14	0.00	0.00	0.00
21	8	0.00	0.00	0.00	0.00	0.00	0.00	0.00	0.00	0.00
22	1	0.00	0.00	0.01	0.01	0.01	0.02	0.00	0.00	0.00
23	7	0.00	0.00	0.00	0.00	0.00	0.00	0.01	0.02	0.14
24	1	0.00	0.00	0.00	0.01	0.00	0.01	-0.31	-0.05	-0.29
25	7	0.00	0.00	0.00	0.00	0.00	-0.01	0.16	-0.02	-0.15
26	1	0.00	0.01	0.02	-0.01	0.01	0.08	0.01	-0.03	-0.23
27	6	0.00	0.00	0.00	0.00	0.00	0.00	-0.17	0.02	0.14

28	1	0.00	0.00	0.00	0.00	0.00	0.00	0.08	0.03	0.19
29	6	0.00	0.00	0.00	0.00	0.00	0.00	-0.08	-0.01	-0.06
30	1	0.00	0.00	0.00	0.00	0.00	-0.01	-0.06	0.06	0.39
31	6	0.00	0.00	0.00	0.00	0.00	0.00	0.14	-0.01	-0.09
32	1	0.00	0.00	0.00	0.00	0.00	0.00	-0.62	0.02	0.13
33	1	0.01	0.00	0.00	0.02	-0.01	0.01	0.00	0.00	0.00

	70	71	72
	A	A	A
Frequencies --	1456.2150	1506.8138	1515.6774
Red. masses --	4.0610	2.5523	2.9778
Frc consts --	5.0738	3.4143	4.0305
IR Inten --	25.7346	6.4817	4.5594
Raman Activ --	35.3430	0.4034	10.8438
Depolar (P) --	0.6097	0.4183	0.2504
Depolar (U) --	0.7576	0.5899	0.4005

Atom	AN	X	Y	Z	X	Y	Z	X	Y	Z
1	6	-0.01	0.01	0.00	-0.11	-0.12	-0.01	0.12	-0.17	-0.02
2	6	0.00	0.00	0.00	-0.03	0.10	0.01	-0.20	-0.02	0.00
3	6	0.00	0.00	0.00	0.15	-0.01	0.00	0.03	0.09	0.01
4	6	0.00	-0.01	0.00	-0.01	0.05	0.02	-0.02	0.09	0.01
5	6	-0.01	-0.01	0.00	0.13	-0.06	0.00	0.07	0.20	0.02
6	6	0.03	0.00	0.00	0.01	-0.02	0.00	0.04	0.01	0.00
7	6	0.00	0.00	0.00	-0.02	0.19	0.02	-0.07	-0.03	0.00
8	1	0.03	0.01	0.00	-0.32	-0.33	-0.03	-0.33	-0.12	-0.01
9	8	0.00	0.00	0.00	0.03	0.00	-0.01	0.00	-0.01	0.00
10	8	0.00	0.00	0.00	0.00	-0.01	0.00	0.01	-0.01	0.00
11	6	0.00	0.01	0.00	-0.12	-0.05	-0.01	0.08	-0.08	-0.01
12	6	0.00	0.00	0.00	0.01	0.02	0.01	-0.01	-0.09	-0.01
13	8	-0.01	0.00	-0.01	0.00	0.00	-0.01	-0.01	0.00	-0.02
14	8	-0.01	0.00	0.01	0.00	0.00	0.01	0.02	0.01	0.04
15	1	0.00	0.00	0.00	0.02	-0.67	-0.06	-0.10	0.09	0.01
16	1	0.00	0.00	0.00	0.05	-0.03	-0.04	0.04	-0.03	-0.03
17	1	0.03	-0.01	0.00	0.31	-0.29	-0.02	-0.48	0.20	0.01
18	8	0.00	0.00	0.00	-0.02	0.00	-0.01	0.00	0.01	0.00
19	8	0.00	0.00	0.00	0.00	0.00	0.00	0.00	0.01	0.00
20	1	0.00	0.00	0.00	-0.02	-0.02	-0.01	0.04	0.04	0.02
21	8	0.00	0.00	0.00	0.00	0.00	0.00	0.00	0.00	0.00
22	1	0.00	0.00	0.00	0.00	0.00	0.00	0.02	0.01	0.00
23	7	-0.08	0.00	0.00	0.00	0.00	0.00	-0.01	0.00	-0.02
24	1	0.13	0.04	0.28	0.01	0.00	0.01	0.07	0.01	0.09
25	7	0.12	0.01	0.07	0.00	0.00	0.00	-0.01	0.00	0.03
26	1	-0.02	0.00	0.03	0.01	0.00	-0.08	0.05	-0.09	-0.62
27	6	-0.20	-0.03	-0.18	0.00	0.00	0.00	0.01	0.00	-0.02
28	1	0.49	-0.02	-0.13	0.00	0.00	0.00	0.02	0.00	-0.02
29	6	0.03	0.05	0.35	0.00	0.00	0.00	0.00	0.00	0.01
30	1	-0.06	-0.08	-0.55	0.00	0.00	0.00	0.00	0.00	-0.02
31	6	0.09	-0.03	-0.20	0.00	0.00	0.00	0.00	0.00	0.01
32	1	-0.20	-0.02	-0.15	0.00	0.00	0.00	0.03	0.00	0.00
33	1	0.00	0.00	0.00	0.00	0.00	0.00	0.01	-0.01	0.00

	73	74	75
	A	A	A
Frequencies --	1538.4662	1553.0064	1592.3007
Red. masses --	2.3482	1.8990	2.6438
Frc consts --	3.2746	2.6985	3.9494
IR Inten --	35.4002	33.4515	11.3114
Raman Activ --	10.5449	1.7861	6.2853
Depolar (P) --	0.7014	0.3137	0.6277
Depolar (U) --	0.8245	0.4776	0.7712

Atom	AN	X	Y	Z	X	Y	Z	X	Y	Z
1	6	0.02	-0.04	0.00	0.01	-0.04	0.00	0.00	0.01	0.00

2	6	-0.05	0.00	0.00	-0.06	0.00	0.00	0.02	0.00	0.00
3	6	0.02	0.02	0.00	0.02	0.02	0.00	-0.01	-0.01	0.00
4	6	0.00	0.01	0.00	-0.01	0.02	0.00	0.00	0.00	0.00
5	6	0.02	0.04	0.00	0.01	0.04	0.00	0.00	-0.01	0.00
6	6	0.05	-0.02	-0.09	0.08	-0.02	-0.10	-0.05	0.00	0.01
7	6	-0.02	0.00	0.00	-0.02	0.00	0.00	0.01	0.00	0.00
8	1	-0.08	-0.04	0.00	-0.08	-0.04	0.00	0.02	0.02	0.00
9	8	0.00	0.00	0.00	0.00	0.00	0.00	0.00	0.00	0.00
10	8	0.00	0.00	0.00	0.00	0.00	0.00	0.00	0.00	0.00
11	6	0.02	-0.02	0.00	0.03	-0.02	0.00	-0.01	0.01	0.00
12	6	0.00	-0.01	0.00	0.00	-0.02	0.00	0.00	0.00	0.00
13	8	0.01	0.01	0.05	0.01	0.01	0.05	0.01	0.00	0.00
14	8	-0.04	0.00	-0.01	-0.05	0.00	-0.02	0.02	0.00	0.01
15	1	-0.02	0.00	0.00	-0.03	0.00	0.00	0.01	0.00	0.00
16	1	0.01	0.00	-0.01	0.01	-0.01	-0.01	0.00	0.00	0.00
17	1	-0.09	0.04	0.00	-0.09	0.04	0.00	0.03	-0.01	0.00
18	8	0.00	0.00	0.00	0.00	0.00	0.00	0.00	0.00	0.00
19	8	0.00	0.00	0.00	0.00	0.00	0.00	0.00	0.00	0.00
20	1	0.00	0.00	0.00	0.00	0.00	0.00	0.00	0.00	0.00
21	8	0.00	0.00	0.00	0.00	0.00	0.00	0.00	0.00	0.00
22	1	-0.03	-0.02	-0.03	-0.03	-0.02	-0.03	0.01	0.01	0.02
23	7	0.17	0.01	0.08	-0.09	0.00	-0.02	0.08	0.01	0.09
24	1	-0.30	-0.09	-0.58	0.02	0.02	0.16	-0.39	-0.08	-0.54
25	7	0.00	-0.01	-0.04	-0.01	0.00	-0.02	-0.16	0.00	0.02
26	1	-0.03	0.08	0.58	-0.07	0.12	0.87	0.07	-0.05	-0.32
27	6	-0.09	0.00	-0.01	0.09	0.00	0.03	0.21	0.00	-0.03
28	1	0.10	0.00	0.01	-0.19	0.00	0.00	-0.38	-0.02	-0.12
29	6	0.08	0.01	0.04	-0.08	0.00	0.00	-0.10	0.02	0.13
30	1	0.07	-0.02	-0.14	-0.09	0.01	0.06	-0.16	-0.03	-0.20
31	6	-0.18	0.00	-0.03	0.14	0.00	-0.03	0.08	-0.02	-0.14
32	1	0.16	-0.02	-0.14	-0.21	0.01	0.08	-0.28	-0.01	-0.07
33	1	-0.02	0.04	-0.03	-0.03	0.04	-0.03	0.01	-0.02	0.02

	76	77	78
	A	A	A
Frequencies --	1660.3035	1666.4214	1669.8032
Red. masses --	7.4933	1.7771	1.9525
Frc consts --	12.1702	2.9076	3.2075
IR Inten --	63.8524	91.0739	63.0408
Raman Activ --	35.7473	13.9560	9.9870
Depolar (P) --	0.7492	0.6968	0.7163
Depolar (U) --	0.8566	0.8213	0.8347

Atom	AN	X	Y	Z	X	Y	Z	X	Y	Z
1	6	-0.03	0.26	0.02	-0.10	0.02	0.00	-0.12	0.02	0.00
2	6	0.00	-0.35	-0.03	0.06	-0.01	0.00	0.08	-0.01	0.00
3	6	-0.14	-0.26	-0.02	0.11	0.04	0.00	0.12	0.04	0.00
4	6	0.00	-0.06	0.00	-0.02	-0.02	0.01	0.00	0.00	0.00
5	6	0.08	0.27	0.02	-0.10	-0.01	0.00	-0.11	-0.01	0.00
6	6	0.00	0.04	-0.02	0.01	0.01	0.02	-0.03	0.00	0.02
7	6	0.00	0.41	0.04	-0.07	0.01	0.00	-0.07	0.02	0.00
8	1	0.34	0.01	0.00	-0.12	-0.11	-0.01	-0.13	-0.12	-0.01
9	8	0.00	0.01	0.00	0.02	0.01	-0.01	0.01	0.00	0.00
10	8	0.00	0.00	0.00	0.00	0.00	0.00	0.00	0.00	0.00
11	6	0.09	-0.22	-0.02	0.12	-0.05	0.00	0.14	-0.05	0.00
12	6	0.02	-0.06	0.00	-0.01	0.01	0.00	-0.01	0.00	0.00
13	8	0.01	0.00	0.01	-0.01	0.00	-0.01	0.00	0.00	0.00
14	8	0.00	0.00	0.00	0.00	0.00	0.00	0.01	0.00	-0.01
15	1	0.05	-0.45	-0.05	-0.08	-0.01	0.00	-0.08	-0.02	0.00
16	1	-0.03	0.01	0.02	-0.01	0.01	0.01	0.00	0.00	-0.01
17	1	-0.27	-0.07	-0.01	-0.17	0.10	0.01	-0.19	0.11	0.01
18	8	-0.01	0.01	0.00	0.02	0.00	0.00	0.01	0.00	0.00

19	8	0.00	0.00	0.00	0.00	0.00	0.00	0.00	0.00	0.00
20	1	0.03	0.02	0.01	0.00	0.00	0.00	0.00	0.00	0.00
21	8	0.00	0.00	0.00	-0.03	0.01	-0.05	0.03	-0.01	0.05
22	1	0.02	0.02	0.04	0.25	0.32	0.52	-0.24	-0.32	-0.51
23	7	0.00	0.00	0.00	0.00	0.00	0.00	0.00	0.00	0.00
24	1	0.00	-0.01	0.01	-0.02	0.00	-0.03	0.00	0.00	0.00
25	7	0.00	0.00	0.00	0.00	0.00	0.00	-0.01	0.00	0.00
26	1	0.00	-0.03	0.02	0.00	0.01	0.05	0.00	-0.02	-0.08
27	6	0.00	0.00	0.00	0.00	0.00	0.00	0.00	0.00	0.00
28	1	0.00	0.00	0.00	0.00	0.00	0.00	0.00	0.00	0.00
29	6	0.00	0.00	0.00	0.00	0.00	0.00	0.00	0.00	0.00
30	1	0.00	0.00	0.00	0.00	0.00	0.00	0.00	0.00	0.00
31	6	0.00	0.00	0.00	0.00	0.00	0.00	0.00	0.00	0.00
32	1	0.00	0.00	0.00	0.00	0.00	0.00	0.00	0.00	0.00
33	1	0.02	-0.03	0.02	0.31	-0.46	0.34	-0.30	0.46	-0.34

	79	80	81
	A	A	A
Frequencies --	1807.2590	1833.8714	1853.2079
Red. masses --	7.0440	10.1103	10.3695
Frc consts --	13.5553	20.0332	20.9825
IR Inten --	259.5250	180.3863	596.2173
Raman Activ --	25.5500	32.1524	75.6214
Depolar (P) --	0.2784	0.3705	0.1039
Depolar (U) --	0.4355	0.5407	0.1882

Atom	AN	X	Y	Z	X	Y	Z	X	Y	Z
1	6	0.03	0.01	0.00	-0.08	-0.03	0.01	-0.04	-0.02	0.00
2	6	-0.07	-0.01	-0.02	0.01	-0.01	-0.01	0.03	0.01	0.00
3	6	-0.01	-0.01	0.00	0.01	-0.02	0.00	0.02	0.01	0.00
4	6	-0.08	-0.02	0.03	0.60	0.18	-0.24	0.23	0.07	-0.09
5	6	0.02	0.01	0.00	0.02	0.00	0.00	-0.10	0.02	-0.01
6	6	0.30	0.11	0.49	0.05	0.03	0.06	0.06	0.01	0.05
7	6	0.00	0.00	0.00	0.00	0.03	0.00	-0.01	-0.01	0.00
8	1	0.02	0.02	0.00	0.00	-0.04	0.00	-0.02	-0.02	0.00
9	8	0.05	0.01	-0.02	-0.37	-0.09	0.16	-0.14	-0.04	0.06
10	8	0.01	0.00	0.00	-0.06	0.00	0.03	-0.02	0.00	0.01
11	6	-0.01	0.00	0.00	0.01	-0.02	0.00	0.02	0.01	0.00
12	6	-0.03	0.00	-0.01	-0.26	0.03	-0.06	0.66	-0.10	0.15
13	8	-0.18	-0.06	-0.30	-0.03	-0.01	-0.04	-0.03	-0.01	-0.04
14	8	0.01	-0.01	-0.08	0.00	-0.01	-0.01	-0.01	0.00	-0.01
15	1	0.01	0.00	0.00	0.00	-0.03	0.00	-0.01	0.01	0.00
16	1	-0.04	0.02	0.03	0.31	-0.22	-0.21	0.12	-0.08	-0.08
17	1	0.01	-0.01	0.00	-0.01	-0.01	0.00	-0.01	0.04	0.00
18	8	0.02	0.00	0.00	0.16	-0.02	0.04	-0.41	0.04	-0.10
19	8	0.00	0.00	0.00	0.02	0.00	0.01	-0.06	-0.01	-0.02
20	1	-0.01	-0.01	0.00	-0.12	-0.10	-0.05	0.31	0.26	0.13
21	8	0.01	0.00	0.01	0.00	0.00	-0.01	0.00	0.00	0.00
22	1	-0.05	-0.07	-0.11	0.03	0.03	0.06	0.02	0.02	0.03
23	7	0.01	0.00	0.03	0.00	0.00	0.00	0.00	0.00	0.00
24	1	-0.13	-0.04	-0.24	-0.02	0.01	-0.04	-0.02	-0.01	-0.03
25	7	-0.03	0.00	-0.01	0.00	0.00	0.00	0.00	0.00	0.00
26	1	-0.17	0.08	0.59	-0.03	0.05	0.09	-0.02	0.00	0.10
27	6	0.02	0.00	0.00	0.00	0.00	0.00	0.00	0.00	0.00
28	1	-0.02	0.00	-0.01	0.00	0.00	0.00	0.00	0.00	0.00
29	6	0.00	0.00	0.00	0.00	0.00	0.00	0.00	0.00	0.00
30	1	-0.01	0.00	-0.02	0.00	0.00	0.00	0.00	0.00	0.00
31	6	0.00	0.00	-0.02	0.00	0.00	0.00	0.00	0.00	0.00
32	1	-0.03	0.00	-0.01	0.00	0.00	0.00	0.00	0.00	0.00
33	1	-0.06	0.09	-0.07	0.00	-0.08	0.06	0.01	-0.04	0.02

82	83	84
A	A	A

Frequencies -- 2865.5254 3215.7496 3235.7032
Red. masses -- 1.0796 1.0888 1.0923
Frc consts -- 5.2232 6.6338 6.7382
IR Inten -- 3339.3498 3.5099 0.1232
Raman Activ -- 457.6215 72.9676 61.6941
Depolar (P) -- 0.3299 0.5834 0.7449
Depolar (U) -- 0.4962 0.7369 0.8538

Atom	AN	X	Y	Z	X	Y	Z	X	Y	Z
1	6	0.00	0.00	0.00	0.00	0.00	0.00	0.00	0.00	0.00
2	6	0.00	0.00	0.00	0.00	0.00	0.00	0.00	0.00	0.00
3	6	0.00	0.00	0.00	0.02	-0.02	0.00	0.03	-0.05	0.00
4	6	0.00	0.00	0.00	0.00	0.00	0.00	0.00	0.00	0.00
5	6	0.00	0.00	0.00	0.00	0.00	0.00	0.00	0.00	0.00
6	6	0.00	0.00	0.02	0.00	0.00	0.00	0.00	0.00	0.00
7	6	0.00	0.00	0.00	-0.08	0.00	0.00	0.00	0.01	0.00
8	1	0.00	0.00	0.00	-0.18	0.28	0.03	-0.38	0.61	0.06
9	8	0.00	0.00	0.00	0.00	0.00	0.00	0.00	0.00	0.00
10	8	0.00	0.00	0.00	0.00	0.00	0.00	0.00	0.00	0.00
11	6	0.00	0.00	0.00	0.01	0.02	0.00	-0.03	-0.05	0.00
12	6	0.00	0.00	0.00	0.00	0.00	0.00	0.00	0.00	0.00
13	8	0.00	0.00	-0.01	0.00	0.00	0.00	0.00	0.00	0.00
14	8	-0.07	0.00	-0.01	0.00	0.00	0.00	0.00	0.00	0.00
15	1	0.00	0.00	0.00	0.88	0.04	0.01	-0.02	-0.01	0.00
16	1	0.00	0.00	0.00	0.00	0.00	0.00	0.00	0.00	0.00
17	1	0.00	0.00	0.00	-0.15	-0.29	-0.03	0.31	0.61	0.06
18	8	0.00	0.00	0.00	0.00	0.00	0.00	0.00	0.00	0.00
19	8	0.00	0.00	0.00	0.00	0.00	0.00	0.00	0.00	0.00
20	1	0.00	0.00	0.00	0.00	0.00	0.00	0.00	0.00	0.00
21	8	0.00	0.00	0.00	0.00	0.00	0.00	0.00	0.00	0.00
22	1	0.00	-0.01	-0.01	0.00	0.00	0.00	0.00	0.00	0.00
23	7	0.00	0.00	-0.01	0.00	0.00	0.00	0.00	0.00	0.00
24	1	-0.02	0.01	0.04	0.00	0.00	0.00	0.00	0.00	0.00
25	7	0.00	0.00	0.00	0.00	0.00	0.00	0.00	0.00	0.00
26	1	1.00	0.02	0.04	0.00	0.00	0.00	0.00	0.00	0.00
27	6	0.00	0.00	0.00	0.00	0.00	0.00	0.00	0.00	0.00
28	1	0.00	0.00	0.02	0.00	0.00	0.00	0.00	0.00	0.00
29	6	0.00	0.00	0.00	0.00	0.00	0.00	0.00	0.00	0.00
30	1	0.00	0.00	0.00	0.00	0.00	0.00	0.00	0.00	0.00
31	6	0.00	0.00	0.00	0.00	0.00	0.00	0.00	0.00	0.00
32	1	0.01	0.00	0.00	0.00	0.00	0.00	0.00	0.00	0.00
33	1	0.00	0.00	-0.01	0.00	0.00	0.00	0.00	0.00	0.00

85 86 87
A A A

Frequencies -- 3241.0335 3268.5754 3282.1381
Red. masses -- 1.0973 1.0937 1.0960
Frc consts -- 6.7910 6.8841 6.9560
IR Inten -- 2.7298 1.5472 0.3987
Raman Activ -- 200.5251 76.4417 52.9038
Depolar (P) -- 0.1068 0.5847 0.6198
Depolar (U) -- 0.1930 0.7380 0.7653

Atom	AN	X	Y	Z	X	Y	Z	X	Y	Z
1	6	0.00	0.00	0.00	0.00	0.00	0.00	0.00	0.00	0.00
2	6	0.00	0.00	0.00	0.00	0.00	0.00	0.00	0.00	0.00
3	6	-0.03	0.05	0.00	0.00	0.00	0.00	0.00	0.00	0.00
4	6	0.00	0.00	0.00	0.00	0.00	0.00	0.00	0.00	0.00
5	6	0.00	0.00	0.00	0.00	0.00	0.00	0.00	0.00	0.00
6	6	0.00	0.00	0.00	0.00	0.00	0.00	0.00	0.00	0.00
7	6	-0.04	0.00	0.00	0.00	0.00	0.00	0.00	0.00	0.00
8	1	0.31	-0.50	-0.05	0.00	0.00	0.00	0.00	0.00	0.00
9	8	0.00	0.00	0.00	0.00	0.00	0.00	0.00	0.00	0.00

10	8	0.00	0.00	0.00	0.00	0.00	0.00	0.00	0.00	0.00
11	6	-0.02	-0.05	0.00	0.00	0.00	0.00	0.00	0.00	0.00
12	6	0.00	0.00	0.00	0.00	0.00	0.00	0.00	0.00	0.00
13	8	0.00	0.00	0.00	0.00	0.00	0.00	0.00	0.00	0.00
14	8	0.00	0.00	0.00	0.00	0.00	0.00	0.00	0.00	0.00
15	1	0.46	0.02	0.00	0.00	0.00	0.00	0.00	0.00	0.00
16	1	0.00	0.00	0.00	0.00	0.00	0.00	0.00	0.00	0.00
17	1	0.29	0.58	0.05	0.00	0.00	0.00	0.00	0.00	0.00
18	8	0.00	0.00	0.00	0.00	0.00	0.00	0.00	0.00	0.00
19	8	0.00	0.00	0.00	0.00	0.00	0.00	0.00	0.00	0.00
20	1	0.00	0.00	0.00	0.00	0.00	0.00	0.00	0.00	0.00
21	8	0.00	0.00	0.00	0.00	0.00	0.00	0.00	0.00	0.00
22	1	0.00	0.00	0.00	0.00	0.00	0.00	0.00	0.00	0.00
23	7	0.00	0.00	0.00	0.00	0.00	0.00	0.00	0.00	0.00
24	1	0.00	0.00	0.00	0.00	0.00	0.00	0.00	0.00	0.01
25	7	0.00	0.00	0.00	0.00	0.00	0.00	0.00	0.00	0.00
26	1	0.00	0.00	0.00	-0.01	0.00	0.00	0.01	0.00	0.00
27	6	0.00	0.00	0.00	0.01	-0.01	-0.08	0.00	0.01	0.04
28	1	0.00	0.00	0.00	-0.11	0.13	0.86	0.05	-0.06	-0.40
29	6	0.00	0.00	0.00	-0.04	0.00	0.01	-0.05	0.00	0.00
30	1	0.00	0.00	0.00	0.44	-0.01	-0.05	0.56	-0.01	-0.05
31	6	0.00	0.00	0.00	0.00	0.00	0.01	0.02	0.01	0.06
32	1	0.00	0.00	0.00	-0.05	-0.02	-0.15	-0.21	-0.10	-0.67
33	1	0.00	0.00	0.00	0.00	0.00	0.00	0.00	0.00	0.00

	88	89	90
	A	A	A
Frequencies --	3298.5533	3507.5915	3842.8696
Red. masses --	1.1085	1.0863	1.0647
Frc consts --	7.1058	7.8744	9.2635
IR Inten --	0.0069	813.7683	128.5563
Raman Activ --	173.9057	159.2094	123.4045
Depolar (P) --	0.1205	0.2382	0.2482
Depolar (U) --	0.2151	0.3847	0.3977

Atom	AN	X	Y	Z	X	Y	Z	X	Y	Z
1	6	0.00	0.00	0.00	0.00	0.00	0.00	0.00	0.00	0.00
2	6	0.00	0.00	0.00	0.00	0.00	0.00	0.00	0.00	0.00
3	6	0.00	0.00	0.00	0.00	0.00	0.00	0.00	0.00	0.00
4	6	0.00	0.00	0.00	0.00	0.00	0.00	0.00	0.00	0.00
5	6	0.00	0.00	0.00	0.00	0.00	0.00	0.00	0.00	0.00
6	6	0.00	0.00	0.00	0.00	0.00	0.00	0.00	0.00	0.00
7	6	0.00	0.00	0.00	0.00	0.00	0.00	0.00	0.00	0.00
8	1	0.00	0.00	0.00	0.00	0.00	0.00	0.00	0.00	0.00
9	8	0.00	0.00	0.00	0.00	0.00	0.00	0.00	0.00	0.00
10	8	0.00	0.00	0.00	0.00	0.00	0.00	-0.03	-0.05	0.00
11	6	0.00	0.00	0.00	0.00	0.00	0.00	0.00	0.00	0.00
12	6	0.00	0.00	0.00	0.00	0.00	0.00	0.00	0.00	0.00
13	8	0.00	0.00	0.00	0.00	0.00	0.00	0.00	0.00	0.00
14	8	0.00	0.00	0.00	0.00	0.00	0.00	0.00	0.00	0.00
15	1	0.00	0.00	0.00	0.00	0.00	0.00	0.00	0.00	0.00
16	1	0.00	0.00	0.00	0.00	0.00	0.00	0.48	0.87	-0.05
17	1	0.00	0.00	0.00	0.00	0.00	0.00	0.00	0.00	0.00
18	8	0.00	0.00	0.00	0.00	0.00	0.00	0.00	0.00	0.00
19	8	0.00	0.00	0.00	0.00	0.00	0.00	0.00	0.00	0.00
20	1	0.00	0.00	0.00	0.00	0.00	0.00	-0.01	0.02	0.00
21	8	0.00	0.00	0.00	0.00	0.00	0.00	0.00	0.00	0.00
22	1	0.00	0.00	0.00	0.00	0.00	0.00	0.00	-0.01	0.00
23	7	0.00	0.00	0.00	-0.06	0.01	0.04	0.00	0.00	0.00
24	1	0.01	0.00	-0.01	0.80	-0.09	-0.58	0.00	0.00	0.00
25	7	0.00	0.00	0.00	0.00	0.00	0.00	0.00	0.00	0.00
26	1	0.01	0.00	0.00	0.05	0.00	0.00	0.00	0.00	0.00

27	6	0.00	0.00	0.02	0.00	0.00	0.00	0.00	0.00	0.00
28	1	0.02	-0.03	-0.22	0.00	0.00	0.00	0.00	0.00	0.00
29	6	-0.07	0.00	0.01	0.00	0.00	0.00	0.00	0.00	0.00
30	1	0.69	-0.01	-0.07	0.00	0.00	0.00	0.00	0.00	0.00
31	6	-0.02	-0.01	-0.06	0.00	0.00	0.00	0.00	0.00	0.00
32	1	0.19	0.10	0.64	-0.01	0.00	-0.01	0.00	0.00	0.00
33	1	0.00	0.00	0.00	0.00	0.00	0.00	0.01	0.02	0.01

	91	92	93
	A	A	A
Frequencies --	3851.3965	3865.3861	3933.6807
Red. masses --	1.0648	1.0464	1.0801
Frc consts --	9.3055	9.2117	9.8470
IR Inten --	117.8240	82.7927	72.6648
Raman Activ --	130.0299	68.8623	22.8479
Depolar (P) --	0.2528	0.0790	0.7486
Depolar (U) --	0.4035	0.1465	0.8562

Atom	AN	X	Y	Z	X	Y	Z	X	Y	Z
1	6	0.00	0.00	0.00	0.00	0.00	0.00	0.00	0.00	0.00
2	6	0.00	0.00	0.00	0.00	0.00	0.00	0.00	0.00	0.00
3	6	0.00	0.00	0.00	0.00	0.00	0.00	0.00	0.00	0.00
4	6	0.00	0.00	0.00	0.00	0.00	0.00	0.00	0.00	0.00
5	6	0.00	0.00	0.00	0.00	0.00	0.00	0.00	0.00	0.00
6	6	0.00	0.00	0.00	0.00	0.00	0.00	0.00	0.00	0.00
7	6	0.00	0.00	0.00	0.00	0.00	0.00	0.00	0.00	0.00
8	1	0.00	0.00	0.00	0.00	0.00	0.00	0.00	0.00	0.00
9	8	0.00	0.00	0.00	0.00	0.00	0.00	0.00	0.00	0.00
10	8	0.00	0.00	0.00	0.00	0.00	0.00	0.00	0.00	0.00
11	6	0.00	0.00	0.00	0.00	0.00	0.00	0.00	0.00	0.00
12	6	0.00	0.00	0.00	0.00	0.00	0.00	0.00	0.00	0.00
13	8	0.00	0.00	0.00	0.00	0.00	0.00	0.00	0.00	0.00
14	8	0.00	0.00	0.00	0.00	0.00	0.00	0.00	0.00	0.00
15	1	0.00	0.00	0.00	0.00	0.00	0.00	0.00	0.00	0.00
16	1	-0.01	-0.02	0.00	0.01	0.02	0.00	0.00	0.01	0.00
17	1	0.00	0.00	0.00	0.00	0.00	0.00	0.00	0.00	0.00
18	8	0.00	0.00	0.00	0.00	0.00	0.00	0.00	0.00	0.00
19	8	0.04	-0.05	0.00	0.00	0.00	0.00	0.00	0.00	0.00
20	1	-0.58	0.81	-0.02	0.01	-0.01	0.00	0.00	0.00	0.00
21	8	0.00	0.00	0.00	0.03	-0.01	0.04	0.00	0.07	0.02
22	1	0.00	0.01	0.00	-0.27	0.64	-0.22	0.27	-0.58	0.24
23	7	0.00	0.00	0.00	0.00	0.00	0.00	0.00	0.00	0.00
24	1	0.00	0.00	0.00	0.00	0.00	0.00	0.00	0.00	0.00
25	7	0.00	0.00	0.00	0.00	0.00	0.00	0.00	0.00	0.00
26	1	0.00	0.00	0.00	0.01	0.00	0.00	-0.01	0.00	0.00
27	6	0.00	0.00	0.00	0.00	0.00	0.00	0.00	0.00	0.00
28	1	0.00	0.00	0.00	0.00	0.00	0.00	0.00	0.00	0.00
29	6	0.00	0.00	0.00	0.00	0.00	0.00	0.00	0.00	0.00
30	1	0.00	0.00	0.00	0.00	0.00	0.00	0.00	0.00	0.00
31	6	0.00	0.00	0.00	0.00	0.00	0.00	0.00	0.00	0.00
32	1	0.00	0.00	0.00	0.00	0.00	0.00	0.00	0.00	0.00
33	1	0.00	-0.01	0.00	-0.16	-0.49	-0.45	-0.19	-0.49	-0.50

PYRHEMx2H2O

Harmonic frequencies (cm**-1), IR intensities (KM/Mole), Raman scattering activities (A**4/AMU), depolarization ratios for plane and unpolarized incident light, reduced masses (AMU), force constants (mDyne/A), and normal coordinates:

	1	2	3
	A	A	A
Frequencies --	15.4329	26.9787	40.1672

Red. masses -- 6.5240 6.5273 6.9554
Frc consts -- 0.0009 0.0028 0.0066
IR Inten -- 1.1870 1.1282 1.2141
Raman Activ -- 1.4175 0.8241 1.5354
Depolar (P) -- 0.7483 0.7482 0.7346
Depolar (U) -- 0.8560 0.8560 0.8470

Atom	AN	X	Y	Z	X	Y	Z	X	Y	Z
1	8	-0.17	0.10	0.00	-0.03	-0.05	0.14	0.05	-0.01	-0.02
2	1	-0.12	0.10	-0.02	-0.16	-0.08	0.17	-0.03	0.01	0.04
3	1	-0.19	0.15	-0.02	0.07	-0.08	0.23	0.07	-0.12	0.01
4	6	-0.04	0.02	0.02	0.03	0.01	-0.07	0.01	0.02	0.03
5	6	0.01	0.04	-0.01	0.03	0.02	-0.03	0.01	0.02	0.05
6	6	-0.04	-0.03	0.05	0.03	0.02	-0.08	0.01	0.01	0.01
7	6	-0.09	0.05	0.01	0.03	0.01	-0.07	0.01	0.01	0.00
8	6	0.06	0.01	0.00	0.02	0.03	0.03	0.01	0.02	0.01
9	6	0.01	0.09	-0.05	0.03	0.02	-0.03	0.01	0.00	0.06
10	6	0.01	-0.06	0.06	0.03	0.03	-0.03	0.01	0.01	-0.01
11	1	-0.08	-0.04	0.07	0.03	0.01	-0.11	0.01	0.01	0.01
12	8	-0.09	0.10	-0.01	0.06	0.02	0.00	0.00	0.00	-0.03
13	8	-0.12	0.03	0.04	0.00	0.01	-0.16	0.02	0.02	0.01
14	6	0.06	-0.04	0.03	0.02	0.04	0.02	0.01	0.00	-0.02
15	6	0.11	0.03	-0.03	0.02	0.05	0.07	0.01	0.02	0.02
16	8	0.01	0.11	-0.06	0.02	0.05	-0.05	-0.01	-0.11	0.11
17	8	0.02	0.09	-0.06	0.03	-0.02	-0.02	0.03	0.09	0.00
18	1	0.01	-0.10	0.09	0.03	0.03	-0.04	0.01	0.00	-0.03
19	1	-0.14	0.05	0.03	0.00	0.01	-0.08	0.02	0.01	-0.01
20	1	0.09	-0.06	0.04	0.02	0.05	0.07	0.02	0.00	-0.05
21	8	0.13	0.07	-0.06	0.06	0.05	-0.04	-0.08	0.05	0.28
22	8	0.15	0.01	-0.03	-0.03	0.05	0.26	0.13	-0.01	-0.32
23	1	0.18	0.02	-0.05	-0.02	0.07	0.27	0.11	-0.02	-0.29
24	8	-0.02	0.10	-0.05	-0.36	-0.14	0.18	-0.22	0.00	0.11
25	1	0.01	0.10	-0.07	-0.21	-0.13	0.16	-0.14	0.00	0.12
26	7	0.00	-0.09	0.01	0.02	0.01	-0.06	-0.01	-0.12	0.02
27	1	0.00	-0.01	-0.02	0.01	0.04	-0.06	-0.03	-0.18	0.08
28	7	0.01	-0.05	-0.01	0.03	-0.02	-0.05	0.03	0.07	-0.07
29	1	0.02	0.07	-0.05	0.03	0.00	-0.03	0.03	0.10	-0.03
30	6	0.02	-0.18	0.05	0.04	-0.06	-0.04	0.04	0.13	-0.14
31	1	0.02	-0.18	0.04	0.05	-0.09	-0.03	0.08	0.27	-0.22
32	6	0.00	-0.31	0.10	0.03	-0.07	-0.05	0.01	-0.02	-0.10
33	1	0.00	-0.43	0.15	0.03	-0.10	-0.05	0.01	-0.01	-0.14
34	6	0.00	-0.24	0.08	0.01	-0.02	-0.06	-0.03	-0.18	0.00
35	1	-0.01	-0.30	0.10	0.00	-0.01	-0.07	-0.06	-0.32	0.06
36	1	0.02	0.07	-0.04	-0.45	-0.19	0.13	-0.33	0.01	0.07

4 5 6
A A A
Frequencies -- 43.2738 55.0453 63.7116
Red. masses -- 5.7647 5.4946 5.4305
Frc consts -- 0.0064 0.0098 0.0130
IR Inten -- 1.4757 2.8424 2.6869
Raman Activ -- 2.4327 1.5344 1.8011
Depolar (P) -- 0.7394 0.7446 0.7216
Depolar (U) -- 0.8502 0.8536 0.8383

Atom	AN	X	Y	Z	X	Y	Z	X	Y	Z
1	8	0.14	-0.07	-0.18	0.12	-0.08	0.05	0.21	-0.12	-0.10
2	1	0.05	-0.04	-0.11	0.12	-0.08	0.06	0.16	-0.07	-0.04
3	1	0.13	-0.25	-0.18	0.06	-0.18	0.00	0.11	-0.39	-0.18
4	6	0.00	0.01	0.06	-0.02	-0.01	-0.03	-0.04	0.00	-0.01
5	6	0.00	0.00	0.00	-0.01	-0.01	-0.06	-0.02	0.02	-0.01
6	6	0.00	0.04	0.21	-0.02	-0.01	-0.02	-0.04	-0.04	-0.06
7	6	0.00	-0.01	-0.03	-0.03	0.00	0.00	-0.05	0.01	0.01

8	6	0.00	0.01	0.03	0.00	-0.01	-0.04	0.01	0.00	-0.01
9	6	0.00	0.00	-0.05	-0.01	0.01	-0.08	-0.02	0.04	-0.01
10	6	0.00	0.05	0.28	-0.01	-0.02	-0.02	-0.02	-0.06	-0.10
11	1	0.01	0.05	0.26	-0.03	-0.01	0.00	-0.06	-0.05	-0.07
12	8	-0.02	-0.04	-0.08	-0.07	0.02	-0.13	-0.09	0.01	-0.12
13	8	0.03	0.02	-0.05	0.02	-0.01	0.18	0.01	0.00	0.19
14	6	0.00	0.03	0.17	0.00	-0.01	-0.02	0.01	-0.04	-0.06
15	6	0.01	-0.03	-0.10	0.00	0.00	-0.01	0.04	0.04	0.06
16	8	0.01	0.03	-0.05	0.00	0.00	-0.06	-0.02	0.04	-0.01
17	8	-0.01	-0.01	-0.03	-0.02	0.06	-0.09	-0.01	0.06	-0.03
18	1	0.00	0.08	0.40	-0.01	-0.02	-0.01	-0.02	-0.09	-0.14
19	1	0.05	0.00	-0.11	0.03	-0.02	0.16	0.04	-0.02	0.13
20	1	0.00	0.04	0.20	0.00	-0.01	0.00	0.03	-0.06	-0.08
21	8	0.04	-0.07	-0.17	0.04	0.01	-0.10	0.02	0.11	0.14
22	8	-0.01	0.00	-0.13	-0.03	0.00	0.15	0.11	0.00	0.03
23	1	-0.01	-0.03	-0.22	-0.02	0.02	0.16	0.13	0.03	0.08
24	8	-0.19	-0.05	-0.01	0.08	-0.08	0.07	0.00	-0.04	0.07
25	1	-0.12	-0.07	0.03	-0.01	-0.07	0.05	-0.03	-0.05	0.09
26	7	0.02	0.05	0.02	0.01	-0.09	0.05	-0.03	0.15	-0.08
27	1	0.04	0.06	-0.01	0.02	-0.16	0.06	-0.02	0.21	-0.12
28	7	-0.02	0.02	0.03	-0.03	0.17	-0.08	-0.04	-0.04	0.01
29	1	-0.01	0.00	-0.01	-0.01	0.12	-0.11	-0.01	0.02	-0.01
30	6	-0.03	0.00	0.08	-0.05	0.25	-0.07	-0.04	-0.18	0.09
31	1	-0.06	-0.02	0.10	-0.08	0.45	-0.16	-0.05	-0.33	0.16
32	6	0.00	0.02	0.11	-0.02	0.03	0.07	-0.04	-0.07	0.04
33	1	0.00	0.01	0.15	-0.02	0.04	0.11	-0.04	-0.13	0.08
34	6	0.03	0.05	0.07	0.02	-0.19	0.15	-0.03	0.14	-0.07
35	1	0.07	0.07	0.07	0.05	-0.39	0.26	-0.02	0.27	-0.13
36	1	-0.28	-0.01	-0.04	-0.02	-0.05	0.03	-0.13	0.02	0.03

	7	8	9
	A	A	A
Frequencies --	73.8558	89.5653	91.5869
Red. masses --	6.6719	6.3756	5.9916
Frc consts --	0.0214	0.0301	0.0296
IR Inten --	5.5975	2.1294	0.9515
Raman Activ --	0.8225	1.6054	1.3695
Depolar (P) --	0.7380	0.7417	0.7417
Depolar (U) --	0.8492	0.8517	0.8517

Atom	AN	X	Y	Z	X	Y	Z	X	Y	Z
1	8	-0.09	-0.05	0.26	-0.07	0.18	0.02	-0.07	0.08	0.04
2	1	-0.05	-0.15	0.15	0.01	0.14	-0.05	-0.02	0.07	0.00
3	1	0.00	0.25	0.32	-0.11	0.30	-0.03	-0.04	0.22	0.06
4	6	-0.01	0.01	-0.08	0.04	0.00	0.01	0.04	-0.04	0.01
5	6	-0.01	0.01	-0.07	0.02	-0.02	-0.04	0.01	-0.05	0.01
6	6	-0.01	0.02	0.03	0.04	0.02	0.03	0.04	0.00	0.01
7	6	-0.01	0.00	-0.15	0.02	0.02	0.03	0.04	-0.04	0.01
8	6	0.00	0.03	0.03	0.00	-0.01	-0.04	-0.03	-0.03	0.01
9	6	-0.01	-0.02	-0.10	0.02	-0.05	-0.05	0.01	-0.05	0.00
10	6	-0.01	0.04	0.15	0.02	0.03	0.00	0.00	0.02	0.01
11	1	-0.01	0.02	0.03	0.05	0.04	0.07	0.07	0.02	0.01
12	8	-0.03	-0.01	-0.18	-0.05	0.07	-0.17	0.03	-0.03	-0.05
13	8	0.00	0.00	-0.14	0.05	-0.02	0.29	0.06	-0.05	0.07
14	6	0.00	0.04	0.14	0.00	0.02	-0.02	-0.03	0.01	0.00
15	6	0.01	0.05	0.06	-0.04	-0.01	0.01	-0.08	-0.06	0.03
16	8	-0.02	-0.09	-0.07	0.02	-0.13	-0.01	0.01	-0.07	0.02
17	8	0.00	0.02	-0.13	0.03	0.02	-0.09	0.02	0.02	-0.03
18	1	0.00	0.06	0.25	0.02	0.05	0.01	0.01	0.05	0.00
19	1	0.01	-0.01	-0.06	0.02	0.02	0.20	0.04	-0.03	0.04
20	1	0.00	0.06	0.23	-0.01	0.03	-0.02	-0.05	0.02	0.00
21	8	-0.04	0.08	0.19	-0.05	-0.03	0.03	-0.10	-0.10	0.07

22	8	0.08	0.02	-0.09	-0.07	0.01	0.06	-0.12	-0.03	-0.01
23	1	0.08	0.03	-0.05	-0.10	0.00	0.11	-0.16	-0.05	0.01
24	8	0.16	-0.16	-0.02	-0.19	0.04	-0.13	-0.04	0.05	-0.03
25	1	0.06	-0.13	-0.08	-0.05	0.04	-0.14	-0.01	0.05	-0.04
26	7	0.03	0.01	-0.02	0.08	-0.13	0.03	0.07	0.28	-0.16
27	1	0.06	-0.01	-0.07	0.11	-0.18	0.01	0.08	0.32	-0.19
28	7	-0.05	-0.05	0.00	0.03	-0.20	0.06	0.05	0.27	-0.15
29	1	0.00	-0.03	-0.10	0.02	-0.07	-0.04	0.02	0.12	-0.09
30	6	-0.08	-0.02	0.07	0.02	-0.05	0.03	0.03	-0.02	0.02
31	1	-0.15	-0.06	0.10	-0.02	-0.07	0.05	0.01	-0.08	0.05
32	6	-0.02	0.06	0.10	0.06	0.13	-0.02	0.05	-0.22	0.13
33	1	-0.02	0.10	0.15	0.06	0.28	-0.04	0.04	-0.47	0.28
34	6	0.06	0.07	0.03	0.10	0.07	-0.02	0.07	-0.02	0.02
35	1	0.13	0.13	0.03	0.15	0.16	-0.05	0.09	-0.07	0.05
36	1	0.21	-0.20	0.00	-0.39	0.01	-0.22	-0.10	0.04	-0.06

	10	11	12
	A	A	A
Frequencies --	117.0127	120.1911	131.3333
Red. masses --	6.2585	6.9908	5.5673
Frc consts --	0.0505	0.0595	0.0566
IR Inten --	7.9666	5.3090	1.7898
Raman Activ --	0.4540	0.1995	0.1267
Depolar (P) --	0.7494	0.7457	0.5928
Depolar (U) --	0.8567	0.8543	0.7443

Atom	AN	X	Y	Z	X	Y	Z	X	Y	Z
1	8	0.02	-0.07	-0.10	0.06	0.15	0.04	-0.01	0.04	-0.07
2	1	-0.02	-0.08	-0.10	0.10	0.03	-0.08	-0.04	0.17	0.06
3	1	-0.08	-0.29	-0.18	0.08	0.37	0.05	0.00	-0.13	-0.04
4	6	-0.04	0.03	0.04	0.06	0.00	0.02	0.03	-0.02	-0.02
5	6	-0.02	0.02	-0.01	0.03	-0.02	-0.01	0.02	-0.03	-0.03
6	6	-0.04	0.02	0.04	0.06	0.01	0.01	0.03	-0.01	0.01
7	6	-0.06	0.05	0.12	0.11	-0.01	0.07	0.04	-0.01	0.02
8	6	-0.03	0.02	-0.03	0.04	-0.02	-0.01	0.02	-0.02	-0.01
9	6	-0.01	-0.01	-0.05	0.01	-0.03	-0.01	0.01	-0.01	-0.04
10	6	-0.04	0.01	-0.02	0.06	0.00	-0.01	0.02	0.00	0.02
11	1	-0.04	0.03	0.08	0.06	0.01	0.03	0.03	-0.01	0.02
12	8	0.00	0.07	0.27	0.13	-0.04	0.12	0.07	0.00	0.11
13	8	-0.12	0.04	0.00	0.13	0.00	0.05	0.01	-0.02	-0.04
14	6	-0.03	0.01	-0.05	0.05	-0.01	-0.02	0.02	-0.01	0.02
15	6	-0.04	0.04	0.04	0.08	0.01	0.02	0.01	-0.02	0.00
16	8	-0.03	0.05	-0.10	-0.01	0.00	-0.05	0.01	-0.06	-0.02
17	8	0.02	-0.12	-0.02	0.00	-0.10	0.01	0.00	0.05	-0.07
18	1	-0.04	0.00	-0.04	0.06	0.01	-0.02	0.02	0.00	0.05
19	1	-0.12	0.04	0.03	0.13	0.01	0.03	0.00	-0.01	-0.03
20	1	-0.02	0.00	-0.07	0.04	-0.01	-0.03	0.01	0.00	0.04
21	8	-0.07	0.08	0.13	0.08	0.06	0.04	0.00	-0.03	0.01
22	8	0.00	0.02	0.01	0.14	-0.03	0.02	0.00	-0.02	0.02
23	1	-0.01	0.04	0.07	0.17	0.00	0.04	-0.01	-0.02	0.04
24	8	-0.04	-0.16	-0.15	-0.11	-0.10	-0.24	0.04	0.23	0.26
25	1	-0.01	-0.17	-0.13	-0.03	-0.11	-0.22	0.05	0.26	0.19
26	7	0.17	-0.05	-0.08	-0.18	0.03	-0.03	0.03	-0.09	-0.15
27	1	0.23	-0.08	-0.16	-0.17	0.03	-0.05	0.10	-0.14	-0.23
28	7	0.05	-0.02	-0.11	-0.21	0.06	-0.06	-0.14	-0.12	-0.16
29	1	0.01	-0.10	-0.09	0.03	-0.05	-0.01	0.01	0.00	-0.12
30	6	0.00	0.04	-0.01	-0.23	0.05	0.00	-0.22	-0.02	-0.01
31	1	-0.11	0.07	0.00	-0.26	0.07	-0.01	-0.38	-0.02	0.02
32	6	0.10	0.04	0.10	-0.20	0.00	0.05	-0.07	0.07	0.10
33	1	0.09	0.08	0.20	-0.21	-0.03	0.10	-0.08	0.16	0.23
34	6	0.22	-0.02	0.04	-0.17	-0.02	0.03	0.09	0.02	0.00
35	1	0.33	-0.04	0.09	-0.14	-0.05	0.06	0.25	0.05	0.03

36	1	0.24	-0.20	-0.04	-0.09	-0.11	-0.23	-0.19	0.20	0.16

13 14 15
A A A

Frequencies -- 153.2835 165.0599 173.8999
Red. masses -- 7.3987 4.9749 5.4792
Frc consts -- 0.1024 0.0799 0.0976
IR Inten -- 8.6795 5.8052 1.5273
Raman Activ -- 0.3733 0.1210 2.5698
Depolar (P) -- 0.5922 0.7179 0.6983
Depolar (U) -- 0.7439 0.8358 0.8223

Atom	AN	X	Y	Z	X	Y	Z	X	Y	Z
1	8	0.00	-0.04	0.05	-0.11	-0.14	-0.08	0.06	0.01	0.05
2	1	0.01	-0.14	-0.04	-0.15	-0.06	0.01	0.00	-0.04	0.04
3	1	0.01	0.10	0.05	-0.24	-0.50	-0.17	0.16	0.13	0.15
4	6	0.00	0.03	0.07	0.10	0.02	0.00	-0.02	0.04	0.17
5	6	0.02	0.06	0.20	0.10	0.01	-0.02	-0.01	0.01	0.01
6	6	0.00	-0.01	-0.08	0.10	0.03	0.00	-0.02	0.04	0.26
7	6	-0.03	0.03	-0.04	0.01	0.08	0.02	-0.03	0.02	0.03
8	6	0.03	0.04	0.15	0.08	0.02	-0.02	0.00	-0.03	-0.16
9	6	0.02	0.07	0.22	0.08	-0.02	-0.01	-0.01	-0.01	0.02
10	6	0.01	-0.02	-0.10	0.09	0.04	0.00	-0.01	-0.02	0.00
11	1	-0.01	-0.04	-0.21	0.12	0.04	0.02	-0.03	0.07	0.45
12	8	-0.08	0.03	-0.17	0.02	0.21	0.03	-0.04	-0.06	0.00
13	8	-0.03	0.03	-0.03	-0.12	0.00	0.03	-0.01	0.08	-0.15
14	6	0.03	0.01	0.02	0.08	0.04	-0.01	0.00	-0.07	-0.26
15	6	0.02	0.00	0.00	0.01	-0.02	0.00	0.01	0.00	-0.06
16	8	0.02	0.07	0.22	0.06	-0.14	0.04	-0.01	-0.18	0.12
17	8	0.06	0.07	0.20	0.09	0.09	-0.08	0.01	0.15	-0.06
18	1	0.01	-0.06	-0.24	0.09	0.05	0.01	-0.01	-0.03	-0.01
19	1	-0.03	0.03	-0.05	-0.17	0.05	0.08	0.02	0.04	-0.17
20	1	0.03	0.00	-0.03	0.07	0.05	0.00	0.01	-0.12	-0.46
21	8	0.06	-0.10	-0.12	-0.01	-0.12	0.03	0.01	0.07	-0.05
22	8	-0.04	0.05	-0.05	-0.09	0.04	0.00	0.02	-0.03	0.11
23	1	-0.04	-0.01	-0.19	-0.16	0.00	0.03	0.04	0.02	0.21
24	8	0.00	-0.12	-0.12	0.01	-0.09	-0.01	-0.01	-0.02	-0.03
25	1	-0.01	-0.16	-0.03	-0.02	-0.09	-0.01	-0.05	-0.03	-0.02
26	7	0.07	-0.07	-0.16	-0.10	0.04	0.03	0.01	0.02	-0.02
27	1	0.13	-0.11	-0.22	-0.12	0.03	0.06	0.01	-0.02	0.00
28	7	-0.09	-0.08	-0.18	-0.06	0.02	0.04	0.00	-0.01	-0.01
29	1	0.06	0.02	0.05	0.12	0.07	-0.04	0.00	0.10	-0.05
30	6	-0.16	-0.01	-0.03	-0.05	-0.01	0.02	0.00	-0.02	0.01
31	1	-0.29	0.00	-0.02	-0.02	-0.03	0.03	-0.01	-0.04	0.02
32	6	-0.03	0.03	0.08	-0.08	-0.01	-0.01	0.00	-0.01	0.01
33	1	-0.04	0.09	0.20	-0.08	-0.04	-0.03	0.00	-0.01	0.02
34	6	0.12	-0.01	-0.01	-0.12	0.02	0.00	0.01	0.02	-0.01
35	1	0.26	0.00	0.03	-0.15	0.03	-0.01	0.02	0.03	-0.01
36	1	0.02	-0.03	-0.09	0.35	-0.14	0.12	0.19	-0.02	0.05

16 17 18
A A A

Frequencies -- 188.1315 195.6819 203.0733
Red. masses -- 7.3469 7.3975 1.2346
Frc consts -- 0.1532 0.1669 0.0300
IR Inten -- 1.5941 5.3482 80.6503
Raman Activ -- 0.4845 0.8121 0.5097
Depolar (P) -- 0.7453 0.4645 0.7038
Depolar (U) -- 0.8541 0.6344 0.8262

Atom	AN	X	Y	Z	X	Y	Z	X	Y	Z
1	8	-0.09	-0.03	-0.04	-0.23	-0.14	-0.10	-0.01	-0.04	0.00
2	1	-0.03	-0.04	-0.07	-0.12	-0.05	-0.07	0.08	-0.12	-0.11
3	1	-0.07	0.10	-0.03	-0.31	-0.18	-0.17	-0.04	0.16	-0.04

4	6	0.07	0.00	0.10	0.05	0.01	-0.03	0.00	0.01	0.02
5	6	0.06	-0.02	-0.01	-0.02	-0.01	-0.06	0.00	0.01	0.02
6	6	0.07	0.04	0.22	0.05	0.10	0.05	0.00	0.00	0.00
7	6	0.02	0.00	-0.03	0.05	-0.01	-0.02	-0.01	0.01	0.01
8	6	0.05	-0.04	-0.13	-0.08	0.03	-0.09	0.01	0.01	0.02
9	6	0.06	0.07	0.00	-0.01	-0.13	0.08	0.00	0.00	0.00
10	6	0.06	0.01	0.03	-0.01	0.14	0.05	0.00	0.00	-0.02
11	1	0.09	0.07	0.39	0.11	0.14	0.12	-0.01	0.00	-0.01
12	8	-0.03	0.01	-0.19	0.03	0.02	-0.09	0.00	0.00	0.04
13	8	0.01	-0.01	-0.04	0.03	-0.05	0.10	-0.02	0.01	-0.02
14	6	0.05	-0.04	-0.20	-0.07	0.08	-0.05	0.01	0.00	0.00
15	6	-0.04	-0.05	0.02	0.01	0.10	-0.07	0.00	0.00	0.00
16	8	0.10	0.34	-0.13	0.05	-0.14	0.13	-0.03	-0.02	-0.01
17	8	0.01	-0.13	0.12	-0.08	-0.20	0.16	0.03	0.02	-0.03
18	1	0.06	0.02	0.05	-0.01	0.22	0.12	0.00	-0.01	-0.04
19	1	-0.01	0.01	-0.08	0.00	-0.01	0.11	-0.01	0.01	-0.02
20	1	0.05	-0.06	-0.36	-0.11	0.10	-0.06	0.01	0.00	-0.01
21	8	-0.11	-0.07	0.20	0.04	0.27	-0.11	0.01	-0.01	-0.01
22	8	-0.08	-0.03	0.03	0.14	0.00	0.02	-0.01	0.01	0.00
23	1	-0.17	-0.03	0.19	0.25	0.08	0.04	-0.01	0.00	-0.02
24	8	0.05	-0.01	0.07	0.05	-0.01	0.07	0.06	-0.02	0.00
25	1	0.07	-0.02	0.10	0.06	-0.01	0.06	0.13	0.02	-0.09
26	7	-0.03	-0.05	0.01	0.02	0.02	-0.05	0.00	0.01	0.01
27	1	-0.04	0.02	-0.02	0.02	-0.01	-0.04	-0.01	0.01	0.02
28	7	-0.04	-0.02	-0.01	0.00	0.03	-0.05	0.01	0.01	0.01
29	1	0.03	-0.07	0.09	-0.09	-0.16	0.14	0.03	0.00	-0.02
30	6	-0.04	0.02	-0.02	-0.01	0.00	-0.02	0.01	0.00	0.01
31	1	-0.04	0.04	-0.03	-0.03	0.01	-0.02	0.01	0.00	0.01
32	6	-0.04	0.02	-0.02	0.01	-0.03	0.01	0.00	0.00	0.00
33	1	-0.04	0.04	-0.02	0.00	-0.05	0.04	0.00	-0.01	-0.01
34	6	-0.03	-0.02	0.00	0.02	-0.02	-0.01	-0.01	0.00	0.00
35	1	-0.03	-0.03	0.01	0.04	-0.04	0.01	-0.02	0.00	0.00
36	1	-0.29	0.07	-0.06	-0.28	0.01	-0.05	-0.87	0.01	-0.37

	19	20	21
	A	A	A
Frequencies --	216.1443	227.5314	254.3365
Red. masses --	8.9547	6.0887	6.3695
Frc consts --	0.2465	0.1857	0.2428
IR Inten --	11.6216	41.0244	22.8625
Raman Activ --	0.2233	1.3401	0.2209
Depolar (P) --	0.6033	0.7467	0.6206
Depolar (U) --	0.7525	0.8550	0.7659

Atom	AN	X	Y	Z	X	Y	Z	X	Y	Z
1	8	0.20	0.34	-0.05	0.14	-0.05	0.11	-0.09	0.24	-0.20
2	1	0.11	0.31	-0.06	0.17	-0.17	-0.01	0.03	0.28	-0.22
3	1	0.16	0.18	-0.07	0.06	0.01	0.03	-0.10	0.36	-0.22
4	6	0.01	0.03	-0.01	-0.03	-0.03	-0.20	-0.03	-0.03	-0.06
5	6	0.01	0.01	-0.02	0.01	-0.02	-0.22	-0.01	-0.01	-0.05
6	6	0.00	-0.04	-0.01	-0.03	-0.04	-0.02	-0.03	-0.01	-0.03
7	6	-0.10	0.11	-0.06	-0.05	0.03	-0.04	0.02	-0.05	0.01
8	6	0.03	-0.01	0.00	0.04	-0.03	-0.17	-0.01	-0.01	-0.05
9	6	0.00	-0.15	0.02	0.02	0.04	0.02	0.00	0.09	0.01
10	6	0.02	-0.05	0.00	0.00	-0.03	0.16	-0.02	0.00	0.05
11	1	-0.04	-0.06	-0.02	-0.06	-0.04	0.05	-0.02	-0.01	-0.02
12	8	-0.12	0.24	-0.10	0.00	0.09	0.10	0.04	-0.08	0.06
13	8	-0.20	0.10	-0.10	-0.07	0.01	0.00	0.05	-0.05	0.04
14	6	0.03	-0.04	0.01	0.04	-0.03	0.05	-0.01	0.00	0.02
15	6	0.04	-0.02	0.01	0.00	-0.02	-0.05	-0.02	0.00	-0.02
16	8	-0.04	-0.17	0.00	0.23	0.02	0.16	0.09	0.06	0.09
17	8	0.00	-0.32	0.09	-0.16	0.06	0.13	-0.05	0.20	-0.01

18	1	0.02	-0.07	-0.01	0.00	-0.01	0.40	-0.02	0.01	0.12
19	1	-0.25	0.11	-0.18	-0.06	-0.01	0.09	0.01	-0.02	-0.05
20	1	0.05	-0.04	0.02	0.06	-0.02	0.18	-0.01	0.01	0.07
21	8	0.03	-0.05	0.01	-0.04	-0.02	0.05	-0.03	0.02	0.00
22	8	0.02	-0.01	0.01	-0.04	-0.01	0.03	-0.02	-0.01	0.00
23	1	0.01	-0.01	0.02	-0.10	0.00	0.17	-0.02	0.00	0.04
24	8	0.07	-0.09	0.16	-0.01	0.06	-0.12	0.09	-0.34	0.15
25	1	0.10	-0.05	0.10	-0.08	0.05	-0.09	-0.04	-0.30	0.06
26	7	-0.01	0.04	0.00	0.01	-0.01	-0.04	0.00	-0.01	-0.01
27	1	-0.01	0.01	0.02	0.01	-0.02	-0.02	0.00	0.00	0.00
28	7	0.01	0.05	0.00	-0.01	-0.01	-0.04	0.00	-0.02	0.00
29	1	0.01	-0.25	0.08	-0.17	0.05	0.17	-0.06	0.17	0.01
30	6	0.01	0.02	0.00	-0.01	-0.02	-0.04	0.00	-0.01	-0.01
31	1	0.02	0.02	0.00	-0.01	-0.02	-0.04	0.00	-0.02	-0.01
32	6	0.00	-0.03	0.01	0.00	-0.02	-0.03	0.00	0.00	-0.02
33	1	0.00	-0.06	0.01	0.00	-0.03	-0.02	0.00	0.01	-0.02
34	6	-0.01	-0.01	0.01	0.00	-0.02	-0.03	0.00	0.00	-0.02
35	1	-0.03	-0.03	0.02	0.00	-0.02	-0.03	0.00	0.01	-0.02
36	1	-0.18	-0.15	0.05	-0.35	0.18	-0.24	-0.05	-0.39	0.09

	22	23	24
	A	A	A
Frequencies --	286.4211	297.6712	321.8827
Red. masses --	6.3761	1.0819	9.1047
Frc consts --	0.3082	0.0565	0.5558
IR Inten --	42.5755	70.1604	7.5258
Raman Activ --	1.0560	0.3744	2.7218
Depolar (P) --	0.7003	0.5035	0.7377
Depolar (U) --	0.8237	0.6697	0.8491

Atom	AN	X	Y	Z	X	Y	Z	X	Y	Z
1	8	0.10	0.05	0.05	0.00	-0.03	-0.01	0.11	0.07	0.05
2	1	-0.04	0.05	0.11	0.21	-0.24	-0.28	0.11	-0.05	-0.06
3	1	0.03	-0.32	0.02	-0.04	0.50	-0.09	0.08	0.16	0.01
4	6	0.12	-0.07	0.04	0.01	0.00	0.01	0.01	-0.14	0.03
5	6	-0.03	-0.14	0.10	0.00	-0.01	0.00	0.13	-0.05	0.04
6	6	0.12	0.03	-0.02	0.01	0.01	-0.01	0.01	-0.08	0.00
7	6	0.07	-0.02	-0.02	0.01	0.01	0.01	-0.10	-0.16	0.03
8	6	-0.15	-0.11	0.05	-0.01	-0.01	0.01	-0.05	0.07	0.00
9	6	-0.03	-0.03	0.06	-0.01	-0.01	-0.01	0.23	-0.04	0.03
10	6	0.00	0.12	-0.01	0.01	0.01	-0.01	-0.08	0.00	0.01
11	1	0.21	0.07	-0.06	0.02	0.01	-0.01	0.11	-0.04	-0.03
12	8	0.06	0.21	-0.03	0.00	0.03	0.01	-0.10	-0.14	0.03
13	8	-0.11	-0.10	-0.06	-0.02	0.00	0.00	-0.19	-0.20	0.00
14	6	-0.14	0.03	0.02	-0.01	0.01	0.01	-0.05	0.04	0.02
15	6	-0.10	-0.11	0.01	0.00	-0.01	0.00	-0.11	0.14	-0.03
16	8	-0.02	0.12	-0.02	-0.01	-0.01	-0.01	0.24	0.01	-0.02
17	8	-0.01	0.15	-0.04	-0.01	0.00	-0.01	0.30	-0.03	-0.04
18	1	0.00	0.25	-0.04	0.01	0.02	-0.02	-0.08	0.04	0.00
19	1	-0.21	-0.03	-0.05	-0.02	0.00	-0.02	-0.27	-0.17	-0.07
20	1	-0.25	0.08	-0.01	-0.02	0.01	0.01	0.00	0.01	0.03
21	8	-0.06	0.09	-0.05	0.00	0.00	0.00	-0.09	0.26	-0.07
22	8	0.11	-0.27	0.03	0.01	-0.02	0.00	-0.09	0.14	-0.05
23	1	0.30	-0.16	-0.01	0.03	-0.01	0.00	-0.10	0.12	-0.10
24	8	0.02	0.00	-0.04	-0.01	-0.01	0.01	-0.02	0.04	-0.01
25	1	0.04	0.06	-0.17	-0.24	-0.20	0.40	0.06	-0.03	0.14
26	7	0.01	-0.02	0.01	0.00	0.00	0.00	-0.05	0.00	0.01
27	1	0.01	0.02	-0.01	0.00	0.00	0.00	-0.07	0.02	0.02
28	7	0.01	-0.02	0.01	0.00	0.00	0.00	-0.03	0.00	0.00
29	1	-0.01	0.16	-0.05	-0.01	0.03	-0.02	0.35	-0.01	-0.09
30	6	0.01	-0.01	0.00	0.00	0.00	0.00	-0.04	0.01	0.01
31	1	0.01	-0.01	0.00	0.00	0.00	0.00	-0.04	0.01	0.01

32	6	0.01	0.01	-0.01	0.00	0.00	0.00	-0.05	0.01	0.01
33	1	0.01	0.02	-0.02	0.00	0.00	0.00	-0.05	0.00	0.00
34	6	0.01	0.00	0.00	0.00	0.00	0.00	-0.06	0.00	0.01
35	1	0.00	0.01	-0.01	0.00	0.00	0.00	-0.06	0.01	0.01
36	1	-0.17	-0.13	-0.13	0.10	0.52	0.12	0.04	0.17	0.04

	25	26	27
	A	A	A
Frequencies --	375.5475	380.7118	406.1245
Red. masses --	5.9273	4.8319	5.4645
Frc consts --	0.4925	0.4126	0.5310
IR Inten --	32.3658	61.6855	1.3217
Raman Activ --	0.1158	3.3113	2.8732
Depolar (P) --	0.5897	0.1240	0.7457
Depolar (U) --	0.7419	0.2207	0.8543

Atom	AN	X	Y	Z	X	Y	Z	X	Y	Z
1	8	0.01	0.00	0.00	-0.05	-0.07	-0.05	0.03	0.07	0.05
2	1	-0.05	0.02	0.04	-0.03	-0.03	-0.01	0.02	0.04	0.02
3	1	0.12	0.05	0.10	0.23	0.38	0.18	-0.24	-0.38	-0.18
4	6	0.02	0.01	0.17	-0.04	0.06	0.06	-0.07	0.01	-0.08
5	6	0.01	-0.06	-0.09	0.02	0.02	-0.02	0.00	0.06	-0.03
6	6	0.04	0.08	0.09	-0.05	0.01	-0.12	-0.04	0.22	-0.05
7	6	-0.04	-0.01	0.09	-0.08	0.11	0.06	-0.09	-0.09	-0.02
8	6	-0.02	0.03	0.19	-0.02	-0.02	-0.08	0.06	0.02	-0.05
9	6	0.01	-0.07	-0.16	0.10	0.01	-0.01	0.01	0.08	0.01
10	6	0.00	0.05	-0.23	-0.05	0.03	-0.01	0.00	0.24	0.00
11	1	0.11	0.13	0.14	-0.06	-0.01	-0.25	0.06	0.27	-0.01
12	8	-0.08	-0.06	0.00	-0.10	-0.05	0.00	-0.08	-0.18	0.03
13	8	-0.05	0.02	-0.02	0.08	0.22	0.00	-0.11	-0.10	0.02
14	6	-0.03	0.09	0.07	-0.03	0.06	0.10	0.04	0.21	-0.09
15	6	0.03	0.01	0.11	-0.07	-0.09	-0.05	0.08	-0.07	0.01
16	8	0.28	-0.02	-0.02	0.13	0.00	0.01	-0.06	-0.02	0.02
17	8	-0.25	0.00	-0.04	0.15	-0.04	-0.01	0.07	-0.02	0.02
18	1	0.00	0.02	-0.55	-0.05	0.02	0.00	0.00	0.19	0.10
19	1	-0.05	0.01	-0.10	0.22	0.12	-0.08	-0.17	-0.07	0.12
20	1	-0.08	0.12	0.08	-0.03	0.08	0.25	-0.04	0.25	-0.11
21	8	0.07	-0.06	0.00	-0.08	-0.01	0.00	0.06	-0.17	0.04
22	8	0.03	0.03	-0.02	0.06	-0.22	0.05	0.10	-0.10	0.04
23	1	0.07	-0.03	-0.25	0.19	-0.09	0.19	0.14	-0.05	0.10
24	8	0.01	0.00	0.00	-0.02	0.02	0.03	0.01	-0.01	-0.02
25	1	-0.12	0.06	-0.12	0.20	0.10	-0.14	-0.06	-0.10	0.18
26	7	0.00	0.00	0.00	-0.02	0.00	0.00	0.00	0.01	0.00
27	1	0.01	0.00	0.00	-0.03	0.00	0.01	0.00	-0.01	0.01
28	7	-0.01	0.00	0.00	-0.01	0.00	0.00	0.00	0.00	0.00
29	1	-0.26	0.05	0.10	0.16	-0.07	-0.04	0.08	-0.05	-0.01
30	6	0.00	-0.01	-0.02	-0.01	0.01	0.00	0.00	0.00	0.00
31	1	0.01	-0.01	-0.02	-0.02	0.00	0.01	0.00	0.00	0.00
32	6	0.00	-0.01	-0.02	-0.02	0.00	0.00	0.00	0.00	0.00
33	1	0.00	-0.01	-0.02	-0.02	0.00	0.00	0.00	0.00	0.00
34	6	0.00	-0.01	-0.01	-0.02	0.00	0.00	0.00	0.00	0.00
35	1	-0.02	-0.01	-0.02	-0.02	0.00	0.01	0.00	0.00	0.00
36	1	-0.03	-0.08	-0.02	0.12	-0.28	0.05	-0.09	0.26	-0.02

	28	29	30
	A	A	A
Frequencies --	416.9455	446.6578	490.8540
Red. masses --	1.4255	3.8984	1.1546
Frc consts --	0.1460	0.4582	0.1639
IR Inten --	57.4163	15.4117	27.5832
Raman Activ --	0.9269	3.6535	0.6709
Depolar (P) --	0.4062	0.1751	0.5954
Depolar (U) --	0.5777	0.2981	0.7464

Atom	AN	X	Y	Z	X	Y	Z	X	Y	Z
1	8	0.02	-0.06	-0.04	0.00	0.01	0.00	-0.02	0.00	-0.04
2	1	0.09	-0.07	-0.09	-0.22	0.01	0.09	-0.48	0.02	0.18
3	1	0.33	0.63	0.20	0.18	-0.12	0.19	0.51	-0.07	0.48
4	6	0.01	-0.02	-0.03	0.07	0.02	0.22	-0.01	-0.01	-0.04
5	6	0.00	0.00	0.00	0.03	-0.01	0.02	0.00	0.00	0.00
6	6	0.02	0.03	0.00	0.09	-0.02	-0.13	-0.01	0.00	0.00
7	6	-0.01	-0.03	0.00	0.01	-0.03	0.12	0.00	0.00	0.00
8	6	0.01	0.00	-0.01	0.07	-0.04	-0.20	-0.01	0.00	0.01
9	6	-0.02	0.01	0.01	-0.05	0.00	0.00	0.01	0.00	0.00
10	6	0.02	0.04	0.01	0.11	0.01	0.00	-0.02	0.01	0.01
11	1	0.04	0.05	0.02	0.10	-0.06	-0.37	-0.01	0.01	0.02
12	8	-0.01	0.02	0.01	-0.05	-0.07	-0.03	0.00	0.03	0.01
13	8	-0.05	-0.07	0.03	-0.06	-0.03	-0.02	0.01	-0.01	0.04
14	6	0.01	0.03	-0.03	0.09	0.04	0.13	-0.01	0.00	-0.03
15	6	0.02	0.00	0.00	0.03	0.00	-0.13	0.00	0.00	0.02
16	8	-0.04	0.00	0.00	-0.10	0.00	-0.02	0.01	0.00	0.00
17	8	-0.01	0.01	0.01	-0.10	0.02	0.02	0.02	0.00	0.00
18	1	0.02	0.05	0.05	0.11	0.02	-0.02	-0.02	0.01	0.04
19	1	-0.06	-0.06	-0.11	-0.12	0.01	-0.20	-0.01	0.01	-0.26
20	1	-0.01	0.03	-0.05	0.07	0.09	0.35	-0.01	-0.01	-0.05
21	8	0.02	-0.02	0.01	-0.02	0.07	0.03	0.01	-0.01	0.00
22	8	0.01	0.02	0.00	-0.03	0.04	0.00	0.01	0.00	0.00
23	1	-0.02	0.01	0.00	-0.22	0.06	0.39	0.02	0.00	-0.06
24	8	-0.03	0.03	0.03	0.02	-0.01	-0.02	0.03	-0.02	-0.02
25	1	0.21	0.17	-0.27	-0.12	-0.04	0.05	-0.20	-0.09	0.14
26	7	0.00	0.00	0.00	0.01	0.00	0.00	0.00	0.00	0.00
27	1	0.00	0.00	0.00	0.02	0.00	-0.01	0.00	0.00	0.00
28	7	0.00	0.00	0.00	0.00	0.00	0.00	0.00	0.00	0.00
29	1	-0.02	-0.02	0.01	-0.11	0.04	0.05	0.03	0.02	-0.01
30	6	0.00	0.00	0.00	0.00	0.00	0.00	0.00	0.00	0.00
31	1	0.00	0.00	0.00	0.01	0.00	0.00	0.00	0.00	0.00
32	6	0.00	0.00	0.00	0.01	0.00	0.00	0.00	0.00	0.00
33	1	0.00	0.00	0.00	0.01	0.00	0.00	0.00	0.00	0.00
34	6	0.00	0.00	0.00	0.01	0.00	0.00	0.00	0.00	0.00
35	1	0.01	0.00	0.00	0.02	0.00	0.00	0.00	0.00	0.00
36	1	0.16	-0.44	0.04	-0.08	0.13	-0.03	-0.11	0.27	-0.04

	31	32	33
	A	A	A
Frequencies --	547.9792	553.0716	597.1266
Red. masses --	4.9908	4.3530	3.7970
Frc consts --	0.8830	0.7845	0.7977
IR Inten --	2.0101	14.0298	29.3137
Raman Activ --	0.6730	6.6938	0.6397
Depolar (P) --	0.4958	0.2227	0.7142
Depolar (U) --	0.6629	0.3643	0.8332

Atom	AN	X	Y	Z	X	Y	Z	X	Y	Z
1	8	0.01	0.00	0.00	0.00	0.00	-0.01	0.01	0.00	0.01
2	1	-0.10	-0.06	-0.02	0.01	0.07	0.05	0.06	0.01	-0.01
3	1	0.10	0.01	0.08	0.05	0.03	0.04	-0.04	0.02	-0.04
4	6	-0.07	0.08	0.03	0.07	-0.02	-0.13	-0.05	0.06	0.13
5	6	0.01	0.15	-0.08	0.09	0.03	0.01	0.01	0.11	0.20
6	6	-0.13	0.04	0.14	0.13	-0.01	0.01	-0.10	-0.01	-0.15
7	6	0.10	-0.05	-0.05	-0.10	0.03	-0.04	0.05	-0.02	-0.01
8	6	0.09	0.10	0.04	0.10	0.04	0.12	0.03	0.06	0.04
9	6	0.00	0.16	-0.07	0.00	0.01	-0.01	0.02	-0.01	-0.10
10	6	0.00	-0.09	-0.15	0.19	-0.01	0.04	-0.04	0.02	0.20
11	1	-0.23	0.00	0.21	0.11	-0.01	0.08	-0.15	-0.10	-0.54
12	8	0.12	0.06	-0.03	-0.08	-0.12	0.05	0.05	0.03	-0.02
13	8	-0.03	-0.17	0.02	-0.03	0.08	0.03	0.00	-0.07	0.00

14	6	0.14	0.04	0.13	0.17	0.00	-0.06	0.05	0.00	-0.14
15	6	-0.10	-0.01	-0.06	-0.13	-0.02	0.06	-0.02	0.01	-0.03
16	8	-0.02	-0.05	0.04	-0.08	-0.03	-0.04	0.12	-0.04	-0.03
17	8	0.01	-0.04	0.03	-0.08	0.02	0.04	-0.10	-0.04	-0.04
18	1	-0.01	-0.30	-0.38	0.19	-0.03	0.08	-0.05	-0.07	0.23
19	1	-0.18	-0.06	-0.08	0.04	0.03	0.08	-0.07	-0.02	-0.02
20	1	0.24	-0.01	0.21	0.18	-0.03	-0.19	0.11	-0.10	-0.46
21	8	-0.12	0.05	-0.04	-0.10	0.13	-0.06	-0.04	-0.02	0.00
22	8	0.00	-0.15	0.01	-0.06	-0.11	0.02	0.01	-0.03	0.03
23	1	0.13	0.01	0.22	0.27	-0.12	-0.61	0.15	0.01	-0.11
24	8	-0.01	0.00	-0.01	0.01	-0.01	0.00	0.01	0.00	0.01
25	1	0.24	-0.05	0.10	-0.29	0.05	-0.11	-0.23	0.07	-0.12
26	7	0.00	0.01	-0.01	0.00	0.00	0.00	0.00	0.00	0.00
27	1	0.00	-0.04	0.02	0.01	-0.01	-0.01	0.01	-0.01	0.00
28	7	0.00	0.00	0.00	0.00	0.00	0.00	-0.01	0.00	0.00
29	1	0.00	-0.13	0.06	-0.08	0.06	0.10	-0.10	0.01	0.05
30	6	0.00	0.01	0.00	0.00	0.00	0.00	0.00	0.00	0.00
31	1	0.00	0.01	0.00	0.00	0.00	0.00	0.00	0.00	-0.01
32	6	0.00	0.00	0.00	0.01	0.00	0.00	0.00	0.00	0.00
33	1	0.00	0.00	0.00	0.01	0.00	0.00	0.00	0.00	0.00
34	6	0.00	0.00	0.00	0.01	0.00	0.00	0.00	0.00	0.00
35	1	0.00	-0.01	0.01	0.01	0.00	0.00	-0.01	0.00	0.00
36	1	0.01	0.02	0.00	-0.02	-0.01	-0.01	0.00	-0.04	0.00

	34	35	36
	A	A	A
Frequencies --	599.6541	624.2088	628.6379
Red. masses --	1.3125	1.3710	2.9615
Frc consts --	0.2781	0.3147	0.6895
IR Inten --	76.4465	55.1212	4.3421
Raman Activ --	2.0618	1.5890	0.3522
Depolar (P) --	0.6609	0.3636	0.5036
Depolar (U) --	0.7958	0.5333	0.6699

Atom	AN	X	Y	Z	X	Y	Z	X	Y	Z
1	8	0.00	-0.01	0.00	0.00	0.01	0.00	0.00	-0.01	0.00
2	1	0.06	0.08	0.04	-0.15	-0.14	-0.07	0.07	0.07	0.04
3	1	0.01	0.04	0.00	0.03	-0.06	0.04	-0.01	0.03	-0.01
4	6	0.00	-0.02	-0.05	0.01	0.01	-0.03	0.00	-0.01	0.01
5	6	0.02	0.00	0.05	-0.02	0.00	0.02	0.01	0.00	-0.01
6	6	0.01	0.00	0.03	0.03	0.00	-0.02	-0.01	0.00	0.01
7	6	-0.03	0.00	-0.05	0.00	0.02	0.01	0.00	-0.01	-0.01
8	6	0.01	0.02	0.08	-0.01	0.00	0.07	0.00	0.00	-0.03
9	6	0.02	-0.03	0.01	-0.03	0.01	-0.03	0.02	0.00	0.01
10	6	0.00	0.00	-0.01	0.04	0.01	0.04	-0.02	0.00	-0.01
11	1	0.02	0.01	0.05	0.02	-0.01	-0.03	0.00	0.00	0.01
12	8	-0.01	0.00	0.02	-0.01	-0.03	0.00	0.00	0.01	0.00
13	8	0.01	0.01	0.01	-0.02	0.02	0.01	0.01	-0.01	-0.01
14	6	0.01	0.01	0.00	0.00	-0.03	-0.03	0.00	0.01	0.01
15	6	0.00	-0.01	-0.04	0.00	-0.01	0.01	0.00	0.01	0.00
16	8	0.01	0.00	-0.02	0.03	0.00	0.01	-0.01	0.00	-0.01
17	8	-0.01	0.00	0.00	-0.03	0.00	-0.02	0.01	0.00	0.01
18	1	0.00	-0.01	-0.06	0.04	0.03	0.04	-0.02	-0.01	-0.02
19	1	0.05	-0.02	0.06	-0.05	0.04	-0.05	0.03	-0.02	0.03
20	1	0.01	-0.01	-0.10	-0.02	-0.04	-0.13	0.00	0.02	0.06
21	8	0.00	0.01	-0.01	0.00	0.02	-0.01	0.00	-0.01	0.01
22	8	0.02	-0.02	-0.05	0.00	-0.01	-0.03	0.00	0.01	0.01
23	1	-0.30	0.08	0.82	-0.15	0.01	0.30	0.06	0.00	-0.12
24	8	0.01	0.00	0.01	-0.02	0.00	-0.01	0.01	0.00	0.01
25	1	-0.38	0.08	-0.15	0.77	-0.17	0.33	-0.38	0.08	-0.16
26	7	0.00	0.00	0.00	0.00	0.05	-0.02	0.00	0.19	-0.09
27	1	0.00	0.00	0.00	0.00	-0.02	0.01	0.00	-0.07	0.03

28	7	0.00	0.00	0.00	0.00	-0.01	0.01	0.00	-0.05	0.03
29	1	-0.01	0.06	0.02	-0.05	-0.10	-0.02	0.02	0.02	0.03
30	6	0.00	0.00	0.00	0.00	-0.02	0.01	0.00	-0.10	0.05
31	1	0.00	0.00	0.00	0.00	-0.05	0.03	-0.01	-0.29	0.14
32	6	0.00	0.00	0.00	0.00	0.04	-0.02	0.00	0.20	-0.10
33	1	0.00	0.00	0.00	0.00	0.06	-0.03	0.01	0.30	-0.15
34	6	0.00	0.00	0.00	0.00	-0.05	0.02	0.00	-0.21	0.10
35	1	0.00	0.01	0.00	-0.01	-0.12	0.06	-0.01	-0.57	0.28
36	1	-0.01	-0.02	-0.01	0.03	0.06	0.01	-0.01	-0.02	-0.01

	37	38	39
	A	A	A
Frequencies --	661.8456	673.0182	698.1152
Red. masses --	4.6323	5.2897	4.8311
Frc consts --	1.1955	1.4117	1.3872
IR Inten --	82.9748	0.3002	45.2418
Raman Activ --	2.3569	0.3728	0.8714
Depolar (P) --	0.1663	0.7346	0.2121
Depolar (U) --	0.2852	0.8470	0.3500

Atom	AN	X	Y	Z	X	Y	Z	X	Y	Z
1	8	-0.01	0.00	0.00	0.00	0.00	0.00	0.01	-0.01	-0.01
2	1	0.04	0.04	0.02	0.02	0.02	0.01	0.00	-0.01	0.00
3	1	-0.02	0.00	-0.02	0.00	0.00	0.00	0.06	0.07	0.03
4	6	-0.11	-0.03	0.02	0.00	-0.01	0.00	0.01	0.19	-0.03
5	6	-0.07	-0.06	0.04	0.01	-0.01	0.00	-0.16	0.07	0.00
6	6	-0.13	0.15	-0.02	0.01	-0.01	0.00	0.04	0.08	0.00
7	6	-0.02	-0.05	-0.01	0.00	0.00	0.00	0.08	0.12	-0.06
8	6	0.07	-0.17	0.02	0.00	-0.01	0.00	-0.08	-0.04	0.00
9	6	-0.03	0.01	0.00	0.01	0.02	-0.01	-0.10	-0.02	0.03
10	6	0.06	0.04	-0.01	-0.01	0.00	0.00	0.19	-0.03	0.00
11	1	-0.14	0.14	-0.04	0.02	0.00	0.00	-0.18	-0.03	-0.02
12	8	-0.02	0.09	0.01	-0.01	0.01	0.00	0.10	-0.16	-0.02
13	8	0.06	-0.01	-0.02	0.01	0.00	0.00	-0.17	0.00	0.07
14	6	0.10	0.04	0.00	-0.01	0.00	0.00	-0.07	-0.16	0.05
15	6	0.10	-0.18	0.05	0.00	0.00	0.01	0.02	-0.01	-0.02
16	8	0.04	0.01	0.04	-0.01	0.00	-0.01	0.08	0.06	0.09
17	8	0.04	-0.04	-0.04	0.00	0.01	0.00	0.08	-0.07	-0.11
18	1	0.05	-0.15	0.01	-0.01	0.02	0.01	0.19	0.05	-0.07
19	1	0.14	-0.08	0.00	0.02	-0.01	0.00	-0.39	0.18	0.07
20	1	-0.03	0.12	-0.01	-0.01	0.00	0.00	-0.18	-0.10	0.03
21	8	0.14	0.21	0.03	0.01	0.00	0.00	0.01	0.00	0.01
22	8	-0.19	-0.05	-0.05	0.00	0.00	0.00	-0.01	0.00	0.01
23	1	-0.60	-0.40	-0.26	-0.02	-0.01	0.00	-0.03	-0.02	-0.05
24	8	0.00	0.00	0.00	0.00	0.00	0.00	0.02	0.01	0.01
25	1	-0.08	0.01	-0.02	-0.07	0.01	-0.02	-0.48	0.12	-0.22
26	7	0.00	0.00	0.00	0.01	0.24	-0.12	0.00	0.01	0.00
27	1	-0.01	0.02	0.01	0.00	-0.14	0.06	-0.02	0.00	0.03
28	7	0.00	0.00	0.00	-0.01	-0.34	0.17	0.01	-0.01	0.00
29	1	0.03	-0.03	-0.09	-0.01	-0.13	0.09	0.08	-0.05	-0.25
30	6	0.00	0.00	0.01	0.01	0.28	-0.14	0.00	0.01	0.01
31	1	0.00	-0.01	0.01	0.01	0.65	-0.32	0.00	0.02	0.00
32	6	0.00	0.01	0.00	0.00	-0.16	0.08	-0.01	0.00	0.00
33	1	0.00	0.01	-0.01	0.00	-0.24	0.12	-0.01	-0.01	0.00
34	6	0.00	0.00	0.00	0.00	-0.02	0.01	-0.01	0.00	0.00
35	1	0.00	-0.01	0.00	0.00	-0.01	0.01	-0.01	-0.01	0.00
36	1	0.00	0.01	0.00	0.00	0.00	0.00	-0.02	-0.05	-0.01

	40	41	42
	A	A	A
Frequencies --	730.5715	756.2342	757.1292
Red. masses --	5.4394	2.6835	4.6255
Frc consts --	1.7105	0.9042	1.5622

IR Inten -- 12.9042 66.8115 24.5966
Raman Activ -- 2.7741 9.7700 5.9212
Depolar (P) -- 0.0443 0.0269 0.0898
Depolar (U) -- 0.0849 0.0523 0.1649

Atom	AN	X	Y	Z	X	Y	Z	X	Y	Z
1	8	0.00	0.01	-0.01	-0.02	-0.03	-0.01	0.00	-0.01	-0.01
2	1	-0.05	0.01	0.02	0.33	0.52	0.32	0.06	0.15	0.10
3	1	0.06	-0.01	0.05	0.08	0.06	0.07	0.08	0.04	0.07
4	6	-0.04	-0.02	-0.08	0.00	-0.03	0.03	0.08	0.00	0.01
5	6	-0.01	0.01	0.26	-0.06	-0.01	-0.01	0.05	0.04	0.09
6	6	-0.02	0.06	0.10	0.07	0.02	-0.01	0.11	-0.09	0.07
7	6	-0.11	-0.02	-0.23	-0.08	-0.08	0.01	-0.04	0.05	-0.20
8	6	0.04	-0.01	-0.10	-0.03	-0.01	0.02	-0.05	-0.02	-0.03
9	6	-0.05	-0.25	0.09	-0.08	0.15	-0.06	0.08	0.25	-0.15
10	6	0.04	-0.01	-0.08	0.12	0.01	0.02	-0.10	0.03	0.00
11	1	-0.02	0.06	0.04	0.06	0.00	-0.09	0.17	-0.11	-0.21
12	8	-0.01	0.02	0.08	-0.09	0.08	0.03	0.04	-0.05	0.04
13	8	0.04	0.00	0.04	0.07	-0.05	-0.05	-0.05	0.03	0.05
14	6	0.06	0.06	0.11	-0.01	-0.05	0.01	-0.16	-0.02	0.06
15	6	0.06	0.01	-0.25	-0.03	0.05	-0.03	0.12	-0.08	-0.11
16	8	0.07	0.06	-0.02	0.02	0.01	0.10	-0.02	-0.09	-0.03
17	8	-0.06	0.02	-0.09	0.03	-0.07	-0.06	-0.02	-0.01	0.09
18	1	0.04	-0.13	-0.30	0.13	0.06	-0.08	-0.10	0.20	-0.40
19	1	0.16	-0.09	0.12	0.31	-0.24	0.21	-0.03	0.01	0.21
20	1	0.05	0.06	0.06	-0.07	-0.03	-0.08	-0.17	-0.07	-0.17
21	8	-0.07	-0.03	0.07	-0.05	-0.03	-0.01	0.08	0.04	0.07
22	8	0.02	0.05	0.09	0.04	0.04	0.01	-0.08	-0.03	0.03
23	1	0.28	0.07	-0.34	0.16	0.13	0.05	-0.20	-0.21	-0.25
24	8	-0.01	-0.01	-0.01	0.00	-0.01	-0.02	0.00	-0.01	-0.01
25	1	0.38	-0.10	0.18	-0.01	-0.06	0.08	0.06	-0.04	0.05
26	7	0.00	0.01	0.00	0.00	0.00	0.00	0.00	0.00	0.00
27	1	0.01	0.12	-0.05	-0.01	-0.06	0.05	0.00	-0.13	0.05
28	7	0.00	-0.02	0.01	0.00	0.01	-0.01	0.00	0.02	-0.01
29	1	-0.07	0.07	-0.11	0.02	-0.14	-0.14	-0.03	-0.08	0.21
30	6	0.00	0.01	-0.01	0.00	0.00	0.01	0.00	-0.01	0.00
31	1	0.01	0.01	-0.01	0.00	0.00	0.01	0.00	0.00	0.00
32	6	0.00	0.00	0.00	0.00	0.00	0.00	0.00	-0.01	0.00
33	1	0.00	-0.03	0.01	0.00	0.01	-0.01	0.00	0.06	-0.02
34	6	0.00	-0.01	0.00	0.00	0.00	0.00	0.00	0.00	0.00
35	1	-0.01	-0.01	0.00	-0.01	0.00	0.00	0.01	0.03	-0.01
36	1	0.01	0.06	0.01	-0.05	0.13	-0.02	0.00	0.03	0.00

43 44 45
A A A
Frequencies -- 770.3337 789.9819 796.3533
Red. masses -- 1.1848 1.4306 1.7481
Frc consts -- 0.4142 0.5260 0.6532
IR Inten -- 38.8178 335.8789 38.1649
Raman Activ -- 0.3213 2.1504 1.0217
Depolar (P) -- 0.6637 0.3045 0.6559
Depolar (U) -- 0.7978 0.4669 0.7922

Atom	AN	X	Y	Z	X	Y	Z	X	Y	Z
1	8	0.00	0.00	0.00	-0.01	-0.03	-0.02	0.01	0.02	0.01
2	1	0.02	0.03	0.02	0.32	0.54	0.34	-0.18	-0.31	-0.19
3	1	0.01	0.00	0.01	0.14	0.10	0.12	-0.08	-0.06	-0.07
4	6	0.00	0.00	0.00	0.00	0.02	0.01	0.00	-0.02	-0.04
5	6	0.00	0.00	0.00	0.02	0.03	0.06	0.00	0.04	0.16
6	6	0.00	0.00	0.00	-0.02	-0.01	-0.01	0.02	-0.01	-0.02
7	6	0.00	0.00	0.00	0.03	0.03	-0.01	-0.01	-0.02	0.02
8	6	0.00	0.00	0.00	0.01	0.00	-0.05	-0.01	-0.02	-0.04
9	6	0.00	0.01	0.00	0.01	-0.04	0.00	0.00	0.10	-0.07

10	6	0.00	0.00	0.00	-0.04	-0.02	-0.04	0.02	-0.01	-0.09
11	1	0.00	0.00	-0.01	-0.02	0.03	0.19	0.05	0.08	0.40
12	8	0.00	0.00	0.00	0.04	-0.02	-0.01	-0.02	0.01	0.00
13	8	0.00	0.00	0.00	-0.06	0.01	0.00	0.03	-0.01	0.00
14	6	0.00	0.00	0.00	0.01	0.01	-0.02	-0.02	-0.02	-0.02
15	6	0.00	0.00	0.00	-0.02	0.00	0.08	-0.01	0.01	0.01
16	8	0.00	-0.01	0.00	0.01	0.00	-0.02	0.03	-0.02	0.02
17	8	0.00	0.00	0.00	-0.02	0.01	0.00	-0.02	-0.04	-0.02
18	1	0.00	0.00	-0.01	-0.04	0.03	0.32	0.02	0.15	0.52
19	1	0.00	0.00	0.02	-0.01	-0.04	0.31	0.00	0.03	-0.18
20	1	0.00	0.00	-0.01	0.03	0.06	0.28	-0.05	0.09	0.43
21	8	0.00	0.00	0.00	0.02	0.00	-0.02	0.00	-0.01	-0.01
22	8	0.00	0.00	0.00	-0.01	-0.02	-0.02	0.01	0.01	0.00
23	1	-0.01	-0.01	0.00	-0.06	-0.03	0.04	0.02	0.02	0.01
24	8	0.00	0.00	0.00	-0.01	-0.01	-0.02	0.00	0.01	0.01
25	1	0.00	0.00	0.01	0.16	-0.09	0.15	-0.11	0.06	-0.09
26	7	0.00	-0.03	0.02	0.00	0.00	0.00	0.00	0.00	0.00
27	1	0.00	0.19	-0.10	0.00	0.03	-0.02	0.00	-0.14	0.07
28	7	0.00	-0.03	0.01	0.00	0.00	0.00	0.00	0.01	0.00
29	1	0.00	-0.01	0.01	-0.02	0.02	0.02	-0.02	-0.04	0.01
30	6	0.00	0.04	-0.02	0.00	0.00	0.00	0.00	-0.01	0.00
31	1	-0.01	-0.22	0.11	0.00	0.00	0.00	0.00	-0.01	0.00
32	6	0.00	0.08	-0.04	0.00	0.00	0.00	0.00	0.00	0.00
33	1	-0.01	-0.74	0.35	0.00	0.03	-0.01	0.00	-0.04	0.02
34	6	0.00	0.06	-0.03	0.00	-0.01	0.00	0.00	0.01	-0.01
35	1	-0.01	-0.40	0.20	0.00	0.02	-0.01	-0.01	-0.05	0.02
36	1	0.00	0.01	0.00	-0.04	0.12	-0.01	0.02	-0.07	0.01

	46	47	48
	A	A	A
Frequencies --	808.5027	817.0078	832.3006
Red. masses --	5.8145	1.1793	6.0272
Frc consts --	2.2394	0.4638	2.4600
IR Inten --	20.4804	71.6444	13.8389
Raman Activ --	0.4271	0.1264	4.1935
Depolar (P) --	0.7096	0.6618	0.0821
Depolar (U) --	0.8302	0.7965	0.1517

Atom	AN	X	Y	Z	X	Y	Z	X	Y	Z
1	8	0.01	0.02	0.00	0.00	0.00	0.00	0.00	0.01	0.00
2	1	-0.22	-0.31	-0.19	-0.03	-0.05	-0.03	-0.06	-0.10	-0.06
3	1	-0.03	-0.05	-0.03	-0.01	-0.01	-0.01	-0.01	-0.01	-0.01
4	6	-0.01	0.05	0.20	0.00	0.00	0.01	0.10	-0.07	0.03
5	6	0.00	-0.01	-0.02	0.00	0.01	0.01	0.06	0.01	-0.02
6	6	0.01	0.03	0.03	0.01	0.00	0.00	0.10	-0.22	0.04
7	6	-0.14	-0.02	-0.36	-0.01	0.00	-0.01	0.00	0.03	-0.05
8	6	-0.01	-0.06	-0.19	-0.01	0.00	-0.01	0.09	0.08	-0.04
9	6	0.01	-0.02	0.01	0.00	0.03	-0.02	-0.23	0.04	0.05
10	6	0.08	0.01	0.03	0.01	0.00	-0.01	-0.24	0.01	0.01
11	1	-0.05	-0.09	-0.43	0.01	0.00	0.01	0.32	-0.13	-0.03
12	8	0.01	0.02	0.10	0.00	0.00	0.01	0.03	-0.03	0.00
13	8	0.05	-0.02	0.07	0.01	0.00	0.00	-0.02	0.04	0.02
14	6	0.01	-0.02	-0.01	-0.01	-0.01	0.00	0.09	0.20	-0.03
15	6	-0.14	0.06	0.35	0.00	0.00	0.01	0.01	-0.03	0.06
16	8	-0.01	0.00	-0.02	0.00	-0.02	0.01	0.00	0.11	0.22
17	8	0.00	0.01	0.02	0.00	0.00	0.00	-0.02	-0.13	-0.23
18	1	0.08	-0.03	-0.18	0.01	0.03	0.04	-0.24	0.02	-0.07
19	1	0.10	-0.06	-0.02	0.01	0.00	-0.02	-0.05	0.07	0.00
20	1	-0.03	0.05	0.19	-0.01	0.00	0.04	0.31	0.06	-0.06
21	8	0.02	-0.03	-0.10	0.00	0.00	0.00	0.04	0.02	-0.01
22	8	0.05	0.01	-0.08	0.00	0.00	0.00	-0.03	-0.05	-0.02
23	1	-0.04	0.06	0.22	0.00	0.00	0.01	-0.09	-0.09	-0.01

24	8	0.00	0.00	0.01	0.00	0.00	0.00	0.01	0.01	0.01
25	1	0.06	0.01	-0.01	-0.03	0.01	-0.02	-0.15	0.04	-0.07
26	7	0.00	0.00	0.00	0.00	-0.02	0.01	0.00	0.00	0.01
27	1	0.00	-0.18	0.08	0.02	0.83	-0.41	-0.01	0.04	0.02
28	7	0.00	0.01	0.00	0.00	-0.02	0.01	0.01	0.00	0.00
29	1	0.01	0.03	0.02	0.00	-0.07	0.04	-0.04	-0.18	-0.45
30	6	0.00	-0.01	0.00	0.00	0.04	-0.02	0.01	0.01	0.01
31	1	0.00	0.01	-0.01	0.00	-0.07	0.03	0.01	0.01	0.01
32	6	0.00	0.00	0.00	0.00	0.02	-0.01	-0.01	0.00	0.00
33	1	0.00	-0.03	0.01	0.00	0.08	-0.04	0.00	0.00	-0.02
34	6	0.00	0.02	-0.01	0.00	-0.08	0.04	-0.01	0.00	0.00
35	1	0.00	-0.06	0.03	0.01	0.29	-0.14	-0.01	0.01	-0.01
36	1	0.03	-0.05	0.01	0.00	-0.01	0.00	0.00	-0.02	0.00

	49	50	51
	A	A	A
Frequencies --	866.0874	888.3743	911.6112
Red. masses --	3.6798	1.1975	6.5056
Frc consts --	1.6263	0.5568	3.1853
IR Inten --	2.5492	14.3651	5.5548
Raman Activ --	0.8781	0.2935	3.6509
Depolar (P) --	0.7452	0.7301	0.7315
Depolar (U) --	0.8540	0.8440	0.8449

Atom	AN	X	Y	Z	X	Y	Z	X	Y	Z
1	8	0.00	0.00	0.00	0.00	0.00	0.00	0.00	0.00	0.01
2	1	-0.03	-0.04	-0.02	0.00	0.00	0.00	0.06	0.04	0.01
3	1	0.01	-0.01	0.01	0.00	0.00	0.00	-0.06	-0.03	-0.04
4	6	0.01	0.06	0.21	0.00	0.00	0.00	0.05	0.22	-0.07
5	6	0.00	-0.03	-0.19	0.00	0.01	0.00	0.01	0.30	-0.04
6	6	0.02	-0.03	-0.09	0.00	0.00	0.00	0.12	-0.07	0.03
7	6	-0.07	-0.01	-0.19	0.00	0.00	0.00	-0.08	-0.08	0.10
8	6	0.00	0.07	0.20	0.00	0.00	0.00	-0.05	0.24	-0.09
9	6	-0.01	-0.04	0.04	0.00	0.00	0.00	0.00	-0.17	0.08
10	6	0.00	-0.01	-0.05	0.00	0.00	0.00	0.02	-0.01	0.00
11	1	0.03	0.04	0.25	0.00	0.00	0.00	-0.05	-0.17	0.00
12	8	0.01	0.01	0.05	0.00	0.00	0.00	-0.15	0.05	0.04
13	8	0.02	-0.02	0.03	0.00	0.00	0.00	0.12	-0.17	-0.05
14	6	-0.01	-0.02	-0.10	0.00	0.00	0.00	-0.13	-0.08	0.03
15	6	0.06	-0.03	-0.17	0.00	0.00	0.00	0.07	-0.02	0.07
16	8	-0.02	0.02	0.00	0.00	-0.01	0.00	0.02	0.03	-0.03
17	8	0.02	0.02	0.01	0.00	0.00	0.00	-0.02	0.03	-0.02
18	1	0.00	0.17	0.74	0.00	0.01	-0.01	0.03	0.36	-0.09
19	1	0.05	-0.05	0.04	0.00	0.00	0.00	0.19	-0.23	-0.26
20	1	-0.01	0.06	0.31	0.00	0.00	0.00	0.02	-0.18	0.02
21	8	-0.01	0.01	0.04	0.00	0.00	0.00	0.12	0.01	0.03
22	8	-0.02	-0.02	0.03	0.00	0.00	0.00	-0.10	-0.16	-0.03
23	1	0.01	-0.03	-0.05	0.00	0.00	0.00	-0.26	-0.28	-0.06
24	8	0.00	0.00	0.00	0.00	0.00	0.00	0.00	0.00	0.00
25	1	0.04	-0.01	0.02	0.00	0.00	0.00	0.05	-0.01	0.02
26	7	0.00	0.00	0.00	0.00	-0.02	0.01	0.00	0.00	0.00
27	1	-0.01	-0.01	0.01	0.01	0.36	-0.18	0.01	-0.05	0.02
28	7	0.00	0.00	0.00	0.00	-0.01	0.00	-0.01	0.00	0.00
29	1	0.02	0.02	-0.03	0.00	0.00	0.00	-0.01	0.08	-0.02
30	6	0.00	0.00	0.00	0.00	-0.06	0.03	0.00	0.00	0.00
31	1	0.00	0.02	-0.01	0.01	0.42	-0.21	0.01	0.00	-0.01
32	6	0.00	0.00	0.00	0.00	-0.04	0.02	0.00	0.00	0.00
33	1	0.00	0.01	-0.01	0.01	0.32	-0.15	0.00	-0.03	0.02
34	6	0.00	0.01	0.00	0.00	0.09	-0.05	0.00	-0.01	0.00
35	1	0.00	-0.03	0.02	-0.01	-0.62	0.30	-0.01	0.04	-0.02
36	1	0.01	0.00	0.00	0.00	0.00	0.00	0.00	0.02	0.00

52	53	54

A A A

Frequencies -- 923.1983 940.8096 959.6346

Red. masses -- 1.3430 6.3520 3.0356

Frc consts -- 0.6744 3.3126 1.6470

IR Inten -- 8.2741 3.2581 38.6616

Raman Activ -- 0.1079 5.3334 2.2040

Depolar (P) -- 0.5557 0.7455 0.3234

Depolar (U) -- 0.7144 0.8542 0.4887

Atom	AN	X	Y	Z	X	Y	Z	X	Y	Z
1	8	0.00	0.00	0.00	0.00	0.00	0.00	0.00	0.00	0.00
2	1	0.00	0.00	0.00	0.00	0.00	0.00	0.00	0.00	0.00
3	1	0.00	0.00	0.00	0.00	0.00	0.00	0.00	0.00	0.00
4	6	0.00	0.00	0.00	0.00	0.00	0.00	0.00	0.00	0.00
5	6	0.00	0.00	0.00	0.00	0.00	0.00	0.00	0.00	-0.01
6	6	0.00	0.00	0.00	0.00	0.00	0.00	0.00	0.00	0.00
7	6	0.00	0.00	0.00	0.00	0.00	0.00	0.00	0.00	0.00
8	6	0.00	0.00	0.00	0.00	0.00	0.00	0.00	0.00	0.00
9	6	0.00	0.00	0.00	0.00	-0.01	-0.01	0.00	0.00	0.00
10	6	0.00	0.00	0.00	0.00	0.00	0.00	0.00	0.00	0.00
11	1	0.00	0.00	0.00	0.00	0.00	0.00	0.00	0.00	0.00
12	8	0.00	0.00	0.00	0.00	0.00	0.00	0.00	0.00	0.00
13	8	0.00	0.00	0.00	0.00	0.00	0.00	0.00	0.00	0.00
14	6	0.00	0.00	0.00	0.00	0.00	0.00	0.00	0.00	0.00
15	6	0.00	0.00	0.00	0.00	0.00	0.00	0.00	0.00	0.00
16	8	0.00	0.00	0.00	0.00	0.00	-0.01	-0.01	0.00	0.01
17	8	0.00	0.00	0.00	-0.01	0.01	0.02	0.02	0.00	-0.01
18	1	0.00	0.00	0.00	0.00	0.00	0.00	0.00	0.00	0.00
19	1	0.00	0.00	0.00	0.00	0.00	0.00	0.00	0.00	0.01
20	1	0.00	0.00	0.00	0.00	0.00	0.00	0.00	0.00	0.01
21	8	0.00	0.00	0.00	0.00	0.00	0.00	0.00	0.00	0.00
22	8	0.00	0.00	0.00	0.00	0.00	0.00	0.00	0.00	0.00
23	1	0.00	0.00	0.00	0.00	0.00	0.00	0.00	0.00	0.00
24	8	0.00	0.00	0.00	0.00	0.00	0.00	0.00	0.00	0.00
25	1	0.01	0.00	0.00	0.01	0.00	0.00	0.00	0.00	0.00
26	7	0.00	0.01	-0.01	0.19	0.00	0.00	0.15	0.07	0.14
27	1	0.00	0.00	0.00	0.05	0.10	0.20	0.32	-0.03	-0.05
28	7	0.00	0.01	-0.01	0.15	0.02	0.04	-0.19	0.06	0.12
29	1	0.00	0.04	-0.02	-0.01	0.00	-0.01	0.00	-0.01	-0.02
30	6	0.00	-0.12	0.06	-0.02	0.14	0.29	-0.11	-0.07	-0.14
31	1	0.01	0.74	-0.36	0.14	0.13	0.28	0.27	-0.08	-0.20
32	6	0.00	0.09	-0.04	-0.42	-0.01	-0.03	0.02	-0.03	-0.06
33	1	-0.01	-0.39	0.17	-0.44	0.03	0.03	-0.03	0.23	0.54
34	6	0.00	-0.05	0.02	0.07	-0.16	-0.33	0.10	-0.05	-0.10
35	1	0.01	0.28	-0.13	0.24	-0.15	-0.29	-0.44	-0.10	-0.25
36	1	0.00	0.00	0.00	0.00	0.00	0.00	0.00	0.00	0.00

55 56 57

A A A

Frequencies -- 984.6249 1024.9010 1044.5780

Red. masses -- 1.3972 1.3520 1.0813

Frc consts -- 0.7981 0.8367 0.6951

IR Inten -- 0.2426 3.0796 100.9580

Raman Activ -- 0.0845 0.1433 1.3168

Depolar (P) -- 0.6363 0.3085 0.7298

Depolar (U) -- 0.7777 0.4715 0.8438

Atom	AN	X	Y	Z	X	Y	Z	X	Y	Z
1	8	0.00	0.00	0.00	0.00	0.00	0.00	0.01	0.02	-0.02
2	1	0.01	0.01	0.00	0.02	0.02	0.01	-0.29	-0.27	-0.15
3	1	0.00	0.00	0.00	-0.01	0.00	-0.01	0.15	0.02	0.12
4	6	0.00	-0.01	-0.04	0.00	0.00	-0.02	0.00	0.00	0.01
5	6	0.00	0.00	0.00	0.00	-0.01	-0.01	0.00	0.01	0.00

6	6	0.00	0.01	0.11	0.00	0.01	0.10	0.00	0.00	0.00
7	6	0.01	0.00	0.03	0.00	0.00	0.01	0.01	-0.02	0.03
8	6	0.00	0.01	0.04	0.00	-0.01	-0.01	0.00	0.01	0.00
9	6	0.00	0.00	0.00	0.00	0.00	0.00	0.00	-0.01	0.00
10	6	0.00	0.01	0.02	0.00	-0.02	-0.12	0.00	0.00	0.00
11	1	-0.05	-0.15	-0.63	-0.05	-0.13	-0.57	0.01	0.00	-0.04
12	8	0.00	0.00	-0.01	0.00	0.00	0.00	-0.01	0.01	-0.01
13	8	0.00	0.00	0.00	0.00	0.00	0.00	-0.01	0.00	-0.04
14	6	0.00	-0.02	-0.13	0.00	0.02	0.07	0.00	-0.01	0.00
15	6	0.01	0.00	-0.03	0.00	0.00	0.01	0.00	0.00	0.00
16	8	0.00	0.00	0.00	0.00	0.00	0.00	0.00	0.00	0.00
17	8	0.00	0.00	0.00	0.00	0.00	0.00	0.00	0.00	0.00
18	1	0.00	-0.01	-0.09	0.01	0.14	0.64	0.00	0.02	0.03
19	1	0.00	0.00	-0.01	0.00	0.01	-0.05	0.12	-0.11	0.86
20	1	-0.03	0.18	0.71	0.01	-0.09	-0.43	0.02	-0.02	-0.01
21	8	0.00	0.00	0.01	0.00	0.00	0.00	0.00	0.00	0.00
22	8	0.00	0.00	0.00	0.00	0.01	0.00	0.00	-0.01	0.00
23	1	0.01	0.01	0.00	-0.01	0.00	0.00	0.00	-0.01	0.00
24	8	0.00	0.00	0.00	0.00	0.00	0.00	0.01	0.00	0.01
25	1	0.00	0.00	0.00	0.00	0.00	0.00	-0.04	0.01	-0.03
26	7	0.00	0.00	0.00	0.00	0.00	0.00	0.00	0.00	0.00
27	1	0.00	0.00	0.00	0.00	0.00	0.00	0.00	0.00	0.00
28	7	0.00	0.00	0.00	0.00	0.00	0.00	0.00	0.00	0.00
29	1	0.00	0.00	0.00	0.00	-0.01	0.00	0.00	-0.03	0.01
30	6	0.00	0.00	0.00	0.00	0.00	0.00	0.00	0.00	0.00
31	1	0.00	0.00	0.00	0.00	0.00	0.00	0.00	0.00	0.00
32	6	0.00	0.00	0.00	0.00	0.00	0.00	0.00	0.00	0.00
33	1	0.00	0.00	0.00	0.00	0.00	0.00	0.00	0.00	0.00
34	6	0.00	0.00	0.00	0.00	0.00	0.00	0.00	0.00	0.00
35	1	0.00	0.00	0.00	0.00	0.00	0.00	0.00	0.00	0.00
36	1	0.00	0.00	0.00	0.00	0.00	0.00	0.00	-0.02	0.00

	58	59	60
	A	A	A
Frequencies --	1056.2368	1069.4986	1079.2017
Red. masses --	1.0841	1.3967	1.5100
Frc consts --	0.7126	0.9412	1.0362
IR Inten --	79.8786	12.1252	101.9325
Raman Activ --	0.3958	2.7375	1.6672
Depolar (P) --	0.5790	0.5858	0.7481
Depolar (U) --	0.7334	0.7388	0.8559

Atom	AN	X	Y	Z	X	Y	Z	X	Y	Z
1	8	0.00	0.00	0.00	0.00	0.00	0.00	0.00	0.00	0.00
2	1	-0.01	-0.01	0.00	0.00	0.00	0.00	-0.01	-0.01	0.00
3	1	0.00	0.00	0.00	0.00	0.00	0.00	0.00	0.00	0.00
4	6	0.00	0.00	0.00	0.00	0.00	0.00	0.00	0.01	0.00
5	6	0.00	0.00	0.00	0.00	0.00	0.00	-0.01	0.00	0.00
6	6	0.00	0.00	0.00	0.00	0.00	0.00	0.00	-0.01	0.00
7	6	0.00	0.00	0.00	0.00	0.00	0.00	0.00	0.00	0.00
8	6	0.00	0.01	0.00	0.00	0.00	0.00	0.00	0.00	0.00
9	6	0.00	0.02	-0.01	0.00	0.00	0.00	-0.01	0.00	-0.01
10	6	0.00	0.00	0.00	0.00	0.00	0.00	0.00	0.00	0.00
11	1	0.00	0.00	0.00	0.00	0.00	0.00	-0.01	-0.02	0.01
12	8	0.00	0.00	0.00	0.00	0.00	0.00	0.00	0.00	0.00
13	8	0.00	0.00	0.00	0.00	0.00	0.00	0.00	0.00	0.00
14	6	0.00	0.00	0.00	0.00	0.00	0.00	0.01	0.01	0.00
15	6	0.00	0.00	0.00	0.00	0.00	0.00	0.00	0.00	0.00
16	8	0.00	-0.02	0.00	0.00	0.00	0.00	0.00	0.00	-0.01
17	8	-0.01	-0.04	0.03	-0.01	0.00	0.00	0.00	0.01	0.01
18	1	0.00	0.00	0.00	0.00	0.00	0.00	0.00	0.00	0.00
19	1	0.00	0.00	0.03	0.00	0.00	0.00	0.00	0.00	0.01

20	1	0.01	-0.01	0.00	0.00	0.00	0.00	-0.01	0.02	0.00
21	8	0.00	0.00	0.00	0.00	0.00	0.00	0.00	0.00	0.00
22	8	0.00	0.00	0.00	0.00	0.00	0.00	0.00	0.00	0.00
23	1	0.01	0.00	0.00	0.00	0.00	0.00	-0.01	0.00	0.00
24	8	0.00	0.00	0.00	0.00	0.00	0.00	0.00	0.00	0.00
25	1	0.08	-0.01	0.03	0.00	0.00	0.00	0.00	0.00	0.00
26	7	0.00	0.00	0.00	-0.09	-0.02	-0.05	0.01	0.02	0.04
27	1	0.01	0.04	-0.03	-0.20	0.04	0.07	-0.04	0.05	0.10
28	7	0.00	-0.03	0.01	0.05	0.00	0.01	0.09	-0.05	-0.11
29	1	0.05	0.85	-0.50	0.01	0.04	0.02	0.00	-0.03	0.01
30	6	0.00	0.01	0.00	0.07	-0.03	-0.07	0.01	0.01	0.03
31	1	-0.01	-0.03	0.02	0.45	-0.07	-0.12	-0.38	0.05	0.08
32	6	0.00	0.00	0.00	-0.05	0.04	0.07	0.05	0.03	0.06
33	1	0.00	0.02	-0.02	-0.11	0.34	0.72	0.03	0.17	0.37
34	6	0.00	0.00	0.00	-0.01	-0.01	-0.01	-0.07	-0.02	-0.04
35	1	-0.03	0.01	-0.01	0.21	0.02	0.04	-0.75	-0.09	-0.21
36	1	-0.01	-0.04	-0.01	0.00	0.00	0.00	0.00	0.00	0.00

	61	62	63
	A	A	A
Frequencies --	1096.7671	1154.7690	1172.8348
Red. masses --	3.5516	3.7316	2.6700
Frc consts --	2.5171	2.9318	2.1639
IR Inten --	29.6736	64.9008	76.8147
Raman Activ --	26.0289	1.9865	9.6927
Depolar (P) --	0.0707	0.0378	0.2282
Depolar (U) --	0.1320	0.0728	0.3716

Atom	AN	X	Y	Z	X	Y	Z	X	Y	Z
1	8	0.00	0.00	0.00	0.00	0.00	0.00	0.00	0.00	0.00
2	1	0.02	0.02	0.01	0.01	0.02	0.01	-0.01	-0.01	0.00
3	1	-0.01	0.00	-0.01	-0.01	0.00	-0.01	0.01	0.00	0.01
4	6	0.04	-0.08	0.01	-0.09	-0.16	0.04	0.04	0.05	-0.01
5	6	0.20	-0.01	-0.01	0.14	-0.04	0.00	0.19	0.03	-0.02
6	6	-0.05	0.25	-0.03	-0.09	-0.06	0.00	-0.08	-0.01	0.00
7	6	0.00	-0.03	-0.02	0.02	-0.06	-0.01	-0.01	0.02	0.01
8	6	0.05	0.07	-0.01	-0.08	0.15	-0.04	0.05	-0.07	0.03
9	6	0.02	0.02	0.04	0.05	0.03	0.04	0.06	0.02	0.06
10	6	-0.09	0.00	-0.01	0.20	-0.01	0.00	0.01	0.05	-0.01
11	1	0.39	0.48	-0.18	-0.51	-0.27	0.10	-0.58	-0.25	0.11
12	8	0.01	0.00	0.00	0.06	0.01	-0.02	-0.01	0.00	0.00
13	8	-0.02	0.04	0.01	-0.04	0.08	0.02	0.01	-0.03	-0.01
14	6	-0.07	-0.24	0.04	-0.09	0.08	-0.01	-0.05	-0.04	0.01
15	6	0.01	0.06	0.01	0.05	0.13	-0.01	-0.05	-0.14	0.00
16	8	-0.04	0.03	0.05	-0.03	0.02	0.03	-0.04	0.02	0.04
17	8	-0.05	-0.03	-0.07	-0.05	-0.03	-0.06	-0.06	-0.04	-0.08
18	1	-0.11	-0.03	0.04	0.22	-0.06	0.01	0.02	0.43	-0.10
19	1	0.00	0.02	-0.07	0.00	0.04	-0.03	0.01	-0.03	0.04
20	1	0.28	-0.46	0.12	-0.46	0.31	-0.11	-0.26	0.08	-0.02
21	8	0.01	0.00	0.00	0.08	-0.02	0.03	-0.06	0.01	-0.02
22	8	-0.04	-0.04	-0.01	-0.09	-0.11	-0.02	0.10	0.10	0.03
23	1	0.06	0.03	0.02	0.17	0.09	0.06	-0.27	-0.18	-0.08
24	8	0.00	0.00	0.00	0.00	0.00	0.00	0.00	0.00	0.00
25	1	0.02	0.00	0.01	0.02	0.00	0.01	0.03	0.00	0.01
26	7	0.00	0.00	0.00	0.01	-0.01	-0.01	-0.03	0.00	0.00
27	1	-0.01	0.00	0.01	0.02	-0.02	-0.04	-0.11	0.05	0.10
28	7	0.01	-0.01	-0.01	0.00	0.00	0.00	0.00	0.01	0.02
29	1	-0.05	-0.06	-0.04	-0.03	-0.03	0.00	-0.04	0.03	-0.01
30	6	0.01	0.00	0.01	0.01	0.01	0.01	-0.02	0.01	0.01
31	1	-0.04	0.01	0.02	0.04	0.00	0.01	-0.18	0.02	0.04
32	6	0.01	0.00	0.01	0.00	0.00	0.01	0.04	-0.01	-0.01
33	1	0.01	0.02	0.05	0.00	0.00	0.00	0.04	0.02	0.05

34	6	-0.01	0.00	-0.01	-0.01	0.00	0.00	0.03	-0.02	-0.04
35	1	-0.11	-0.01	-0.03	-0.08	-0.01	-0.02	0.09	-0.01	-0.03
36	1	0.00	0.00	0.00	0.00	0.00	0.00	0.00	0.01	0.00

64 65 66

A A A

Frequencies -- 1177.9496 1193.9519 1217.3571

Red. masses -- 1.7934 1.4350 3.9718

Frc consts -- 1.4661 1.2053 3.4680

IR Inten -- 58.3831 11.9914 52.6600

Raman Activ -- 10.3177 8.0188 42.0062

Depolar (P) -- 0.1254 0.4952 0.0595

Depolar (U) -- 0.2228 0.6624 0.1123

Atom	AN	X	Y	Z	X	Y	Z	X	Y	Z
1	8	0.00	0.00	0.00	0.00	0.00	0.00	0.00	0.00	0.00
2	1	0.00	0.00	0.00	0.01	0.01	0.01	0.00	0.00	0.00
3	1	0.00	0.00	0.00	-0.01	0.00	-0.01	0.00	0.00	0.00
4	6	0.01	0.01	0.00	-0.07	-0.07	0.02	0.00	-0.02	0.00
5	6	0.03	0.01	0.00	-0.06	0.01	0.00	0.03	0.00	0.00
6	6	-0.01	0.00	0.00	-0.02	-0.04	0.01	-0.01	0.01	0.00
7	6	0.00	0.00	0.00	0.01	-0.05	0.00	0.00	-0.01	0.00
8	6	0.01	-0.02	0.01	0.02	0.01	0.00	-0.02	0.03	-0.01
9	6	0.01	0.00	0.01	-0.02	-0.02	-0.01	-0.01	0.00	-0.01
10	6	-0.01	0.01	0.00	0.01	0.08	-0.01	0.02	-0.01	0.00
11	1	-0.11	-0.05	0.02	-0.10	-0.09	0.03	-0.02	0.00	0.00
12	8	0.00	0.00	0.00	0.03	0.01	-0.01	0.01	0.00	0.00
13	8	0.00	-0.01	0.00	-0.02	0.05	0.01	0.00	0.01	0.00
14	6	0.00	-0.01	0.00	0.05	-0.04	0.01	-0.01	0.00	0.00
15	6	-0.01	-0.03	0.00	0.00	0.02	0.00	0.00	-0.02	0.00
16	8	-0.01	0.00	0.00	0.01	0.00	-0.01	0.00	0.00	0.00
17	8	-0.01	0.00	0.00	0.02	0.01	0.02	0.00	0.00	0.00
18	1	0.00	0.11	-0.03	0.04	0.83	-0.17	0.02	-0.07	0.01
19	1	0.00	-0.01	0.00	0.02	0.01	-0.03	0.00	0.00	0.00
20	1	-0.03	0.00	0.00	0.40	-0.25	0.07	-0.03	0.01	0.00
21	8	-0.02	0.00	-0.01	0.00	0.00	0.00	-0.01	0.00	0.00
22	8	0.02	0.02	0.01	-0.01	-0.01	0.00	0.02	0.01	0.01
23	1	-0.06	-0.04	-0.02	0.03	0.02	0.00	-0.11	-0.09	-0.03
24	8	0.00	0.00	0.00	0.00	0.00	0.00	0.00	0.00	0.00
25	1	-0.01	0.00	0.00	0.00	0.00	0.00	0.00	0.00	0.00
26	7	0.11	-0.02	-0.03	0.00	0.00	0.01	0.07	0.12	0.25
27	1	0.34	-0.16	-0.32	-0.01	0.00	0.01	0.00	0.19	0.39
28	7	-0.02	-0.03	-0.07	0.00	0.00	0.00	0.14	-0.08	-0.16
29	1	-0.01	-0.05	-0.05	0.01	0.00	0.01	-0.03	0.00	-0.02
30	6	0.08	0.00	0.00	0.00	0.00	0.00	-0.06	-0.10	-0.21
31	1	0.64	-0.05	-0.08	-0.02	0.00	0.00	-0.29	-0.10	-0.21
32	6	-0.11	0.03	0.06	0.00	0.00	0.00	-0.18	-0.02	-0.04
33	1	-0.10	-0.10	-0.21	0.00	0.00	0.01	-0.22	-0.02	-0.05
34	6	-0.10	0.05	0.10	0.00	0.00	0.00	0.00	0.06	0.11
35	1	-0.41	0.02	0.04	0.02	0.00	0.01	0.51	0.12	0.27
36	1	0.00	0.00	0.00	0.00	0.00	0.00	0.00	0.00	0.00

67 68 69

A A A

Frequencies -- 1224.4365 1256.0356 1315.4040

Red. masses -- 2.0248 1.7520 3.0264

Frc consts -- 1.7886 1.6285 3.0852

IR Inten -- 327.0681 0.9941 269.0708

Raman Activ -- 14.4206 0.9147 6.1299

Depolar (P) -- 0.2462 0.7474 0.3163

Depolar (U) -- 0.3952 0.8554 0.4806

Atom	AN	X	Y	Z	X	Y	Z	X	Y	Z
1	8	0.00	0.00	0.00	0.00	0.00	0.00	0.00	-0.01	0.00

2	1	0.00	-0.01	0.00	0.00	0.01	0.00	0.02	0.04	0.02
3	1	0.01	0.00	0.01	0.00	0.00	0.00	0.04	0.03	0.03
4	6	0.05	0.08	-0.02	-0.14	-0.02	0.01	-0.18	-0.02	0.00
5	6	0.02	-0.05	0.01	0.00	0.11	-0.02	0.02	-0.15	0.03
6	6	-0.01	0.01	0.00	0.03	-0.06	0.01	0.05	0.02	0.00
7	6	-0.01	0.07	0.00	0.01	-0.04	0.01	0.00	0.22	0.00
8	6	0.10	-0.13	0.03	0.13	-0.03	0.01	0.06	0.08	-0.02
9	6	0.01	0.00	0.01	0.01	-0.03	0.02	0.01	0.02	0.01
10	6	-0.07	0.02	0.00	0.01	-0.02	0.00	0.05	-0.07	0.01
11	1	-0.27	-0.12	0.04	0.60	0.22	-0.08	0.16	0.06	-0.04
12	8	-0.04	-0.01	0.01	0.03	0.01	-0.01	-0.04	-0.01	0.01
13	8	0.03	-0.06	-0.01	-0.01	0.03	0.00	0.10	-0.12	-0.03
14	6	0.00	-0.01	0.01	-0.03	-0.04	0.01	-0.06	0.05	-0.01
15	6	0.01	0.08	0.00	-0.02	-0.04	0.01	0.01	0.04	0.00
16	8	-0.01	0.00	0.01	0.00	0.00	0.00	-0.01	0.00	0.00
17	8	-0.01	0.00	-0.01	-0.01	0.00	-0.01	-0.01	-0.01	-0.01
18	1	-0.07	0.24	-0.05	0.01	0.18	-0.02	0.06	0.30	-0.05
19	1	-0.05	0.01	0.04	-0.02	0.03	0.00	-0.49	0.35	0.15
20	1	-0.15	0.06	-0.04	-0.60	0.29	-0.08	0.16	-0.08	0.03
21	8	0.05	0.01	0.02	-0.01	0.01	-0.01	-0.02	-0.01	-0.01
22	8	-0.11	-0.04	-0.04	0.00	0.02	0.00	0.02	-0.02	0.01
23	1	0.65	0.52	0.18	0.12	0.11	0.03	-0.22	-0.19	-0.06
24	8	0.00	0.00	0.00	0.00	0.00	0.00	0.00	0.00	0.00
25	1	0.00	0.00	0.00	0.00	0.00	0.00	0.00	0.00	0.00
26	7	0.01	0.01	0.02	0.00	0.00	0.00	-0.02	0.01	0.03
27	1	0.00	0.01	0.04	0.00	0.01	0.00	-0.17	0.11	0.21
28	7	0.01	-0.01	-0.01	0.00	0.00	0.00	-0.03	-0.02	-0.05
29	1	-0.01	-0.01	0.01	0.00	0.03	0.00	0.00	-0.03	0.00
30	6	0.00	-0.01	-0.02	0.00	0.00	0.00	-0.01	0.01	0.02
31	1	-0.03	-0.01	-0.02	0.00	0.00	0.00	0.29	-0.01	-0.01
32	6	-0.01	0.00	0.00	0.00	0.00	0.00	0.02	0.01	0.02
33	1	-0.02	0.00	0.00	0.00	0.00	0.00	0.04	-0.06	-0.13
34	6	0.00	0.00	0.01	0.00	0.00	0.00	0.05	-0.01	-0.02
35	1	0.04	0.01	0.02	0.00	0.00	0.00	-0.05	-0.02	-0.04
36	1	0.00	0.00	0.00	0.00	0.00	0.00	-0.01	0.01	0.00

	70	71	72
	A	A	A
Frequencies --	1318.9746	1341.5445	1353.1468
Red. masses --	1.7804	4.8842	5.3691
Frc consts --	1.8249	5.1791	5.7922
IR Inten --	27.2007	144.9293	346.0570
Raman Activ --	32.0379	23.0397	6.4257
Depolar (P) --	0.1580	0.3407	0.0931
Depolar (U) --	0.2729	0.5082	0.1704

Atom	AN	X	Y	Z	X	Y	Z	X	Y	Z
1	8	0.00	0.00	0.00	0.00	0.01	0.00	0.00	0.00	0.00
2	1	-0.01	-0.01	-0.01	-0.03	-0.04	-0.03	-0.01	0.00	-0.01
3	1	-0.01	-0.01	-0.01	-0.03	-0.03	-0.03	0.00	0.00	0.00
4	6	0.05	0.00	0.00	-0.08	0.18	-0.03	-0.09	0.12	-0.01
5	6	-0.02	0.04	-0.01	-0.02	-0.21	0.05	-0.17	-0.03	-0.01
6	6	-0.01	-0.01	0.00	0.18	0.12	-0.03	0.08	-0.04	0.01
7	6	0.00	-0.06	0.00	0.00	-0.20	0.02	0.00	-0.06	0.01
8	6	-0.03	-0.02	0.01	0.23	0.10	-0.02	-0.08	-0.07	0.01
9	6	0.02	0.01	0.02	-0.04	0.01	-0.04	0.40	0.11	0.21
10	6	-0.01	0.02	0.00	-0.06	-0.19	0.04	0.01	-0.03	0.00
11	1	-0.01	-0.01	0.01	-0.45	-0.19	0.07	0.26	0.04	-0.02
12	8	0.01	0.00	0.00	0.06	0.02	-0.02	0.02	0.01	-0.01
13	8	-0.02	0.03	0.01	-0.09	0.09	0.03	-0.02	0.02	0.00
14	6	0.02	-0.01	0.00	-0.16	0.08	-0.02	0.03	0.08	-0.01
15	6	0.00	-0.01	0.00	-0.01	-0.02	0.01	0.00	0.01	-0.01

16	8	0.00	0.00	-0.01	0.01	-0.01	0.00	-0.07	0.02	0.03
17	8	-0.01	0.00	-0.01	0.02	0.01	0.03	-0.13	-0.10	-0.19
18	1	-0.01	-0.09	0.01	-0.05	0.14	-0.03	0.01	-0.03	0.00
19	1	0.13	-0.09	-0.04	0.47	-0.36	-0.12	0.06	-0.06	0.02
20	1	-0.01	0.01	0.00	0.06	-0.05	0.03	0.47	-0.18	0.05
21	8	0.01	0.00	0.00	-0.03	0.01	-0.01	0.01	0.00	0.00
22	8	-0.01	0.00	0.00	0.01	0.00	0.00	-0.01	0.00	0.00
23	1	0.07	0.06	0.02	-0.05	-0.05	-0.01	0.03	0.03	0.01
24	8	0.00	0.00	0.00	0.00	0.00	0.00	0.00	0.00	0.00
25	1	0.00	0.00	0.00	-0.02	0.01	-0.01	0.09	0.01	0.03
26	7	-0.05	0.03	0.06	-0.01	0.00	0.01	0.04	0.00	-0.01
27	1	-0.37	0.23	0.46	-0.02	0.01	0.03	0.02	0.00	0.00
28	7	-0.07	-0.06	-0.12	-0.01	0.00	-0.01	0.02	0.00	0.01
29	1	0.01	0.00	0.00	0.01	-0.06	-0.03	0.01	0.26	0.43
30	6	-0.03	0.03	0.05	0.00	0.00	0.01	0.01	-0.02	-0.03
31	1	0.61	-0.03	-0.03	0.05	0.00	0.00	-0.16	-0.01	-0.02
32	6	0.05	0.02	0.05	0.01	0.00	0.00	-0.03	0.00	0.01
33	1	0.09	-0.13	-0.27	0.01	-0.01	-0.01	-0.04	-0.01	-0.03
34	6	0.10	-0.02	-0.04	0.01	0.00	0.00	-0.01	0.01	0.01
35	1	-0.09	-0.05	-0.09	0.00	0.00	-0.01	-0.03	0.01	0.01
36	1	0.00	0.00	0.00	0.00	-0.01	0.00	0.01	0.01	0.00

	73	74	75
	A	A	A
Frequencies --	1390.8586	1415.4398	1455.0786
Red. masses --	2.6547	2.8691	4.1258
Frc consts --	3.0257	3.3867	5.1467
IR Inten --	110.0538	54.6905	25.7234
Raman Activ --	12.5555	11.3145	35.8420
Depolar (P) --	0.1806	0.1446	0.6023
Depolar (U) --	0.3060	0.2527	0.7518

Atom	AN	X	Y	Z	X	Y	Z	X	Y	Z
1	8	0.00	0.00	0.00	0.00	0.00	0.00	0.00	0.00	0.00
2	1	-0.01	-0.01	-0.01	0.00	0.00	0.00	0.00	0.00	0.00
3	1	-0.01	-0.01	0.00	0.00	0.00	0.00	0.00	0.00	0.00
4	6	0.06	0.02	0.00	0.01	-0.01	0.00	0.01	-0.01	0.00
5	6	-0.05	0.00	0.00	0.00	0.00	0.00	0.00	0.00	0.00
6	6	-0.02	0.00	0.00	0.00	0.01	0.00	0.00	0.01	0.00
7	6	0.00	0.00	0.00	0.00	0.00	0.00	0.00	0.00	0.00
8	6	0.08	-0.09	0.02	0.01	0.01	0.00	0.01	0.01	0.00
9	6	0.02	0.00	0.01	-0.03	-0.01	-0.02	-0.02	0.00	0.00
10	6	-0.06	0.02	0.00	0.00	0.00	0.00	0.00	0.00	0.00
11	1	-0.03	0.00	0.00	-0.03	-0.01	0.00	-0.03	-0.01	0.00
12	8	0.00	0.00	0.00	0.00	0.00	0.00	0.00	0.00	0.00
13	8	-0.01	0.00	0.00	0.00	0.00	0.00	0.00	0.00	0.00
14	6	0.06	-0.04	0.01	0.00	-0.01	0.00	0.00	-0.01	0.00
15	6	0.06	0.30	-0.01	0.00	0.00	0.00	0.00	-0.01	0.00
16	8	0.00	0.00	0.00	0.00	0.00	0.01	0.00	0.00	-0.01
17	8	-0.01	0.00	-0.01	0.01	0.00	0.00	0.01	0.00	0.01
18	1	-0.06	0.13	-0.02	0.00	0.00	0.00	0.00	0.00	0.00
19	1	0.11	-0.08	-0.03	0.01	0.00	0.00	-0.01	0.01	0.00
20	1	-0.36	0.20	-0.06	-0.04	0.01	0.00	-0.03	0.01	0.00
21	8	-0.06	-0.04	-0.02	0.00	0.00	0.00	0.00	0.00	0.00
22	8	0.03	-0.09	0.02	0.00	0.00	0.00	0.00	0.00	0.00
23	1	-0.58	-0.52	-0.16	0.00	0.00	0.00	0.00	0.00	0.00
24	8	0.00	0.00	0.00	0.00	0.00	0.00	0.00	0.00	0.00
25	1	0.00	0.00	0.00	0.01	0.00	0.00	0.00	0.00	0.00
26	7	0.00	0.00	0.00	0.01	-0.06	-0.13	0.08	0.00	0.00
27	1	0.00	0.01	0.01	-0.30	0.14	0.27	-0.12	0.12	0.25
28	7	0.00	0.00	-0.01	0.16	0.06	0.14	-0.12	0.03	0.06
29	1	0.01	0.02	0.03	0.02	0.12	0.20	0.02	0.01	0.03

30 6 0.00 0.00 0.00 -0.17 -0.06 -0.12 0.20 -0.09 -0.17
31 1 0.00 0.00 0.00 0.06 -0.09 -0.17 -0.50 -0.04 -0.11
32 6 0.00 0.00 0.00 -0.08 0.03 0.06 -0.02 0.16 0.32
33 1 0.00 0.00 0.00 -0.07 -0.17 -0.35 0.05 -0.24 -0.49
34 6 0.00 0.00 0.00 0.13 0.03 0.08 -0.10 -0.09 -0.18
35 1 0.01 0.00 0.00 -0.63 -0.04 -0.11 0.21 -0.07 -0.13
36 1 0.00 0.00 0.00 0.00 0.00 0.00 0.00 0.00 0.00

76 77 78
A A A
Frequencies -- 1467.7639 1504.8904 1513.8943
Red. masses -- 1.5716 2.6187 3.8712
Frc consts -- 1.9948 3.4942 5.2274
IR Inten -- 25.4042 13.5268 22.4760
Raman Activ -- 1.5164 0.1133 12.6690
Depolar (P) -- 0.7350 0.5318 0.2611
Depolar (U) -- 0.8472 0.6943 0.4141

Atom	AN	X	Y	Z	X	Y	Z	X	Y	Z
1	8	0.00	0.02	0.01	0.00	0.00	0.00	0.00	-0.01	0.00
2	1	-0.01	-0.07	-0.05	0.00	-0.02	-0.02	0.00	0.02	0.02
3	1	-0.01	-0.05	-0.01	0.00	-0.02	0.00	0.00	0.02	0.00
4	6	-0.03	0.02	0.00	-0.13	-0.09	0.02	-0.10	0.22	-0.04
5	6	0.05	-0.03	0.01	0.01	0.10	-0.02	0.24	0.00	0.00
6	6	-0.06	-0.02	0.01	0.13	-0.04	0.00	-0.06	-0.10	0.02
7	6	-0.03	0.16	0.03	-0.01	0.08	0.01	0.01	-0.15	0.01
8	6	-0.05	0.00	0.00	0.12	-0.08	0.02	-0.10	-0.22	0.05
9	6	-0.01	0.00	-0.01	-0.01	-0.02	0.01	-0.07	-0.01	-0.03
10	6	0.03	-0.02	0.00	0.01	0.19	-0.04	0.08	0.00	0.00
11	1	0.24	0.13	-0.04	-0.29	-0.27	0.06	0.41	0.13	-0.04
12	8	0.05	-0.02	-0.02	0.03	-0.01	-0.01	0.00	0.02	0.00
13	8	-0.05	-0.05	0.00	-0.01	-0.02	0.00	0.00	0.03	0.00
14	6	0.02	0.02	0.00	-0.13	-0.03	0.00	-0.08	0.10	-0.02
15	6	0.00	0.00	0.00	0.01	0.04	0.00	0.02	0.11	-0.02
16	8	0.00	0.00	0.00	0.00	0.00	-0.01	0.00	0.00	0.00
17	8	0.00	0.00	0.00	0.00	0.00	0.00	0.00	0.02	0.04
18	1	0.05	0.15	-0.03	-0.02	-0.66	0.13	0.11	-0.04	0.01
19	1	0.68	-0.58	-0.18	0.18	-0.15	-0.05	-0.20	0.17	0.05
20	1	0.04	0.01	0.00	0.29	-0.31	0.07	0.48	-0.22	0.05
21	8	0.01	0.00	0.00	-0.02	0.00	-0.01	0.00	-0.01	0.00
22	8	0.00	0.00	0.00	0.00	-0.01	0.00	-0.01	-0.02	0.00
23	1	0.01	0.01	0.00	-0.04	-0.03	-0.01	-0.06	-0.05	-0.02
24	8	0.00	0.00	0.00	0.00	0.00	0.00	0.00	0.00	0.00
25	1	-0.01	0.00	-0.01	0.00	0.00	-0.01	-0.03	0.00	-0.01
26	7	0.00	0.00	0.00	0.00	0.00	0.00	-0.01	0.00	-0.01
27	1	0.00	0.00	0.01	0.00	0.00	0.00	-0.03	0.01	0.02
28	7	0.00	0.00	0.00	0.00	0.00	0.00	0.01	0.01	0.02
29	1	-0.01	-0.03	-0.04	0.00	0.01	-0.02	-0.06	-0.21	-0.36
30	6	0.00	0.00	0.00	0.00	0.00	0.00	-0.01	-0.01	-0.01
31	1	-0.01	0.00	0.00	0.00	0.00	0.00	-0.01	-0.01	-0.02
32	6	0.00	0.00	0.00	0.00	0.00	0.00	0.00	0.01	0.02
33	1	0.00	0.00	-0.01	0.00	0.00	0.00	0.00	-0.02	-0.03
34	6	0.00	0.00	0.00	0.00	0.00	0.00	0.01	0.00	0.00
35	1	0.00	0.00	0.00	0.00	0.00	0.00	-0.02	-0.01	-0.01
36	1	0.00	0.00	0.00	0.00	0.01	0.00	0.00	0.00	0.00

79 80 81
A A A
Frequencies -- 1539.4849 1561.7226 1591.6837
Red. masses -- 2.5891 1.5496 2.5276
Frc consts -- 3.6153 2.2268 3.7729
IR Inten -- 48.5029 10.6083 9.1632
Raman Activ -- 10.9951 2.4322 5.4600

Depolar (P) -- 0.5805 0.7431 0.6302
Depolar (U) -- 0.7346 0.8526 0.7732

Atom	AN	X	Y	Z	X	Y	Z	X	Y	Z
1	8	0.00	0.00	0.00	0.00	0.00	0.00	0.00	0.00	0.00
2	1	0.00	0.00	0.00	0.00	0.00	0.00	0.00	0.00	0.00
3	1	0.00	0.00	0.00	0.00	0.00	0.00	0.00	0.00	0.00
4	6	0.01	-0.01	0.00	0.00	0.02	0.00	0.00	0.01	0.00
5	6	-0.01	0.00	0.00	0.04	0.00	0.00	0.02	0.00	0.00
6	6	0.00	0.00	0.00	-0.02	-0.01	0.00	-0.01	-0.01	0.00
7	6	0.00	0.00	0.00	0.00	-0.01	0.00	0.00	-0.01	0.00
8	6	0.00	0.01	0.00	0.00	-0.02	0.00	0.00	-0.01	0.00
9	6	0.01	0.03	0.05	-0.06	-0.04	-0.09	-0.05	-0.01	-0.02
10	6	-0.01	0.00	0.00	0.01	0.00	0.00	0.01	0.00	0.00
11	1	-0.02	-0.01	0.00	0.04	0.02	-0.01	0.02	0.01	0.00
12	8	0.00	0.00	0.00	0.00	0.00	0.00	0.00	0.00	0.00
13	8	0.00	0.00	0.00	0.00	0.00	0.00	0.00	0.00	0.00
14	6	0.01	0.00	0.00	-0.02	0.01	0.00	-0.01	0.01	0.00
15	6	0.00	0.00	0.00	0.00	0.01	0.00	0.00	0.00	0.00
16	8	0.01	-0.02	-0.04	-0.02	0.03	0.06	0.00	0.00	0.01
17	8	-0.02	0.01	0.02	0.04	-0.02	-0.03	0.02	-0.01	-0.01
18	1	-0.01	0.00	0.00	0.02	0.00	0.00	0.01	0.00	0.00
19	1	0.00	0.00	0.00	0.00	0.00	0.00	0.00	0.00	0.00
20	1	-0.02	0.01	0.00	0.05	-0.03	0.01	0.03	-0.02	0.00
21	8	0.00	0.00	0.00	0.00	0.00	0.00	0.00	0.00	0.00
22	8	0.00	0.00	0.00	0.00	0.00	0.00	0.00	0.00	0.00
23	1	0.00	0.00	0.00	0.00	0.00	0.00	0.00	0.00	0.00
24	8	0.00	0.00	0.00	0.00	0.00	0.00	0.00	0.00	0.00
25	1	-0.01	0.01	-0.02	0.02	-0.03	0.04	0.00	-0.01	0.03
26	7	0.20	-0.05	-0.09	0.05	0.00	0.00	0.08	-0.04	-0.07
27	1	-0.31	0.30	0.60	0.06	0.00	0.00	-0.35	0.24	0.47
28	7	0.00	0.02	0.03	0.01	-0.01	-0.02	-0.15	-0.01	-0.02
29	1	-0.04	-0.22	-0.37	0.12	0.46	0.80	0.10	0.20	0.37
30	6	-0.11	0.01	0.01	-0.07	0.01	0.02	0.20	0.01	0.03
31	1	0.15	-0.01	-0.01	0.17	-0.01	-0.01	-0.35	0.06	0.11
32	6	0.10	-0.02	-0.04	0.06	0.01	0.01	-0.09	-0.05	-0.11
33	1	0.10	0.07	0.14	0.07	0.01	0.02	-0.15	0.09	0.18
34	6	-0.22	0.02	0.02	-0.10	-0.01	-0.03	0.08	0.06	0.12
35	1	0.22	0.07	0.15	0.17	0.01	0.03	-0.26	0.04	0.06
36	1	0.00	0.02	0.00	0.00	-0.04	-0.01	0.00	-0.03	0.00

82 83 84
A A A

Frequencies -- 1649.2659 1657.8124 1666.0277
Red. masses -- 1.0945 7.4008 6.3963
Frc consts -- 1.7540 11.9839 10.4603
IR Inten -- 31.2017 59.8975 9.5473
Raman Activ -- 1.9853 35.5496 19.2973
Depolar (P) -- 0.5098 0.7500 0.7497
Depolar (U) -- 0.6753 0.8571 0.8569

Atom	AN	X	Y	Z	X	Y	Z	X	Y	Z
1	8	0.01	-0.01	0.00	0.00	0.00	0.00	0.00	0.00	0.00
2	1	-0.08	0.03	0.05	-0.02	0.01	0.02	0.04	0.00	-0.02
3	1	-0.05	0.06	-0.05	-0.01	0.02	-0.01	0.02	-0.02	0.02
4	6	0.00	-0.01	0.00	-0.08	0.27	-0.05	-0.29	0.02	0.00
5	6	0.00	0.01	0.00	0.01	-0.35	0.07	0.18	0.02	-0.01
6	6	0.00	0.00	0.00	-0.09	-0.22	0.04	0.34	0.13	-0.03
7	6	0.02	0.00	-0.01	-0.02	-0.06	0.01	-0.03	0.00	0.02
8	6	0.01	0.00	0.00	0.04	0.24	-0.05	-0.30	-0.05	0.01
9	6	0.01	0.00	0.01	0.01	0.03	-0.03	-0.03	-0.02	-0.04
10	6	0.00	-0.01	0.00	0.00	0.40	-0.08	-0.18	-0.03	0.01
11	1	0.00	0.00	0.00	0.27	-0.07	0.01	-0.40	-0.25	0.06

12	8	-0.01	0.00	0.00	0.01	0.01	-0.01	0.04	0.01	-0.02
13	8	0.00	0.00	0.00	0.00	0.00	0.00	0.00	0.00	0.00
14	6	-0.01	0.01	0.00	0.13	-0.24	0.05	0.31	-0.11	0.02
15	6	0.00	0.00	0.00	0.00	-0.05	0.01	-0.02	0.02	-0.02
16	8	0.00	0.00	0.00	-0.01	0.00	0.01	-0.01	0.01	0.02
17	8	0.00	0.00	0.00	0.00	0.00	0.00	0.01	0.00	0.01
18	1	0.00	0.01	0.00	-0.04	-0.45	0.08	-0.21	0.05	-0.01
19	1	0.02	-0.02	-0.01	-0.04	0.03	0.01	-0.01	0.00	0.00
20	1	0.01	-0.01	0.00	-0.33	0.01	-0.01	-0.35	0.29	-0.07
21	8	0.00	0.00	0.00	0.00	0.01	0.00	0.04	-0.01	0.02
22	8	0.00	0.00	0.00	0.00	0.00	0.00	0.00	0.00	0.00
23	1	0.00	0.00	0.00	0.03	0.02	0.01	-0.01	0.00	0.00
24	8	0.02	0.06	-0.03	0.00	0.01	0.00	0.00	0.01	0.00
25	1	-0.27	-0.28	0.62	-0.03	-0.04	0.08	-0.03	-0.04	0.08
26	7	0.00	0.00	0.00	0.00	0.00	0.00	0.01	0.00	-0.01
27	1	0.00	0.00	0.00	-0.01	0.00	0.01	-0.02	0.02	0.04
28	7	0.00	0.00	0.00	0.00	0.00	0.00	-0.01	0.00	0.00
29	1	0.00	-0.05	-0.03	0.00	-0.03	0.02	0.00	0.02	0.03
30	6	0.00	0.00	0.00	0.00	0.00	0.00	0.01	0.00	0.00
31	1	0.00	0.00	0.00	0.00	0.00	0.00	-0.01	0.00	0.00
32	6	0.00	0.00	0.00	0.00	0.00	0.00	0.00	0.00	0.00
33	1	0.00	0.00	0.00	0.00	0.00	0.00	0.00	0.00	0.01
34	6	0.00	0.00	0.00	0.00	0.00	0.00	0.00	0.00	0.00
35	1	0.00	0.00	0.00	0.00	0.00	0.00	-0.01	0.00	0.00
36	1	-0.07	-0.64	-0.12	-0.01	-0.08	-0.01	-0.01	-0.08	-0.01

	85	86	87
	A	A	A
Frequencies --	1685.9291	1811.9039	1821.0979
Red. masses --	1.0765	6.9126	8.5160
Frc consts --	1.8028	13.3710	16.6399
IR Inten --	55.5552	217.7786	290.3235
Raman Activ --	1.2354	20.0688	34.4412
Depolar (P) --	0.6273	0.3735	0.2434
Depolar (U) --	0.7710	0.5438	0.3915

Atom	AN	X	Y	Z	X	Y	Z	X	Y	Z
1	8	-0.06	0.03	0.00	0.00	0.00	0.00	-0.01	0.01	0.00
2	1	0.62	-0.15	-0.41	0.01	-0.01	-0.03	0.08	-0.06	-0.09
3	1	0.33	-0.40	0.36	0.01	-0.02	0.01	0.01	-0.11	0.03
4	6	0.01	0.00	0.00	-0.03	0.01	0.00	-0.09	-0.01	0.01
5	6	0.00	0.00	0.00	0.07	-0.02	-0.01	0.01	-0.01	0.00
6	6	0.00	0.00	0.00	0.01	0.00	0.00	0.02	-0.02	0.00
7	6	-0.01	-0.01	0.00	0.12	0.00	-0.04	0.60	0.01	-0.19
8	6	0.00	0.00	0.00	-0.03	0.01	0.00	0.01	0.00	0.00
9	6	0.00	0.00	0.00	-0.30	0.24	0.42	0.07	-0.03	-0.08
10	6	0.00	0.00	0.00	0.00	0.00	0.00	0.00	0.02	-0.01
11	1	0.00	0.00	0.00	-0.01	-0.01	0.00	-0.01	-0.04	0.01
12	8	0.01	0.00	0.00	-0.07	0.00	0.02	-0.36	0.00	0.12
13	8	0.00	0.00	0.00	-0.01	0.01	0.00	-0.06	0.04	0.02
14	6	0.00	0.00	0.00	0.01	-0.01	0.00	0.01	-0.01	0.00
15	6	0.00	0.00	0.00	0.08	-0.01	0.03	-0.15	0.02	-0.06
16	8	0.00	0.00	0.00	0.17	-0.15	-0.26	-0.04	0.03	0.05
17	8	0.00	0.00	0.00	-0.01	-0.03	-0.06	0.00	0.00	0.01
18	1	0.00	0.00	0.00	-0.01	0.00	0.00	-0.01	-0.03	0.01
19	1	-0.04	0.03	-0.01	0.07	-0.08	-0.02	0.35	-0.37	-0.11
20	1	0.00	0.00	0.00	-0.03	0.02	0.00	-0.01	0.00	0.00
21	8	0.00	0.00	0.00	-0.05	0.00	-0.02	0.10	-0.01	0.04
22	8	0.00	0.00	0.00	-0.01	0.00	0.00	0.01	0.00	0.00
23	1	0.00	0.00	0.00	0.04	0.03	0.02	-0.08	-0.06	-0.03
24	8	0.00	0.01	0.00	0.00	0.00	0.00	0.00	-0.01	0.01
25	1	-0.04	-0.03	0.07	0.03	0.03	-0.02	0.03	0.07	-0.15

26	7	0.00	0.00	0.00	-0.01	0.01	0.02	0.00	0.00	0.00
27	1	0.00	0.00	0.00	0.12	-0.11	-0.22	-0.02	0.04	0.04
28	7	0.00	0.00	0.00	0.02	0.00	-0.01	0.00	0.00	0.00
29	1	0.00	0.00	0.00	0.21	0.28	0.54	-0.04	-0.03	-0.14
30	6	0.00	0.00	0.00	-0.02	0.00	0.00	0.00	0.00	0.00
31	1	0.00	0.00	0.00	0.02	0.00	-0.01	0.00	0.00	0.00
32	6	0.00	0.00	0.00	0.00	0.00	0.00	0.00	0.00	0.00
33	1	0.00	0.00	0.00	0.00	-0.01	-0.02	0.00	0.00	0.00
34	6	0.00	0.00	0.00	0.00	-0.01	-0.01	0.00	0.00	0.00
35	1	0.00	0.00	0.00	0.03	-0.01	-0.01	0.00	0.00	0.00
36	1	0.00	-0.11	-0.01	0.01	0.02	0.00	0.02	0.09	0.02

	88	89	90
	A	A	A
Frequencies --	1850.5154	2828.1929	3213.2203
Red. masses --	10.1130	1.0811	1.0887
Frc consts --	20.4040	5.0951	6.6225
IR Inten --	501.9719	3286.0260	5.8060
Raman Activ --	73.2193	403.9878	75.0531
Depolar (P) --	0.1158	0.3263	0.5704
Depolar (U) --	0.2076	0.4921	0.7264

Atom	AN	X	Y	Z	X	Y	Z	X	Y	Z
1	8	0.00	0.00	0.00	0.00	0.00	0.00	0.00	0.00	0.00
2	1	0.02	-0.01	-0.02	0.00	0.00	0.00	0.00	0.00	0.00
3	1	0.00	-0.02	0.01	0.00	0.00	0.00	0.00	0.00	0.00
4	6	-0.03	-0.01	0.01	0.00	0.00	0.00	0.00	0.00	0.00
5	6	0.02	0.01	0.00	0.00	0.00	0.00	0.00	0.00	0.00
6	6	0.02	0.01	0.00	0.00	0.00	0.00	0.02	-0.03	0.00
7	6	0.15	0.01	-0.05	0.00	0.00	0.00	0.00	0.00	0.00
8	6	-0.09	0.03	-0.02	0.00	0.00	0.00	0.00	0.00	0.00
9	6	0.08	-0.06	-0.09	0.00	-0.01	-0.02	0.00	0.00	0.00
10	6	-0.01	-0.02	0.00	0.00	0.00	0.00	-0.08	0.00	0.00
11	1	-0.01	-0.01	0.00	0.00	0.00	0.00	-0.18	0.33	-0.06
12	8	-0.09	0.00	0.03	0.00	0.00	0.00	0.00	0.00	0.00
13	8	-0.02	0.01	0.00	0.00	0.00	0.00	0.00	0.00	0.00
14	6	0.02	0.01	0.00	0.00	0.00	0.00	0.01	0.02	0.00
15	6	0.65	-0.10	0.24	0.00	0.00	0.00	0.00	0.00	0.00
16	8	-0.04	0.04	0.06	0.00	0.00	0.01	0.00	0.00	0.00
17	8	-0.01	0.01	0.01	-0.07	0.01	0.01	0.00	0.00	0.00
18	1	-0.01	0.02	0.00	0.00	0.00	0.00	0.87	-0.03	0.00
19	1	0.08	-0.10	-0.02	-0.01	-0.01	0.00	-0.01	-0.01	0.00
20	1	-0.01	0.04	-0.01	0.00	0.00	0.00	-0.15	-0.24	0.05
21	8	-0.41	0.04	-0.15	0.00	0.00	0.00	0.00	0.00	0.00
22	8	-0.06	-0.01	-0.02	0.00	0.00	0.00	0.00	0.00	0.00
23	1	0.33	0.28	0.09	0.00	0.00	0.00	0.00	0.00	0.00
24	8	0.00	0.00	0.00	0.00	0.00	0.00	0.00	0.00	0.00
25	1	0.00	0.01	-0.02	0.00	-0.02	-0.01	0.00	0.00	0.00
26	7	0.00	0.00	0.00	0.00	0.00	0.00	0.00	0.00	0.00
27	1	-0.03	0.02	0.06	-0.02	-0.02	-0.03	0.00	0.00	0.00
28	7	0.00	0.00	0.00	0.00	0.00	0.00	0.00	0.00	0.00
29	1	-0.06	-0.09	-0.14	0.99	-0.11	-0.06	0.00	0.00	0.00
30	6	0.00	0.00	0.00	0.00	0.00	0.00	0.00	0.00	0.00
31	1	0.00	0.00	0.00	0.00	-0.01	-0.02	0.00	0.00	0.00
32	6	0.00	0.00	0.00	0.00	0.00	0.00	0.00	0.00	0.00
33	1	0.00	0.00	0.00	0.00	0.00	0.00	0.00	0.00	0.00
34	6	0.00	0.00	0.00	0.00	0.00	0.00	0.00	0.00	0.00
35	1	-0.01	0.00	0.00	0.01	0.00	0.00	0.00	0.00	0.00
36	1	0.01	0.01	0.00	0.00	0.00	0.00	0.00	0.00	0.00

	91	92	93
	A	A	A
Frequencies --	3232.4913	3240.0430	3264.9276

Red. masses --	1.0933	1.0962	1.0946
Frc consts --	6.7306	6.7802	6.8744
IR Inten --	0.3201	2.8452	1.6375
Raman Activ --	83.5130	181.3829	71.7821
Depolar (P) --	0.4294	0.1322	0.5193
Depolar (U) --	0.6008	0.2335	0.6836

Atom	AN	X	Y	Z	X	Y	Z	X	Y	Z
1	8	0.00	0.00	0.00	0.00	0.00	0.00	0.00	0.00	0.00
2	1	0.00	0.00	0.00	0.00	0.00	0.00	0.00	0.00	0.00
3	1	0.00	0.00	0.00	0.00	0.00	0.00	0.00	0.00	0.00
4	6	0.00	0.00	0.00	0.00	0.00	0.00	0.00	0.00	0.00
5	6	0.00	0.00	0.00	0.00	0.00	0.00	0.00	0.00	0.00
6	6	0.03	-0.07	0.01	-0.01	0.02	0.00	0.00	0.00	0.00
7	6	0.00	0.00	0.00	0.00	0.00	0.00	0.00	0.00	0.00
8	6	0.00	0.00	0.00	0.00	0.00	0.00	0.00	0.00	0.00
9	6	0.00	0.00	0.00	0.00	0.00	0.00	0.00	0.00	0.00
10	6	0.02	0.00	0.00	-0.04	0.00	0.00	0.00	0.00	0.00
11	1	-0.40	0.76	-0.14	0.14	-0.26	0.05	0.00	0.00	0.00
12	8	0.00	0.00	0.00	0.00	0.00	0.00	0.00	0.00	0.00
13	8	0.00	0.00	0.00	0.00	0.00	0.00	0.00	0.00	0.00
14	6	-0.02	-0.03	0.01	-0.04	-0.06	0.01	0.00	0.00	0.00
15	6	0.00	0.00	0.00	0.00	0.00	0.00	0.00	0.00	0.00
16	8	0.00	0.00	0.00	0.00	0.00	0.00	0.00	0.00	0.00
17	8	0.00	0.00	0.00	0.00	0.00	0.00	0.00	0.00	0.00
18	1	-0.24	0.00	0.00	0.41	-0.02	0.00	0.00	0.00	0.00
19	1	-0.01	-0.01	0.00	0.00	0.00	0.00	0.01	0.01	0.00
20	1	0.22	0.35	-0.07	0.44	0.72	-0.15	0.00	0.00	0.00
21	8	0.00	0.00	0.00	0.00	0.00	0.00	0.00	0.00	0.00
22	8	0.00	0.00	0.00	0.00	0.00	0.00	0.00	0.00	0.00
23	1	0.00	0.00	0.00	0.00	0.00	0.00	0.00	0.00	0.00
24	8	0.00	0.00	0.00	0.00	0.00	0.00	0.00	0.00	0.00
25	1	0.00	0.00	0.00	0.00	0.00	0.00	0.00	0.00	0.00
26	7	0.00	0.00	0.00	0.00	0.00	0.00	0.00	0.00	0.00
27	1	0.00	0.00	0.00	0.00	0.00	0.00	0.00	0.00	0.00
28	7	0.00	0.00	0.00	0.00	0.00	0.00	0.00	0.00	0.00
29	1	0.00	0.00	0.00	0.00	0.00	0.00	0.01	0.00	0.00
30	6	0.00	0.00	0.00	0.00	0.00	0.00	-0.01	-0.04	-0.07
31	1	0.00	0.00	0.00	0.00	0.00	0.00	0.14	0.40	0.82
32	6	0.00	0.00	0.00	0.00	0.00	0.00	0.03	0.00	0.01
33	1	0.00	0.00	0.00	0.00	0.00	0.00	-0.35	-0.01	-0.03
34	6	0.00	0.00	0.00	0.00	0.00	0.00	0.00	0.00	0.01
35	1	0.00	0.00	0.00	0.00	0.00	0.00	0.03	-0.04	-0.09
36	1	0.00	0.00	0.00	0.00	0.00	0.00	0.00	0.00	0.00

	94	95	96
	A	A	A
Frequencies --	3272.2224	3282.1951	3298.9159
Red. masses --	1.0687	1.0953	1.1082
Frc consts --	6.7421	6.9517	7.1057
IR Inten --	1240.4045	0.4849	0.0049
Raman Activ --	174.9914	59.8755	169.5436
Depolar (P) --	0.1990	0.6439	0.1234
Depolar (U) --	0.3320	0.7834	0.2197

Atom	AN	X	Y	Z	X	Y	Z	X	Y	Z
1	8	0.00	0.01	0.00	0.00	0.00	0.00	0.00	0.00	0.00
2	1	0.04	-0.07	0.08	0.00	0.00	0.00	0.00	0.00	0.00
3	1	0.02	0.00	-0.02	0.00	0.00	0.00	0.00	0.00	0.00
4	6	0.00	0.00	0.00	0.00	0.00	0.00	0.00	0.00	0.00
5	6	0.00	0.00	0.00	0.00	0.00	0.00	0.00	0.00	0.00
6	6	0.00	0.00	0.00	0.00	0.00	0.00	0.00	0.00	0.00
7	6	0.01	-0.01	0.00	0.00	0.00	0.00	0.00	0.00	0.00

8	6	0.00	0.00	0.00	0.00	0.00	0.00	0.00	0.00	0.00
9	6	0.00	0.00	0.00	0.00	0.00	0.00	0.00	0.00	0.00
10	6	0.00	0.00	0.00	0.00	0.00	0.00	0.00	0.00	0.00
11	1	-0.01	0.02	0.00	0.00	0.00	0.00	0.00	0.00	0.00
12	8	0.00	0.00	0.00	0.00	0.00	0.00	0.00	0.00	0.00
13	8	-0.04	-0.05	0.00	0.00	0.00	0.00	0.00	0.00	0.00
14	6	0.00	0.00	0.00	0.00	0.00	0.00	0.00	0.00	0.00
15	6	0.00	0.00	0.00	0.00	0.00	0.00	0.00	0.00	0.00
16	8	0.00	0.00	0.00	0.00	0.00	0.00	0.00	0.00	0.00
17	8	0.00	0.00	0.00	0.00	0.00	0.00	0.00	0.00	0.00
18	1	0.01	0.00	0.00	0.00	0.00	0.00	0.00	0.00	0.00
19	1	0.60	0.79	0.00	0.00	0.00	0.00	0.00	0.00	0.00
20	1	0.00	0.00	0.00	0.00	0.00	0.00	0.00	0.00	0.00
21	8	0.00	0.00	0.00	0.00	0.00	0.00	0.00	0.00	0.00
22	8	0.00	0.00	0.00	0.00	0.00	0.00	0.00	0.00	0.00
23	1	0.00	0.00	0.00	0.00	0.00	0.00	0.00	0.00	0.00
24	8	0.00	0.00	0.00	0.00	0.00	0.00	0.00	0.00	0.00
25	1	0.00	-0.02	0.00	0.00	0.00	0.00	0.00	0.00	0.00
26	7	0.00	0.00	0.00	0.00	0.00	0.00	0.00	0.00	0.00
27	1	-0.01	0.00	-0.01	0.00	0.00	-0.01	0.01	0.01	0.01
28	7	0.00	0.00	0.00	0.00	0.00	0.00	0.00	0.00	0.00
29	1	0.01	0.00	0.00	0.01	0.00	0.00	0.00	0.00	0.00
30	6	0.00	0.00	0.00	0.00	-0.01	-0.03	0.00	-0.01	-0.02
31	1	0.00	0.00	-0.01	0.04	0.14	0.28	0.02	0.08	0.16
32	6	0.00	0.00	0.00	-0.06	0.00	0.00	-0.07	0.00	-0.01
33	1	0.01	0.00	0.00	0.63	0.01	0.04	0.68	0.01	0.05
34	6	0.00	0.00	0.00	0.02	-0.03	-0.05	-0.01	0.03	0.06
35	1	0.00	0.00	0.00	-0.19	0.30	0.61	0.18	-0.30	-0.60
36	1	0.00	0.00	0.00	0.00	0.00	0.00	0.00	0.00	0.00

	97	98	99
	A	A	A
Frequencies --	3506.7597	3631.1685	3767.1567
Red. masses --	1.0863	1.0604	1.0558
Frc consts --	7.8703	8.2382	8.8277
IR Inten --	800.4696	570.5238	347.8132
Raman Activ --	157.1147	65.9507	110.8040
Depolar (P) --	0.2477	0.1927	0.2469
Depolar (U) --	0.3971	0.3232	0.3960

Atom	AN	X	Y	Z	X	Y	Z	X	Y	Z
1	8	0.00	0.00	0.00	-0.02	0.04	-0.04	0.00	0.00	0.00
2	1	0.00	0.00	0.00	0.28	-0.61	0.71	0.03	-0.08	0.09
3	1	0.00	0.00	0.00	0.07	0.01	-0.10	0.02	0.00	-0.03
4	6	0.00	0.00	0.00	0.00	0.00	0.00	0.00	0.00	0.00
5	6	0.00	0.00	0.00	0.00	0.00	0.00	0.00	0.00	0.00
6	6	0.00	0.00	0.00	0.00	0.00	0.00	0.00	0.00	0.00
7	6	0.00	0.00	0.00	0.00	0.00	0.00	0.00	0.00	0.00
8	6	0.00	0.00	0.00	0.00	0.00	0.00	0.00	0.00	0.00
9	6	0.00	0.00	0.00	0.00	0.00	0.00	0.00	0.00	0.00
10	6	0.00	0.00	0.00	0.00	0.00	0.00	0.00	0.00	0.00
11	1	0.00	0.00	0.00	0.00	0.00	0.00	0.00	0.00	0.00
12	8	0.00	0.00	0.00	0.00	0.00	0.00	0.00	0.00	0.00
13	8	0.00	0.00	0.00	0.00	0.01	0.00	0.00	0.00	0.00
14	6	0.00	0.00	0.00	0.00	0.00	0.00	0.00	0.00	0.00
15	6	0.00	0.00	0.00	0.00	0.00	0.00	0.00	0.00	0.00
16	8	0.00	0.00	0.00	0.00	0.00	0.00	0.00	0.00	0.00
17	8	0.00	0.00	0.00	0.00	0.00	0.00	0.00	0.00	0.00
18	1	0.00	0.00	0.00	0.00	0.00	0.00	0.00	0.00	0.00
19	1	0.01	0.01	0.00	-0.08	-0.09	0.00	0.00	0.00	0.00
20	1	0.00	0.00	0.00	0.00	0.00	0.00	0.00	0.00	0.00
21	8	0.00	0.00	0.00	0.00	0.00	0.00	0.00	0.00	0.00

22	8	0.00	0.00	0.00	0.00	0.00	0.00	0.00	0.00	0.00
23	1	0.00	0.00	0.00	0.00	0.00	0.00	0.00	0.00	0.00
24	8	0.00	0.00	0.00	0.00	0.01	0.00	-0.01	-0.05	-0.01
25	1	0.00	-0.01	-0.01	0.00	-0.12	-0.05	0.00	0.86	0.44
26	7	-0.06	-0.02	-0.04	0.00	0.00	0.00	0.00	0.00	0.00
27	1	0.82	0.24	0.52	-0.01	0.00	0.00	0.01	0.00	0.01
28	7	0.00	0.00	0.00	0.00	0.00	0.00	0.00	0.00	0.00
29	1	0.04	0.00	0.00	-0.01	0.00	0.00	0.03	0.00	0.00
30	6	0.00	0.00	0.00	0.00	0.00	0.00	0.00	0.00	0.00
31	1	0.00	0.00	0.00	0.00	0.00	0.00	0.00	0.00	0.00
32	6	0.00	0.00	0.00	0.00	0.00	0.00	0.00	0.00	0.00
33	1	0.00	0.00	0.00	0.00	0.00	0.00	0.00	0.00	0.00
34	6	0.00	0.00	0.00	0.00	0.00	0.00	0.00	0.00	0.00
35	1	-0.01	0.01	0.01	0.00	0.00	0.00	0.00	0.00	0.00
36	1	0.00	0.00	0.00	-0.01	-0.01	0.03	0.08	0.00	-0.22

	100	101	102
	A	A	A
Frequencies --	3850.7006	3940.8628	3971.8542
Red. masses --	1.0647	1.0677	1.0721
Frc consts --	9.3017	9.7695	9.9649
IR Inten --	115.6853	73.7155	117.9754
Raman Activ --	127.6820	34.2805	38.6133
Depolar (P) --	0.2588	0.1971	0.3205
Depolar (U) --	0.4111	0.3293	0.4854

Atom	AN	X	Y	Z	X	Y	Z	X	Y	Z
1	8	0.00	0.00	0.00	0.04	0.00	-0.05	0.00	0.00	0.00
2	1	0.00	0.00	0.00	0.06	-0.08	0.07	0.00	0.00	0.00
3	1	0.00	0.00	0.00	-0.68	0.04	0.72	0.00	0.00	0.00
4	6	0.00	0.00	0.00	0.00	0.00	0.00	0.00	0.00	0.00
5	6	0.00	0.00	0.00	0.00	0.00	0.00	0.00	0.00	0.00
6	6	0.00	0.00	0.00	0.00	0.00	0.00	0.00	0.00	0.00
7	6	0.00	0.00	0.00	0.00	0.00	0.00	0.00	0.00	0.00
8	6	0.00	0.00	0.00	0.00	0.00	0.00	0.00	0.00	0.00
9	6	0.00	0.00	0.00	0.00	0.00	0.00	0.00	0.00	0.00
10	6	0.00	0.00	0.00	0.00	0.00	0.00	0.00	0.00	0.00
11	1	0.00	0.00	0.00	0.00	0.00	0.00	0.00	0.00	0.00
12	8	0.00	0.00	0.00	0.00	0.00	0.00	0.00	0.00	0.00
13	8	0.00	0.00	0.00	0.00	0.00	0.00	0.00	0.00	0.00
14	6	0.00	0.00	0.00	0.00	0.00	0.00	0.00	0.00	0.00
15	6	0.00	0.00	0.00	0.00	0.00	0.00	0.00	0.00	0.00
16	8	0.00	0.00	0.00	0.00	0.00	0.00	0.00	0.00	0.00
17	8	0.00	0.00	0.00	0.00	0.00	0.00	0.00	0.00	0.00
18	1	0.00	0.00	0.00	0.00	0.00	0.00	0.00	0.00	0.00
19	1	0.00	0.00	0.00	0.01	0.01	0.00	0.00	0.00	0.00
20	1	0.00	0.00	0.00	0.00	0.00	0.00	0.00	0.00	0.00
21	8	0.00	0.00	0.00	0.00	0.00	0.00	0.00	0.00	0.00
22	8	0.03	-0.05	0.02	0.00	0.00	0.00	0.00	0.00	0.00
23	1	-0.51	0.81	-0.28	0.00	0.00	0.00	0.00	0.00	0.00
24	8	0.00	0.00	0.00	0.00	0.00	0.00	0.02	-0.01	-0.06
25	1	0.00	0.00	0.00	0.00	0.02	0.01	0.01	0.20	0.07
26	7	0.00	0.00	0.00	0.00	0.00	0.00	0.00	0.00	0.00
27	1	0.00	0.00	0.00	0.00	0.00	0.00	0.00	0.00	0.00
28	7	0.00	0.00	0.00	0.00	0.00	0.00	0.00	0.00	0.00
29	1	0.00	0.00	0.00	0.00	0.00	0.00	0.01	0.00	0.00
30	6	0.00	0.00	0.00	0.00	0.00	0.00	0.00	0.00	0.00
31	1	0.00	0.00	0.00	0.00	0.00	0.00	0.00	0.00	0.00
32	6	0.00	0.00	0.00	0.00	0.00	0.00	0.00	0.00	0.00
33	1	0.00	0.00	0.00	0.00	0.00	0.00	0.00	0.00	0.00
34	6	0.00	0.00	0.00	0.00	0.00	0.00	0.00	0.00	0.00
35	1	0.00	0.00	0.00	0.00	0.00	0.00	0.00	0.00	0.00

36	1	0.00	0.00	0.00	0.00	0.00	0.00	-0.36	-0.10	0.90

PYRHEM

Harmonic frequencies (cm**-1), IR intensities (KM/Mole), Raman scattering activities (A**4/AMU), depolarization ratios for plane and unpolarized incident light, reduced masses (AMU), force constants (mDyne/A), and normal coordinates:

	1	2	3
	A	A	A
Frequencies --	13.6471	19.0481	46.4070
Red. masses --	8.4579	7.2008	7.7827
Frc consts --	0.0009	0.0015	0.0099
IR Inten --	0.7682	1.2719	1.3887
Raman Activ --	1.5196	2.1890	0.0249
Depolar (P) --	0.7486	0.7498	0.6851
Depolar (U) --	0.8562	0.8570	0.8131

Atom	AN	X	Y	Z	X	Y	Z	X	Y	Z
1	6	0.05	0.02	0.02	0.04	0.02	-0.01	0.00	0.01	0.06
2	6	0.00	0.05	0.00	0.00	0.04	0.00	0.00	0.01	0.08
3	6	-0.05	0.02	-0.01	-0.05	0.02	0.02	0.00	0.01	0.07
4	6	-0.05	-0.04	0.00	-0.05	-0.04	0.03	0.00	0.00	0.13
5	1	-0.09	-0.06	-0.01	-0.09	-0.05	0.05	0.00	0.00	0.14
6	6	0.00	-0.06	0.03	0.00	-0.06	0.02	0.00	0.01	0.19
7	6	0.04	-0.04	0.04	0.04	-0.04	-0.01	0.00	0.00	0.13
8	1	0.08	-0.06	0.06	0.08	-0.06	-0.02	0.00	0.00	0.12
9	6	0.10	0.04	-0.02	0.10	0.04	-0.01	0.00	0.00	-0.05
10	8	0.14	0.09	-0.22	0.07	0.08	0.15	-0.04	-0.03	0.15
11	6	0.00	0.10	-0.01	0.00	0.09	-0.01	0.00	-0.01	0.06
12	8	0.00	-0.03	0.00	0.00	0.21	-0.03	0.01	-0.01	0.05
13	8	0.00	0.25	-0.04	0.00	0.00	0.00	-0.02	-0.04	0.06
14	6	-0.10	0.05	0.00	-0.10	0.05	0.00	0.00	0.00	-0.04
15	8	-0.07	0.09	0.23	-0.13	0.09	-0.18	0.03	-0.02	0.12
16	8	-0.18	0.03	-0.26	-0.11	0.01	0.20	-0.03	0.03	-0.38
17	1	-0.21	0.05	-0.22	-0.14	0.03	0.16	-0.02	0.02	-0.43
18	7	0.01	-0.08	0.01	0.00	0.06	-0.01	0.01	-0.01	0.01
19	1	0.00	-0.03	0.00	0.00	0.19	-0.03	0.01	-0.02	0.04
20	7	0.00	0.02	-0.01	0.00	-0.12	0.01	-0.01	-0.02	0.01
21	1	0.00	0.14	-0.02	0.00	-0.11	0.01	-0.02	-0.04	0.03
22	6	0.00	-0.07	0.00	0.00	-0.30	0.03	-0.02	0.00	-0.01
23	1	0.00	-0.02	-0.01	0.00	-0.46	0.05	-0.04	0.00	-0.02
24	6	0.01	-0.25	0.02	0.00	-0.23	0.02	0.00	0.03	-0.03
25	1	0.01	-0.36	0.04	0.00	-0.34	0.03	0.00	0.06	-0.05
26	6	0.01	-0.24	0.02	0.00	-0.01	-0.01	0.02	0.02	-0.01
27	1	0.01	-0.35	0.04	0.00	0.10	-0.02	0.04	0.04	-0.02
28	8	0.09	0.00	0.20	0.17	0.02	-0.19	0.05	0.04	-0.42
29	1	-0.01	-0.11	0.05	-0.01	-0.11	0.02	0.00	0.01	0.26
30	1	0.13	0.02	0.14	0.19	0.04	-0.16	0.04	0.03	-0.46

	4	5	6
	A	A	A
Frequencies --	48.8375	58.4350	88.6761
Red. masses --	6.8591	4.6969	5.8088
Frc consts --	0.0096	0.0094	0.0269
IR Inten --	3.1654	0.7498	0.3043
Raman Activ --	4.0897	3.4216	2.1488
Depolar (P) --	0.7495	0.7498	0.7498
Depolar (U) --	0.8568	0.8570	0.8570

Atom	AN	X	Y	Z	X	Y	Z	X	Y	Z
1	6	-0.01	0.00	0.01	-0.01	-0.01	-0.02	-0.02	-0.03	0.01
2	6	0.00	-0.01	-0.07	0.00	-0.01	0.00	0.00	-0.05	0.02
3	6	0.01	0.00	0.01	0.01	0.00	0.02	0.02	-0.03	0.01

4	6	0.01	0.01	0.21	0.01	0.00	0.01	0.02	-0.01	0.00
5	1	0.01	0.01	0.28	0.01	0.01	0.02	0.04	0.00	-0.01
6	6	0.00	0.01	0.32	0.00	0.01	-0.01	0.00	0.00	0.00
7	6	-0.01	0.01	0.21	-0.01	0.00	-0.02	-0.02	-0.01	0.00
8	1	-0.01	0.01	0.28	-0.01	0.01	-0.03	-0.04	0.00	-0.01
9	6	-0.01	-0.01	-0.10	-0.02	-0.01	-0.05	-0.05	-0.04	0.00
10	8	0.02	-0.03	-0.27	0.00	-0.02	-0.16	-0.05	-0.06	0.02
11	6	0.00	-0.01	-0.11	0.00	-0.01	0.00	0.00	-0.03	0.02
12	8	-0.02	0.00	-0.10	0.00	-0.03	0.01	0.00	0.00	0.01
13	8	0.02	0.00	-0.10	0.00	0.02	0.00	0.00	0.01	0.01
14	6	0.01	-0.01	-0.10	0.01	-0.01	0.06	0.04	-0.05	0.00
15	8	-0.02	-0.02	-0.27	0.03	-0.01	0.17	0.05	-0.07	0.01
16	8	0.04	0.00	0.00	0.00	0.00	-0.04	0.06	-0.03	-0.02
17	1	0.04	-0.01	-0.09	0.01	0.00	-0.01	0.08	-0.04	-0.03
18	7	-0.03	0.02	0.04	0.00	0.18	-0.02	0.01	0.36	-0.04
19	1	-0.02	0.01	-0.07	0.00	0.07	0.00	0.00	0.14	-0.01
20	7	0.04	0.01	0.05	0.00	-0.17	0.03	0.00	0.36	-0.04
21	1	0.07	0.00	0.00	0.00	-0.25	0.04	-0.01	0.42	-0.04
22	6	0.06	0.01	0.12	0.00	-0.26	0.03	0.00	-0.04	0.00
23	1	0.12	0.00	0.14	0.00	-0.52	0.06	-0.01	-0.12	0.01
24	6	0.00	0.02	0.17	0.00	0.04	-0.01	0.01	-0.28	0.03
25	1	-0.01	0.02	0.23	0.00	0.07	-0.02	0.02	-0.61	0.06
26	6	-0.06	0.02	0.11	0.00	0.31	-0.04	0.02	-0.01	0.00
27	1	-0.12	0.03	0.12	0.01	0.58	-0.08	0.02	-0.08	0.01
28	8	-0.05	0.00	0.01	-0.05	-0.01	0.05	-0.06	-0.03	-0.02
29	1	0.00	0.02	0.48	0.00	0.01	-0.01	0.00	0.02	-0.01
30	1	-0.05	-0.01	-0.08	-0.05	-0.01	0.01	-0.07	-0.04	-0.02

	7	8	9
	A	A	A
Frequencies --	108.8565	118.6445	148.9925
Red. masses --	6.2913	8.0943	6.1289
Frc consts --	0.0439	0.0671	0.0802
IR Inten --	7.3972	3.9431	9.2642
Raman Activ --	0.1580	0.0805	0.2300
Depolar (P) --	0.5919	0.6159	0.3885
Depolar (U) --	0.7436	0.7623	0.5596

Atom	AN	X	Y	Z	X	Y	Z	X	Y	Z
1	6	-0.03	0.00	-0.03	0.06	-0.01	-0.02	0.02	0.00	0.09
2	6	-0.02	0.00	-0.09	0.03	0.00	-0.07	0.02	0.00	0.15
3	6	-0.02	0.00	-0.03	0.06	0.01	-0.04	0.01	0.00	0.08
4	6	-0.02	0.00	0.03	0.06	0.01	0.01	0.01	0.00	-0.03
5	1	-0.02	0.00	0.08	0.06	0.01	0.04	0.01	0.00	-0.10
6	6	-0.03	0.00	0.04	0.07	0.00	0.04	0.01	0.00	-0.07
7	6	-0.03	0.01	0.02	0.06	-0.01	0.03	0.02	0.00	-0.01
8	1	-0.03	0.01	0.06	0.05	0.00	0.07	0.02	0.00	-0.07
9	6	-0.05	0.00	0.07	0.12	0.02	0.03	0.01	-0.01	-0.01
10	8	-0.08	-0.01	0.22	0.12	0.09	0.11	0.03	-0.04	-0.12
11	6	-0.01	-0.01	-0.16	0.01	0.00	-0.09	0.01	0.01	0.18
12	8	0.02	-0.03	-0.17	-0.01	-0.04	-0.09	0.03	0.03	0.17
13	8	-0.03	-0.01	-0.17	-0.02	0.02	-0.11	0.01	-0.01	0.19
14	6	-0.04	0.01	0.06	0.12	-0.02	0.03	0.00	0.01	-0.01
15	8	-0.02	0.04	0.20	0.14	-0.07	0.11	-0.01	0.02	-0.10
16	8	-0.08	-0.01	0.02	0.17	0.01	0.01	0.00	0.01	-0.02
17	1	-0.08	0.00	0.11	0.21	-0.02	0.08	-0.02	0.01	-0.11
18	7	0.01	-0.01	-0.15	-0.28	0.01	-0.06	-0.13	-0.03	-0.25
19	1	0.02	-0.03	-0.21	0.01	-0.02	-0.08	0.03	0.01	0.02
20	7	0.19	-0.01	-0.13	-0.21	0.00	-0.05	0.08	-0.02	-0.22
21	1	0.28	-0.02	-0.25	-0.20	0.00	-0.08	0.16	-0.04	-0.33
22	6	0.26	0.00	0.05	-0.19	-0.01	0.03	0.15	0.00	-0.01
23	1	0.42	0.00	0.10	-0.13	-0.02	0.05	0.34	0.01	0.04

24	6	0.09	0.02	0.17	-0.26	0.00	0.08	-0.05	0.01	0.13
25	1	0.08	0.03	0.32	-0.26	0.00	0.14	-0.06	0.03	0.31
26	6	-0.07	0.01	0.03	-0.31	0.01	0.02	-0.22	-0.01	-0.04
27	1	-0.22	0.02	0.05	-0.38	0.02	0.03	-0.41	-0.01	-0.01
28	8	-0.04	0.00	0.01	0.19	-0.02	0.01	-0.02	0.01	-0.02
29	1	-0.03	0.01	0.09	0.07	0.00	0.08	0.01	0.00	-0.17
30	1	-0.07	0.00	0.12	0.23	0.01	0.06	-0.01	0.01	-0.12

	10	11	12
	A	A	A
Frequencies --	162.8458	176.5209	181.3001
Red. masses --	9.7533	6.7932	5.7367
Frc consts --	0.1524	0.1247	0.1111
IR Inten --	0.5791	0.2875	0.2772
Raman Activ --	1.0806	0.7277	0.4638
Depolar (P) --	0.7433	0.7499	0.7486
Depolar (U) --	0.8527	0.8571	0.8562

Atom	AN	X	Y	Z	X	Y	Z	X	Y	Z
1	6	0.04	-0.01	-0.15	-0.10	0.00	-0.12	0.06	-0.01	-0.16
2	6	0.04	0.00	-0.03	-0.10	0.00	0.02	0.07	0.00	-0.01
3	6	0.04	0.00	0.11	-0.10	0.00	0.14	0.07	0.00	0.15
4	6	0.04	0.00	0.17	-0.10	0.01	0.21	0.07	0.00	0.26
5	1	0.04	0.00	0.32	-0.09	0.02	0.37	0.07	0.00	0.47
6	6	0.04	0.00	0.02	-0.10	0.00	-0.01	0.07	0.00	0.00
7	6	0.04	0.00	-0.17	-0.10	-0.01	-0.21	0.06	0.00	-0.26
8	1	0.04	0.00	-0.28	-0.10	-0.01	-0.39	0.06	0.00	-0.46
9	6	0.00	-0.03	-0.08	0.01	0.07	-0.03	-0.01	-0.04	-0.01
10	8	0.00	-0.06	-0.11	0.03	0.24	0.01	-0.05	-0.10	0.14
11	6	0.03	-0.01	-0.02	-0.09	0.02	0.02	0.06	0.00	0.00
12	8	0.03	0.46	-0.08	-0.09	0.01	0.03	0.05	-0.16	0.02
13	8	0.03	-0.44	0.04	-0.09	0.04	0.02	0.07	0.14	-0.02
14	6	0.00	0.02	0.08	0.01	-0.08	0.03	-0.01	0.05	0.00
15	8	0.01	0.06	0.16	0.01	-0.26	-0.05	-0.04	0.13	-0.14
16	8	-0.05	0.01	-0.10	0.17	0.04	-0.07	-0.09	0.01	-0.06
17	1	-0.08	0.03	-0.12	0.27	-0.05	-0.18	-0.16	0.05	-0.21
18	7	-0.03	-0.03	0.01	0.06	-0.01	-0.02	-0.04	0.01	0.00
19	1	0.04	0.31	-0.05	-0.11	0.01	0.00	0.06	-0.11	0.02
20	7	-0.04	0.05	0.00	0.09	-0.01	-0.02	-0.05	-0.01	0.01
21	1	-0.05	-0.03	0.02	0.10	-0.01	-0.04	-0.06	0.02	0.01
22	6	-0.05	0.06	-0.01	0.10	0.00	0.00	-0.05	-0.02	0.00
23	1	-0.05	0.11	-0.01	0.12	0.00	0.00	-0.06	-0.03	0.00
24	6	-0.04	-0.02	0.00	0.08	0.01	0.01	-0.05	0.00	0.00
25	1	-0.04	-0.03	-0.01	0.07	0.02	0.03	-0.05	0.00	-0.01
26	6	-0.03	-0.07	0.01	0.05	0.00	-0.01	-0.04	0.02	0.00
27	1	-0.03	-0.13	0.02	0.04	0.00	-0.01	-0.04	0.04	0.00
28	8	-0.06	-0.02	0.11	0.15	-0.06	0.06	-0.08	-0.01	0.06
29	1	0.04	0.00	0.04	-0.10	0.00	-0.02	0.07	0.00	0.01
30	1	-0.09	-0.03	0.17	0.25	0.02	0.15	-0.14	-0.04	0.22

	13	14	15
	A	A	A
Frequencies --	209.7316	221.9707	275.4302
Red. masses --	10.7709	7.2187	7.2527
Frc consts --	0.2791	0.2096	0.3242
IR Inten --	0.1497	23.5505	1.6640
Raman Activ --	0.1100	2.0388	1.6426
Depolar (P) --	0.7376	0.7385	0.7500
Depolar (U) --	0.8490	0.8496	0.8571

Atom	AN	X	Y	Z	X	Y	Z	X	Y	Z
1	6	0.04	-0.06	0.01	-0.01	0.00	-0.22	-0.17	-0.10	0.03
2	6	0.01	-0.04	0.01	0.00	-0.01	-0.27	-0.01	-0.17	0.06
3	6	-0.02	-0.06	0.00	0.00	-0.01	-0.24	0.16	-0.09	0.03

4	6	-0.01	-0.06	-0.01	0.00	0.00	0.00	0.16	0.06	0.00
5	1	-0.02	-0.06	-0.02	0.00	0.00	0.12	0.27	0.11	-0.03
6	6	0.01	-0.08	0.00	-0.01	0.00	0.20	0.01	0.17	0.00
7	6	0.03	-0.06	0.01	-0.01	0.00	0.03	-0.16	0.06	0.02
8	1	0.04	-0.07	0.01	-0.01	0.00	0.17	-0.29	0.13	0.00
9	6	-0.06	-0.11	0.02	-0.02	0.00	-0.06	-0.11	-0.06	0.00
10	8	-0.09	-0.23	0.06	-0.03	0.05	0.06	-0.07	0.19	-0.03
11	6	0.02	0.25	-0.01	0.01	0.01	0.04	-0.02	-0.07	0.02
12	8	0.01	0.42	-0.03	-0.24	0.01	0.18	0.01	0.11	-0.01
13	8	0.04	0.46	-0.03	0.29	0.02	0.20	-0.03	0.11	-0.01
14	6	0.05	-0.11	0.00	0.00	0.00	-0.06	0.12	-0.05	0.00
15	8	0.06	-0.19	0.03	0.02	0.02	0.07	0.09	0.21	-0.02
16	8	0.14	-0.06	-0.01	-0.01	-0.03	0.04	-0.11	-0.20	-0.01
17	1	0.20	-0.10	0.00	-0.01	-0.02	0.24	-0.28	-0.08	-0.04
18	7	-0.01	-0.09	0.00	-0.01	-0.01	-0.06	0.00	-0.02	0.00
19	1	0.01	0.38	-0.03	-0.25	0.01	0.23	0.01	0.16	-0.03
20	7	-0.01	-0.09	0.00	0.02	-0.01	-0.06	0.00	-0.02	0.00
21	1	-0.01	0.02	-0.01	0.02	-0.01	-0.05	0.00	0.02	0.00
22	6	-0.01	0.01	0.00	0.02	-0.01	-0.05	0.00	0.00	0.00
23	1	0.00	0.04	-0.01	0.03	-0.01	-0.05	0.00	0.01	0.00
24	6	-0.01	0.05	-0.01	0.01	-0.01	-0.04	0.00	0.01	0.00
25	1	-0.01	0.12	-0.01	0.01	-0.01	-0.03	0.00	0.03	0.00
26	6	-0.01	-0.02	0.00	-0.01	-0.01	-0.06	0.00	-0.01	0.00
27	1	-0.02	-0.02	0.00	-0.02	-0.01	-0.05	0.00	0.00	0.00
28	8	-0.17	-0.03	-0.02	0.01	-0.03	0.03	0.11	-0.24	-0.02
29	1	0.01	-0.10	-0.01	-0.01	0.01	0.50	0.01	0.33	-0.02
30	1	-0.26	-0.09	-0.01	0.00	-0.02	0.21	0.31	-0.12	-0.05

	16	17	18
	A	A	A
Frequencies --	313.1193	375.3978	379.9374
Red. masses --	10.5287	7.0131	6.9915
Frc consts --	0.6082	0.5823	0.5946
IR Inten --	0.0122	23.0477	10.8695
Raman Activ --	3.0560	3.9705	1.0029
Depolar (P) --	0.7117	0.1326	0.1402
Depolar (U) --	0.8316	0.2341	0.2459

Atom	AN	X	Y	Z	X	Y	Z	X	Y	Z
1	6	0.02	-0.11	-0.01	-0.04	-0.02	-0.16	-0.04	-0.01	0.17
2	6	-0.13	0.02	-0.01	0.03	0.02	0.05	0.02	-0.02	-0.07
3	6	0.00	0.13	-0.01	-0.06	0.03	-0.09	-0.03	0.01	0.22
4	6	0.00	0.06	0.00	-0.08	-0.02	-0.12	-0.02	0.04	0.05
5	1	-0.08	0.02	0.00	-0.08	-0.01	-0.21	0.01	0.06	0.03
6	6	0.08	-0.02	0.00	-0.08	-0.02	0.14	-0.05	0.06	-0.21
7	6	0.02	-0.06	0.00	-0.05	-0.01	0.00	-0.05	0.05	0.13
8	1	-0.05	-0.02	0.00	-0.03	-0.02	0.05	-0.08	0.07	0.21
9	6	0.08	-0.20	0.00	-0.11	-0.08	-0.10	-0.05	-0.07	0.07
10	8	0.07	-0.29	0.01	-0.11	0.04	0.01	-0.03	-0.02	-0.02
11	6	-0.23	0.01	0.00	0.13	0.01	0.11	0.10	-0.02	-0.16
12	8	-0.28	0.01	0.03	0.35	-0.01	0.00	-0.10	-0.01	-0.07
13	8	-0.25	0.00	0.00	0.01	0.00	0.03	0.38	-0.01	-0.01
14	6	0.07	0.21	0.00	-0.09	0.09	-0.03	-0.09	0.05	0.12
15	8	0.07	0.25	-0.01	-0.08	-0.01	0.02	-0.10	-0.05	-0.02
16	8	0.14	0.28	0.00	0.08	0.23	0.02	0.02	0.17	-0.02
17	1	0.24	0.21	-0.02	0.26	0.10	0.08	0.11	0.08	-0.29
18	7	0.03	0.00	0.00	-0.01	0.00	0.00	-0.02	0.00	0.00
19	1	-0.31	0.00	0.07	0.38	-0.03	-0.14	-0.09	0.01	0.05
20	7	0.05	0.00	0.00	-0.03	0.00	0.01	-0.01	0.00	0.00
21	1	0.07	0.00	-0.03	-0.04	0.00	0.02	-0.02	0.00	0.01
22	6	0.06	0.00	-0.01	-0.03	0.00	0.01	-0.02	0.00	-0.01
23	1	0.06	0.00	-0.01	-0.02	0.00	0.01	-0.03	0.00	-0.01

24	6	0.05	0.00	-0.01	-0.03	0.00	0.01	-0.02	0.00	-0.02
25	1	0.05	0.00	0.00	-0.03	0.00	0.01	-0.01	0.00	-0.02
26	6	0.04	0.00	-0.01	-0.02	0.00	0.02	-0.01	0.00	-0.01
27	1	0.05	0.00	-0.01	-0.03	0.00	0.02	0.00	0.00	-0.01
28	8	0.11	-0.25	0.01	0.05	-0.26	0.01	0.06	-0.15	-0.02
29	1	0.08	-0.03	0.01	-0.08	-0.03	0.35	-0.05	0.07	-0.53
30	1	0.17	-0.21	0.04	0.21	-0.13	0.23	0.19	-0.08	-0.18

19 20 21

A A A

Frequencies -- 409.1344 441.1701 538.9746

Red. masses -- 7.8733 3.3084 7.3089

Frc consts -- 0.7765 0.3794 1.2509

IR Inten -- 8.3638 0.2741 8.7593

Raman Activ -- 3.2014 1.3643 8.5982

Depolar (P) -- 0.7392 0.5095 0.2284

Depolar (U) -- 0.8500 0.6750 0.3718

Atom	AN	X	Y	Z	X	Y	Z	X	Y	Z
1	6	0.07	0.03	-0.04	-0.04	0.00	0.22	0.12	-0.01	0.08
2	6	0.00	0.07	-0.01	-0.01	-0.01	0.00	0.12	-0.03	0.02
3	6	-0.06	0.03	-0.06	-0.03	0.00	-0.22	0.14	-0.02	-0.08
4	6	-0.02	0.30	-0.01	-0.04	0.00	0.15	0.24	-0.02	0.01
5	1	0.09	0.35	0.01	-0.03	0.00	0.41	0.25	-0.03	0.05
6	6	0.02	0.32	0.04	-0.05	0.00	0.00	0.29	0.00	0.01
7	6	0.05	0.29	-0.04	-0.05	-0.01	-0.15	0.20	0.00	-0.03
8	1	-0.08	0.37	-0.05	-0.05	-0.01	-0.40	0.18	0.02	-0.10
9	6	0.11	-0.09	0.00	-0.01	-0.01	0.14	-0.13	-0.02	0.03
10	8	0.10	-0.24	0.01	0.02	-0.03	-0.04	-0.11	0.20	-0.02
11	6	0.00	0.08	0.02	0.02	-0.01	0.00	-0.02	-0.02	0.00
12	8	0.03	-0.01	0.01	0.05	0.00	-0.01	-0.13	0.02	0.06
13	8	-0.05	-0.02	0.01	0.05	0.00	0.01	-0.12	0.00	-0.06
14	6	-0.11	-0.10	-0.02	-0.01	0.02	-0.14	-0.16	0.04	-0.03
15	8	-0.10	-0.23	0.01	0.02	0.04	0.05	-0.16	-0.20	0.02
16	8	-0.15	-0.13	0.01	0.02	0.02	0.01	-0.07	0.15	0.01
17	1	-0.21	-0.08	0.11	0.11	-0.01	0.47	0.09	0.06	0.35
18	7	0.00	0.00	0.00	0.00	0.00	0.00	0.00	0.00	0.00
19	1	0.03	-0.05	-0.01	0.05	0.00	-0.03	-0.14	0.04	0.14
20	7	0.00	0.00	0.00	0.00	0.00	0.00	0.01	0.00	0.00
21	1	0.00	-0.01	0.00	-0.01	0.00	0.01	0.02	0.00	-0.02
22	6	0.00	0.00	0.00	-0.01	0.00	0.00	0.01	0.00	0.00
23	1	0.00	0.00	0.00	-0.01	0.00	0.00	0.02	0.00	0.00
24	6	0.00	0.00	0.00	0.00	0.00	0.00	0.01	0.00	0.00
25	1	0.00	0.00	0.00	0.00	0.00	0.00	0.01	0.00	0.01
26	6	0.00	0.00	0.00	0.00	0.00	0.00	0.00	0.00	-0.01
27	1	0.00	0.00	0.00	0.00	0.00	0.00	0.00	0.00	-0.01
28	8	0.13	-0.11	0.02	0.01	-0.01	-0.01	-0.09	-0.10	-0.01
29	1	0.01	0.25	0.11	-0.05	0.00	0.00	0.29	0.02	0.02
30	1	0.17	-0.08	0.07	0.11	0.01	-0.47	0.04	-0.05	-0.36

22 23 24

A A A

Frequencies -- 545.9700 573.8281 598.5294

Red. masses -- 5.8333 1.3600 2.6885

Frc consts -- 1.0245 0.2639 0.5675

IR Inten -- 24.4800 142.4403 21.5317

Raman Activ -- 1.2492 0.2693 0.1856

Depolar (P) -- 0.6662 0.7477 0.7360

Depolar (U) -- 0.7997 0.8556 0.8479

Atom	AN	X	Y	Z	X	Y	Z	X	Y	Z
1	6	0.11	0.11	0.05	-0.01	-0.01	0.00	0.01	0.02	0.08
2	6	0.02	0.22	-0.01	0.00	-0.03	-0.01	0.00	0.02	0.20
3	6	-0.08	0.12	0.02	0.01	-0.01	0.01	-0.01	0.01	0.09

4	6	-0.15	0.02	0.09	0.02	0.00	0.07	-0.02	0.00	-0.12
5	1	-0.28	-0.05	0.14	0.04	0.01	0.14	-0.03	0.00	-0.47
6	6	0.02	-0.09	-0.09	0.00	0.01	-0.08	-0.01	0.00	0.17
7	6	0.19	0.01	0.08	-0.02	0.00	0.07	0.01	0.00	-0.12
8	1	0.32	-0.07	0.10	-0.04	0.01	0.15	0.03	-0.01	-0.45
9	6	-0.14	-0.01	-0.04	0.02	0.00	-0.05	0.00	0.00	-0.03
10	8	-0.16	0.07	-0.02	0.01	0.00	0.00	-0.01	0.00	0.00
11	6	0.00	0.20	-0.02	0.00	-0.02	0.02	0.01	-0.01	-0.08
12	8	-0.01	-0.05	0.02	0.02	0.01	0.01	-0.09	-0.01	-0.05
13	8	-0.02	-0.07	0.01	-0.02	0.01	0.01	0.10	-0.01	-0.03
14	6	0.12	-0.01	-0.05	-0.02	0.00	-0.05	0.01	0.00	-0.03
15	8	0.14	0.02	-0.01	-0.01	0.00	0.00	0.01	0.00	0.00
16	8	-0.03	-0.17	-0.02	0.00	0.02	-0.04	-0.01	-0.01	-0.02
17	1	-0.23	-0.01	0.28	0.13	-0.03	0.67	0.04	-0.01	0.49
18	7	0.00	0.00	0.00	0.00	0.00	0.00	-0.01	0.00	0.00
19	1	-0.01	-0.16	0.04	0.02	0.02	-0.01	-0.09	0.00	0.04
20	7	0.00	0.02	0.00	0.00	0.00	0.00	0.00	0.00	0.00
21	1	0.00	-0.05	0.00	0.00	0.00	0.00	0.01	0.00	0.00
22	6	0.00	0.00	0.00	0.00	0.00	0.00	0.00	0.00	0.00
23	1	0.00	-0.01	0.00	0.00	0.00	0.00	0.00	0.00	0.00
24	6	0.00	0.00	0.00	0.00	0.00	0.00	0.00	0.00	0.00
25	1	0.00	-0.01	0.00	0.00	0.00	0.00	0.00	0.00	0.00
26	6	0.00	0.01	0.00	0.00	0.00	0.00	0.00	0.00	0.00
27	1	0.00	0.01	0.00	0.00	0.00	0.00	0.00	0.00	0.00
28	8	0.00	-0.20	-0.01	0.01	0.02	-0.04	0.01	-0.01	-0.02
29	1	0.01	-0.29	-0.25	0.00	0.04	-0.18	-0.01	-0.02	0.16
30	1	0.24	-0.03	0.13	-0.16	-0.03	0.63	-0.05	-0.01	0.40

	25	26	27
	A	A	A
Frequencies --	604.9771	629.3275	655.7220
Red. masses --	1.3317	3.4156	4.6756
Frc consts --	0.2872	0.7970	1.1845
IR Inten --	4.3369	11.3077	132.3157
Raman Activ --	3.7900	0.1452	0.4104
Depolar (P) --	0.7251	0.7498	0.7500
Depolar (U) --	0.8406	0.8570	0.8571

Atom	AN	X	Y	Z	X	Y	Z	X	Y	Z
1	6	0.00	0.00	-0.10	0.00	0.00	0.00	0.10	-0.11	0.01
2	6	-0.01	0.00	-0.01	0.00	0.00	0.00	0.00	-0.07	0.01
3	6	0.00	0.00	0.09	0.00	0.00	0.00	-0.11	-0.11	0.00
4	6	-0.01	0.00	-0.02	0.00	0.00	0.00	-0.13	0.11	0.00
5	1	-0.01	0.00	-0.09	0.01	0.00	0.00	-0.06	0.14	-0.01
6	6	-0.01	0.00	0.00	0.00	0.00	0.00	0.00	0.05	0.00
7	6	-0.01	0.00	0.02	0.00	0.00	0.00	0.14	0.10	0.00
8	1	-0.01	0.00	0.11	-0.01	0.00	0.00	0.07	0.14	-0.02
9	6	0.01	0.00	0.01	0.00	0.00	0.00	0.06	-0.14	0.00
10	8	0.01	-0.01	0.02	0.00	0.00	0.00	0.10	0.17	0.03
11	6	0.00	0.00	0.00	0.00	0.00	0.00	0.00	0.04	-0.01
12	8	0.01	0.00	0.00	0.00	0.00	0.00	0.00	-0.02	0.00
13	8	0.00	0.00	0.00	0.00	0.00	0.00	0.00	-0.02	0.00
14	6	0.01	0.00	0.00	0.00	0.00	0.00	-0.07	-0.14	0.00
15	8	0.00	0.01	-0.02	0.00	0.00	0.00	-0.09	0.18	0.02
16	8	0.00	-0.01	-0.05	0.00	0.00	0.00	0.15	-0.05	-0.02
17	1	0.10	-0.03	0.65	0.00	0.00	0.01	0.47	-0.29	-0.10
18	7	0.00	0.00	0.00	0.00	-0.06	0.01	0.00	-0.01	0.00
19	1	0.01	0.00	-0.01	0.00	-0.03	0.00	0.00	-0.02	0.00
20	7	0.00	0.00	0.00	0.00	0.23	-0.03	0.00	0.01	0.00
21	1	0.00	0.00	0.00	0.00	-0.07	0.01	0.00	0.00	0.00
22	6	0.00	0.00	0.00	0.00	-0.26	0.03	0.00	0.00	0.00
23	1	0.00	0.00	0.00	0.01	-0.71	0.09	0.00	0.00	0.00

```
  24   6     0.00   0.00   0.00     0.00   0.25  -0.03     0.00   0.00   0.00
  25   1     0.00   0.00   0.00    -0.01   0.37  -0.05     0.00   0.00   0.00
  26   6     0.00   0.00   0.00     0.00  -0.12   0.02     0.00   0.00   0.00
  27   1     0.00   0.00   0.00     0.00  -0.37   0.05     0.00   0.01   0.00
  28   8    -0.01   0.01   0.05     0.00   0.00   0.00    -0.15  -0.04  -0.03
  29   1    -0.01   0.00   0.00     0.00   0.01   0.00     0.00  -0.16  -0.01
  30   1     0.13   0.03  -0.71     0.00   0.00  -0.01    -0.49  -0.26  -0.12
                    28                     29                     30
                     A                      A                      A
 Frequencies --    671.7280               674.4960               736.3658
 Red. masses --      4.7341                 5.4219                 5.4493
 Frc consts  --      1.2586                 1.4533                 1.7409
 IR Inten    --     10.4683                 0.3058                79.0514
 Raman Activ --      5.0313                 0.3383                 0.1718
 Depolar (P) --      0.1604                 0.7155                 0.6001
 Depolar (U) --      0.2764                 0.8342                 0.7501
  Atom  AN      X      Y      Z        X      Y      Z        X      Y      Z
   1   6     0.04   0.13   0.00     0.01   0.00   0.00    -0.01   0.00   0.08
   2   6     0.17  -0.01   0.00    -0.01   0.00   0.00     0.01   0.02  -0.27
   3   6     0.03  -0.14   0.00    -0.01   0.01   0.00     0.02   0.00   0.08
   4   6     0.01  -0.12   0.00    -0.01   0.01   0.00     0.02  -0.02  -0.13
   5   1     0.18  -0.03   0.01    -0.03   0.01   0.00     0.02  -0.02   0.07
   6   6    -0.16   0.01   0.00     0.01   0.00   0.00     0.00   0.00   0.07
   7   6     0.03   0.12   0.00     0.01   0.00   0.00    -0.02  -0.01  -0.13
   8   1     0.18   0.02   0.01     0.01   0.00   0.00    -0.01  -0.02   0.07
   9   6    -0.07   0.12  -0.01     0.00  -0.01   0.00    -0.05  -0.02   0.31
  10   8    -0.11  -0.12  -0.02     0.00   0.01   0.00     0.02   0.01  -0.09
  11   6     0.10  -0.01   0.00     0.00  -0.02   0.00     0.00   0.13   0.02
  12   8    -0.08   0.02   0.11     0.00  -0.01   0.00     0.05  -0.02   0.04
  13   8    -0.08  -0.01  -0.11     0.00   0.00   0.00    -0.05  -0.03   0.02
  14   6    -0.08  -0.11   0.00     0.01   0.00   0.00     0.04  -0.02   0.31
  15   8    -0.10   0.12   0.02     0.01   0.00   0.00    -0.02   0.01  -0.09
  16   8     0.13  -0.03  -0.02     0.00   0.00   0.00    -0.01  -0.01  -0.10
  17   1     0.44  -0.26  -0.07    -0.03   0.01  -0.01     0.08  -0.04   0.40
  18   7    -0.01   0.02   0.00     0.00   0.39  -0.05     0.00   0.01   0.00
  19   1    -0.07   0.05   0.26     0.00   0.17  -0.04     0.05  -0.09   0.00
  20   7     0.00  -0.01   0.00     0.00  -0.27   0.03     0.00   0.00   0.00
  21   1     0.02   0.00  -0.03     0.00   0.13  -0.02     0.00  -0.05   0.01
  22   6     0.01   0.00   0.00     0.00   0.03   0.00     0.00   0.00   0.00
  23   1     0.01   0.00   0.00     0.00   0.03   0.00     0.00   0.01   0.00
  24   6     0.01   0.01   0.00     0.00   0.18  -0.02     0.00   0.00   0.00
  25   1     0.01   0.01   0.00     0.00   0.27  -0.03     0.00   0.01   0.00
  26   6     0.00  -0.01  -0.01     0.00  -0.31   0.04     0.00  -0.01   0.00
  27   1     0.00  -0.03   0.00     0.01  -0.71   0.09     0.00  -0.01   0.00
  28   8     0.13   0.02   0.03    -0.01   0.00   0.00     0.01  -0.01  -0.10
  29   1    -0.16   0.00  -0.01     0.01  -0.02  -0.01     0.00   0.04   0.49
  30   1     0.45   0.23   0.08    -0.02  -0.01  -0.01    -0.11  -0.04   0.41
                    31                     32                     33
                     A                      A                      A
 Frequencies --    744.1085               765.9607               768.1628
 Red. masses --      4.9917                 3.1659                 1.3711
 Frc consts  --      1.6284                 1.0944                 0.4767
 IR Inten    --      9.4573                31.9210                37.5534
 Raman Activ --     18.9309                 0.8721                 0.0774
 Depolar (P) --      0.0283                 0.7397                 0.7478
 Depolar (U) --      0.0551                 0.8504                 0.8557
  Atom  AN      X      Y      Z        X      Y      Z        X      Y      Z
   1   6    -0.03   0.00   0.02    -0.06  -0.01   0.01     0.02   0.00   0.00
   2   6    -0.14   0.01  -0.01     0.00   0.03  -0.01     0.00  -0.01   0.01
   3   6    -0.03   0.01  -0.01     0.06  -0.02   0.01    -0.02   0.00   0.00
```

4	6	0.06	0.09	0.00	0.11	-0.07	0.02	-0.03	0.02	-0.01
5	1	-0.04	0.03	-0.01	0.15	-0.05	-0.19	-0.05	0.02	0.06
6	6	0.26	-0.01	0.01	0.00	0.02	0.03	0.00	-0.01	-0.01
7	6	0.04	-0.09	0.00	-0.12	-0.06	0.02	0.04	0.02	-0.01
8	1	-0.05	-0.03	-0.03	-0.15	-0.04	-0.17	0.05	0.01	0.06
9	6	-0.08	0.12	-0.02	0.04	-0.02	-0.06	-0.01	0.01	0.02
10	8	-0.12	-0.07	-0.02	0.03	0.00	0.03	-0.01	0.00	-0.01
11	6	-0.16	0.02	0.02	0.01	0.33	-0.04	0.00	-0.10	0.01
12	8	0.05	-0.03	-0.17	0.00	-0.06	0.02	0.00	0.02	-0.01
13	8	0.06	0.02	0.17	-0.01	-0.07	0.01	0.00	0.03	0.00
14	6	-0.09	-0.11	0.03	-0.04	-0.01	-0.07	0.01	0.00	0.02
15	8	-0.12	0.08	0.02	-0.03	0.01	0.03	0.01	0.00	-0.01
16	8	0.10	-0.07	-0.02	0.02	-0.01	0.02	-0.01	0.00	0.00
17	1	0.39	-0.28	-0.06	0.08	-0.06	-0.07	-0.03	0.02	0.02
18	7	0.01	0.00	0.00	0.00	0.04	-0.01	0.00	0.02	0.00
19	1	0.05	-0.06	-0.37	0.00	-0.21	0.04	0.00	0.07	-0.01
20	7	0.00	0.00	0.01	0.00	0.02	0.00	0.00	0.04	0.00
21	1	-0.01	0.00	0.03	0.00	-0.26	0.03	0.00	-0.12	0.02
22	6	-0.01	0.00	0.00	0.00	-0.03	0.00	0.00	-0.07	0.01
23	1	-0.01	0.00	0.00	0.00	0.30	-0.04	-0.01	0.45	-0.06
24	6	-0.01	0.00	0.00	0.00	-0.06	0.01	0.00	-0.08	0.01
25	1	-0.01	0.00	-0.01	-0.01	0.56	-0.07	-0.01	0.81	-0.10
26	6	0.00	0.00	0.01	0.00	-0.04	0.00	0.00	-0.03	0.00
27	1	0.00	0.00	0.01	0.00	0.14	-0.02	0.00	0.24	-0.03
28	8	0.10	0.06	0.03	-0.03	-0.01	0.01	0.01	0.00	0.00
29	1	0.26	0.00	-0.01	0.00	0.24	-0.25	0.00	-0.07	0.08
30	1	0.39	0.26	0.09	-0.09	-0.06	-0.07	0.03	0.02	0.02

	34	35	36
	A	A	A
Frequencies --	789.5997	804.7294	825.3019
Red. masses --	1.7758	7.5816	1.1813
Frc consts --	0.6523	2.8927	0.4741
IR Inten --	47.2697	2.4221	64.8777
Raman Activ --	0.3418	0.1789	0.1976
Depolar (P) --	0.7483	0.5356	0.7090
Depolar (U) --	0.8561	0.6976	0.8297

Atom	AN	X	Y	Z	X	Y	Z	X	Y	Z
1	6	-0.01	0.00	-0.05	0.00	-0.01	-0.24	0.00	0.00	0.00
2	6	0.00	0.01	0.19	0.00	0.00	0.02	0.00	0.01	0.00
3	6	0.01	0.00	-0.05	0.00	0.01	0.23	0.00	0.00	0.00
4	6	0.02	-0.01	-0.03	0.00	0.01	0.03	0.01	-0.01	0.00
5	1	0.03	0.00	0.51	-0.02	-0.01	-0.35	0.01	-0.01	0.00
6	6	0.00	0.00	-0.11	0.04	0.00	-0.01	0.00	0.00	0.00
7	6	-0.02	-0.01	-0.03	0.01	-0.01	-0.03	-0.01	0.00	0.00
8	1	-0.03	0.00	0.49	0.00	0.01	0.40	0.00	0.00	0.01
9	6	0.00	0.00	0.02	-0.09	-0.01	0.45	0.00	0.00	0.01
10	8	0.01	0.00	0.00	0.02	0.00	-0.13	0.00	0.00	0.00
11	6	0.01	0.08	-0.04	0.00	0.00	0.00	0.00	0.02	0.00
12	8	-0.03	-0.02	-0.02	0.00	0.00	0.00	0.00	0.00	0.00
13	8	0.03	-0.02	-0.01	0.00	0.00	0.00	0.00	-0.02	0.00
14	6	0.00	0.00	0.03	-0.07	0.01	-0.44	0.00	0.00	-0.01
15	8	-0.01	0.00	-0.01	0.01	0.00	0.12	0.00	0.00	0.00
16	8	0.00	0.00	-0.01	0.02	-0.01	0.11	0.00	0.00	0.00
17	1	0.02	-0.01	0.00	-0.02	0.00	-0.25	0.00	0.00	-0.01
18	7	0.00	0.00	0.00	0.00	0.00	0.00	0.00	-0.03	0.00
19	1	-0.04	-0.05	0.03	0.00	0.02	-0.01	0.00	-0.10	0.01
20	7	0.00	0.00	0.00	0.00	0.00	0.00	0.00	-0.03	0.00
21	1	0.01	-0.05	0.00	0.00	-0.08	0.01	-0.01	0.90	-0.12
22	6	0.00	0.00	0.00	0.00	0.01	0.00	0.00	-0.10	0.01
23	1	0.00	-0.02	0.00	0.00	-0.03	0.00	0.00	0.36	-0.05

24	6	0.00	0.00	0.00	0.00	0.00	0.00	0.00	0.03	0.00
25	1	0.00	-0.01	0.00	0.00	-0.01	0.00	0.00	0.02	0.00
26	6	0.00	0.00	0.00	0.00	0.00	0.00	0.00	0.05	-0.01
27	1	0.00	-0.01	0.00	0.00	0.01	0.00	0.00	-0.13	0.02
28	8	0.00	0.00	0.00	0.03	0.02	-0.11	0.00	0.00	0.00
29	1	0.01	0.05	0.65	0.04	-0.01	0.03	0.00	0.02	0.01
30	1	-0.01	-0.01	-0.01	-0.03	0.01	0.26	-0.01	-0.01	0.01

	37	38	39
	A	A	A
Frequencies --	832.0654	863.0575	886.9482
Red. masses --	6.0185	3.7533	1.1754
Frc consts --	2.4550	1.6472	0.5448
IR Inten --	10.0322	0.4857	18.4837
Raman Activ --	4.9925	0.4649	0.3703
Depolar (P) --	0.0576	0.7499	0.7380
Depolar (U) --	0.1088	0.8571	0.8493

Atom	AN	X	Y	Z	X	Y	Z	X	Y	Z
1	6	-0.10	-0.08	0.00	0.00	0.01	0.22	0.00	-0.01	0.00
2	6	-0.06	0.00	0.00	0.00	0.00	-0.20	0.00	-0.01	0.00
3	6	-0.09	0.09	-0.01	0.00	0.01	0.22	0.00	-0.01	0.00
4	6	-0.09	0.23	0.00	0.00	0.00	-0.11	0.00	0.00	0.00
5	1	-0.32	0.11	-0.01	0.00	0.00	0.29	0.00	0.01	0.00
6	6	0.25	-0.01	0.00	0.00	0.00	-0.05	0.00	0.00	0.00
7	6	-0.11	-0.22	0.00	0.00	0.00	-0.10	0.00	0.00	0.00
8	1	-0.34	-0.08	0.00	-0.01	0.00	0.28	0.00	0.01	0.00
9	6	-0.02	0.03	-0.01	0.03	0.01	-0.18	0.00	0.00	0.00
10	8	-0.03	-0.02	0.00	-0.01	0.00	0.05	0.00	0.00	0.00
11	6	0.25	-0.01	-0.04	0.00	-0.03	0.02	0.00	0.00	0.00
12	8	0.01	0.03	0.24	0.03	0.01	0.02	0.00	0.00	0.00
13	8	0.00	-0.03	-0.24	-0.03	0.01	0.01	0.00	0.01	0.00
14	6	-0.02	-0.03	0.02	-0.03	0.01	-0.19	0.00	0.00	0.00
15	8	-0.03	0.02	0.00	0.01	0.00	0.05	0.00	0.00	0.00
16	8	0.02	-0.04	-0.01	0.01	-0.01	0.04	0.00	0.00	0.00
17	1	0.06	-0.08	-0.01	-0.01	-0.01	-0.06	-0.01	0.01	0.00
18	7	-0.01	0.00	0.00	0.00	0.00	0.00	0.00	0.01	0.00
19	1	0.03	0.08	0.51	0.03	0.02	-0.02	0.00	0.02	0.00
20	7	0.00	0.00	-0.01	0.00	0.00	0.00	0.00	0.02	0.00
21	1	0.01	0.04	-0.04	-0.01	-0.01	0.00	0.00	-0.47	0.06
22	6	0.01	0.00	0.00	0.00	0.00	0.00	0.00	-0.09	0.01
23	1	0.01	0.02	0.00	0.00	-0.02	0.00	0.00	0.65	-0.08
24	6	0.01	0.00	0.00	0.00	0.00	0.00	0.00	0.04	-0.01
25	1	0.01	0.00	0.02	0.00	0.01	0.00	0.00	-0.33	0.04
26	6	-0.01	0.00	-0.01	0.00	0.00	0.00	0.00	0.06	-0.01
27	1	-0.01	-0.01	-0.01	0.00	0.01	0.00	0.00	-0.47	0.06
28	8	0.03	0.04	0.01	-0.01	-0.01	0.04	0.00	0.00	0.00
29	1	0.25	-0.01	-0.02	0.00	0.01	0.76	0.00	-0.01	0.00
30	1	0.07	0.07	0.02	0.01	0.00	-0.06	0.01	0.01	0.00

	40	41	42
	A	A	A
Frequencies --	902.7842	918.2416	941.1231
Red. masses --	6.3392	1.3441	6.3637
Frc consts --	3.0440	0.6677	3.3209
IR Inten --	4.9920	8.8771	3.1181
Raman Activ --	5.3333	0.1183	5.4465
Depolar (P) --	0.7474	0.7402	0.7431
Depolar (U) --	0.8554	0.8507	0.8526

Atom	AN	X	Y	Z	X	Y	Z	X	Y	Z
1	6	-0.05	0.25	-0.02	0.00	0.00	0.00	0.00	0.00	0.00
2	6	0.01	0.29	0.01	0.00	0.00	0.00	0.00	0.00	0.00
3	6	0.06	0.25	-0.02	0.00	0.00	0.00	0.00	0.00	0.00

4	6	0.11	-0.08	0.01	0.00	0.00	0.00	0.00	0.00	0.00
5	1	-0.05	-0.16	-0.01	0.00	0.00	0.00	0.00	0.00	0.00
6	6	0.00	-0.02	0.00	0.00	0.00	0.00	0.00	0.00	0.00
7	6	-0.12	-0.08	0.01	0.00	0.00	0.00	0.00	0.00	0.00
8	1	0.04	-0.18	-0.01	0.00	0.00	0.00	0.00	0.00	0.00
9	6	0.08	-0.04	0.04	0.00	0.00	0.00	0.00	0.00	0.00
10	8	0.13	0.01	0.02	0.00	0.00	0.00	0.00	0.00	0.00
11	6	0.00	-0.24	0.03	0.00	-0.01	0.00	0.00	0.00	0.01
12	8	-0.01	0.03	-0.01	0.00	0.00	0.00	0.01	0.00	-0.02
13	8	0.01	0.05	-0.01	0.00	0.00	0.00	0.00	0.00	0.01
14	6	-0.09	-0.03	0.03	0.00	0.00	0.00	0.00	0.00	0.00
15	8	-0.12	0.02	0.01	0.00	0.00	0.00	0.00	0.00	0.00
16	8	0.10	-0.16	-0.03	0.00	0.00	0.00	0.00	0.00	0.00
17	1	0.27	-0.29	-0.05	0.00	0.00	0.00	0.00	0.00	0.00
18	7	0.00	0.00	0.00	0.00	-0.01	0.00	-0.16	-0.01	-0.04
19	1	-0.01	0.16	-0.02	0.00	-0.06	0.01	0.01	0.00	0.01
20	7	0.00	0.01	0.00	0.00	-0.01	0.00	-0.18	0.00	0.01
21	1	0.00	-0.10	0.01	0.00	0.01	0.00	-0.04	-0.04	-0.22
22	6	0.00	-0.01	0.00	0.00	0.05	-0.01	-0.06	0.04	0.36
23	1	0.00	0.09	-0.01	0.00	-0.33	0.04	-0.26	0.05	0.31
24	6	0.00	0.01	0.00	0.00	-0.10	0.01	0.42	0.01	0.03
25	1	0.00	-0.06	0.01	0.00	0.43	-0.05	0.44	-0.01	-0.02
26	6	0.00	0.00	0.00	0.00	0.13	-0.02	0.01	-0.05	-0.32
27	1	0.00	-0.02	0.00	0.01	-0.81	0.10	-0.12	-0.02	-0.32
28	8	-0.11	-0.16	-0.03	0.00	0.00	0.00	0.00	0.00	0.00
29	1	0.01	0.34	-0.02	0.00	0.00	0.00	0.00	0.00	0.00
30	1	-0.29	-0.27	-0.07	0.00	0.00	0.00	0.00	0.00	0.00

	43	44	45
	A	A	A
Frequencies --	956.6282	981.5591	1008.6459
Red. masses --	3.1237	1.4044	1.0998
Frc consts --	1.6843	0.7972	0.6592
IR Inten --	40.3462	0.0173	103.4630
Raman Activ --	3.1298	0.0111	0.2430
Depolar (P) --	0.3394	0.7467	0.7203
Depolar (U) --	0.5068	0.8550	0.8374

Atom	AN	X	Y	Z	X	Y	Z	X	Y	Z
1	6	0.00	0.00	0.00	0.00	0.00	-0.04	0.00	0.00	0.00
2	6	0.00	0.00	-0.01	0.00	0.00	0.00	0.00	0.00	0.00
3	6	0.00	0.00	0.00	0.00	0.00	0.04	0.00	0.00	0.00
4	6	0.00	0.00	0.00	0.00	0.00	-0.13	0.00	0.00	0.00
5	1	0.00	0.00	0.00	0.01	0.01	0.71	0.00	0.00	0.00
6	6	0.00	0.00	0.00	0.00	0.00	0.01	0.00	0.00	0.00
7	6	0.00	0.00	0.00	0.00	0.00	0.12	0.00	0.00	0.00
8	1	0.00	0.00	0.00	0.01	-0.02	-0.67	0.00	0.00	0.01
9	6	0.00	0.00	0.00	-0.01	0.00	0.03	0.00	0.00	0.00
10	8	0.00	0.00	0.00	0.00	0.00	-0.01	0.00	0.00	0.00
11	6	0.00	0.00	0.00	0.00	0.00	0.00	0.00	0.04	-0.01
12	8	0.02	0.00	-0.01	0.00	0.00	0.00	0.00	-0.06	0.01
13	8	-0.01	0.00	0.01	0.00	0.00	0.00	0.00	-0.02	0.00
14	6	0.00	0.00	0.00	0.00	0.00	-0.03	0.00	0.00	0.00
15	8	0.00	0.00	0.00	0.00	0.00	0.01	0.00	0.00	0.00
16	8	0.00	0.00	0.00	0.00	0.00	0.00	0.00	0.00	0.00
17	1	0.00	0.00	0.00	-0.01	0.00	-0.01	-0.01	0.01	0.00
18	7	-0.20	0.02	0.14	0.00	0.00	0.00	0.00	-0.03	0.00
19	1	0.00	-0.02	-0.02	0.00	0.01	0.00	0.01	0.98	-0.13
20	7	0.16	0.02	0.16	0.00	0.00	0.00	0.00	0.00	0.00
21	1	0.33	-0.01	-0.05	0.00	0.00	0.00	0.00	0.07	-0.01
22	6	0.10	-0.02	-0.12	0.00	0.00	0.00	0.00	0.00	0.00
23	1	-0.43	-0.03	-0.27	0.00	0.00	0.00	0.00	0.01	0.00

24	6	0.01	-0.01	-0.07	0.00	0.00	0.00	0.00	0.00	0.00
25	1	-0.03	0.06	0.58	0.00	0.00	0.00	0.00	0.03	0.00
26	6	-0.11	-0.02	-0.15	0.00	0.00	0.00	0.00	0.01	0.00
27	1	0.27	-0.03	-0.21	0.00	0.00	0.00	0.00	-0.04	0.00
28	8	0.00	0.00	0.00	0.00	0.00	0.00	0.00	0.00	0.00
29	1	0.00	0.00	0.00	0.00	0.00	-0.05	0.00	0.00	0.00
30	1	0.00	0.00	0.00	-0.01	0.00	0.00	0.01	0.01	0.00

	46	47	48
	A	A	A
Frequencies --	1022.4054	1068.0212	1079.5025
Red. masses --	1.3594	1.3951	1.5005
Frc consts --	0.8372	0.9376	1.0303
IR Inten --	0.8597	14.1728	89.9351
Raman Activ --	0.0266	2.9769	1.7822
Depolar (P) --	0.4602	0.5927	0.7480
Depolar (U) --	0.6303	0.7443	0.8558

Atom	AN	X	Y	Z	X	Y	Z	X	Y	Z
1	6	0.00	0.00	0.01	0.00	0.00	0.00	0.00	0.00	0.00
2	6	0.00	0.00	0.02	0.00	0.00	0.00	0.01	0.00	0.00
3	6	0.00	0.00	0.01	0.00	0.00	0.00	0.00	0.00	0.00
4	6	0.00	0.00	-0.08	0.00	0.00	0.00	0.00	0.01	0.00
5	1	0.01	0.00	0.47	0.00	-0.01	0.00	0.01	0.01	0.00
6	6	0.00	0.00	0.12	0.00	0.00	0.00	0.00	0.00	0.00
7	6	0.00	0.00	-0.09	0.00	0.00	0.00	0.00	-0.01	0.00
8	1	-0.01	0.02	0.55	0.00	0.01	0.00	0.01	-0.02	0.00
9	6	0.00	0.00	-0.01	0.00	0.00	0.00	0.00	0.00	0.00
10	8	0.00	0.00	0.00	0.00	0.00	0.00	0.00	0.00	0.00
11	6	0.00	0.00	0.00	0.00	0.00	0.00	0.00	0.00	0.00
12	8	0.00	0.00	0.00	0.00	0.00	0.00	0.00	0.00	-0.01
13	8	0.00	0.00	0.00	0.00	0.00	0.00	0.00	0.00	0.01
14	6	0.00	0.00	-0.01	0.00	0.00	0.00	0.00	0.00	0.00
15	8	0.00	0.00	0.00	0.00	0.00	0.00	0.00	0.00	0.00
16	8	0.00	0.00	0.00	0.00	0.00	0.00	0.00	0.00	0.00
17	1	0.00	0.00	-0.01	0.00	0.00	0.00	0.01	0.00	0.00
18	7	0.00	0.00	0.00	0.06	0.00	0.00	-0.09	0.01	0.12
19	1	0.00	-0.01	0.00	0.01	0.00	0.03	0.00	0.01	0.01
20	7	0.00	0.00	0.00	-0.08	-0.01	-0.05	-0.02	-0.01	-0.05
21	1	0.00	0.00	0.00	-0.20	0.01	0.09	0.01	-0.01	-0.10
22	6	0.00	0.00	0.00	-0.01	0.00	-0.02	0.07	0.01	0.04
23	1	0.00	0.00	0.00	0.14	0.00	0.02	0.78	0.03	0.23
24	6	0.00	0.00	0.00	-0.05	0.01	0.08	-0.06	-0.01	-0.06
25	1	0.00	0.00	0.00	-0.10	0.10	0.83	-0.04	-0.04	-0.33
26	6	0.00	0.00	0.00	0.07	-0.01	-0.07	0.00	0.00	-0.04
27	1	0.00	0.00	0.00	0.41	-0.01	-0.13	0.41	-0.01	-0.10
28	8	0.00	0.00	0.00	0.00	0.00	0.00	0.00	0.00	0.00
29	1	0.00	-0.01	-0.67	0.00	0.00	0.00	0.00	0.00	0.00
30	1	0.01	0.00	0.00	0.00	0.00	0.00	0.00	0.00	0.00

	49	50	51
	A	A	A
Frequencies --	1099.9775	1147.6967	1178.6511
Red. masses --	3.6664	5.4318	2.1339
Frc consts --	2.6137	4.2155	1.7466
IR Inten --	36.2416	45.9180	100.7700
Raman Activ --	25.1438	7.0272	18.2634
Depolar (P) --	0.0827	0.0528	0.1640
Depolar (U) --	0.1527	0.1004	0.2818

Atom	AN	X	Y	Z	X	Y	Z	X	Y	Z
1	6	0.04	0.10	0.00	-0.11	0.21	-0.01	0.01	0.00	0.00
2	6	0.19	-0.01	-0.01	0.00	0.00	0.00	0.16	-0.02	-0.01
3	6	0.03	-0.10	0.00	-0.13	-0.20	0.01	0.02	0.01	0.00

4	6	-0.05	0.25	0.00	-0.05	-0.12	0.00	-0.05	0.02	0.00
5	1	0.32	0.46	-0.01	-0.30	-0.27	0.02	-0.22	-0.06	0.00
6	6	-0.07	0.01	0.00	0.23	-0.02	0.00	0.04	-0.03	0.00
7	6	-0.07	-0.24	0.00	-0.05	0.12	0.00	-0.07	0.01	0.00
8	1	0.37	-0.53	0.02	-0.40	0.36	-0.03	-0.48	0.26	-0.02
9	6	0.02	0.07	0.01	0.08	0.17	0.02	0.00	0.00	0.00
10	8	0.02	0.00	0.00	0.11	-0.03	0.02	0.01	0.00	0.00
11	6	0.01	0.01	0.03	0.01	0.00	0.00	0.03	0.01	0.04
12	8	-0.04	-0.01	-0.07	-0.01	0.00	-0.01	-0.04	-0.01	-0.07
13	8	-0.03	0.01	0.06	0.00	0.00	0.01	-0.03	0.01	0.04
14	6	0.02	-0.08	-0.01	0.06	-0.17	-0.01	-0.02	0.07	0.01
15	8	0.03	0.00	0.00	0.11	0.03	-0.02	-0.03	-0.01	0.00
16	8	-0.05	0.06	0.01	-0.11	0.14	0.02	0.05	-0.04	-0.01
17	1	0.08	-0.03	-0.01	0.20	-0.08	-0.04	-0.17	0.11	0.03
18	7	0.01	0.00	-0.01	0.00	0.00	-0.01	0.01	0.01	0.06
19	1	-0.04	-0.01	-0.06	0.00	0.00	0.00	-0.03	0.00	0.04
20	7	0.00	0.00	0.00	0.01	0.00	-0.01	-0.08	0.00	0.01
21	1	0.00	0.00	0.00	0.04	-0.01	-0.05	-0.25	0.03	0.23
22	6	-0.01	0.00	0.00	-0.01	0.00	0.01	0.07	-0.01	-0.09
23	1	-0.08	0.00	-0.02	-0.06	0.00	0.00	0.24	0.00	-0.06
24	6	0.00	0.00	0.01	-0.01	0.00	0.01	0.09	0.00	-0.04
25	1	0.00	0.01	0.06	-0.01	0.00	0.01	0.09	0.02	0.16
26	6	0.01	0.00	0.01	0.01	0.00	0.00	-0.05	0.00	0.02
27	1	-0.01	0.00	0.01	0.05	0.00	0.00	-0.44	0.01	0.09
28	8	-0.05	-0.06	-0.01	-0.13	-0.14	-0.03	0.00	0.00	0.00
29	1	-0.09	0.04	0.00	0.25	-0.07	0.00	0.03	-0.31	0.01
30	1	0.07	0.02	0.01	0.22	0.09	0.05	0.03	0.02	0.01

	52	53	54
	A	A	A
Frequencies --	1179.6295	1183.7091	1208.0740
Red. masses --	1.9485	2.0676	1.5214
Frc consts --	1.5975	1.7069	1.3083
IR Inten --	66.4296	315.2288	313.3065
Raman Activ --	5.3436	0.8049	9.4591
Depolar (P) --	0.1232	0.5641	0.7485
Depolar (U) --	0.2194	0.7213	0.8562

Atom	AN	X	Y	Z	X	Y	Z	X	Y	Z
1	6	0.01	-0.01	0.00	0.02	-0.01	0.00	-0.06	0.04	0.00
2	6	0.11	-0.02	0.00	0.12	0.06	-0.01	-0.01	0.03	0.00
3	6	0.03	0.02	0.00	-0.01	-0.01	0.00	0.07	0.05	0.00
4	6	-0.03	0.02	0.00	-0.07	-0.05	0.00	0.03	0.04	0.00
5	1	-0.13	-0.02	0.00	-0.45	-0.25	0.01	0.18	0.12	0.00
6	6	0.01	-0.03	0.00	0.04	0.06	0.00	-0.01	-0.06	0.00
7	6	-0.05	0.01	0.00	-0.02	-0.04	0.00	-0.01	0.03	0.00
8	1	-0.37	0.20	-0.01	-0.10	0.01	0.01	-0.13	0.10	0.00
9	6	0.00	0.01	0.00	-0.04	-0.12	-0.01	-0.01	-0.06	-0.01
10	8	0.01	0.00	0.00	-0.05	0.01	-0.01	-0.03	0.00	0.00
11	6	0.02	0.01	0.02	0.03	-0.01	0.03	0.00	0.01	0.00
12	8	-0.03	-0.01	-0.04	-0.03	0.00	-0.05	0.00	0.00	0.00
13	8	-0.02	0.00	0.03	-0.02	0.01	0.03	0.00	0.00	0.00
14	6	-0.02	0.07	0.01	0.02	-0.07	0.00	0.01	-0.07	-0.01
15	8	-0.03	-0.01	0.00	0.04	0.00	-0.01	0.03	0.00	0.00
16	8	0.05	-0.05	-0.01	-0.05	0.04	0.01	-0.07	0.04	0.01
17	1	-0.16	0.10	0.03	0.21	-0.15	-0.04	0.35	-0.27	-0.06
18	7	-0.02	-0.01	-0.06	0.00	0.00	0.00	0.00	0.00	0.00
19	1	-0.03	-0.02	-0.07	-0.03	0.02	-0.02	0.00	0.01	0.00
20	7	0.09	0.00	-0.02	0.01	0.00	-0.01	0.00	0.00	0.00
21	1	0.28	-0.03	-0.28	0.03	0.00	-0.03	0.00	0.00	0.00
22	6	-0.08	0.01	0.09	-0.01	0.00	0.01	0.00	0.00	0.00
23	1	-0.32	0.00	0.04	-0.05	0.00	0.00	0.01	0.00	0.00

24	6	-0.09	0.01	0.05	-0.01	0.00	0.01	0.00	0.00	0.00
25	1	-0.09	-0.03	-0.19	0.00	0.00	-0.02	0.00	0.00	0.00
26	6	0.06	0.00	-0.01	0.01	0.00	0.01	0.00	0.00	0.00
27	1	0.53	-0.01	-0.09	0.06	0.00	0.00	0.00	0.00	0.00
28	8	-0.01	0.00	0.00	0.10	0.07	0.02	0.07	0.03	0.01
29	1	0.00	-0.30	0.01	0.06	0.63	-0.01	-0.03	-0.72	0.00
30	1	0.05	0.04	0.01	-0.34	-0.21	-0.08	-0.33	-0.22	-0.07

	55	56	57
	A	A	A
Frequencies --	1217.4363	1231.9354	1256.0451
Red. masses --	3.6666	1.7996	1.7240
Frc consts --	3.2019	1.6092	1.6025
IR Inten --	41.5219	18.2754	0.7600
Raman Activ --	38.9926	27.3819	1.8933
Depolar (P) --	0.0607	0.1140	0.5802
Depolar (U) --	0.1144	0.2047	0.7343

Atom	AN	X	Y	Z	X	Y	Z	X	Y	Z
1	6	0.00	0.02	0.00	-0.10	0.13	0.00	0.13	-0.01	0.00
2	6	0.03	0.00	0.00	0.00	-0.01	0.00	-0.01	0.11	0.00
3	6	-0.01	-0.02	0.00	-0.07	-0.11	0.00	-0.14	-0.03	0.00
4	6	-0.01	0.00	0.00	0.00	0.00	0.00	0.04	-0.04	0.00
5	1	-0.03	-0.01	0.00	0.17	0.08	-0.01	0.67	0.28	-0.01
6	6	0.01	0.00	0.00	0.08	0.00	0.00	-0.01	-0.02	0.00
7	6	-0.01	0.00	0.00	0.00	0.02	0.00	-0.03	-0.05	0.00
8	1	-0.03	0.01	0.00	0.28	-0.14	0.02	-0.54	0.25	-0.01
9	6	0.00	0.00	0.00	0.00	-0.03	0.00	-0.02	-0.05	0.00
10	8	0.00	0.00	0.00	-0.03	-0.01	-0.01	-0.02	0.01	0.00
11	6	-0.01	0.00	-0.01	0.00	0.00	0.00	-0.01	-0.04	0.00
12	8	0.00	0.00	0.00	0.00	0.00	0.01	0.00	0.00	0.00
13	8	0.00	0.00	0.00	0.00	0.00	0.00	0.00	0.00	-0.01
14	6	0.00	0.01	0.00	0.00	0.03	0.00	0.02	-0.05	0.00
15	8	0.00	0.00	0.00	-0.03	0.01	0.00	0.02	0.02	0.00
16	8	0.01	0.00	0.00	0.08	-0.02	-0.01	0.00	0.02	0.00
17	1	-0.05	0.04	0.01	-0.47	0.38	0.09	-0.14	0.12	0.03
18	7	0.13	-0.02	-0.16	-0.01	0.00	0.01	0.00	0.00	0.00
19	1	-0.02	0.00	-0.01	0.00	-0.01	-0.01	0.00	0.03	0.00
20	7	0.06	0.04	0.27	0.00	0.00	-0.01	0.00	0.00	0.00
21	1	-0.02	0.06	0.44	0.00	0.00	-0.02	0.00	0.00	0.00
22	6	0.01	0.01	0.11	0.00	0.00	0.00	0.00	0.00	0.00
23	1	0.54	0.04	0.28	-0.03	0.00	-0.01	0.00	0.00	0.00
24	6	-0.17	-0.01	-0.05	0.01	0.00	0.00	0.00	0.00	0.00
25	1	-0.20	-0.01	-0.03	0.01	0.00	-0.01	0.00	0.00	0.00
26	6	-0.06	-0.03	-0.22	0.00	0.00	0.01	0.00	0.00	0.00
27	1	-0.34	-0.03	-0.21	0.03	0.00	0.01	0.00	0.00	0.00
28	8	0.01	0.00	0.00	0.08	0.01	0.02	0.01	0.02	0.00
29	1	0.02	0.01	0.00	0.08	-0.06	0.00	0.00	0.11	0.00
30	1	-0.04	-0.03	-0.01	-0.52	-0.37	-0.12	0.04	0.04	0.01

	58	59	60
	A	A	A
Frequencies --	1320.6289	1332.8494	1368.3502
Red. masses --	1.6927	5.9039	3.8322
Frc consts --	1.7394	6.1794	4.2276
IR Inten --	34.3351	0.6540	271.9121
Raman Activ --	31.6668	12.4888	5.6087
Depolar (P) --	0.2042	0.7499	0.0687
Depolar (U) --	0.3392	0.8571	0.1285

Atom	AN	X	Y	Z	X	Y	Z	X	Y	Z
1	6	0.00	0.00	0.00	-0.26	-0.14	0.00	-0.08	-0.06	0.00
2	6	0.00	0.00	0.00	0.00	0.32	-0.01	-0.10	0.00	-0.02
3	6	0.00	0.00	0.00	0.25	-0.16	0.00	-0.09	0.07	0.01

4	6	0.00	0.00	0.00	-0.19	-0.10	0.00	0.05	-0.05	0.00
5	1	-0.01	0.00	0.00	0.18	0.08	0.00	0.30	0.08	-0.01
6	6	0.00	0.00	0.00	0.00	0.23	0.00	0.02	0.00	0.00
7	6	0.00	0.00	0.00	0.19	-0.12	0.00	0.03	0.05	0.00
8	1	-0.01	0.00	0.00	-0.15	0.08	-0.01	0.33	-0.12	0.00
9	6	0.00	0.00	0.00	-0.01	-0.05	-0.01	-0.01	-0.02	-0.01
10	8	0.00	0.00	0.00	0.05	0.00	0.01	0.02	0.00	0.00
11	6	0.00	0.00	-0.01	0.00	-0.04	0.01	0.31	0.01	0.16
12	8	0.00	0.00	0.00	0.00	0.00	0.00	-0.09	-0.03	-0.21
13	8	0.00	0.00	0.01	0.00	0.01	0.00	-0.07	0.01	0.07
14	6	0.00	0.00	0.00	0.01	-0.05	-0.01	0.00	-0.02	0.00
15	8	0.00	0.00	0.00	-0.05	0.00	0.01	0.01	0.00	0.00
16	8	0.00	0.00	0.00	0.04	0.03	0.00	-0.01	0.00	0.00
17	1	-0.02	0.01	0.00	-0.31	0.28	0.06	0.02	-0.02	0.00
18	7	0.08	0.02	0.13	0.00	0.00	0.00	0.02	0.00	0.00
19	1	-0.01	0.00	0.01	0.00	0.05	0.00	-0.01	0.09	0.70
20	7	0.05	-0.01	-0.07	0.00	0.00	0.00	0.02	0.00	-0.01
21	1	0.38	-0.06	-0.52	-0.01	0.01	0.01	-0.03	0.01	0.06
22	6	-0.11	0.00	0.05	0.00	0.00	0.00	0.01	0.00	0.01
23	1	0.09	0.01	0.11	0.00	0.00	0.00	-0.10	0.00	-0.02
24	6	-0.05	-0.01	-0.06	0.00	0.00	0.00	-0.03	0.00	0.02
25	1	-0.09	0.04	0.30	0.00	0.00	-0.01	-0.03	-0.01	-0.10
26	6	0.03	-0.01	-0.06	0.00	0.00	0.00	-0.01	0.00	-0.03
27	1	-0.64	0.00	0.04	0.01	0.00	0.00	-0.05	-0.01	-0.03
28	8	0.00	0.00	0.00	-0.04	0.03	0.00	-0.01	0.01	0.00
29	1	0.00	0.00	0.00	-0.02	-0.40	0.00	0.03	-0.04	0.00
30	1	-0.01	-0.01	0.00	0.30	0.24	0.07	0.09	0.08	0.02

		61			62			63		
		A			A			A		
Frequencies --		1381.2525			1392.8422			1416.2379		
Red. masses --		2.5524			2.8170			2.9169		
Frc consts --		2.8691			3.2198			3.4470		
IR Inten --		231.0886			6.8511			76.1215		
Raman Activ --		1.1551			19.9617			9.8404		
Depolar (P) --		0.7500			0.1993			0.1513		
Depolar (U) --		0.8571			0.3324			0.2628		
Atom	AN	X	Y	Z	X	Y	Z	X	Y	Z
1	6	0.00	-0.07	0.00	-0.09	0.07	0.00	0.02	0.02	0.00
2	6	-0.02	0.01	0.00	0.07	0.01	0.00	0.01	0.00	0.00
3	6	0.00	-0.06	0.00	-0.10	-0.08	0.00	0.02	-0.02	0.00
4	6	-0.07	-0.04	0.00	-0.03	-0.03	0.00	-0.01	0.01	0.00
5	1	0.25	0.13	-0.01	0.30	0.14	-0.01	-0.06	-0.02	0.00
6	6	-0.01	0.05	0.00	0.08	0.01	0.00	0.00	0.00	0.00
7	6	0.08	-0.05	0.00	-0.02	0.02	0.00	0.00	-0.01	0.00
8	1	-0.24	0.15	-0.01	0.22	-0.12	0.01	-0.06	0.02	0.00
9	6	0.05	0.23	0.02	-0.04	-0.19	-0.02	0.00	-0.01	0.00
10	8	-0.05	-0.03	-0.01	0.05	0.02	0.01	0.00	0.00	0.00
11	6	0.02	0.00	0.01	-0.03	0.00	-0.01	-0.06	0.00	-0.04
12	8	-0.01	0.00	-0.01	0.01	0.00	0.02	0.02	0.00	0.01
13	8	0.00	0.00	0.00	0.00	0.00	-0.01	0.01	0.00	0.00
14	6	-0.03	0.20	0.01	-0.04	0.23	0.02	0.00	0.01	0.00
15	8	0.04	-0.03	-0.01	0.06	-0.03	-0.01	0.00	0.00	0.00
16	8	-0.02	-0.06	0.00	-0.03	-0.07	0.00	0.00	0.00	0.00
17	1	0.39	-0.35	-0.07	0.46	-0.41	-0.09	0.00	0.00	0.00
18	7	0.00	0.00	0.00	0.00	0.00	0.01	0.16	0.02	0.15
19	1	0.00	0.01	0.05	-0.01	-0.01	-0.11	0.02	0.02	0.19
20	7	0.00	0.00	0.00	0.00	0.00	0.00	0.01	-0.02	-0.14
21	1	0.00	0.00	0.01	0.01	0.00	-0.02	-0.31	0.04	0.31
22	6	0.00	0.00	0.00	-0.01	0.00	0.00	0.13	0.01	0.08
23	1	-0.01	0.00	0.00	0.01	0.00	0.01	-0.62	-0.02	-0.12

24	6	0.00	0.00	0.00	0.00	0.00	0.00	-0.08	0.01	0.07
25	1	0.00	0.00	-0.01	0.00	0.00	0.01	-0.06	-0.05	-0.39
26	6	0.00	0.00	0.00	0.00	0.00	0.00	-0.18	-0.02	-0.13
27	1	0.00	0.00	0.00	-0.01	0.00	0.00	0.07	-0.03	-0.20
28	8	0.02	-0.07	0.00	-0.02	0.06	0.00	0.00	0.00	0.00
29	1	0.00	0.17	0.00	0.09	-0.01	0.00	-0.01	0.01	0.00
30	1	-0.49	-0.38	-0.11	0.40	0.31	0.09	0.01	0.00	0.00

64 65 66
A A A
Frequencies -- 1454.4942 1502.0561 1505.5010
Red. masses -- 4.1260 2.5939 2.4600
Frc consts -- 5.1429 3.4480 3.2851
IR Inten -- 24.3309 3.8038 3.6320
Raman Activ -- 34.3836 4.3616 8.3585
Depolar (P) -- 0.6153 0.2903 0.2939
Depolar (U) -- 0.7618 0.4499 0.4543

Atom	AN	X	Y	Z	X	Y	Z	X	Y	Z
1	6	0.01	0.01	0.00	-0.06	0.17	0.00	-0.14	-0.09	0.00
2	6	0.00	0.00	0.00	-0.09	-0.09	0.00	0.14	-0.06	0.00
3	6	0.01	-0.01	0.00	0.15	-0.01	0.00	-0.01	0.18	0.00
4	6	0.00	0.01	0.00	-0.09	0.07	0.00	-0.11	-0.05	0.00
5	1	-0.03	-0.01	0.00	0.11	0.20	0.00	0.49	0.27	-0.01
6	6	0.00	0.00	0.00	-0.04	-0.17	0.00	0.05	-0.10	0.00
7	6	0.00	-0.01	0.00	0.15	-0.02	0.00	0.04	0.08	0.00
8	1	-0.03	0.01	0.00	-0.42	0.34	-0.01	0.13	0.03	0.00
9	6	0.00	-0.01	0.00	-0.02	-0.07	-0.01	0.00	0.05	0.00
10	8	0.00	0.00	0.00	0.02	0.00	0.00	0.02	-0.01	0.00
11	6	-0.02	0.00	0.00	0.01	0.02	-0.02	0.00	0.02	0.03
12	8	0.01	0.00	0.01	0.02	-0.01	-0.02	-0.03	0.00	0.03
13	8	0.01	0.00	-0.01	-0.01	0.00	0.02	0.00	-0.01	-0.02
14	6	0.00	0.00	0.00	0.00	0.01	0.00	0.01	-0.08	0.00
15	8	0.00	0.00	0.00	-0.02	-0.01	0.00	-0.01	0.01	0.00
16	8	0.00	0.00	0.00	0.00	0.00	0.00	0.00	0.01	0.00
17	1	0.00	0.00	0.00	-0.01	0.01	0.00	-0.05	0.04	0.01
18	7	-0.12	0.01	0.06	-0.01	0.00	-0.02	0.02	0.00	0.03
19	1	0.01	0.00	0.03	0.03	0.03	0.38	-0.05	-0.09	-0.63
20	7	0.07	0.00	0.00	0.00	0.00	0.01	0.00	0.00	-0.01
21	1	-0.11	0.03	0.26	0.04	-0.01	-0.05	-0.07	0.01	0.08
22	6	-0.09	-0.03	-0.20	0.00	0.00	0.00	0.01	0.00	0.00
23	1	0.21	-0.02	-0.15	0.02	0.00	0.00	-0.04	0.00	-0.01
24	6	-0.02	0.05	0.36	0.00	0.00	-0.01	0.00	0.00	0.01
25	1	0.04	-0.07	-0.54	0.00	0.00	0.02	0.00	0.00	-0.03
26	6	0.20	-0.02	-0.19	0.01	0.00	0.01	-0.01	0.00	-0.02
27	1	-0.51	-0.02	-0.11	0.01	0.00	0.01	-0.02	0.00	-0.02
28	8	0.00	0.00	0.00	0.00	0.01	0.00	0.00	-0.01	0.00
29	1	0.00	0.00	0.00	-0.02	0.60	-0.01	0.08	0.33	0.00
30	1	0.00	0.00	0.00	0.06	0.04	0.02	-0.02	-0.01	0.00

67 68 69
A A A
Frequencies -- 1537.6826 1546.9871 1590.9958
Red. masses -- 2.1452 2.5815 2.6627
Frc consts -- 2.9884 3.6399 3.9712
IR Inten -- 18.1301 60.2346 18.4715
Raman Activ -- 10.2354 3.9435 8.2400
Depolar (P) -- 0.7498 0.2019 0.6157
Depolar (U) -- 0.8570 0.3359 0.7622

Atom	AN	X	Y	Z	X	Y	Z	X	Y	Z
1	6	-0.03	-0.06	0.00	0.01	0.04	0.00	0.00	0.01	0.00
2	6	0.09	0.00	0.00	-0.07	0.00	0.00	-0.02	0.00	0.00
3	6	-0.02	0.06	0.00	0.01	-0.04	0.00	0.00	-0.01	0.00

4	6	-0.04	-0.03	0.00	0.03	0.02	0.00	0.01	0.01	0.00
5	1	0.15	0.06	0.00	-0.10	-0.05	0.00	-0.02	-0.01	0.00
6	6	0.03	0.00	0.00	-0.03	0.00	0.00	-0.01	0.00	0.00
7	6	-0.03	0.03	0.00	0.03	-0.02	0.00	0.01	-0.01	0.00
8	1	0.13	-0.06	0.00	-0.09	0.05	0.00	-0.02	0.01	0.00
9	6	0.01	0.02	0.00	-0.01	-0.02	0.00	0.00	0.00	0.00
10	8	0.00	0.00	0.00	0.00	0.00	0.00	0.00	0.00	0.00
11	6	-0.11	-0.02	-0.12	0.10	0.01	0.08	0.05	0.00	-0.01
12	8	0.06	0.00	0.00	-0.05	0.00	0.01	-0.01	0.00	0.00
13	8	-0.01	0.01	0.06	0.00	0.00	-0.03	-0.01	0.00	0.01
14	6	0.01	-0.02	0.00	0.00	0.02	0.00	0.00	0.00	0.00
15	8	0.00	0.00	0.00	0.00	0.00	0.00	0.00	0.00	0.00
16	8	0.00	0.00	0.00	0.00	0.00	0.00	0.00	0.00	0.00
17	1	-0.01	0.01	0.00	0.01	-0.01	0.00	0.00	0.00	0.00
18	7	-0.01	0.00	-0.04	0.00	0.00	0.00	0.16	0.00	0.01
19	1	0.06	0.10	0.75	-0.07	-0.09	-0.67	-0.05	-0.03	-0.20
20	7	-0.12	0.01	0.06	-0.16	0.01	0.06	-0.08	0.01	0.09
21	1	0.23	-0.06	-0.44	0.14	-0.05	-0.42	0.40	-0.07	-0.58
22	6	0.12	0.00	-0.03	0.22	0.00	0.01	-0.09	-0.02	-0.14
23	1	-0.10	-0.01	-0.09	-0.29	-0.02	-0.13	0.29	-0.01	-0.08
24	6	-0.05	0.00	0.03	-0.11	0.00	0.01	0.10	0.02	0.13
25	1	-0.05	-0.01	-0.08	-0.12	-0.02	-0.12	0.16	-0.02	-0.20
26	6	0.06	0.00	0.00	0.12	0.00	-0.03	-0.21	-0.01	-0.02
27	1	-0.05	0.00	0.02	-0.23	0.00	0.01	0.38	-0.01	-0.13
28	8	0.00	0.00	0.00	0.00	0.00	0.00	0.00	0.00	0.00
29	1	0.04	0.00	0.00	-0.03	0.00	0.00	-0.01	0.00	0.00
30	1	-0.01	-0.01	0.00	0.01	0.01	0.00	0.00	0.00	0.00

	70	71	72
	A	A	A
Frequencies --	1657.2191	1666.9224	1801.8087
Red. masses --	7.5034	6.3713	7.6991
Frc consts --	12.1413	10.4305	14.7268
IR Inten --	67.8906	6.6709	276.2578
Raman Activ --	40.1278	19.8467	24.4109
Depolar (P) --	0.7500	0.7500	0.2354
Depolar (U) --	0.8571	0.8571	0.3810

Atom	AN	X	Y	Z	X	Y	Z	X	Y	Z
1	6	0.05	0.26	0.00	-0.29	-0.04	0.00	-0.02	0.00	0.00
2	6	0.00	-0.35	0.01	0.18	0.01	0.00	0.06	-0.01	-0.02
3	6	-0.07	0.26	0.00	-0.28	0.03	0.00	-0.02	0.00	0.00
4	6	-0.10	-0.23	0.00	0.33	0.13	0.00	0.01	0.00	0.00
5	1	0.28	-0.06	0.00	-0.43	-0.28	0.01	-0.02	-0.01	0.00
6	6	0.00	0.42	0.00	-0.18	-0.01	0.00	0.00	0.00	0.00
7	6	0.13	-0.25	0.00	0.31	-0.12	0.00	0.01	-0.01	0.00
8	1	-0.32	-0.01	0.00	-0.34	0.29	-0.01	-0.02	0.01	0.00
9	6	0.01	-0.05	0.00	-0.02	0.02	-0.01	0.05	-0.01	0.01
10	8	-0.01	0.01	0.00	0.04	-0.01	0.01	-0.03	0.00	-0.01
11	6	0.00	0.04	-0.01	-0.04	-0.01	-0.05	-0.30	0.09	0.54
12	8	0.00	0.00	0.00	0.01	0.00	0.01	-0.01	-0.01	-0.10
13	8	0.00	-0.01	0.01	-0.01	0.00	0.02	0.19	-0.05	-0.32
14	6	-0.02	-0.05	0.00	-0.02	-0.01	0.01	0.04	0.00	0.00
15	8	0.01	0.01	0.00	0.04	0.01	-0.01	-0.02	0.00	0.00
16	8	0.00	0.00	0.00	0.00	0.00	0.00	0.00	0.00	0.00
17	1	-0.03	0.02	0.01	-0.01	0.00	0.00	0.02	-0.01	0.00
18	7	0.00	0.00	0.00	-0.01	0.00	0.00	0.03	0.00	0.00
19	1	0.00	-0.04	0.01	0.01	0.01	0.05	0.14	0.07	0.57
20	7	0.00	0.00	0.00	0.01	0.00	-0.01	-0.01	0.00	0.03
21	1	0.00	-0.01	0.01	-0.02	0.01	0.04	0.13	-0.04	-0.28
22	6	0.00	0.00	0.00	0.00	0.00	0.01	0.01	0.00	-0.02
23	1	0.00	0.00	0.00	-0.01	0.00	0.00	0.03	0.00	-0.02

24	6	0.00	0.00	0.00	0.00	0.00	0.00	0.00	0.00	0.00
25	1	0.00	0.00	0.00	0.00	0.00	0.01	0.00	0.00	-0.02
26	6	0.00	0.00	0.00	0.01	0.00	0.00	-0.02	0.00	0.00
27	1	0.00	0.00	0.00	-0.01	0.00	0.00	0.02	0.00	-0.01
28	8	0.00	0.00	0.00	0.00	0.00	0.00	0.00	0.00	0.00
29	1	-0.03	-0.47	0.00	-0.21	0.03	0.00	-0.01	0.00	0.00
30	1	0.03	0.02	0.01	-0.01	0.00	0.00	0.02	0.02	0.01

	73	74	75
	A	A	A
Frequencies --	1847.9354	1861.1166	3028.6340
Red. masses --	10.4904	10.4408	1.0745
Frc consts --	21.1064	21.3074	5.8072
IR Inten --	29.5365	744.7051	2777.4662
Raman Activ --	25.6960	95.0616	487.5985
Depolar (P) --	0.7500	0.0956	0.3339
Depolar (U) --	0.8571	0.1746	0.5007

Atom	AN	X	Y	Z	X	Y	Z	X	Y	Z
1	6	0.06	-0.01	0.00	-0.08	0.03	-0.01	0.00	0.00	0.00
2	6	0.00	-0.01	0.00	0.03	0.00	0.00	0.00	0.00	0.00
3	6	-0.06	-0.01	0.00	-0.08	-0.02	0.00	0.00	0.00	0.00
4	6	0.00	-0.02	0.00	0.02	0.00	0.00	0.00	0.00	0.00
5	1	0.01	-0.03	0.00	-0.01	-0.03	0.00	0.00	0.00	0.00
6	6	0.00	0.03	0.00	-0.01	0.00	0.00	0.00	0.00	0.00
7	6	0.00	-0.02	0.00	0.02	0.00	0.00	0.00	0.00	0.00
8	1	-0.01	-0.03	0.00	-0.01	0.03	0.00	0.00	0.00	0.00
9	6	-0.50	0.11	-0.09	0.50	-0.11	0.09	0.00	0.00	0.00
10	8	0.32	-0.05	0.06	-0.31	0.05	-0.06	0.00	0.00	0.00
11	6	0.00	0.02	0.01	0.05	-0.01	-0.05	0.00	0.00	-0.01
12	8	0.00	-0.01	0.00	-0.01	0.00	0.01	-0.06	0.00	0.01
13	8	0.00	-0.01	0.00	-0.03	0.01	0.04	0.00	0.00	0.01
14	6	0.52	0.07	-0.07	0.51	0.08	-0.07	0.00	0.00	0.00
15	8	-0.33	-0.03	0.05	-0.32	-0.03	0.05	0.00	0.00	0.00
16	8	-0.05	0.00	0.01	-0.05	0.00	0.01	0.00	0.00	0.00
17	1	0.25	-0.21	-0.05	0.24	-0.20	-0.05	0.00	0.00	0.00
18	7	0.00	0.00	0.00	0.00	0.00	0.00	0.00	0.00	0.00
19	1	0.00	0.04	0.00	-0.02	-0.01	-0.09	0.99	-0.02	-0.04
20	7	0.00	0.00	0.00	0.00	0.00	0.00	0.01	0.00	0.01
21	1	0.00	0.02	-0.01	-0.02	0.01	0.04	-0.04	-0.01	-0.05
22	6	0.00	0.00	0.00	0.00	0.00	0.00	0.00	0.00	0.00
23	1	0.00	0.00	0.00	0.00	0.00	0.00	0.00	0.00	0.00
24	6	0.00	0.00	0.00	0.00	0.00	0.00	0.00	0.00	0.00
25	1	0.00	0.00	0.00	0.00	0.00	0.00	0.00	0.00	0.00
26	6	0.00	0.00	0.00	0.00	0.00	0.00	0.00	0.00	0.00
27	1	0.00	0.00	0.00	0.00	0.00	0.00	0.00	0.00	-0.02
28	8	0.04	0.00	0.01	-0.04	0.00	-0.01	0.00	0.00	0.00
29	1	0.00	-0.03	0.00	-0.01	0.00	0.00	0.00	0.00	0.00
30	1	-0.26	-0.19	-0.06	0.25	0.19	0.06	0.00	0.00	0.00

	76	77	78
	A	A	A
Frequencies --	3215.7144	3238.2455	3247.8452
Red. masses --	1.0893	1.0938	1.0949
Frc consts --	6.6365	6.7576	6.8046
IR Inten --	4.5602	1.3300	1.7735
Raman Activ --	82.5126	103.0388	145.8926
Depolar (P) --	0.5224	0.2958	0.1529
Depolar (U) --	0.6863	0.4566	0.2652

Atom	AN	X	Y	Z	X	Y	Z	X	Y	Z
1	6	0.00	0.00	0.00	0.00	0.00	0.00	0.00	0.00	0.00
2	6	0.00	0.00	0.00	0.00	0.00	0.00	0.00	0.00	0.00
3	6	0.00	0.00	0.00	0.00	0.00	0.00	0.00	0.00	0.00

4	6	0.01	-0.01	0.00	0.01	-0.02	0.00	0.04	-0.07	0.00
5	1	-0.11	0.20	0.00	-0.12	0.23	0.00	-0.43	0.83	0.00
6	6	-0.08	0.00	0.00	-0.03	0.01	0.00	0.03	0.00	0.00
7	6	0.02	0.02	0.00	-0.04	-0.07	0.00	0.01	0.01	0.00
8	1	-0.18	-0.29	0.00	0.48	0.79	-0.01	-0.09	-0.15	0.00
9	6	0.00	0.00	0.00	0.00	0.00	0.00	0.00	0.00	0.00
10	8	0.00	0.00	0.00	0.00	0.00	0.00	0.00	0.00	0.00
11	6	0.00	0.00	0.00	0.00	0.00	0.00	0.00	0.00	0.00
12	8	0.00	0.00	0.00	0.00	0.00	0.00	0.00	0.00	0.00
13	8	0.00	0.00	0.00	0.00	0.00	0.00	0.00	0.00	0.00
14	6	0.00	0.00	0.00	0.00	0.00	0.00	0.00	0.00	0.00
15	8	0.00	0.00	0.00	0.00	0.00	0.00	0.00	0.00	0.00
16	8	0.00	0.00	0.00	0.00	0.00	0.00	0.00	0.00	0.00
17	1	0.00	0.00	0.00	0.00	0.00	0.00	0.00	0.00	0.00
18	7	0.00	0.00	0.00	0.00	0.00	0.00	0.00	0.00	0.00
19	1	0.00	0.00	0.00	0.00	0.00	0.00	0.00	0.00	0.00
20	7	0.00	0.00	0.00	0.00	0.00	0.00	0.00	0.00	0.00
21	1	0.00	0.00	0.00	0.00	0.00	0.00	0.00	0.00	0.00
22	6	0.00	0.00	0.00	0.00	0.00	0.00	0.00	0.00	0.00
23	1	0.00	0.00	0.00	0.00	0.00	0.00	0.00	0.00	0.00
24	6	0.00	0.00	0.00	0.00	0.00	0.00	0.00	0.00	0.00
25	1	0.00	0.00	0.00	0.00	0.00	0.00	0.00	0.00	0.00
26	6	0.00	0.00	0.00	0.00	0.00	0.00	0.00	0.00	0.00
27	1	0.00	0.00	0.00	0.00	0.00	0.00	0.00	0.00	0.00
28	8	0.00	0.00	0.00	0.00	0.00	0.00	0.00	0.00	0.00
29	1	0.91	-0.03	0.00	0.27	-0.01	0.00	-0.30	0.01	0.00
30	1	0.00	0.00	0.00	0.00	0.00	0.00	0.00	0.00	0.00

	79	80	81
	A	A	A
Frequencies --	3266.6606	3281.1495	3297.7933
Red. masses --	1.0936	1.0957	1.1083
Frc consts --	6.8755	6.9500	7.1013
IR Inten --	2.5810	0.6993	0.0027
Raman Activ --	79.4813	57.1616	173.4142
Depolar (P) --	0.5629	0.5974	0.1211
Depolar (U) --	0.7204	0.7480	0.2160

Atom	AN	X	Y	Z	X	Y	Z	X	Y	Z
1	6	0.00	0.00	0.00	0.00	0.00	0.00	0.00	0.00	0.00
2	6	0.00	0.00	0.00	0.00	0.00	0.00	0.00	0.00	0.00
3	6	0.00	0.00	0.00	0.00	0.00	0.00	0.00	0.00	0.00
4	6	0.00	0.00	0.00	0.00	0.00	0.00	0.00	0.00	0.00
5	1	0.00	0.00	0.00	0.00	0.00	0.00	0.00	0.00	0.00
6	6	0.00	0.00	0.00	0.00	0.00	0.00	0.00	0.00	0.00
7	6	0.00	0.00	0.00	0.00	0.00	0.00	0.00	0.00	0.00
8	1	0.00	0.00	0.00	0.00	0.00	0.00	0.00	0.00	0.00
9	6	0.00	0.00	0.00	0.00	0.00	0.00	0.00	0.00	0.00
10	8	0.00	0.00	0.00	0.00	0.00	0.00	0.00	0.00	0.00
11	6	0.00	0.00	0.00	0.00	0.00	0.00	0.00	0.00	0.00
12	8	0.00	0.00	0.00	0.00	0.00	0.00	0.00	0.00	0.00
13	8	0.00	0.00	0.00	0.00	0.00	0.00	0.00	0.00	0.00
14	6	0.00	0.00	0.00	0.00	0.00	0.00	0.00	0.00	0.00
15	8	0.00	0.00	0.00	0.00	0.00	0.00	0.00	0.00	0.00
16	8	0.00	0.00	0.00	0.00	0.00	0.00	0.00	0.00	0.00
17	1	0.00	0.00	0.00	0.00	0.00	0.00	0.00	0.00	0.00
18	7	0.00	0.00	0.00	0.00	0.00	0.00	0.00	0.00	0.00
19	1	-0.02	0.00	0.00	0.01	0.00	0.00	0.01	0.00	0.00
20	7	0.00	0.00	0.00	0.00	0.00	0.00	0.00	0.00	0.00
21	1	0.00	0.00	0.00	0.00	0.00	-0.01	0.01	0.00	0.01
22	6	0.00	0.00	-0.01	0.02	-0.01	-0.06	-0.01	0.01	0.06
23	1	-0.04	0.02	0.14	-0.18	0.09	0.67	0.18	-0.08	-0.66

24	6	-0.04	0.00	-0.01	-0.05	0.00	0.00	-0.07	0.00	-0.01
25	1	0.42	0.01	0.03	0.59	0.01	0.04	0.68	0.02	0.05
26	6	0.01	0.01	0.08	0.00	0.00	-0.04	0.00	0.00	-0.02
27	1	-0.14	-0.12	-0.87	0.06	0.05	0.38	0.03	0.03	0.21
28	8	0.00	0.00	0.00	0.00	0.00	0.00	0.00	0.00	0.00
29	1	0.00	0.00	0.00	0.00	0.00	0.00	0.00	0.00	0.00
30	1	0.00	0.00	0.00	0.00	0.00	0.00	0.00	0.00	0.00

	82	83	84
	A	A	A
Frequencies --	3477.0674	3852.3619	3853.6365
Red. masses --	1.0874	1.0647	1.0648
Frc consts --	7.7458	9.3100	9.3165
IR Inten --	972.0908	148.0809	90.7402
Raman Activ --	186.0570	92.1618	153.9378
Depolar (P) --	0.2455	0.2643	0.2543
Depolar (U) --	0.3942	0.4181	0.4055

Atom	AN	X	Y	Z	X	Y	Z	X	Y	Z
1	6	0.00	0.00	0.00	0.00	0.00	0.00	0.00	0.00	0.00
2	6	0.00	0.00	0.00	0.00	0.00	0.00	0.00	0.00	0.00
3	6	0.00	0.00	0.00	0.00	0.00	0.00	0.00	0.00	0.00
4	6	0.00	0.00	0.00	0.00	0.00	0.00	0.00	0.00	0.00
5	1	0.00	0.00	0.00	0.00	0.00	0.00	0.00	0.00	0.00
6	6	0.00	0.00	0.00	0.00	0.00	0.00	0.00	0.00	0.00
7	6	0.00	0.00	0.00	0.00	0.00	0.00	0.00	0.00	0.00
8	1	0.00	0.00	0.00	0.00	0.00	0.00	0.00	0.00	0.00
9	6	0.00	0.00	0.00	0.00	0.00	0.00	0.00	0.00	0.00
10	8	0.00	0.00	0.00	0.00	0.00	0.00	0.00	0.00	0.00
11	6	0.00	0.00	0.00	0.00	0.00	0.00	0.00	0.00	0.00
12	8	0.00	0.00	0.00	0.00	0.00	0.00	0.00	0.00	0.00
13	8	0.00	0.00	0.00	0.00	0.00	0.00	0.00	0.00	0.00
14	6	0.00	0.00	0.00	0.00	0.00	0.00	0.00	0.00	0.00
15	8	0.00	0.00	0.00	0.00	0.00	0.00	0.00	0.00	0.00
16	8	0.00	0.00	0.00	0.00	-0.01	0.00	-0.04	-0.05	0.00
17	1	0.00	0.00	0.00	0.08	0.11	-0.01	0.57	0.81	-0.05
18	7	0.00	0.00	0.00	0.00	0.00	0.00	0.00	0.00	0.00
19	1	0.06	0.00	0.00	0.00	0.00	0.00	0.00	0.00	0.00
20	7	-0.07	-0.01	-0.04	0.00	0.00	0.00	0.00	0.00	0.00
21	1	0.82	0.08	0.56	0.00	0.00	0.00	0.00	0.00	0.00
22	6	0.00	0.00	0.00	0.00	0.00	0.00	0.00	0.00	0.00
23	1	-0.01	0.00	0.01	0.00	0.00	0.00	0.00	0.00	0.00
24	6	0.00	0.00	0.00	0.00	0.00	0.00	0.00	0.00	0.00
25	1	0.00	0.00	0.00	0.00	0.00	0.00	0.00	0.00	0.00
26	6	0.00	0.00	0.00	0.00	0.00	0.00	0.00	0.00	0.00
27	1	0.00	0.00	0.00	0.00	0.00	0.00	0.00	0.00	0.00
28	8	0.00	0.00	0.00	0.03	-0.05	0.00	0.00	0.01	0.00
29	1	0.00	0.00	0.00	0.00	0.00	0.00	0.00	0.00	0.00
30	1	0.00	0.00	0.00	-0.51	0.84	-0.07	0.07	-0.12	0.01
